# Enantioselective Synthesis of α‐Aryl‐β^2^‐Amino‐Esters by Cooperative Isothiourea and Brønsted Acid Catalysis

**DOI:** 10.1002/anie.202016220

**Published:** 2021-05-04

**Authors:** Feng Zhao, Chang Shu, Claire M. Young, Cameron Carpenter‐Warren, Alexandra M. Z. Slawin, Andrew D. Smith

**Affiliations:** ^1^ EaStCHEM School of Chemistry University of St Andrews St Andrews Fife KY16 9ST UK

**Keywords:** aminomethylation, cooperative catalysis, isothiourea, mechanistic studies, α-aryl-β-amino acid

## Abstract

The synthesis of α‐aryl‐β^2^‐amino esters through enantioselective aminomethylation of an arylacetic acid ester in high yields and enantioselectivity via cooperative isothiourea and Brønsted acid catalysis is demonstrated. The scope and limitations of this process are explored (25 examples, up to 94 % yield and 96:4 er), with applications to the synthesis of (*S*)‐Venlafaxine⋅HCl and (*S*)‐Nakinadine B. Mechanistic studies are consistent with a C(1)‐ammonium enolate pathway being followed rather than an alternative dynamic kinetic resolution process. Control studies indicate that (i) a linear effect between catalyst and product er is observed; (ii) an acyl ammonium ion can be used as a precatalyst; (iii) reversible isothiourea addition to an in situ generated iminium ion leads to an off‐cycle intermediate that can be used as a productive precatalyst.

## Graphical abstract

An operationally simple and effective catalytic enantioselective approach to α‐aryl‐β^2^‐amino esters is demonstrated and applied to the synthesis of (*S*)‐Venlafaxine⋅HCl and (*S*)‐Nakinadine B. Mechanistic investigations support a C(1)‐ammonium enolate pathway with reversible isothiourea addition to an in situ generated iminium ion leading to an off‐cycle intermediate that can be used as a productive precatalyst.

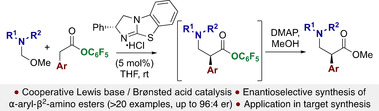

## Introduction

**Figure 1:**
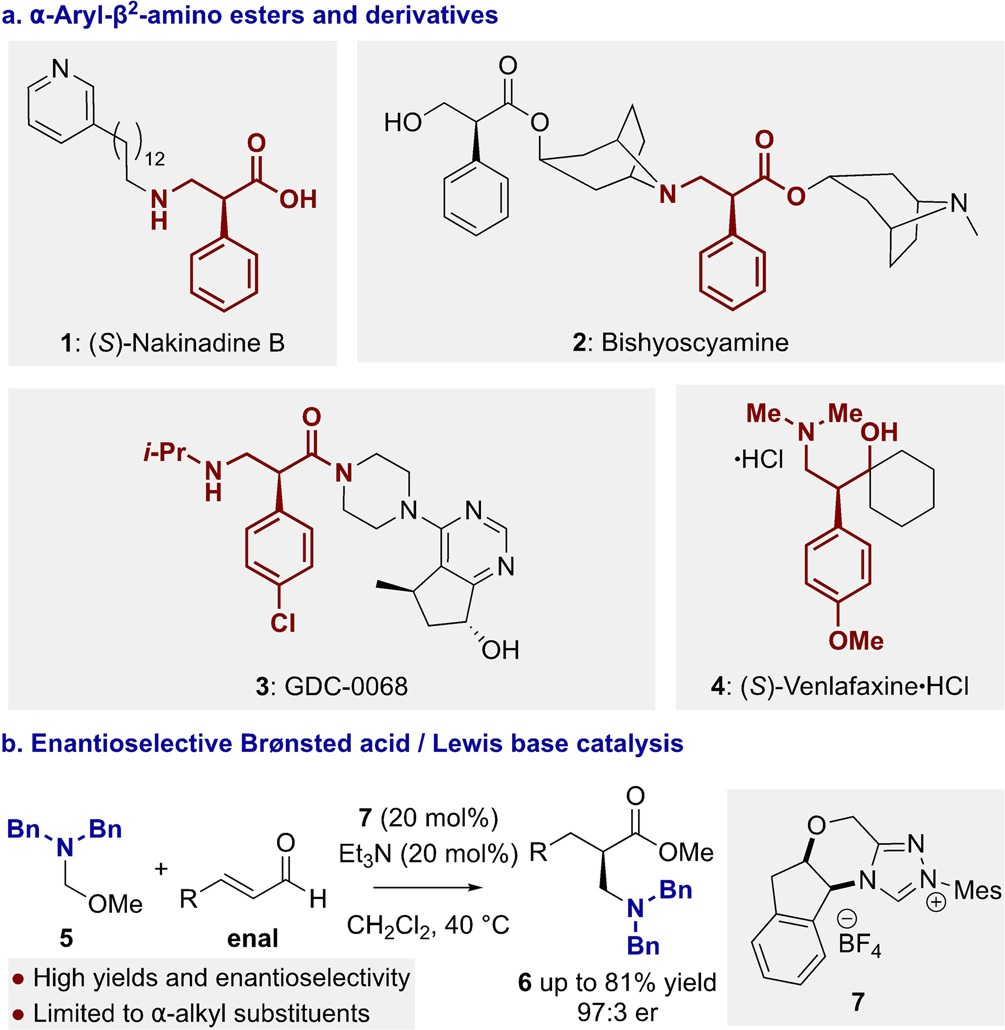
a) Naturally occurring and medicinally active α‐aryl‐β^2^‐amino acid derivatives. b) State of the art organocatalytic enantioselective synthesis of α‐alkyl‐β^2^‐amino esters.

β‐Amino acids are important functional groups in an abundance of natural products and medicinally relevant compounds. Although less common than their α‐homologues, many naturally occurring compounds contain the β‐amino acid motif as a key structural feature.^[1]^ The incorporation of β‐amino acids within bioactive peptides allows access to structures with modulated conformations and properties such as antimicrobial peptides,^[2]^ while β‐amino acid containing oligomers have important applications in the chemistry of unnatural peptides such as foldamers.^[3]^ Many examples of pharmaceutically active compounds contain a β‐amino carbonyl motif,^[4]^ with α‐aryl‐β^2^‐amino acids representing an important subclass. For example, natural products (*S*)‐Nakinadine B **1** isolated from the marine sponge *Amphimedon* sp.^[5]^ and Bishyocyamine **2** from *Anisodus acutangulus* ^[6]^ both contain an α‐aryl‐β^2^‐amino motif (Figure 1 a). Biologically active α‐aryl‐β^2^‐amino acid derivatives include amide GDC‐0068 **3** (ipatasertib), an effective pan‐Akt inhibitor,^[7]^ while amino‐alcohol derivative Venlafaxine⋅HCl **4** is a therapeutically approved treatment for depression.^[8]^

Enantioselective routes to β‐amino acids and their derivatives therefore represent an important synthetic challenge with a range of methods developed for their synthesis. Until recently, synthetic routes to β^3^‐amino acid derivatives typically relied upon the elegant and versatile lithium amide conjugate addition approach developed by Davies,^[9]^ or the use of chiral auxiliaries for the preparation of β^2^‐amino acids.[^10^, ^11^] Catalytic, enantioselective routes to β^2^‐amino acid derivatives have been established, and include the use of metal complexes with chiral ligands^[12]^ or complexes that exhibit metal‐centered chirality.^[13]^ Organocatalytic routes have also been reported, predominantly employing secondary amine Lewis base catalysts and aldehydes to access β‐amino aldehydes through enamine intermediates.^[14]^ Additionally, a thiourea‐catalyzed route to enantioenriched nitro‐compounds has been developed that can be effectively reduced to β‐amino acids,^[15]^ as have Brønsted acid catalyzed routes that harness the stereodirecting effect of a chiral anion.^[16]^

In 2015, Chi and co‐workers reported an enantioselective NHC‐catalyzed route to α‐alkyl‐β^2^‐amino esters **6** from enals using NHC‐precatalyst **7** and hemiaminal ether **5** as a precursor to an in situ generated iminium ion.^[17]^ This method incorporates Brønsted acid activation of amino‐ether **5** with the NHC‐catalyzed formation of azolium enolates, generating a range of α‐alkyl‐β^2^‐amino esters in good yields and excellent enantioselectivity (up to 97:3 er). Important to this method was the use of catalytic weak base (NEt_3_) that served a dual purpose; firstly to generate the free NHC, but secondly to allow the in situ acid‐catalyzed formation of a formyl iminium ion (as its conjugate acid). Despite its synthetic utility, one inherent limitation of this methodology is that the resulting products are restricted to α‐alkyl‐β^2^‐amino esters, and limited mechanistic understanding of this process has been developed. Given their importance, the development of a simple and effective enantioselective route to α‐aryl‐β^2^‐amino esters would be of significant synthetic value.

C(1)‐Ammonium enolates are powerful catalytically generated synthetic intermediates that have been widely used in a range of enantioselective C‐C and C‐X bond forming processes through reaction with suitable electrophilic partners.^[18]^ In recent years the reactivity of C(1)‐ammonium enolate intermediates has been significantly broadened through the development of aryl esters as precursors using isothioureas as catalysts.^[19]^ These substrates allow aryloxide promoted catalyst turnover and have been applied to enantioselective [2,3]‐sigmatropic rearrangements,^[20]^ cooperative catalysis with Pd‐, Ir‐ and Cu‐derived electrophiles,^[21]^ as well as base free ammonium enolate catalysis.^[22]^ Building upon this work, we proposed that enantioselective aminomethylation of C(1)‐ammonium enolates, catalytically generated from chiral isothioureas and arylacetic acid esters, would provide ready access to enantioenriched α‐aryl‐β^2^‐amino esters.

**Figure 2:**
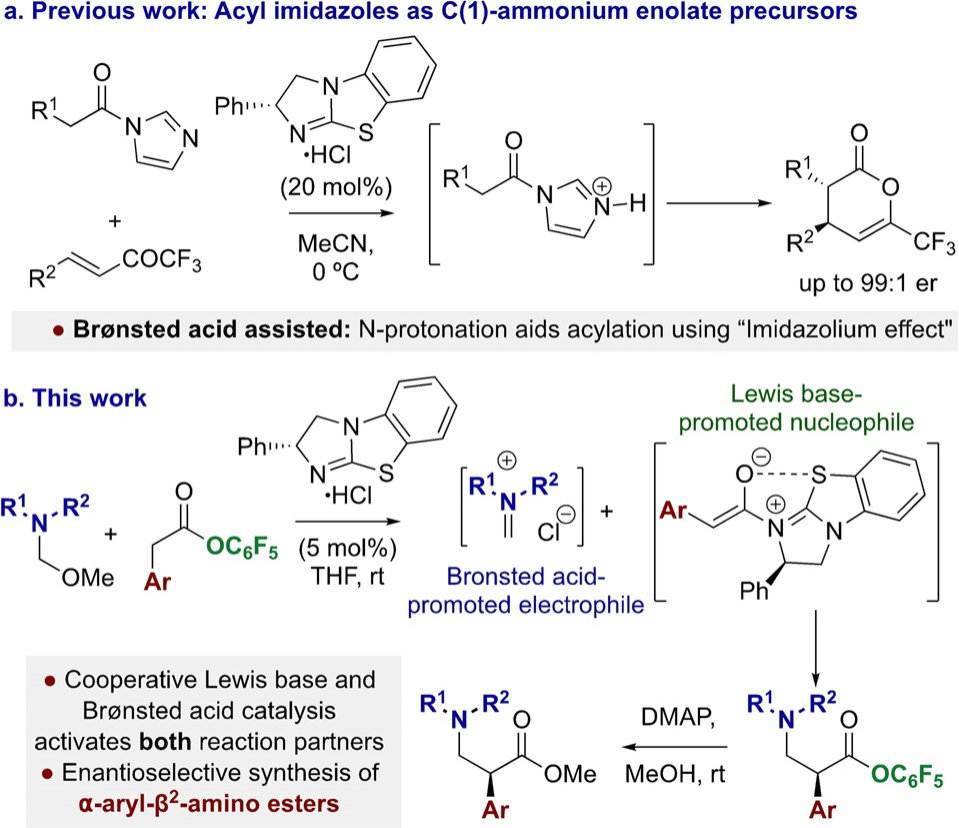
a) Precedent for using acyl imidazole and isothiourea⋅HCl salts in C(1)‐ammonium enolate generation. b) Proposed dual‐catalytic enantioselective synthesis of α‐aryl‐β^2^‐amino esters.

The process would require a cooperative Brønsted acid co‐catalyst to generate an iminium ion in situ from an amino ether precursor. This contrasts with the typical necessity to generate the C(1)‐ammonium enolate using a chiral tertiary amine in the presence of a stoichiometric Brønsted base. The addition of this auxiliary base is usually considered necessary to neutralize acidic by‐products, and to prevent protonation (and thus deactivation) of the Lewis base catalyst. The use of tertiary amine salts as Lewis base catalysts has limited precedent, with DMAP (4‐dimethylaminopyridine) salts having been used as acylation catalysts,^[23]^ and sub‐stoichiometric acid additives used sporadically with other tertiary amine Lewis base catalysis.[^24^, ^25^] In previous work we demonstrated the use of an isothiourea hydrochloride salt as a catalyst for C(1)‐ammonium enolate generation from N‐acyl imidazole precursors (Figure 2 a). N‐Protonation of the acyl imidazole (acting as a Brønsted base) both activated the substrate to nucleophilic attack and generated free isothiourea for Lewis base catalysis.^[26]^ Taking inspiration from this work, in this manuscript isothiourea hydrochloride salts are used to enable the catalytic enantioselective synthesis of α‐aryl‐β^2^‐amino esters.

The protocol harnesses Brønsted acid and isothiourea Lewis base catalysis for the catalytic activation of both reaction partners. The Brønsted acid promotes in situ generation of a reactive iminium species from a hemiaminal ether presumably through initial N‐protonation, generating the free isothiourea that is required for generation of a C(1)‐ammonium enolate (Figure 2 b). Comprehensive control studies aimed at understanding the mechanism of this process are consistent with a C(1)‐ammonium enolate pathway and highlight the subtle equilibria between species along the productive reaction pathway that lead to off‐cycle intermediates.

## Results and Discussion

### Investigation of Optimal Reaction Conditions

The effective enantioselective construction of α‐aryl‐β^2^‐amino ester **9** was realized via the benzotetramisole⋅HCl (BTM⋅HCl, 5 mol %) catalyzed Mannich addition of pentafluorophenyl ester pronucleophile **8** to hemiaminal ether iminium precursor **5** in THF at room temperature in the presence of 4 Å molecular sieves (MS). Subsequent addition of MeOH gave the isolable methyl ester **9** in excellent yield (81 %) and with high enantioselectivity (96:4 er) (Table 1, entry 1).^[27]^ Control experiments indicated that without the hydrochloric acid co‐catalyst, yield and enantioselectivity were significantly reduced even after extended reaction time (entry 2). Other halide salts gave high product yield but the er values were lower than for BTM⋅HCl (entries 3 and 4). Other classes of Brønsted acid co‐catalysts again led to high product yield but significantly diminished enantioselectivity compared with halide salts (entries 5–7).^[28]^ Removing molecular sieves led to reduced yield while maintaining product enantioselectivity (entry 8). This observation can be rationalized by the dual benefit of trapping adventitious water (to suppress hydrolysis of either ester **8**, or the in situ formed iminium ion generated from **5**) as well as trapping the in situ generated methanol (that can lead to the unproductive formation of the methyl ester of **8**). Further reduction in catalyst loading to 1 mol % gave reduced yield and slightly reduced enantioselectivity (entry 9). Alternative catalysts such as HyperBTM⋅HCl gave reduced enantioselectivity (entry 10), while use of a 4‐nitrophenyl ester led to comparable yield and enantioselectivity (entry 11). A simple solvent screen showed that CH_2_Cl_2_ led to reduced yield and enantioselectivity (entry 12), while toluene gave comparable enantioselectivity, but reduced yield, relative to THF (entry 13).

**Table 1:** Variation of Reaction Conditions.^[a]^ 
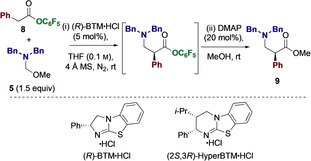

Entry	Variation	Yield^[b]^ [%]	er^[c]^
1	–	81	96:4
2	without HCl	40^[d]^	72:28
3	(*R*)‐BTM⋅HBr (5 mol %)	95	93:7
4	(*R*)‐BTM⋅HI (5 mol %)	94	89:11
5	(*R*)‐BTM⋅HBF_4_ (5 mol %)	89	71:29
6	(*R*)‐BTM⋅HO_2_CCF_3_ (5 mol %)	95	72:28
7	(*R*)‐BTM⋅HOTf (5 mol %)	88	66:34
8	without 4 Å MS^[e]^	44	94:6
9	(*R*)‐BTM⋅HCl (1 mol %)	55^[d]^	93:7
10	HyperBTM⋅HCl (5 mol %)	61	85:15
11	Ester=4‐nitrophenyl (OC_6_H_4_4‐NO_2_)	75	94:6
12	CH_2_Cl_2_ (0.1 m)	70	87:13
13	toluene (0.1 m)	60	95:5

[a] The mixture of **5** (0.3 mmol), **8** (0.2 mmol), (*R*)‐BTM⋅HCl (5 mol %), and 4 Å molecular sieves (MS, 100 mg) in dry THF (2 mL, 0.1 m) was stirred at rt under a N_2_ atmosphere for up to 24 hours before treatment with MeOH (0.5 mL) and DMAP (20 mol %) for 4 h. [b] Isolated yield. [c] Determined by HPLC analysis on a chiral stationary phase. [d] Incomplete reaction after 2–3 days before addition of MeOH. [e] Molecular sieves were activated in a furnace at 400 °C for 16 h. THF=Tetrahydrofuran. TfOH=Triflic acid. rt=room temperature.

### Scope, Limitations and Synthetic Applications

With the optimal conditions (Table 1, Entry 1) established, the generality of this protocol was investigated through variation of both the arylacetic ester and N‐substituents of the hemiaminal ether (Table 2). Initially, a range of substituted arylacetic pentafluorophenyl esters with different steric and electronic properties was tested through reaction with hemiaminal ether **5** under the developed conditions (Table 2 A). Importantly, arylacetic esters bearing electron‐donating (4‐dimethylamino‐, 4‐methoxy‐ and 4‐tolyl) substituents, as well as halogen substituents (4‐bromo‐, 4‐chloro‐ and 4‐fluoro‐) and electron‐withdrawing substituents (4‐trifluoromethyl‐) were all tolerated, with α‐aryl‐β^2^‐amino esters **10**–**16** all obtained with excellent yields (77 % to 94 %) and enantioselectivity (≈95:5 er). The absolute configuration of (*S*)‐**13** was determined by single crystal X‐ray crystallography, with all other products assigned by analogy.^[29]^ This stereochemical outcome is consistent with the expected configuration at C(2) if this process proceeds through a C(1)‐ammonium enolate intermediate.^[19c]^ Only the introduction of a 4‐nitro‐substituent led to the product **17** in reduced yield and enantioselectivity. Further variation of the arylacetic ester showed that incorporation of substituents at both the 3‐position (such as 3,4‐dimethoxy‐, 3‐methoxy‐ and 3‐bromo‐ to give **18**–**21**) and the 2‐position (2‐methoxy‐ and 2‐bromo‐ to give **22**–**23**) gave products with consistently high yields and er. Notably, the 2‐substitution pattern frequently provides reduced stereoselectivity in related isothiourea‐catalyzed methods.^[30]^ β^2^‐Amino esters **24** and **25** with 1‐ and 2‐naphthyl substituents were readily prepared, as was heteroaromatic 2‐thienyl **26**. Beyond aryl acetic acid derivatives, the use of (*E*)‐pent‐3‐enoic acid pentafluorophenyl ester was also productive, providing 3‐alkenyl‐substituted β^2^‐amino ester **27** in excellent er. The consistent yield and enantiocontrol observed with variation in both steric and electronic aryl substitution within the ester component indicate that neither provide a significant bias in this process. Further investigations probed the effect of N‐substituents within the hemiaminal ether reaction partner (Table 2 B). Halogen‐ (4‐fluoro and 4‐bromo) and electron donating (4‐methoxy) substituents were readily incorporated within the N‐benzyl groups, giving α‐aryl‐β^2^‐amino esters **28**–**30** in consistent yield and enantioselectivity. The hemiaminal ether derived from diallylamine also worked well in catalysis, giving β^2^‐amino ester **31** in 80 % yield and 94:6 er.

**Table 2:** Scope, Limitations and Synthetic Applications of the Enantioselective Aminomethylation.^[a,b]^

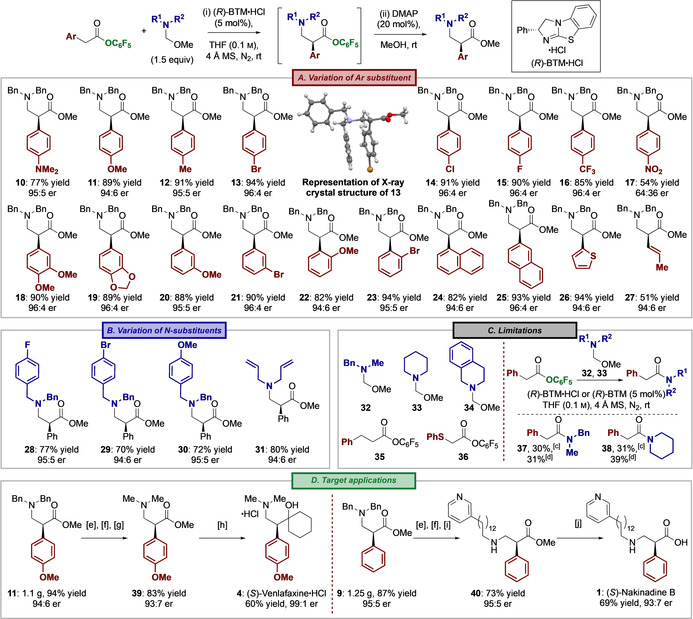

[a] Isolated yield; reaction progress followed by TLC analysis; see SI for reaction times; [b] er determined by HPLC analysis on a chiral stationary phase. [c] Using (*R*)‐BTM⋅HCl. [d] Using (*R*)‐BTM. [e] Et_2_O⋅HCl, CH_2_Cl_2_. [f] H_2_ (30 bar), Pd/C, MeOH, rt. [g] HCHO (37 wt % in H_2_O), NaBH(OAc)_3_, MgSO_4_, CH_2_Cl_2_, rt. [h] 1,5‐dibromopentane, Mg, ether; recrystallization after treatment with Et_2_O⋅HCl. [i] 13‐(pyridin‐3‐yl)tridecanal, NaBH(OAc)_3_, AcOH, 1,2‐dichloroethane, rt. [j] LiOH, THF:H_2_O (2:1), rt.

The limitations of this methodology were also probed (Table 2 C). Notably, the *N*‐benzyl‐*N*‐methyl and cyclic N‐alkyl substituted hemiaminal ether variants **32**–**34** led to complex product distributions that included <5 % product formation in THF. In the case of **32** and **33**, the corresponding amides **37** and **38** were isolated in 30–39 % yield upon treatment with either BTM⋅HCl or BTM (5 mol %). The formation of these amides is consistent with preferential N‐acylation of the corresponding hemiaminal ether with either the pentafluorophenyl ester or an in situ generated acyl ammonium ion, followed by fragmentation to generate the corresponding amide. This is consistent with observations from Böhme and Sickmüller,^[31]^ who demonstrated the ambident nature of hemiaminal ethers in reactions with acyl halides, with acyl transfer reactions leading to the formation of amides and esters. That this fragmentation pathway is preferred over iminium ion formation when using hemiaminal ethers **32**–**34** presumably reflects the increased nucleophilicity of these species relative to the corresponding *N*,*N*‐diallyl‐ or *N*,*N*‐dibenzyl substituted hemiaminal ethers that lead to constructive enantioselective aminomethylation. Alternative pathways involving either iminium hydrolysis, or formation of an oxonium ion and amine elimination, that subsequently lead to amide formation cannot be ruled out at this stage. Within the ester component, and consistent with common observations using isothiourea catalysis, 3‐phenylpropanoate and phenylthioacetate pentafluorophenyl esters **35** and **36** led to <5 % product,^[32]^ showing that this process complements the NHC‐catalyzed protocol developed by Chi and co‐workers.^[17]^

Having demonstrated the scope and limitations of this process, two target compounds, (*S*)‐Venlafaxine⋅HCl **4** ^[33]^ and (*S*)‐Nakinadine B **1**,^[34]^ were synthesized to demonstrate the utility of this methodology (Table 2 D). Each route began with the preparation of α‐aryl‐β^2^‐amino esters **11** and **9** on a >1 g scale, giving the products in excellent yield and er. For the synthesis of (*S*)‐Venlafaxine⋅HCl **4**, **11** was subjected to hydrogenolysis and reductive amination to give *N*,*N*‐dimethyl derivative **39** in 83 % yield. Double addition of the Grignard reagent derived from 1,5‐dibromopentane, followed by recrystallization, afforded the target compound in 99:1 er and 47 % overall yield over 5 steps. To prepare (*S*)‐Nakinadine B **1**, hydrogenolysis of **9**, followed by reductive amination with 13‐(pyridin‐3‐yl)tridecanal gave **40** in 73 % yield and 95:5 er, with ester hydrolysis giving **1** in 93:7 er and 44 % overall yield over 5 steps. In both cases correlation of the products with literature data served as proof of both structure and configuration.

### Mechanistic Investigations

Having explored the scope and limitations of this process, further investigations focused on developing a mechanistic understanding. Initial studies attempted to elucidate reaction orders and track any potential intermediates through temporal reaction monitoring using ^19^F{^1^H} NMR spectroscopy and ^19^F labelled reactants (see SI). However, the heterogeneous nature of the reaction, together with the observation of multiple resonances that could not be fully deconvoluted meant that these investigations were abandoned.^[28]^ Instead, a series of control experiments were performed to give insight to the productive reaction mechanism. At the onset of these investigations, two feasible mechanisms were considered that involved either enantioselective aminomethylation of a C(1)‐ammonium enolate, or an alternative dynamic kinetic resolution (DKR) process of a racemic (or scalemic) β‐amino pentafluorophenyl ester product. If a DKR were operative, a racemic β‐amino pentafluorophenyl ester added to an enantioselective reaction would be predicted to become enantioenriched over the reaction course. To test this hypothesis, racemic β‐amino ester **41** was prepared under standard reaction conditions using racemic BTM⋅HCl (10 mol %). β‐Amino ester **41** proved difficult to isolate due to the lability of the pentafluorophenyl ester and so was used directly without purification. Subsequent addition of 2‐naphthyl substituted pentafluorophenyl ester **42**, enantiopure (*R*)‐BTM⋅HCl (15 mol %) and **5** allowed potential DKR of the β‐amino ester **41** under the reaction conditions to be assessed alongside the enantioselective aminomethylation of **42** (Table 3 A). Upon isolation, 2‐naphthyl β‐amino ester **25** was obtained in 92:8 er and 81 % yield, with **9** isolated in 61 % yield and racemic form. This is inconsistent with a DKR of the β‐amino pentafluorophenyl ester product being operative under catalytically competent conditions, although DKR of a post‐aminomethylation acyl ammonium ion (prior to catalyst release by phenoxide) cannot be currently ruled out. Interestingly, while the overall BTM enantiomeric ratio (after second charge of catalyst) is expected to be 80:20, the er of product **25** was 92:8. As a linear correlation between the er of β‐amino ester product **9** and catalyst BTM⋅HCl was observed (Table 3 B), the er of **25** was higher than expected. We assume that either partial degradation or deactivation of racemic‐BTM⋅HCl from the first charge had occurred under the reaction conditions and may account for this discrepancy.

**Table 3:** Mechanistic Control Studies and Proposed Mechanism for Cooperative Lewis Base and Brønsted Acid Catalysis.

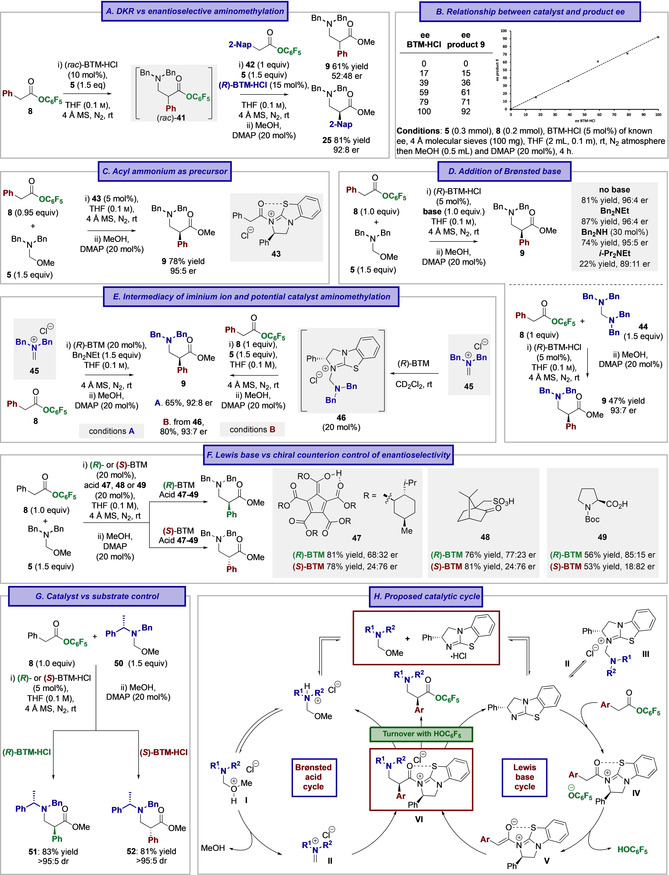

As DKR of the β‐amino pentafluorophenyl ester can be excluded, this reaction was proposed to proceed through enantioselective aminomethylation of a C(1)‐ammonium enolate intermediate generated from an acyl ammonium ion. To further probe this proposal, catalyst (*R*)‐BTM was acylated with phenylacetyl chloride to give isolable acyl ammonium ion **43**. Acyl ammonium **43** (5 mol %) was subsequently used as a precatalyst in the reaction process (Table 3 C), giving β‐amino ester **9** in comparable yield and er (78 % yield, 95:5 er) to using (*R*)‐BTM⋅HCl, indicating that **43** is a competent precatalyst for this transformation. Further studies considered the function of the hemiaminal ether within this process. If a pentafluorophenyl ester is used as an acyl ammonium ion precursor, the pentafluorophenolate formed upon acylation of the isothiourea is typically invoked as the functioning base for C(1)‐ammonium enolate formation.^[22]^ However, the reaction using acyl ammonium **43** as precatalyst proceeded without pentafluorophenolate, leading to the conclusion that the hemiaminal ether could function as a Brønsted base to promote deprotonation and C(1)‐ammonium enolate formation in this instance. The effect of addition of an amine base upon reaction conversion and product enantioselectivity was therefore investigated (Table 3 D). Performing the reaction with one equivalent of Bn_2_NEt (structurally similar to the hemiaminal ether **5**) gave β‐amino ester product **9** in 87 % yield and 96:4 er, while the use of secondary amine Bn_2_NH also gave **9** in high er. However, the use of *i*‐Pr_2_NEt (a commonly used Brønsted base in tertiary amine Lewis base catalysis) showed a significant reduction in product conversion and reduced product er (22 % yield, 89:11 er). As the addition of dibenzylamine did not lead to significant deviation in er, an alternative iminium ion precursor, tetra‐*N*‐benzylmethanediamine **44** was trialed in this process, giving β‐amino ester **9** in 47 % yield and 93:7 er. The nature of the base present in the reaction is therefore highly significant, presumably effecting both iminium ion and C(1)‐ammonium enolate formation though modulation of equilibrium distributions.

Further mechanistic investigations tested the feasibility of an iminium ion intermediate in the reaction process (Table 3 E). *N*,*N*‐Dibenzyliminium ion **45** was prepared from hemiaminal ether **5** using TMSCl (trimethylsilyl chloride)^[35]^ and used as a stoichiometric electrophile in a reaction with (*R*)‐BTM and ester **8** in the presence of Bn_2_NEt. β‐Amino ester **9** was generated in 65 % yield and 92:8 er, consistent with iminium ion **45** being a potential intermediate in this reaction process. Given the known ability of tertiary amines to add reversibly to iminium ions,^[36]^ further consideration led to the possibility of the iminium ion **45** being intercepted in situ by the Lewis base (*R*)‐BTM. Addition of (*R*)‐BTM to *N*,*N*‐dibenzyliminium chloride **45** showed complex behavior and gave a mixture of products by ^1^H NMR analysis, with aminomethylation of the Lewis base to give **46** tentatively assigned as a significant component.^[37]^ The use of this mixture as a potential precatalyst (20 mol %) gave β‐amino ester **9** in 80 % yield and 93:7 er upon treatment with pentafluorophenyl ester **8** and hemiaminal ether **5**. These control studies indicate that while **46** may be a potential reaction intermediate it is inconsequential to the outcome of the reaction process, presumably due to its reversible formation. The enantiodetermining step of the reaction was next investigated. Aminomethylation of the C(1)‐ammonium enolate was proposed to proceed enantioselectively, and the observed product configuration at C(2) is consistent with the known facial bias of the Lewis base catalyst. However, during optimization, variation of the counterion of the (*R*)‐BTM⋅HX salt led to significant variation in product er, and so further experiments considered the effect of a chiral counterion through use of chiral acid additives. Performing the reaction using either (*R*)‐ or (*S*)‐BTM alongside chiral acids **47**–**49** resulted in enantiocontrol being essentially completely dictated by the isothiourea, though with significant erosion of product er observed compared with the use of (*R*)‐BTM⋅HCl (Table 3 F). To further probe the factors leading to product enantioselectivity, the effect of using a chiral iminium ion was investigated. Using a single enantiomer of (*S*)‐hemiaminal ether **50**, and both enantiomers of BTM⋅HCl, gave β‐amino esters **51** and **52** as a single diastereoisomer in each case, consistent with the isothiourea catalyst determining enantioselectivity and overriding any control from the chiral iminium ion (Table 3 G).

Based on these experiments, the reaction is proposed to proceed via cooperative Lewis base and Brønsted acid catalytic cycles (Table 3 H). Reversible proton transfer from (*R*)‐BTM⋅HCl to the hemiaminal ether generates the free isothiourea catalyst (for the Lewis base cycle) and the N‐protonated hemiaminal ether for the Brønsted acid cycle. In the Brønsted acid cycle, proton transfer generates **I**, with subsequent formation of the iminium ion chloride salt **II**. In the Lewis base cycle, (*R*)‐BTM can undergo reversible addition to the iminium ion **II** to give aminomethylated catalyst **III** as an off‐cycle intermediate that is inconsequential to the outcome of the reaction.^[38]^ For the constructive formation of the β‐amino ester, *N*‐acylation of (*R*)‐BTM with the pentafluorophenyl ester generates an acyl ammonium pentafluorophenolate ion pair **IV**. Subsequent deprotonation generates the (*Z*)‐C(1)‐ammonium enolate **V**, with a key 1,5‐O⋅⋅⋅S contact,^[39]^ that can be classified as either a chalcogen‐chalcogen interaction[^40^, ^41^] or chalcogen bond (n_O_ to σ*_C‐S_),^[42]^ providing a conformational bias and ensuring coplanarity between the 1,5‐O‐ and S‐ atoms. Enantioselective aminomethylation proceeds preferentially through reaction of the *Re*‐face of the C(1)‐ammonium enolate with the iminium ion **II**, leading to intermediate **VI**. Subsequent reaction with the pentafluorophenol generated in situ leads to the desired β^2^‐amino ester and regenerates the isothiourea Lewis base and Brønsted acid catalysts.

## Conclusion

The development of an operationally simple, scalable and effective approach to the synthesis of α‐aryl‐β^2^‐amino esters via cooperative isothiourea tertiary amine Lewis base and Brønsted acid catalysis has been described. The utility of the method was demonstrated through an extensive substrate scope and the total synthesis of (*S*)‐Venlafaxine⋅HCl **4** and (*S*)‐Nakinadine B **1**. Mechanistic investigations support a C(1)‐ammonium enolate pathway rather than a DKR of an α‐aryl‐β^2^‐amino pentafluorophenyl ester. Control studies indicate that (i) a linear effect between catalyst and product er is observed; (ii) an acyl ammonium ion can be used as a precatalyst; (iii) reversible isothiourea addition to an in situ generated iminium ion leads to an off‐cycle intermediate that is inconsequential to the outcome of the reaction. Ongoing work within this laboratory is exploring alternative cooperative catalytic procedures.^[43]^

## Conflict of interest

The authors declare no conflict of interest.

## Supporting information

As a service to our authors and readers, this journal provides supporting information supplied by the authors. Such materials are peer reviewed and may be re‐organized for online delivery, but are not copy‐edited or typeset. Technical support issues arising from supporting information (other than missing files) should be addressed to the authors.

Supplementary
