# Supplementary material for: Enantioselective Synthesis of α‐Aryl‐β2‐Amino‐Esters by Cooperative Isothiourea and Brønsted Acid Catalysis
Source: Angew Chem Int Ed Engl. 2021 May 4;60(21):11892–900. doi: 10.1002/anie.202016220 (PMC8252622; doi:10.1002/anie.202016220)
Supplement: Supplementary file 1 — Supplementary [file ANIE-60-11892-s001.pdf]

Angewandte Chemie
Eine Zeitschrift der Gesellschaft Deutscher Chemiker

Supporting Information

Enantioselective Synthesis of α-Aryl-β^2-Amino-Esters by Cooperative Isothiourea and Brønsted Acid Catalysis

*Feng Zhao[+], Chang Shu[+], Claire M. Young, Cameron Carpenter-Warren, Alexandra M. Z. Slawin, and Andrew D. Smith**

anie_202016220_sm_miscellaneous_information.pdf

CONTENTS

1 General Experimental S1
2 Condition Optimization S3
3 General Procedures S5
3.1 General Procedure 1: Synthesis of pentafluorophenyl esters S5
3.2 General Procedure 2: Synthesis of hemiaminal ethers S6
3.3 General Procedure 3: Synthesis of isothiourea salts S6
3.4 General Procedure 4: Enantioselective synthesis of β^2-amino-esters S7
4 Data for substrates and scope S7
4.1 Data for pentafluorophenyl ester S7
4.2 Data for hemiaminal ethers S17
4.3 Data for isothiourea salts S22
4.4 Data for β^2-amino-esters S25
4.5 X-ray crystal structure S44
5 Target applications S45
5.1 Synthesis of (*S*)-Venlafaxine·HCl S45
5.2 Synthesis of (*S*)-Nakinadine B S47
6 Mechanistic Investigation S50
6.1 Reaction monitoring by ^{19}F NMR S50
6.2 DKR vs enantioselective aminomethylation S51
6.3 Relationship between catalyst and product ee S53
6.4 Acyl ammonium as a precursor S54
6.5 Addition of Brønsted bases S55
6.6 BTM alkylation S56
6.7 Chiral counterion control of enantioselectivity S59
6.8 Substrate control of stereoselectivity S60
7 References S62
Appendix I: NMR Spectra S63
Appendix II: HPLC Traces S178

1 General Experimental

Reactions involving moisture sensitive reagents were carried out in flame-dried glassware under a nitrogen atmosphere using standard vacuum line techniques. Anhydrous solvents (THF, CH_2Cl_2, Et_2O and toluene) were obtained from an anhydrous solvent system (purified using an alumina column, Mbraun SPS-800). Petrol is defined as petroleum ether 40–60 °C. All other solvents and commercial reagents were used as received without further purification unless otherwise stated.

Room temperature (rt) refers to 20–25 °C. Temperature of 0 °C was obtained using ice/water bath. Reactions involving heating were performed using DrySyn blocks and a contact thermocouple.

Under reduced pressure or '*In vacuo*' refers to the use of either a Büchi Rotavapor R-200 with a Büchi V-491 heating bath and Büchi V-800 vacuum controller, a Büchi Rotavapor R-210 with a Büchi V-491 heating bath and Büchi V-850 vacuum controller, a Heidolph Laborota 4001 with vacuum controller, an IKA RV10 rotary evaporator with a IKA HB10 heating bath and ILMVAC vacuum controller, or an IKA RV10 rotary evaporator with a IKA HB10 heating bath and Vacuubrand CVC3000 vacuum controller. Rotary evaporator condensers are fitted to Julabo FL601 Recirculating Coolers filled with ethylene glycol and set to –5 °C.

Analytical thin layer chromatography (TLC) was performed on pre-coated aluminium plates (Kieselgel 60 F254 silica). TLC visualisation was carried out with ultraviolet light (254 nm). Manual column chromatography was performed in glass columns fitted with porosity 3 sintered discs over Kieselgel 60 silica using the solvent system stated.

Melting points were recorded on an Electrothermal 9100 melting point apparatus.

Optical rotations were measured on a PerkinElmer Precisly/Model-341 polarimeter operating at the sodium D line with a 100 mm path cell at 20 °C.

HPLC analyses were obtained on either a Shimadzu HPLC consisting of a DGU-20A5 degassing unit, LC-20AT liquid chromatography pump, SIL-20AHT autosampler, CMB-20A communications bus module, SPD-M20A diode array detector and a CTO-20A column oven or a Shimadzu HPLC consisting of a DGU-20A5R degassing unit, LC-20AD liquid chromatography pump, SIL-20AHT autosampler, SPD-20A UV/Vis detector and a CTO-20A column oven. Separation was achieved using either DAICEL

CHIRALCEL OD-H and OJ-H columns or DAICEL CHIRALPAK AD-H, IC columns using the method stated. HPLC traces of enantiomerically enriched compounds were compared with authentic racemic spectra. Racemic compounds were synthesised under analogous reaction conditions using racemic catalysts.

^{1}H, ^{13}C, ^{19}F nuclear magnetic resonance (NMR) spectra were acquired on either a Bruker Avance 300 (^{1}H 300 MHz; ^{13}C 75 MHz; ^{19}F 282 MHz), Bruker Avance II 400 (^{1}H 400 MHz; ^{13}C 101 MHz; ^{19}F 376 MHz) or a Bruker Avance II 500 (^{1}H 500 MHz, ^{13}C 126 MHz, ^{19}F 470 MHz), spectrometer at ambient temperature in the deuterated solvent stated. All chemical shifts are quoted in parts per million (ppm) and referenced to the residual solvent peak. All coupling constants, J, are quoted in Hz. Multiplicities are indicated by: s (singlet), d (doublet), t (triplet), q (quartet), dd (doublet of doublets), dt (doublet of triplets), dq (doublet of quartets), td (triplet of doublets), ddd (doublet of doublet of doublets), ddt (doublet of doublet of triplets), m (multiplet) and dm (doublet of multiplet). The abbreviation Ar is used to denote aromatic, Ph to denote phenyl, Bn to denote benzyl, br to denote broad and app to denote apparent. NMR peak assignments were confirmed using 2D ^{1}H correlated spectroscopy (COSY), 2D ^{1}H–^{13}C heteronuclear multiple-bond correlation spectroscopy (HMBC), and 2D ^{1}H–^{13}C heteronuclear single quantum coherence (HSQC) where necessary.

Infrared spectra were recorded on a Shimadzu IRAffinity-1 Fourier transform IR spectrophotometer fitted with a Specac Quest ATR accessory (diamond puck). Spectra were recorded of either thin films or solids, with characteristic absorption wavenumbers (ν_{max}) reported in cm^{-1}.

Mass spectrometry (*m*/*z*) data were acquired by either electrospray ionisation (ESI) or electron ionization (EI), at the University of St Andrews Mass Spectrometry Facility.

2 Condition Optimization

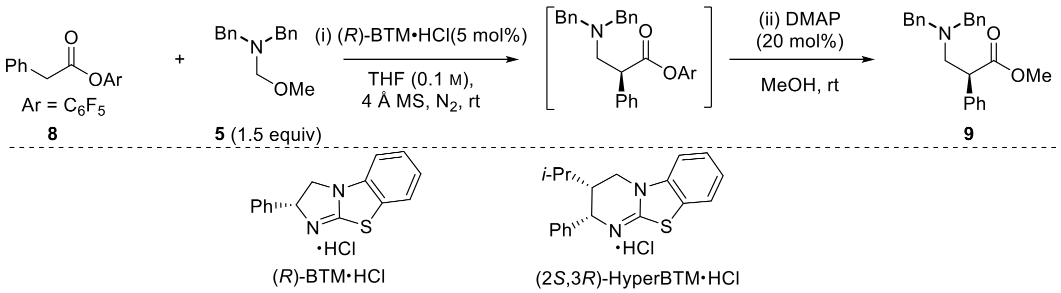

Entry	Variation	Time (h)	Yield[b] (%)	er[c]
1	--	24	81	96:4
2	without HCl	48	40[d]	72:28
3	(*R*)-BTM·HBr (5 mol%)	24	95	93:7
4	(*R*)-BTM·HI (5 mol%)	24	94	89:11
5	(*R*)-BTM·HBF_4 (5 mol%)	24	89	71:29
6	(*R*)-BTM·HCO_2CF_3 (5 mol%)	24	95	72:28
7	(*R*)-BTM (5 mol%) + HOAc (5 mol%)	24	<5%	--
8	(*R*)-BTM·HOTf (5 mol%)	24	88	66:34
9	(*R*)-BTM (5 mol%) and TsOH (5 mol%)	24	82	77:23
10	TsOH (20 mol%)	48	0[d]	--
11	(*R*)-BTM (10 mol%) and C_6F_5OH (10 mol%)	48	36[d]	68:32
12	(*R*)-BTM·HPF_6 (5 mol%)	24	92	66:34
13	(*R*)-BTM·H_3PO_4 (5 mol%)	24	46[d]	81:19
14	Bn_2NEt (0.5 equiv.) was added	24	93	96:4
15	Bn_2NEt (1.0 equiv.) was added	24	87	96:4
16	Bn_2NH (1 mol%) was added	24	80	97:3
17	Bn_2NH (5 mol%) was added	24	77	96:4
18	Bn_2NH (30 mol%) was added	24	74	95:5
19	*i*-Pr_2NEt (1.0 equiv.) was added	96	22[d]	89:11
20	**5** (2.0 equiv.) was used	24	87	96:4
21	**5** (3.0 equiv.) was used	24	73	95:5
22	(*R*)-BTM•HCl (1 mol%)	96	55[d]	93:7
23	(*R*)-BTM•HCl (1 mol%) + (*R*)-BTM (5 mol%)	48	65	95:5
24	(*R*)-BTM•HCl (5 mol%) + (*R*)-BTM (5 mol%)	24	83	95:5
25	Et_2O•HCl (1.0 equiv.) + (*R*)-BTM (5 mol%)	24	58	93:7
26	without 4 Å MS[e]	24	44	94:6
27	(2*S*,3*R*)-HyperBTM•HCl (5 mol%)	24	61	85:15
28	Ar = 4-NO_2-C_6H_4	48	75	94:6
29	CH_2Cl_2 (0.1 M)	48	70	87:13
30	toluene (0.1 M)	48	60	95:5
31	MeCN (0.1 M)	24	80	55:45

[a] **5** (0.3 mmol) was added to a stirred mixture of **8** (0.2 mmol), catalyst (5 mol%), and 4 Å molecular sieves (MS, 100 mg) in anhydrous THF (2 mL, 0.1 M) at rt under a N_2 atmosphere for up to 24 hours before treatment with anhydrous MeOH (0.5 mL) and DMAP (20 mol%) for 4 h. [b] Isolated yield. [c] Determined by HPLC analysis on a chiral stationary phase. [d] Incomplete reaction before addition of MeOH. [e] Molecular sieves were activated in a furnace at 400 ºC for 16 h.

See manuscript ref. [32]

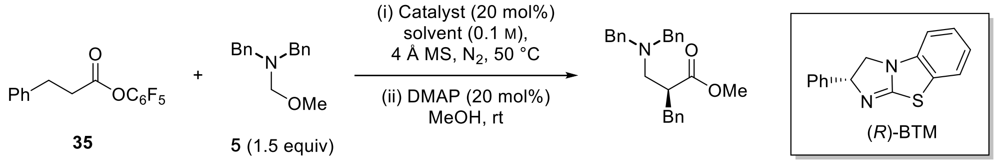

Entry	Catalyst	solvent	Yield[b] (%)
1	(*R*)-BTM·HCl	THF	--
2	(*R*)-BTM·HCl + NaOAc	THF	--
3	(*R*)-BTM·HCl + K_2CO_3	THF	--
4	(*R*)-BTM·HCl + Et_3N	THF	--
5	(*R*)-BTM·HCl + *i*-Pr_2NEt	THF	--
6	(*R*)-BTM·HCO_2CF_3	THF	--
7	(*R*)-BTM·HOTf	THF	--
8	(*R*)-BTM·HBF_4	THF	--
9[c]	(*R*)-BTM·HBF_4	CH_2Cl_2	--
10[c]	(*R*)-BTM·HBF_4 + Et_3N	CH_2Cl_2	--

[a] **5** (0.15 mmol) was added to a stirred mixture of **35** (0.1 mmol), catalyst (20 mol%), and 4 Å molecular sieves (MS, 100 mg) in anhydrous solvent (1 mL, 0.1 M) at 50 ºC under a N_2 atmosphere for up to 24 hours before treatment with anhydrous MeOH (0.5 mL) and DMAP (20 mol%) for 4 h. [b] All reactions gave very low conversion before treatment with MeOH, and no desired product was observed by crude NMR. [c] at 40 ºC.

3 General Procedures

3.1 General Procedure 1: Synthesis of pentafluorophenyl esters

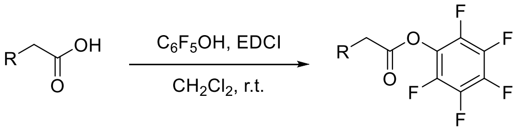

Pentafluorophenyl esters were prepared by a modified literature procedure.[1] The specified phenylacetic acid (1.0 equiv.), pentafluorophenol (1.1 equiv.) and 1-ethyl-3-(3-dimethylaminopropyl) carbodiimide hydrochloride (EDCI, 1.2 equiv.) in anhydrous CH_2Cl_2 (0.5 M) were stirred at room temperature for 16 hours. The reaction mixture was concentrated to dryness *in vacuo* and purified by flash column chromatography using a mixture of petroleum ether and ethyl acetate as eluent.

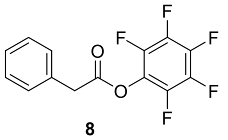

8

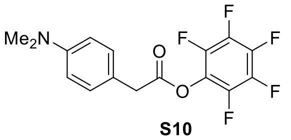

S10

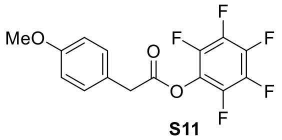

S11

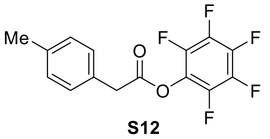

S12

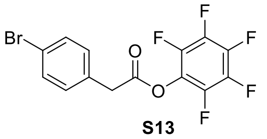

S13

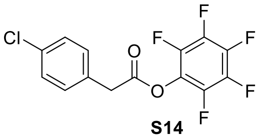

S14

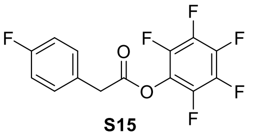

S15

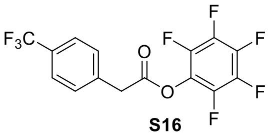

S16

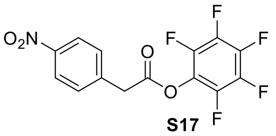

S17

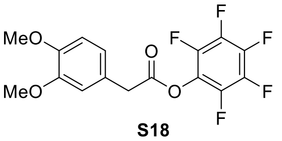

S18

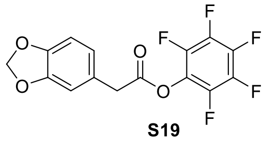

S19

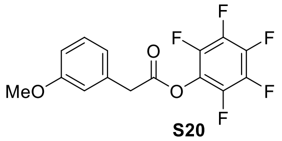

S20

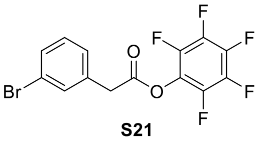

S21

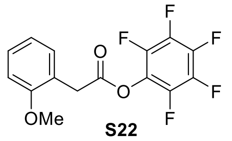

S22

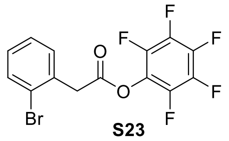

S23

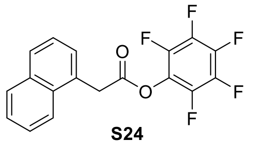

S24

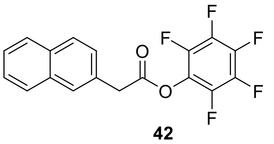

42

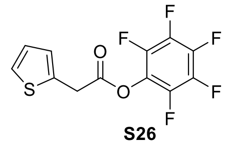

S26

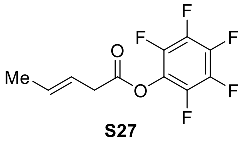

S27

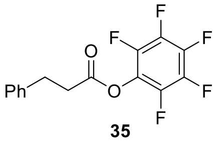

35

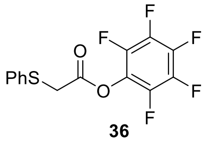

36

3.2 General Procedure 2: Synthesis of hemiaminal ethers

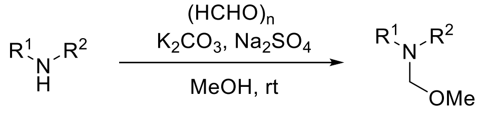

Following literature procedure,[2] a mixture of secondary amine (1.0 equiv.), paraformaldehyde (1.0 equiv.), K_2CO_3 (1.5 equiv.), and anhydrous Na_2SO_4 (1.5 equiv.) in anhydrous MeOH (1.0 M) was stirred at room temperature for 12 h, then filtered and concentrated to give the crude mixture as an oil, which was subsequently distilled under vacuum to obtain desired hemiaminal ether as a colorless liquid and used without further purification. Due to the high moisture sensitivity of these hemiaminal ethers, full characterization these species could not be obtained. NMR data and spectra (some with only ^{1}H NMR) of the partially decomposed materials are provided.

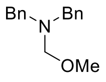
5

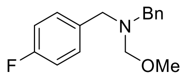
S28

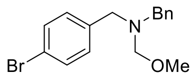
S29

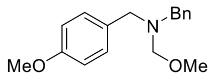
S30

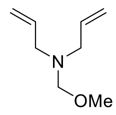
S31

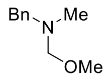
32

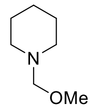
33

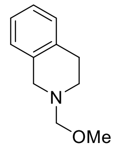
34

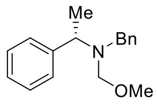
50

3.3 General Procedure 3: Synthesis of isothiourea salts

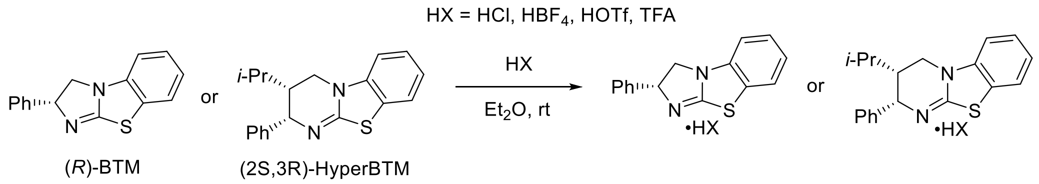

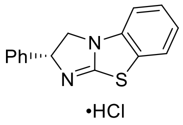
(R)-BTM•HCl

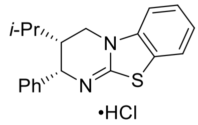
(2S,3R)-HyperBTM•HCl

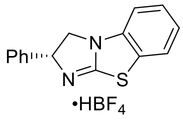
(R)-BTM•HBF$_4$

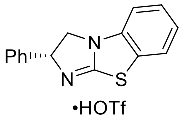
(R)-BTM•HOTf

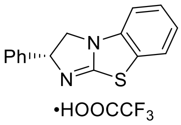
(R)-BTM•HOOCCF$_3$

Following the procedure of Smith and co-workers,[3] the isothiourea (1.0 equiv.) was dissolved in Et_2O (0.25 M). Acid (pure or ether solution, 1.0–2.0 equiv.) was added dropwise and the reaction was stirred at rt for 15 minutes. The reaction was then filtered and the precipitate was washed with Et_2O to give the desired isothiourea salt.

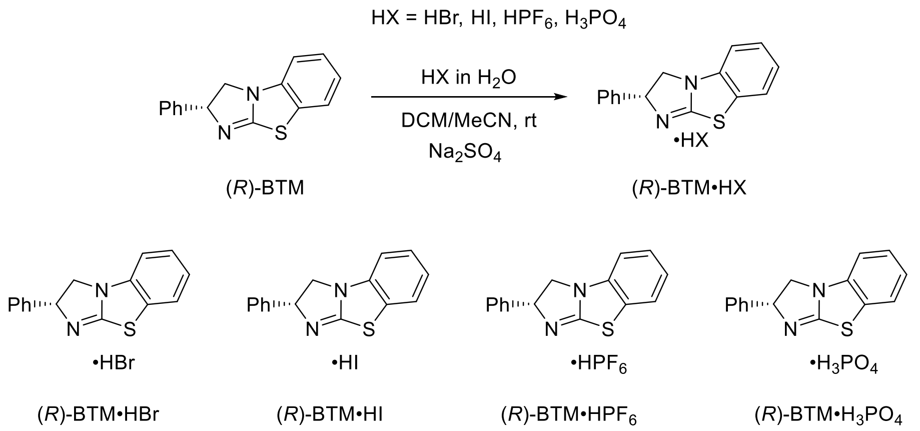

The mixture of (*R*)-BTM (1.0 equiv.) and anhydrous Na_2SO_4 (35.0 equiv.) in a mixed solvent of CH_2Cl_2 and MeCN (4:1, 0.02 M) was stirred vigorously at rt. Aqueous acid (1.0 – 4.0 equiv.) was added dropwise and the reaction was stirred at rt for 30 minutes. The reaction was then filtered and the filter cake was washed with DCM thoroughly. The filtrate was concentrated to dryness and the residual was redissolved in DCM and precipitated with Et_2O to give the desired isothiourea salt after a second filtration.

3.4 General Procedure 4: Enantioselective synthesis of β²-amino-esters

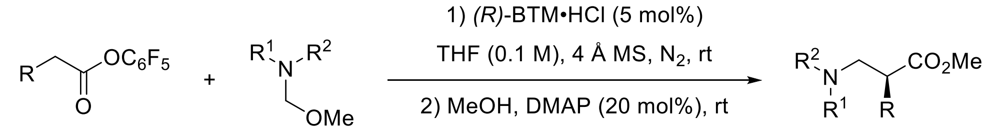

Hemiaminal ether (1.5 equiv.) was added to a mixture of ester (1.0 equiv.), (*R*)-BTM•HCl (5 mol%) and 4 Å MS (500 mg/mmol) in anhydrous THF (0.1 M). The resulting mixture was stirred at room temperature until full consumption of ester was observed by TLC. Anhydrous MeOH (1/4 volume of THF) and DMAP (20 mol%) were added and the mixture was stirred at room temperature for 4 hours. The reaction mixture was concentrated to dryness *in vacuo*, and the residue was purified by column chromatography using a mixture of petroleum ether and ethyl acetate as eluent. Authentic racemic samples were prepared in an analogous fashion using (±)-BTM•HCl.

4 Data for substrates and scope

4.1 Data for pentafluorophenyl ester

Perfluorophenyl 2-phenylacetate (8)[4]

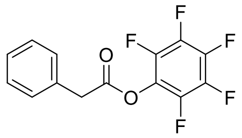

Following **General Procedure 1**, 2-phenylacetic acid (1.36 g, 10 mmol), pentafluorophenol (2.02 g, 11 mmol) and 1-ethyl-3-(3-dimethylaminopropyl) carbodiimide hydrochloride (2.30 g, 1.2 equiv.) in anhydrous CH_2Cl_2 (20 mL) were stirred at room temperature for 16 hours before concentrated and purified by flash column chromatography (95:5 Petrol : EtOAc) to give a colorless oil with spectroscopic data in accordance with the literature.[4] 2.40 g, 80% yield; **^{1}H NMR (400 MHz, $CDCl_3$)** δ_H: 4.00 (2H, s, C(2)H_2), 7.31 – 7.47 (5H, m, ArC*H*); **^{19}F NMR (376 MHz, $CDCl_3$)** δ_F: -162.29 (2F, m), -157.85 (1F, t, *J* 21.6, ArC(4)*F*), -152.69 – -152.52 (2F, m).

Perfluorophenyl 2-(4-(dimethylamino)phenyl)acetate (S10)[5]

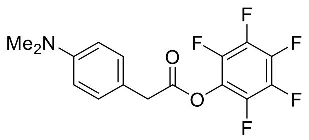

Following **General Procedure 1**, 2-(4-(dimethylamino)phenyl)acetic acid (1.79 g, 10 mmol), pentafluorophenol (2.02 g, 11 mmol) and 1-ethyl-3-(3-dimethylaminopropyl) carbodiimide hydrochloride (2.30 g, 1.2 equiv.) in anhydrous CH_2Cl_2 (20 mL) were stirred at room temperature for 16 hours before concentrated and purified by flash column chromatography (95:5 Petrol : EtOAc) to give a white solid with spectroscopic data in accordance with the literature.[5] 2.66 g, 77% yield; **mp** 57–58 ℃; **^{1}H NMR (400 MHz, $CDCl_3$)** δ_H: 2.98 (6H, s, N(CH_3)$_2$), 3.89 (2H, s, C(2)H_2), 6.71 – 6.79 (2H, m, ArC(3,5)*H*), 7.20 – 7.27 (2H, m, ArC(2,6)*H*); **^{19}F NMR (377 MHz, $CDCl_3$)** δ_F: -162.60 – -162.38 (2F, m), -158.20 (1F, t, *J* 21.7, ArC(4)*F*), -152.63 – -152.42 (2F, m).

Perfluorophenyl 2-(4-methoxyphenyl)acetate (S11)[1]

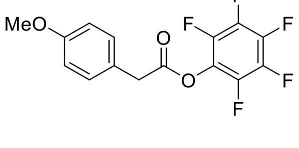

Following **General Procedure 1**, 2-(4-methoxyphenyl)acetic acid (1.66 g, 10 mmol), pentafluorophenol (2.02 g, 11 mmol) and 1-ethyl-3-(3-dimethylaminopropyl) carbodiimide hydrochloride (2.30 g, 1.2 equiv.) in anhydrous CH_2Cl_2 (20 mL) were

stirred at room temperature for 16 hours before concentrated and purified by flash column chromatography (95:5 Petrol： EtOAc) to give a white solid with spectroscopic data in accordance with the literature.[1] 2.49 g, 75% yield; **mp** 56–57 ℃; **^{1}H NMR (400 MHz, CDCl$_3$)** δ_H: 3.84 (3H, s, OC*H*$_3$), 3.93 (2H, s, C(2)*H*$_2$), 6.89 – 6.98 (2H, m, ArC(3,5)*H*), 7.23 – 7.38 (2H, m, ArC(2,6)*H*); **^{19}F NMR (376 MHz, CDCl$_3$)** δ_F: -162.47 – -162.22 (2F, m), -157.94 (1F, t, *J* 21.7, ArC(4)*F*), -152.76 – -152.52 (2F, m).

Perfluorophenyl 2-(p-tolyl)acetate (S12)[1]

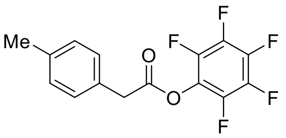

Following **General Procedure 1**, 2-(p-tolyl)acetic acid (1.50 g, 10 mmol), pentafluorophenol (2.02 g, 11 mmol) and 1-ethyl-3-(3-dimethylaminopropyl) carbodiimide hydrochloride (2.30 g, 1.2 equiv.) in anhydrous CH_2Cl_2 (20 mL) were stirred at room temperature for 16 hours before concentrated and purified by flash column chromatography (95:5 Petrol： EtOAc) to give a white solid with spectroscopic data in accordance with the literature.[1] 2.49 g, 79% yield; **mp** 30–31 ℃; **^{1}H NMR (400 MHz, CDCl$_3$)** δ_H: 2.40 (3H, s, Ar-C*H*$_3$), 3.96 (2H, s, C(2)*H*$_2$), 7.19 – 7.25 (2H, m, ArC*H*), 7.26 – 7.30 (2H, m, ArC*H*); **^{19}F NMR (376 MHz, CDCl$_3$)** δ_F: -162.60 – -162.15 (2F, m), -157.96 (1F, t, *J* 21.7, ArC(4)*F*), -152.90 – -152.29 (2F, m).

Perfluorophenyl 2-(4-bromophenyl)acetate (S13)[5]

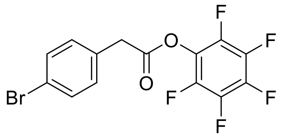

Following **General Procedure 1**, 2-(4-bromophenyl)acetic acid (2.15 g, 10 mmol), pentafluorophenol (2.02 g, 11 mmol) and 1-ethyl-3-(3-dimethylaminopropyl) carbodiimide hydrochloride (2.30 g, 1.2 equiv.) in anhydrous CH_2Cl_2 (20 mL) were stirred at room temperature for 16 hours before concentrated and purified by flash column chromatography (95:5 Petrol : EtOAc) to give a colorless oil with spectroscopic data in accordance with the literature.[5] 3.08 g, 81% yield; **^{1}H NMR (400 MHz, CDCl$_3$)** δ_H: 3.96 (2H, s, C(2)*H*$_2$), 7.23 – 7.31 (2H, m, ArC(3,5)*H*), 7.48 – 7.58 (2H,

m, ArC(2,6)*H*); **^{19}F NMR (376 MHz, $CDCl_3$)** δ_F: -162.62 – -161.84 (2F, m), -157.54 (1F, t, *J* 21.7, ArC(4)*F*), -152.78 – -152.52 (2F, m).

Perfluorophenyl 2-(4-chlorophenyl)acetate (S14)[1]

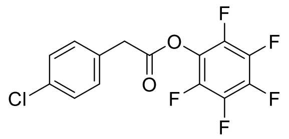

Following **General Procedure 1**, 2-(4-chlorophenyl)acetic acid (1.70 g, 10 mmol), pentafluorophenol (2.02 g, 11 mmol) and 1-ethyl-3-(3-dimethylaminopropyl) carbodiimide hydrochloride (2.30 g, 1.2 equiv.) in anhydrous CH_2Cl_2 (20 mL) were stirred at room temperature for 16 hours before concentrated and purified by flash column chromatography (95:5 Petrol : EtOAc) to give a colorless oil with spectroscopic data in accordance with the literature.[1] 2.45 g, 73% yield; **^{1}H NMR (400 MHz, $CDCl_3$)** δ_H: 3.97 (2H, s, C(2)H_2), 7.29 – 7.34 (2H, m, ArC(3,5)*H*), 7.35 – 7.41 (2H, m,Ar(2,6)*H*); **^{19}F NMR (376 MHz, $CDCl_3$)** δ_F: -162.90 – -161.30 (2F, m), -157.56 (1F, t, *J* 21.4, ArC(4)*F*), -153.62 – -151.87 (2F, m).

Perfluorophenyl 2-(4-fluorophenyl)acetate (S15)[6]

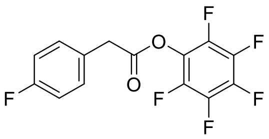

Following **General Procedure 1**, 2-(4-fluorophenyl)acetic acid (1.54 g, 10 mmol), pentafluorophenol (2.02 g, 11 mmol) and 1-ethyl-3-(3-dimethylaminopropyl) carbodiimide hydrochloride (2.30 g, 1.2 equiv.) in anhydrous CH_2Cl_2 (20 mL) were stirred at room temperature for 16 hours before concentrated and purified by flash column chromatography (95:5 Petrol : EtOAc) to give a colorless oil with spectroscopic data in accordance with the literature.[6] 2.27 g, 71% yield; **^{1}H NMR (400 MHz, $CDCl_3$)** δ_H: 3.97 (2H, s, C(2)H_2), 7.02 – 7.16 (2H, m), 7.31 – 7.42 (2H, m); **^{19}F NMR (377 MHz, $CDCl_3$)** δ_F: -162.84 – -161.55 (2F, m), -157.67 (1F, t, *J* 21.7, Ar_FC(4)*F*), -153.24 – -152.42 (2F, m), -114.44 (1F, s, CH_2ArC(4)*F*).

Perfluorophenyl 2-(4-(trifluoromethyl)phenyl)acetate (S16)[5]

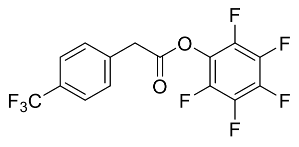

Following **General Procedure 1**, 2-(4-(trifluoromethyl)phenyl)acetic acid (2.04 g, 10 mmol), pentafluorophenol (2.02 g, 11 mmol) and 1-ethyl-3-(3-dimethylaminopropyl) carbodiimide hydrochloride (2.30 g, 1.2 equiv.) in anhydrous CH_2Cl_2 (20 mL) were stirred at room temperature for 16 hours before concentrated and purified by flash column chromatography (95:5 Petrol： EtOAc) to give a white solid with spectroscopic data in accordance with the literature.[5] 2.81 g, 76% yield; **mp** 38–39 ℃; **^{1}H NMR (500 MHz, $CDCl_3$)** δ_H: 4.07 (2H, s, C(2)*H*$_2$), 7.52 (2H, d, *J* 8.3, ArC(3,5)*H*), 7.68 (2H, d, *J* 8.3, ArC(2,6)*H*); **^{19}F NMR (470 MHz, $CDCl_3$)** δ_F: -162.24 – -161.76 (2F, m), -157.37 (1F, t, *J* 21.9, ArC(4)*F*), -153.06 – -152.38 (2F, m), -62.71 (3F, s, C*F*$_3$).

Perfluorophenyl 2-(4-nitrophenyl)acetate (S17)

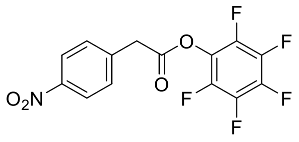

Following **General Procedure 1**, 2-(4-nitrophenyl)acetic acid (1.81 g, 10 mmol), pentafluorophenol (2.02 g, 11 mmol) and 1-ethyl-3-(3-dimethylaminopropyl) carbodiimide hydrochloride (2.30 g, 1.2 equiv.) in anhydrous CH_2Cl_2 (20 mL) were stirred at room temperature for 16 hours before concentrated and purified by flash column chromatography (95:5 Petrol： EtOAc) to give a white solid with spectroscopic data in accordance with the literature. 2.25 g, 65% yield; **mp** 87–88 ℃; **^{1}H NMR (400 MHz, $CDCl_3$)** δ_H: 4.12 (2H, s, C(2)*H*$_2$), 7.51 – 7.68 (2H, m), 8.12 – 8.46 (2H, m); **^{19}F NMR (377 MHz, $CDCl_3$)** δ_F: -163.39 – -160.76 (2F, m), -157.03 (1F, t, *J* 21.7, Ar_FC(4)*F*), -154.74 – -151.17 (2F, m); **^{13}C NMR (101 MHz, $CDCl_3$)** δ_C: 39.7 (*C*(2)H$_2$), 124.1(Ar*C*(3,5)H), 130.4 (Ar*C*(2,6)H), 137.9 (dm, *J* 250), 139.1 (Ar*C*(1)), 139.7 (dm, *J* 250), 141.0 (dm, *J* 250), 147.7 (Ar*C*(4)-NO_2), 166.3 (*C*(1)); **IR** ν_{max} (film) 1774 (C=O), 1517 (NO_2); **HRMS** (EI$^+$) $C_{14}H_6F_5NO_4$ [M]$^+$ found 347.0213, requires 347.0217 (–1.2 ppm).

Perfluorophenyl 2-(3,4-dimethoxyphenyl)acetate (S18)[6]

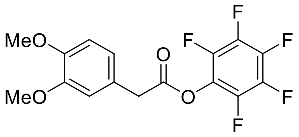

Following **General Procedure 1**, 2-(3,4-dimethoxyphenyl)acetic acid (1.96 g, 10 mmol), pentafluorophenol (2.02 g, 11 mmol) and 1-ethyl-3-(3-dimethylaminopropyl) carbodiimide hydrochloride (2.30 g, 1.2 equiv.) in anhydrous CH_2Cl_2 (20 mL) were stirred at room temperature for 16 hours before concentrated and purified by flash column chromatography (95:5 Petrol：EtOAc) to give a white solid with spectroscopic data in accordance with the literature.[6] 2.89 g, 80% yield; **mp** 66–67 ℃; **^{1}H NMR (500 MHz, $CDCl_3$)** δ_H: 3.86 – 3.96 (8H, m), 6.85 – 6.94 (3H, m); **^{19}F NMR (470 MHz, $CDCl_3$)** δ_F: -162.90 – -161.35 (2F, m), -157.91 (1F, t, *J* 21.9, ArC(4)*F*), -153.44 – -152.30 (2F, m).

Perfluorophenyl 2-(benzo[d][1,3]dioxol-5-yl)acetate (S19)[7]

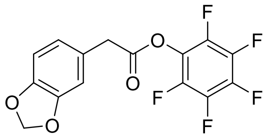

Following **General Procedure 1**, 2-(benzo[d][1,3]dioxol-5-yl)acetic acid (1.80 g, 10 mmol), pentafluorophenol (2.02 g, 11 mmol) and 1-ethyl-3-(3-dimethylaminopropyl) carbodiimide hydrochloride (2.30 g, 1.2 equiv.) in anhydrous CH_2Cl_2 (20 mL) were stirred at room temperature for 16 hours before concentrated and purified by flash column chromatography (95:5 Petrol：EtOAc) to give a white solid with spectroscopic data in accordance with the literature.[7] 2.87 g, 83% yield; **mp** 61–62 ℃; **^{1}H NMR (500 MHz, $CDCl_3$)** δ_H: 3.90 (2H, s, C(2)*H*$_2$), 6.00 (2H, s, OC*H*$_2$O), 6.78 – 6.86 (2H, m, ArC(5,6)*H*), 6.87 (1H, s, ArC(2)*H*); **^{19}F NMR (470 MHz, $CDCl_3$)** δ_F: -162.75 – -161.51 (2F, m), -157.92 (1F, t, *J* 21.6, ArC(4)*F*), -153.48 – -152.04 (2F, m); **^{13}C NMR (126 MHz, $CDCl_3$)** δ_C: 39.8 (*C*(2)H$_2$), 101.3 (OCH$_2$O), 108.5 (C(2)H$_2$Ar*C*(5)H), 109.6 (C(2)H$_2$Ar*C*(2)H), 122.6 (C(2)H$_2$Ar*C*(6)H), 125.1 (m, Ar$_F$*C*(1)), 125.5 (C(2)H$_2$Ar*C*(1)), 137.9 (dm, *J* 250), 139.5 (dm, *J* 250), 141.1 (dm, *J* 250), 147.3 (CH$_2$Ar*C*(4)), 148.1 (CH$_2$Ar*C*(3)), 167.6 (*C*(1)); **IR** ν_{max} (film) 1786 (C=O); **HRMS** (EI$^+$) $C_{15}H_7F_5O_4$ [M]$^+$ found 346.0260, requires 346.0265 (–1.3 ppm).

Perfluorophenyl 2-(3-methoxyphenyl)acetate (S20)[8]

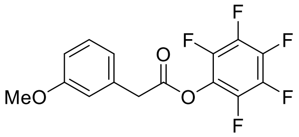

Following **General Procedure 1**, 2-(3-methoxyphenyl)acetic acid (1.66 g, 10 mmol), pentafluorophenol (2.02 g, 11 mmol) and 1-ethyl-3-(3-dimethylaminopropyl) carbodiimide hydrochloride (2.30 g, 1.2 equiv.) in anhydrous CH_2Cl_2 (20 mL) were stirred at room temperature for 16 hours before concentrated and purified by flash column chromatography (95:5 Petrol : EtOAc) to give a colorless oil with spectroscopic data in accordance with the literature.[8] 2.55 g, 77% yield; **^{1}H NMR (400 MHz, $CDCl_3$)** δ_H: 3.85 (3H, s, OC*H*$_3$), 3.97 (2H, s, C(2)*H*$_2$), 6.86 – 7.10 (3H, m), 7.32 (1H, t, *J* 7.9); **^{19}F NMR (376 MHz, $CDCl_3$)** δ_F: -163.76 – -161.63 (2F, m), -157.82 (1F, t, *J* 21.7, Ar$_F$C(4)*F*), -153.49 – -151.77 (2F, m); **^{13}C NMR (101 MHz, $CDCl_3$)** δ_C: 40.2 (*C*(2)H$_2$), 55.3 (OCH$_3$), 113.4 (ArC(4)H), 114.8 (ArC(2)H), 121.5 (ArC(6)H), 125.1 (m, Ar$_F$*C*(1)), 129.9 (Ar*C*(5)H), 133.4 (Ar*C*(1)), 137.87 (dm, *J* 250), 139.54 (dm, *J* 250), 141.17 (dm, *J* 250), 159.9 (Ar*C*(3)-OCH$_3$), 167.4 (*C*(1)); **IR** ν_{max} (film) 1788 (C=O); **HRMS** (EI$^+$) $C_{15}H_9F_5O_3$ [M]$^+$ found 332.0467, requires 332.0472 (–1.5 ppm).

Perfluorophenyl 2-(3-bromophenyl)acetate (S21)[9]

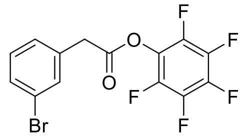

Following **General Procedure 1**, 2-(3-bromophenyl)acetic acid (2.15 g, 10 mmol), pentafluorophenol (2.02 g, 11 mmol) and 1-ethyl-3-(3-dimethylaminopropyl) carbodiimide hydrochloride (2.30 g, 1.2 equiv.) in anhydrous CH_2Cl_2 (20 mL) were stirred at room temperature for 16 hours before concentrated and purified by flash column chromatography (95:5 Petrol : EtOAc) to give a white solid with spectroscopic data in accordance with the literature.[9] 3.16 g, 83% yield; **mp** 36–37 ℃; **^{1}H NMR (400 MHz, $CDCl_3$)** δ_H: 3.97 (2H, s, C(2)*H*$_2$), 7.27 – 7.37 (2H, m), 7.43 – 7.62 (2H, m); **^{19}F NMR (376 MHz, $CDCl_3$)** δ_F: -163.33 – -161.08 (2F, m), -157.51 (1F, t, *J* 21.7, Ar$_F$C(4)*F*), -153.70 – -150.41 (2F, m); **^{13}C NMR (101 MHz, $CDCl_3$)** δ_C: 39.6 (*C*(2)H$_2$), 122.8 (Ar*C*(3)-Br), 127.9, 130.4, 131.1, 132.4, 134.1 (Ar*C*(1)), 137.89 (dm, *J* 250), 139.6 (dm, *J* 250), 141.1 (dm, *J* 250),

166.9 (C(1)); **IR** ν_{max} (film) 1788 (C=O); **HRMS** (EI^+) $C_{14}H_6{}^{79}BrF_5O_2$ $[M]^+$ found 379.9465, requires 379.9471 (–1.7 ppm).

Perfluorophenyl 2-(2-methoxyphenyl)acetate (S22)[1]

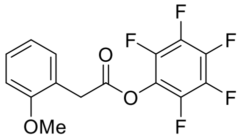

Following **General Procedure 1**, 2-(2-methoxyphenyl)acetic acid (1.66 g, 10 mmol), pentafluorophenol (2.02 g, 11 mmol) and 1-ethyl-3-(3-dimethylaminopropyl) carbodiimide hydrochloride (2.30 g, 1.2 equiv.) in anhydrous CH_2Cl_2 (20 mL) were stirred at room temperature for 16 hours before concentrated and purified by flash column chromatography (95:5 Petrol： EtOAc) to give a white solid with spectroscopic data in accordance with the literature.[1] 2.32 g, 70% yield; **mp** 38–39 ℃; **^{1}H NMR (400 MHz, $CDCl_3$)** δ_H: 3.89 (3H, s, OCH_3), 3.96 (2H, s, C(2)H_2), 6.94 (1H, dd, *J* 8.3, 1.2, ArC(3)*H*), 6.99 (1H, td, *J* 7.4, 1.1, ArC(4)*H*), 7.26 – 7.30 (1H, m, ArC(5)*H*), 7.35 (1H, ddd, *J* 8.2, 7.5, 1.8, ArC(6)*H*); **^{19}F NMR (376 MHz, $CDCl_3$)** δ_F: -163.24 – -162.06 (2F, m), -158.30 (1F, t, *J* 21.4, ArC(4)*F*), -152.88 – -152.67 (2F, m).

Perfluorophenyl 2-(2-bromophenyl)acetate (S23)[8]

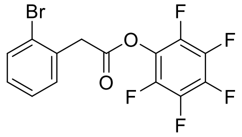

Following **General Procedure 1**, 2-(2-bromophenyl)acetic acid (2.15 g, 10 mmol), pentafluorophenol (2.02 g, 11 mmol) and 1-ethyl-3-(3-dimethylaminopropyl) carbodiimide hydrochloride (2.30 g, 1.2 equiv.) in anhydrous CH_2Cl_2 (20 mL) were stirred at room temperature for 16 hours before concentrated and purified by flash column chromatography (95:5 Petrol： EtOAc) to give a white solid with spectroscopic data in accordance with the literature.[8] 2.93 g, 77% yield; **mp** 69–70 ℃; **^{1}H NMR (400 MHz, $CDCl_3$)** δ_H: 4.17 (2H, s, C(2)H_2), 7.24 (1H, td, *J* 7.8, 1.9, ArC(4)*H*), 7.36 (1H, td, *J* 7.4, 1.2, ArC(5)*H*), 7.41 (1H, dd, *J* 7.6, 1.8, ArC(6)*H*), 7.65 (1H, dd, *J* 8.0, 1.1, ArC(3)*H*); **^{19}F NMR (376 MHz, $CDCl_3$)** δ_F: -164.18 – -161.32 (2F, m), -157.73 (1F, t, *J* 21.7, Ar_FC(4)*F*), -153.22 – -151.05 (2F, m); **^{13}C NMR (101 MHz, $CDCl_3$)** δ_C: 40.7 (C(2)H_2), 125.1 (ArC(2)-Br), 127.9 (ArC(5)H), 129.7 (ArC(6)H), 131.5 (ArC(4)H), 132.4 (ArC(1)), 133.1 (ArC(3)H),

137.9 (dm, *J* 250), 139.6 (dm, *J* 250), 141.2 (dm, *J* 250), 166.5 (*C*(1)); **IR** ν_{max} (film) 1786 (C=O); **HRMS** (EI^+) $C_{14}H_6{}^{79}BrF_5O_2$ $[M]^+$ found 379.9476, requires 379.9471 (+1.2 ppm).

Perfluorophenyl 2-(naphthalen-1-yl)acetate (S24)[8]

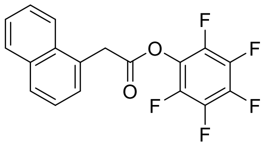

Following **General Procedure 1**, 2-(naphthalen-1-yl)acetic acid (1.86 g, 10 mmol), pentafluorophenol (2.02 g, 11 mmol) and 1-ethyl-3-(3-dimethylaminopropyl) carbodiimide hydrochloride (2.30 g, 1.2 equiv.) in anhydrous CH_2Cl_2 (20 mL) were stirred at room temperature for 16 hours before concentrated and purified by flash column chromatography (95:5 Petrol： EtOAc) to give a white solid with spectroscopic data in accordance with the literature.[8] 2.49 g, 71% yield; **mp** 94–95 ℃; **^{1}H NMR (500 MHz, $CDCl_3$)** δ_H: 4.44 (2H, s, C(2)H_2), 7.45 – 7.60 (3H, m), 7.61 – 7.66 (1H, m), 7.90 (1H, d, *J* 7.9), 7.94 (1H, d, *J* 7.9), 8.03 (1H, d, *J* 8.4); **^{19}F NMR (470 MHz, $CDCl_3$)** δ_F: -163.31 – -161.39 (2F, m), -157.81 (1F, t, *J* 21.7, Ar_FC(4)*F*), -153.25 – -152.06 (2F, m); **^{13}C NMR (126 MHz, $CDCl_3$)** δ_C: 38.0 (*C*(2)H_2), 123.2, 125.0 (m, Ar_F*C*(1)), 125.5, 126.1, 126.8, 128.3, 128.5, 128.9, 129.0, 131.8, 133.9, 137.8 (dm, *J* 250), 139.5 (dm, *J* 250), 141.1 (dm, *J* 250), 167.6 (*C*(1)); **HRMS (ESI$^+$)** $C_{18}H_9F_5NaO_2$ $[M+Na]^+$ requires 375.0420, found 375.0408 (–3.2 ppm); **IR** ν_{max} (film) 1784 (C=O); **HRMS** (ESI$^+$) $C_{18}H_9F_5O_2$ $[M+Na]^+$ found 375.0408, requires 375.0415 (–1.8 ppm).

Perfluorophenyl 2-(naphthalen-2-yl)acetate (42)[1]

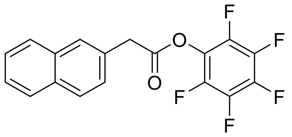

Following **General Procedure 1**, 2-(naphthalen-2-yl)acetic acid (1.86 g, 10 mmol), pentafluorophenol (2.02 g, 11 mmol) and 1-ethyl-3-(3-dimethylaminopropyl) carbodiimide hydrochloride (2.30 g, 1.2 equiv.) in anhydrous CH_2Cl_2 (20 mL) were stirred at room temperature for 16 hours before concentrated and purified by flash column chromatography (95:5 Petrol： EtOAc) to give a white solid with spectroscopic data in accordance with the literature.[1] 2.99 g, 85% yield; **mp** 79–80 ℃ {Lit.[1] 81– 82 ℃}; **^{1}H NMR (500 MHz, $CDCl_3$)** δ_H: 4.17 (2H, s, C(2)H_2), 7.43 – 7.61 (3H, m), 7.82 – 7.96

(4H, m); **^{19}F NMR (470 MHz, $CDCl_3$)** δ_F: -162.91 – -161.67 (2F, m), -157.80 (1F, t, *J* 21.5, ArC(4)*F*), -153.11 – -151.88 (2F, m).

Perfluorophenyl 2-(thiophen-2-yl)acetate (S26)[5]

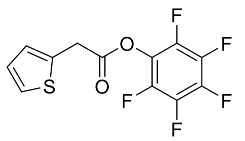

Following **General Procedure 1**, 2-(thiophen-2-yl)acetic acid (1.42 g, 10 mmol), pentafluorophenol (2.02 g, 11 mmol) and 1-ethyl-3-(3-dimethylaminopropyl) carbodiimide hydrochloride (2.30 g, 1.2 equiv.) in anhydrous CH_2Cl_2 (20 mL) were stirred at room temperature for 16 hours before concentrated and purified by flash column chromatography (95:5 Petrol : EtOAc) to give a colorless oil with spectroscopic data in accordance with the literature.[5] 1.91 g, 62% yield; **^{1}H NMR (400 MHz, $CDCl_3$)** δ_H: 4.22 (2H, s, C(2)H_2), 7.04 (1H, dd, *J* 5.1, 3.5), 7.06 – 7.12 (1H, m), 7.32 (1H, dd, *J* 5.2, 1.2); **^{19}F NMR (376 MHz, $CDCl_3$)** δ_F: -163.03 – -161.36 (2F, m), -157.54 (1F, t, *J* 21.4, ArC(4)*F*), -153.31 – -152.06 (2F, m).

Perfluorophenyl (E)-pent-3-enoate (S27)[6]

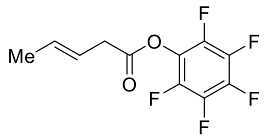

Following **General Procedure 1**, (*E*)-pent-3-enoic acid (1.00 g, 10 mmol), pentafluorophenol (2.02 g, 11 mmol) and 1-ethyl-3-(3-dimethylaminopropyl) carbodiimide hydrochloride (2.30 g, 1.2 equiv.) in anhydrous CH_2Cl_2 (20 mL) were stirred at room temperature for 16 hours before concentrated and purified by flash column chromatography (95:5 Petrol : EtOAc) to give a colorless oil with spectroscopic data in accordance with the literature.[6] 1.19 g, 45% yield; **^{1}H NMR (400 MHz, $CDCl_3$)** δ_H: 1.77 (3H, dq, *J* 6.4, 1.3, C(5)H_3), 3.39 (2H, dp, *J* 6.7, 1.2, C(2)H_2), 5.62 (1H, dtq, *J* 15.3, 6.9, 1.5, C(3)*H*), 5.76 (1H, dqt, *J* 15.3, 6.3, 1.3, C(4)*H*); **^{19}F NMR (377 MHz, $CDCl_3$)** δ_F: -163.46 – -161.79 (2F, m), -158.18 (1F, t, *J* 21.7, ArC(4)*F*), -153.80 – -152.32 (2F, m).

Perfluorophenyl 3-phenylpropanoate (35)[10]

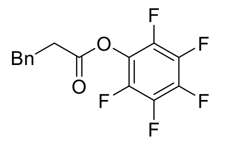

Following **General Procedure 1**, 3-phenylpropanoic acid (1.50 g, 10 mmol), pentafluorophenol (2.02 g, 11 mmol) and 1-ethyl-3-(3-dimethylaminopropyl) carbodiimide hydrochloride (2.30 g, 1.2 equiv.) in anhydrous CH_2Cl_2 (20 mL) were stirred at room temperature for 16 hours before concentrated and purified by flash column chromatography (95:5 Petrol : EtOAc) to give a colorless oil with spectroscopic data in accordance with the literature.[10] 2.30 g, 73% yield; **^{1}H NMR (400 MHz, CDCl$_3$)** δ_H: 2.95 – 3.09 (2H, m), 3.08 – 3.20 (2H, m), 7.25 – 7.34 (3H, m), 7.35 – 7.43 (2H, m); **^{19}F NMR (376 MHz, CDCl$_3$)** δ_F: -163.08 – -161.49 (2F, m), -158.09 (1F, t, *J* 21.4), -153.29 – -152.21 (2F, m).

Perfluorophenyl 2-(phenylthio)acetate (36)[8]

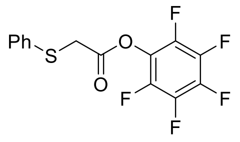

Following **General Procedure 1**, 2-(phenylthio)acetic acid (1.68 g, 10 mmol), pentafluorophenol (2.02 g, 11 mmol) and 1-ethyl-3-(3-dimethylaminopropyl) carbodiimide hydrochloride (2.30 g, 1.2 equiv.) in anhydrous CH_2Cl_2 (20 mL) were stirred at room temperature for 16 hours before concentrated and purified by flash column chromatography (95:5 Petrol : EtOAc) to give a white solid with spectroscopic data in accordance with the literature.[8] 1.97 g, 59% yield; **mp** 50–51 ℃; **^{1}H NMR (400 MHz, CDCl$_3$)** δ_H: 3.93 (2H, s, C(2)*H*$_2$), 7.31 – 7.40 (3H, m), 7.51 – 7.54 (2H, m); **^{13}C NMR (101 MHz, CDCl$_3$)** δ_C: 36.4 (*C*(2)H$_2$), 124.9 (m, Ar$_F$*C*(1)), 128.1 (SAr*C*(4)H), 129.3 (SAr*C*(3,5)H), 131.2 (SAr*C*(2,6)H), 133.4 (SAr*C*(1)), 137.9 (dm, *J* 250, Ar$_F$CF), 139.7 (dm, *J* 250, Ar$_F$CF),141.0 (dm, *J* 250, Ar$_F$CF), 165.9 (*C*(1)); **^{19}F NMR (377 MHz, CDCl$_3$)** δ_F: -163.61 – -161.76 (2F, m), -157.45 (1F, t, *J* 21.7), -153.61 – -151.59 (2F, m); **IR** ν_{max} (film) 1782 (C=O); **HRMS** (EI$^+$) $C_{14}H_7F_5O_2S$ [M]$^+$ found 334.0082, requires 334.0087 (–1.5 ppm).

4.2 Data for hemiaminal ethers

***N,N*-Dibenzyl-1-methoxymethanamine (5)**

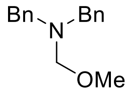

Following **General Procedure 2**, a mixture of dibenzylamine (5.91 g, 30 mmol), paraformaldehyde (900 mg, 30 mmol), K_2CO_3 (6.21 g, 45 mmol), and anhydrous Na_2SO_4 (6.39 g, 45 mmol) in anhydrous MeOH (30 mL) was stirred at room temperature for 12 h, then filtered and concentrated to give the crude mixture as an oil, which was subsequently distilled under vacuum to obtain desired hemiaminal ether as a colorless liquid. 4.33 g, 60% yield; **^{1}H NMR (400 MHz, CDCl$_3$)** δ_H: 3.29 (3H, s, OC*H*$_3$), 3.88 (4H, s, NC*H*$_2$Ph), 4.08 (2H, s, NC*H*$_2$OCH$_3$), 7.03 – 7.55 (10H, m, PhC*H*); **^{13}C NMR (101 MHz, CDCl$_3$)** δ_C: 55.4 (NCH$_2$Ph), 55.7 (OCH$_3$), 85.4 (NCH$_2$OCH$_3$), 127.0 (CH$_2$Ar*C*(4)H), 128.3 (CH$_2$Ar*C*(3,5)H), 128.9 (CH$_2$Ar*C*(2,6)H), 139.3 (CH$_2$Ar*C*(1)).

***N*-Benzyl-*N*-(4-fluorobenzyl)-1-methoxymethanamine (S28)**

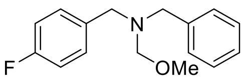

Following **General Procedure 2**, a mixture of *N*-benzyl-1-(4-fluorophenyl)methanamine (6.45 g, 30 mmol), paraformaldehyde (900 mg, 30 mmol), K_2CO_3 (6.21 g, 45 mmol), and anhydrous Na_2SO_4 (6.39 g, 45 mmol) in anhydrous MeOH (30 mL) was stirred at room temperature for 12 h, then filtered and concentrated to give the crude mixture as an oil, which was subsequently distilled under vacuum to obtain desired hemiaminal ether as a colorless liquid. 4.27 g, 55% yield; **^{1}H NMR (500 MHz, CDCl$_3$)** δ_H: 3.27 (3H, s, OC*H*$_3$), 3.82 (2H, s, NC*H*$_2$Ar), 3.85 (2H, s, NC*H*$_2$Ar), 4.05 (2H, s, NC*H*$_2$OCH$_3$), 6.94 – 7.10 (2H, m, Ar$_F$C(3,5)*H*), 7.23 – 7.31 (1H, m, ArC(4)*H*), 7.33 – 7.43 (6H, m); **^{19}F NMR (470 MHz, CDCl$_3$)** δ_F: -116.02; **^{13}C NMR (126 MHz, CDCl$_3$)** δ_C: 54.6 (NCH$_2$Ar), 55.4 (NCH$_2$Ar), 55.7 (OC*H*$_3$), 85.3 (NCH$_2$O), 115.03 (d, *J* 21.2, Ar$_F$*C*(3,5)H), 127.1 (Ar*C*(4)H), 128.3 (Ar*C*(3,5)H), 128.9 (Ar*C*(2,6)H), 130.38 (d, *J* 7.9, Ar$_F$*C*(2,6)H), 134.90 (H, d, *J* 3.0, Ar$_F$*C*(1)), 139.1 (Ar*C*(1)), 161.99 (d, *J* 244.6, Ar$_F$*C*(4)F).

***N*-Benzyl-*N*-(4-bromobenzyl)-1-methoxymethanamine (S29)**

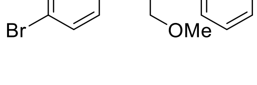

Following **General Procedure 2**, a mixture of *N*-benzyl-1-(4-bromophenyl)methanamine (8.28 g, 30 mmol), paraformaldehyde (900 mg, 30 mmol), K_2CO_3 (6.21 g, 45 mmol), and anhydrous Na_2SO_4 (6.39 g, 45 mmol) in anhydrous MeOH (30 mL) was stirred at room temperature for 12 h, then filtered and concentrated to give the crude mixture as an oil, which was subsequently distilled under vacuum to obtain desired hemiaminal ether as a colorless liquid. 5.18 g, 54% yield; **^{1}H NMR (500 MHz, $CDCl_3$)** δ_H: 3.26 (3H, s, OCH_3), 3.80 (2H, s, NCH_2Ar), 3.84 (2H, s, NCH_2Ar), 4.04 (2H, s, NCH_2O), 7.22 – 7.32 (4H, m), 7.32 – 7.40 (3H, m), 7.47 (2H, d, *J* 7.9, Ar$_{Br}$*C*(3,5)H); **^{13}C NMR (126 MHz, $CDCl_3$)** δ_C: 54.7 (N*C*H_2Ar), 55.5 (N*C*H_2Ar), 55.7 (O*C*H_3), 85.3 (N*C*H_2O), 120.8 (Ar$_{Br}$*C*(4)-Br), 127.1 (Ar*C*(4)H), 128.3 (Ar*C*(3,5)H), 128.9 (Ar*C*(2,6)H), 130.6 (Ar$_{Br}$*C*(3,5)H), 131.4 (Ar$_{Br}$*C*(2,6)H), 138.3 (Ar$_{Br}$*C*(1)), 139.0 (Ar*C*(1)).

***N*-Benzyl-1-methoxy-*N*-(4-methoxybenzyl)methanamine (S30)**

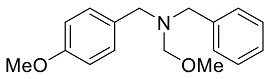

Following **General Procedure 2**, a mixture of *N*-benzyl-1-(4-methoxyphenyl)methanamine (6.81 g, 30 mmol), paraformaldehyde (900 mg, 30 mmol), K_2CO_3 (6.21 g, 45 mmol), and anhydrous Na_2SO_4 (6.39 g, 45 mmol) in MeOH (30 mL) was stirred at room temperature for 12 h, then filtered and concentrated to give the crude mixture as an oil, which was subsequently distilled under vacuum to obtain desired hemiaminal ether as a colorless liquid. 3.82 g, 47% yield; **^{1}H NMR (500 MHz, $CDCl_3$)** δ_H: 3.28 (3H, s, CH_2OCH_3), 3.81 (2H, s, NCH_2Ar), 3.84 (3H, s, Ar-OCH_3), 3.87 (2H, s, NCH_2Ar), 4.07 (2H, s, NCH_2O), 6.91 (2H, d, *J* 8.2, Ar$_{MeO}$*C*(3,5)H), 7.24 – 7.51 (7H, m); **^{13}C NMR (126 MHz, $CDCl_3$)** δ_C: 54.7 (N*C*H_2Ar), 55.3 (Ar-O*C*H_3), 55.3 (N*C*H_2Ar), 55.7 (CH_2O*C*H_3), 85.3 (N*C*H_2O), 113.7 (Ar$_{MeO}$*C*(3,5)H), 127.0 (Ph*C*(4)H), 128.3 (Ph*C*(3,5)H), 128.9 (Ph*C*(2,6)H), 130.1 (Ar$_{MeO}$*C*(2,6)H), 131.3 (Ar$_{MeO}$*C*(1)), 139.4 (Ph*C*(1)), 158.7 (Ar*C*-OMe).

***N*-Allyl-*N*-(methoxymethyl)prop-2-en-1-amine (S31)**[11]

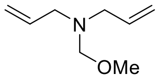

Following **General Procedure 2**, a mixture of diallylamine (2.91 g, 30 mmol), paraformaldehyde (900 mg, 30 mmol), K_2CO_3 (6.21 g, 45 mmol), and anhydrous Na_2SO_4 (6.39 g, 45 mmol) in anhydrous MeOH (30 mL) was stirred at room temperature for 12 h, then filtered and concentrated to give the crude mixture as an oil, which was subsequently distilled under vacuum to obtain desired hemiaminal ether as a colorless liquid. 2.75 g, 65% yield; **^{1}H NMR (400 MHz, $CDCl_3$)** δ_H: 3.27 (3H, s, OCH_3), 3.30 (4H, dt, *J* 6.4, 1.2, NCH_2CH), 4.13 (2H, s, NCH_2O), 5.01 – 5.33 (4H, m, CH=CH_2), 5.85 (2H, ddt, *J* 16.7, 10.1, 6.5, C*H*=CH$_2$); **^{13}C NMR (126 MHz, $CDCl_3$)** δ_C: 54.4 (NCH$_2$CH), 55.6 (OCH$_3$), 86.0 (NCH$_2$O), 117.3 (CH=CH$_2$), 136.1 (CH=CH$_2$).

***N*-Benzyl-1-methoxy-*N*-methylmethanamine (32)**[11]

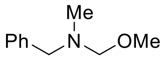

Following **General Procedure 2**, a mixture of *N*-methyl-1-phenylmethanamine (3.63 g, 30 mmol), paraformaldehyde (900 mg, 30 mmol), K_2CO_3 (6.21 g, 45 mmol), and anhydrous Na_2SO_4 (6.39 g, 45 mmol) in MeOH (30 mL) was stirred at room temperature for 12 h, then filtered and concentrated to give the crude mixture as an oil, which was subsequently distilled under vacuum to obtain desired hemiaminal ether as a colorless liquid. 2.23 g, 45% yield; **^{1}H NMR (300 MHz, $CDCl_3$)** δ_H: 2.44 (3H, s, NCH_3), 3.35 (3H, s, OCH_3), 3.79 (2H, s, NCH_2Ph), 4.12 (2H, s, NCH_2O), 7.19 – 7.51 (5H, m, PhC*H*); **^{13}C NMR (126 MHz, $CDCl_3$)** δ_C: 39.4 (NCH$_3$), 56.0 (OCH$_3$), 89.2 (NCH$_2$Ph), 127.0 (PhC(4)H), 128.3 (PhC(3,5)H), 128.8 (PhC(2,6)H), 139.1 (PhC(1)).

1-(Methoxymethyl)piperidine (33)[11]

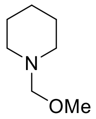

Following **General Procedure 2**, a mixture of piperidine (2.55 g, 30 mmol), paraformaldehyde (900 mg, 30 mmol), K_2CO_3 (6.21 g, 45 mmol), and anhydrous Na_2SO_4 (6.39 g, 45 mmol) in MeOH (30 mL) was stirred at room temperature for 12 h, then filtered and concentrated to give the crude mixture as an oil, which was subsequently distilled under vacuum to obtain desired hemiaminal ether as a colorless liquid. 0.77g, 20% yield; **^{1}H NMR (500 MHz, $CDCl_3$)** δ_H: 1.38 – 1.48 (2H, m, C(4)H_2),

1.48 – 1.57 (4H, m, C(3,5)H_2), 2.39 – 2.79 (4H, m, C(2,6)H_2), 3.29 (3H, s, OCH_3), 4.01 (2H, s, NCH_2O); **^{13}C NMR (126 MHz, CDCl$_3$)** δ_C: 24.2 (C(4)H$_2$), 26.1 (C(3,5)H$_2$), 50.7 (C(2,6)H$_2$), 56.2 (OCH$_3$), 90.9 (NCH$_2$O).

2-(Methoxymethyl)-1,2,3,4-tetrahydroisoquinoline (34)[2]

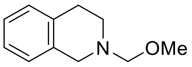

Following **General Procedure 2**, a mixture of 1,2,3,4-tetrahydroisoquinoline (3.99 g, 30 mmol), paraformaldehyde (900 mg, 30 mmol), K_2CO_3 (6.21 g, 45 mmol), and anhydrous Na_2SO_4 (6.39 g, 45 mmol) in anhydrous MeOH (30 mL) was stirred at room temperature for 12 h, then filtered and concentrated to give the crude mixture as an oil, which was subsequently distilled under vacuum to obtain desired hemiaminal ether as a colorless liquid. 1.96 g, 37% yield; **^{1}H NMR (300 MHz, CDCl$_3$)** δ_H: 2.88 – 2.97 (2H, m, NC(3)H_2), 2.98 – 3.05 (2H, m, NC(4)H_2), 3.41 (3H, s, OCH_3), 3.94 (2H, s, NC(1)H_2), 4.25 (2H, s, NCH_2O), 7.00 – 7.11 (1H, m), 7.12 – 7.21 (3H, m).

***N,N,N′,N′*-tetrabenzylmethanediamine (44)**[12]

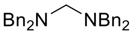

Following optimized literature procedure,[12] a mixture of dibenzylamine (11.8 g, 60 mmol) and paraformaldehyde (900 mg, 30 mmol) in MeCN (30 mL) was stirred at room temperature for 12 h. Then solvent was removed to afford crude product which was recrystallized to give the title compound as a white crystal. 9.50 g, 78% yield; **mp** 98–99 ℃; **^{1}H NMR (400 MHz, CDCl$_3$)** δ_H: 3.14 (2H, s), 3.66 (8H, s), 6.81 – 7.68 (20H, m).

(*S*)-*N*-Benzyl-*N*-(methoxymethyl)-1-phenylethan-1-amine (50)[13]

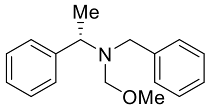

Following **General Procedure 2**, a mixture of (*S*)-*N*-benzyl-1-phenylethan-1-amine (6.33 g, 30 mmol), paraformaldehyde (900 mg, 30 mmol), K_2CO_3 (6.21 g, 45 mmol), and anhydrous Na_2SO_4 (6.39 g, 45 mmol) in anhydrous MeOH (30 mL) was stirred at room temperature for 12 h, then filtered and concentrated to give the crude mixture as an oil, which was subsequently distilled under vacuum to obtain desired hemiaminal

ether as a colorless liquid. 4.20 g, 55% yield; **^{1}H NMR (400 MHz, $CDCl_3$)** δ_H: 1.53 (3H, d, *J* 6.8, CHC*H*3), 3.23 (3H, s, OC*H*3), 3.80 (1H, d, *J* 13.6, NC*H*^{A}H^{B}Ph), 3.85 (1H, d, *J* 13.6, NH^A*H*^{B}Ph), 4.01 (1H, d, *J* 9.4, NC*H*^{A}H^{B}O), 4.14 (1H, q, *J* 6.7, C*H*CH3), 4.26 (1H, d, *J* 9.4, NH^A*H*^{B}O), 7.24 – 7.45 (8H, m), 7.46 – 7.54 (2H, m).

4.3 Data for isothiourea salts

(*R*)-BTM•HCl

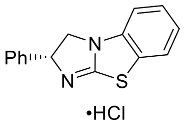

Following **General Procedure 3**, (*R*)-BTM (252 mg, 1.0 mmol) was treated with Et_2O•HCl (2 M, 1 mL) in Et_2O (4 mL) to give (*R*)-BTM•HCl as a white solid with spectroscopic data in accordance with the literature.[3] 230 mg, 80% yield; **mp** 202–203 ℃; **^{1}H NMR (400 MHz, DMSO-*d*$_6$)** δ_H: 4.39 (1H, dd, *J* 10.6, 8.3, C(3)*H*^{A}H^B), 4.99 (1H, t, *J* 10.7, C(3)H^A*H*^{B}), 5.98 (1H, dd, *J* 11.3, 8.3, C(2)*H*), 7.36 – 7.51 (4H, m, Ar*H*), 7.49 – 7.61 (4H, m, Ar*H*), 8.06 (1H, d, *J* 7.9, Ar*H*), 11.29 (1H, brs, N*H*$^+$).

(2*S*,3*R*)-HyperBTM•HCl

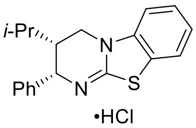

Following **General Procedure 3**, (2*S*,3*R*)-HyperBTM (308 mg, 1.0 mmol) was treated with Et_2O•HCl (2 M, 1 mL) in Et_2O (4 mL) to give (2*S*,3*R*)-HyperBTM•HCl as a white solid with spectroscopic data in accordance with the literature.[3] 285 mg, 83% yield; **mp** 230 ℃ (decompose); **^{1}H NMR (500 MHz, $CDCl_3$)** δ_H: 0.92 (3H, d, *J* 6.7, C*H*3), 1.19 (3H, d, *J* 6.5, C*H*3), 1.33–1.47 (1H, m, C*H*(CH3)), 2.13 (1H, ddt, *J* 11.9, 9.4, 4.7, C(3)*H*), 3.64 (1H, t, *J* 12.2, C(4)*H*^{A}H^B), 4.25 (1H, dd, J 12.7, 4.4, C(4)H^A*H*^{B}), 5.17 (1H, dd, *J* 4.4, 1.5, C(2)*H*), 7.09 – 7.14 (2H, m, Ar*H*), 7.30 – 7.43 (5H, m, Ar*H*), 7.51 (1H, m, Ar*H*), 7.64 (1H, d, *J* 7.9, Ar*H*).

(*R*)-BTM•HBF$_4$

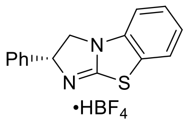

Following **General Procedure 3**, (*R*)-BTM (252 mg, 1.0 mmol) was treated with Et_2O•HBF_4 (162 mg, 1.0 mmol) in Et_2O (4 mL) to give (*R*)-BTM•HBF_4 as an off-white solid with spectroscopic data in accordance with the literature.[3] 261 mg, 77% yield; **mp** 182–183 ℃; **^{1}H NMR (500 MHz, DMSO-*d*$_6$)** δ_H: 4.41 (1H, dd, *J* 10.6, 8.3, C(3)*H*^{A}H^B), 4.99 (1H, t, *J* 10.8, C(3)H^A*H*^{B}), 5.96 (1H, dd, *J* 10.8, 8.3, C(2)*H*), 7.39 – 7.52 (4H, m), 7.51 – 7.62 (4H, m), 8.06 (1H, dd, *J* 8.2, 1.1), 10.86 (1H, brs, N-*H*); **^{19}F NMR (377 MHz, DMSO-*d*$_6$)** δ_F: -148.22.

(*R*)-BTM•HOTf

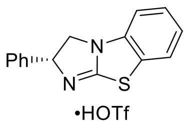

Following **General Procedure 3**, (*R*)-BTM (252 mg, 1.0 mmol) was treated with HOTf (150 mg, 1.0 mmol) in Et_2O (4 mL) to give (*R*)-BTM•HOTf as an off-white solid. 293 mg, 73% yield; **mp** 184–185 ℃; **^{1}H NMR (500 MHz, DMSO-*d*$_6$)** δ_H: 4.42 (1H, dd, *J* 10.6, 8.3, C(3)*H*^{A}H^B), 4.99 (1H, t, *J* 10.8, C(3)H^A*H*^{B}), 5.97 (1H, dd, *J* 10.9, 8.3, C(2)*H*), 7.40 – 7.52 (4H, m), 7.52 – 7.63 (4H, m), 8.07 (1H, dd, *J* 8.2, 1.1), 10.87 (1H, s); **^{19}F NMR (377 MHz, DMSO-*d*$_6$)** δ_F: -77.75; **^{13}C NMR (126 MHz, DMSO-*d*$_6$)** δ_C: 53.5 (*C*(3)H$_2$), 66.8 (*C*(2)H), 113.3 (ArCH), 125.2 (ArCH), 125.3 (ArCH), 127.7 (Ph*C*(2,6)H), 127.9 (Ar*C*-S), 128.6 (ArCH), 129.5 (Ph*C*(3,5)H), 129.5 (ArCH), 135.3 (Ar*C*-N), 139.1 (Ph*C*(1)), 170.1 (N=*C*).

(*R*)-BTM•HOOCCF$_3$

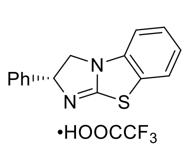

Following **General Procedure 3**, (*R*)-BTM (252 mg, 1.0 mmol) was treated with CF_3COOH (114 mg, 1.0 mmol) in Et_2O (4 mL) to give (*R*)-BTM•HOOCCF$_3$ as an off-white solid. 285 mg, 78% yield; **^{1}H NMR (500 MHz, DMSO-*d*$_6$)** δ_H: 4.38 (1H, dd, *J* 10.6, 8.3, C(3)*H*^{A}H^B), 4.97 (1H, t, *J* 10.7, C(3)H^A*H*^{B}), 5.96 (1H, dd, *J* 10.8, 8.3, C(2)*H*), 7.35 – 7.53 (5H, m, Ar*H*), 7.52 – 7.60 (3H, m, Ar*H*), 8.04 (1H, dd, *J* 8.1, 1.1, Ar*H*), 11.51 (1H, brs, N*H*$^+$); **^{19}F NMR (377 MHz, DMSO-*d*$_6$)** δ_F: -73.66; **^{13}C NMR (126 MHz, DMSO-*d*$_6$)** δ_C:

53.4 (C(3)H_2), 67.2 (C(2)H), 113.1 (ArCH), 124.9 (ArCH), 125.2 (ArCH), 127.6 (PhC(2,6)H), 128.0 (ArC-S), 128.5 (ArCH), 129.36 (ArCH), 129.43 (ArC(3,5)H), 135.3 (ArC-N), 139.4 (PhC(1)), 169.9 (N=C).

(*R*)-BTM•HBr

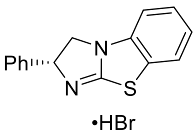

Following **General Procedure 3**, (*R*)-BTM (504 mg, 2.0 mmol) was treated with aqueous HBr (48 wt% in H_2O, 1.35 g, 8 mmol) in DCM/MeCN (4:1, 100 mL) to give (*R*)-BTM•HBr as a white solid. 560 mg, 84% yield; **^{1}H NMR (500 MHz, $CDCl_3$)** δ_H: 4.29 (1H, dd, *J* 10.2, 8.0, C(3)H^AH^B), 5.25 (1H, t, *J* 10.8, C(3)H^AH^B), 6.27 (1H, dd, *J* 10.9, 8.0, C(2)*H*), 7.28 (1H, d, *J* 8.0, Ar*H*), 7.32 – 7.42 (4H, m, Ar*H*), 7.43 – 7.48 (2H, m, Ar*H*), 7.48 – 7.53 (1H, m, Ar*H*), 7.69 (1H, d, *J* 8.0, Ar*H*), 11.58 (1H, s, NH^+); **^{13}C NMR (126 MHz, $CDCl_3$)** δ_C: 54.0 (C(3)H_2), 66.6 (C(2)H), 112.0 (ArCH), 124.4 (ArCH), 125.4 (ArCH), 126.3 (PhC(2,6)H), 128.1 (ArC-S), 128.5 (ArCH), 129.4 (ArCH), 129.5 (ArC(3,5)H), 133.8 (ArC-N), 137.4 (PhC(1)), 170.2 (N=C).

(*R*)-BTM•HI

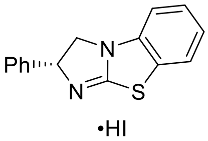

Following **General Procedure 3**, (*R*)-BTM (504 mg, 2.0 mmol) was treated with aqueous HI (47 wt% in H_2O, 2.18 g, 8 mmol) in DCM/MeCN (4:1, 100 mL) to give (*R*)-BTM•HI as a slightly yellow solid. 640 mg, 84% yield; **^{1}H NMR (500 MHz, $CDCl_3$)** δ_H: 4.17 – 4.64 (1H, m, C(3)H^AH^B), 5.32 (1H, t, *J* 10.7, C(3)H^AH^B), 6.33 (1H, dd, *J* 11.0, 8.1, C(2)*H*), 7.30 – 7.43 (5H, m, Ar*H*), 7.43 – 7.54 (3H, m, Ar*H*), 7.66 – 7.77 (1H, m, Ar*H*), 10.56 (1H, s, NH^+); **^{13}C NMR (126 MHz, $CDCl_3$)** δ_C: 54.2 (C(3)H_2), 66.6 (C(2)H), 112.3 (ArCH), 124.5 (ArCH), 125.6 (ArCH), 126.4 (PhC(2,6)H), 127.9 (ArC-S), 128.6 (ArCH), 129.45 (ArCH), 129.49 (ArC(3,5)H), 133.6 (ArC-N), 137.2 (PhC(1)), 169.5 (N=C).

(*R*)-BTM•HPF$_6$

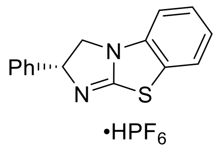

Following **General Procedure 3**, (*R*)-BTM (504 mg, 2.0 mmol) was treated with aqueous HPF_6 (55 wt% in H_2O, 637 mg, 2.4 mmol) in DCM/MeCN (4:1, 100 mL) to give (*R*)-BTM•HPF_6 as a white solid. 680 mg, 85% yield; **^{1}H NMR (500 MHz, DMSO-*d*$_6$)** δ_H: 4.36 – 4.45 (1H, m, C(3)H^AH^B), 4.90 – 5.06 (1H, m, C(3)H^AH^B), 5.89 – 6.04 (1H, m, C(2)*H*), 7.39 – 7.52 (4H, m, Ar*H*), 7.52 – 7.56 (1H, m, Ar*H*), 7.56 – 7.62 (3H, m, Ar*H*), 8.06 (1H, d, *J* 8.1, Ar*H*), 10.87 (1H, s, NH^+); **^{13}C NMR (126 MHz, DMSO-*d*$_6$)** δ_C: 53.5 (*C*(3)H_2), 66.9 (*C*(2)H), 113.3 (ArCH), 125.1 (ArCH), 125.3 (ArCH), 127.7 (Ph*C*(2,6)H), 127.9 (Ar*C*-S), 128.6 (ArCH), 129.48 (ArCH), 129.50 (Ar*C*(3,5)H), 135.3 (ArC-N), 139.2 (Ph*C*(1)), 170.1 (N=C); **^{31}P NMR (202 MHz, DMSO-*d*$_6$)** δ_P: -144.18 (hept, *J* 711.3); **^{19}F NMR (471 MHz, DMSO- *d*$_6$)** δ_F: -70.09 (d, *J* 710.8).

(*R*)-BTM•H_3PO_4

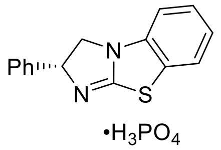

Following **General Procedure 3**, (*R*)-BTM (504 mg, 2.0 mmol) was treated with aqueous H_3PO_4 (85 wt% in H_2O, 230 mg, 2.0 mmol) in DCM/MeCN (4:1, 100 mL) to give (*R*)-BTM•H_3PO_4 as a white solid. 250 mg, 36% yield; **^{1}H NMR (400 MHz, DMSO-*d*$_6$)** δ_H: 3.73 – 3.88 (1H, m, C(3)H^AH^B), 4.39 – 4.58 (1H, m, C(3)H^AH^B), 5.58 – 5.75 (1H, m, C(2)*H*), 6.92 – 7.15 (2H, m, Ar*H*), 7.21 – 7.51 (6H, m, Ar*H*), 7.54 – 7.74 (1H, m, Ar*H*), 9.70 (3H, brs); **^{13}C NMR (126 MHz, DMSO-*d*$_6$)** δ_C: 52.9 (*C*(3)H_2), 73.1 (*C*(2)H), 110.4 (ArCH), 122.6 (ArCH), 124.3 (ArCH), 126.9 (Ph*C*(2,6)H), 127.1 (Ar*C*-S), 127.6 (ArCH), 128.1 (ArCH), 129.1 (Ar*C*(3,5)H), 137.0 (Ar*C*-N), 142.7 (Ph*C*(1)), 166.7 (N=C); **^{31}P NMR (162 MHz, DMSO-*d*$_6$)** δ_P: 0.26.

4.4 Data for β^2-amino-esters

Methyl (*S*)-3-(dibenzylamino)-2-phenylpropanoate (9)

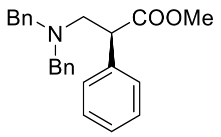

Following **General Procedure 4**, perfluorophenyl 2-phenylacetate **8** (60.4 mg, 0.2 mmol), *N,N*-dibenzyl-1-methoxymethanamine **5** (72.4 mg, 0.3 mmol), (*R*)-BTM·HCl (2.9 mg, 0.01 mmol) and 4 Å molecular sieves (100 mg) in anhydrous THF (2.0 mL) at room temperature for 24 hours then anhydrous MeOH (0.5 mL) and DMAP (4.9 mg, 0.04 mmol) at room temperature for 4 hours gave the crude material that was purified by column chromatography (120:1 Petrol : EtOAc) to give the desired compound **9** as a colorless oil (58.2 mg, 81% yield). $[\alpha]_D^{20}$ +6.2 (*c* 1.0, $CHCl_3$); **Chiral HPLC analysis**: Chiralcel OD-H (98.5:1.5 hexane:IPA, flow rate 1 mLmin^{-1}, 211 nm, 30 °C) t_R (major): 6.7 min, t_R (minor): 8.2 min, 96:4 er; **IR** ν_{max} (film) 1734 (C=O, ester); **^{1}H NMR (400 MHz, $CDCl_3$)** δ_H: 2.94 (1H, dd, *J* 12.9, 6.3, C(3)$H^AH^B$$NBn_2$), 3.40 (1H, dd, *J* 12.9, 9.3, C(3)$H^AH^B$$NBn_2$), 3.70 (2H, d, *J* 13.6, NCH^AH^BPh), 3.75 (3H, s, OCH_3), 3.79 (2H, d, *J* 13.6, NCH^AH^BPh), 4.00 (1H, dd, *J* 9.2, 6.3, C(2)*H*), 7.31 – 7.45 (15H, m, Ar*H*); **^{13}C NMR (101 MHz, $CDCl_3$)** δ_C: 50.9 (*C*(1)H), 52.0 (OCH_3), 57.6 (*C*(3)H_2NBn_2), 58.7 (NCH_2Ph), 127.1 (CH_2Ar*C*(4)H), 127.5 (C(2)HAr*C*(4)H), 128.3 (CH_2Ar*C*(3,5)H), 128.4 (C(2)HAr*C*(3,5)H), 128.6 (C(2)HArC(2,6)H), 129.1 (CH_2ArC(2,6)H), 137.5 (*C*(2)HArC(1)), 139.3 (NCH_2Ar*C*(1)), 173.6 (*C*(1)); **HRMS (ESI$^+$)** $C_{24}H_{26}NO_2$ $[M+H]^+$ found 360.1946, requires 360.1958 (–3.3 ppm).

Methyl (*S*)-3-(dibenzylamino)-2-(4-(dimethylamino)phenyl)propanoate (10)

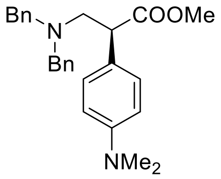

Following **General Procedure 4**, perfluorophenyl 2-(4-(dimethylamino)phenyl)acetate **S10** (69.1 mg, 0.2 mmol), *N,N*-dibenzyl-1-methoxymethanamine **5** (72.4 mg, 0.3 mmol), (*R*)-BTM·HCl (2.9 mg, 0.01 mmol) and 4 Å molecular sieves (100 mg) in anhydrous THF (2.0 mL) at room temperature for 3 days then anhydrous MeOH (0.5 mL) and DMAP (4.9 mg, 0.04 mmol) for 4 hours gave the crude material that was purified by column chromatography (40:1 Petrol : EtOAc) to give the desired compound **10** as a

yellow solid (61.9 mg, 77%); **mp** 58–59 ºC; $[\alpha]_D^{20}$ +10.3 (*c* 1.0, $CHCl_3$); **Chiral HPLC analysis**: Chiralcel OD-H (98.5:1.5 hexane:IPA, flow rate 1 mLmin^{-1}, 211 nm, 30 °C) t_R (*S*): 9.3 min, t_R (*R*): 11.5 min, 95:5 er; **IR** ν_{max} (film) 1732 (C=O); **^{1}H NMR (400 MHz, $CDCl_3$)** δ_H: 2.74 (1H, dd, *J* 12.8, 5.8, C(3)H^AH^B), 2.96 (6H, s, NCH_3), 3.28 (1H, dd, *J* 12.8, 9.9, C(3)H^AH^B), 3.55 (2H, d, *J* 13.7, NCH^AH^BPh), 3.65 (1H, s, OCH_3), 3.73 (2H, d, *J* 13.7, NCH^AH^BPh), 3.81 (1H, dd, *J* 9.9, 5.8, C(2)*H*), 6.65 – 6.72 (2H, m, ArC*H*), 7.05 – 7.16 (2H, m, ArC*H*), 7.22 – 7.39 (10H, m, ArC*H*); **^{13}C NMR (101 MHz, $CDCl_3$)** δ_C: 40.7 (NCH_3), 49.9 (*C*(2)H), 51.8 (OCH_3), 57.7 (*C*(3)H_2), 58.4 (NCH_2Ph), 112.6 (C(2)HAr*C*(3,5)H), 125.1 (C(2)HAr*C*(1)), 126.9 (NCH_2Ar*C*(4)H), 128.1 (NCH_2Ar*C*(3,5)H), 128.8 (C(2)HAr*C*(2,6)H), 129.0 (NCH_2Ar*C*(2,6)H), 139.3 (NCH_2Ar*C*(1)), 150.0 (Ar*C*(4)-NMe_2), 174.1 (*C*(1)); **HRMS (ESI$^+$)** $C_{26}H_{31}N_2O_2$ $[M+H]^+$ found 403.2369, requires 403.2380 (–2.7 ppm).

Methyl (*S*)-3-(dibenzylamino)-2-(4-methoxyphenyl)propanoate (11)

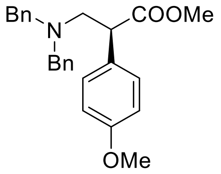

Following **General Procedure 4**, perfluorophenyl 2-(4-methoxyphenyl)acetate **S11** (66.4 mg, 0.2 mmol), *N*,*N*-dibenzyl-1-methoxymethanamine **5** (72.4 mg, 0.3 mmol), (*R*)-BTM·HCl (2.9 mg, 0.01 mmol) and 4 Å molecular sieves (100 mg) in anhydrous THF (2.0 mL) for 48 hours then anhydrous MeOH (0.5 mL) and DMAP (4.9 mg, 0.04 mmol) for 4 hours gave the crude material that was purified by column chromatography (120:1 Petrol : EtOAc) to give the desired compound as a colorless oil (69.2 mg, 89% yield); $[\alpha]_D^{20}$ +16.1 (*c* 1.0, $CHCl_3$); **Chiral HPLC analysis**: Chiralcel OD-H (95:5 hexane:IPA, flow rate 1 mLmin^{-1}, 211 nm, 30 °C) t_R (*S*): 6.1 min, t_R (*R*): 7.2 min, 94:6 er; **IR** ν_{max} (film) 1734 (C=O), 1250 (C-O); **^{1}H NMR (400 MHz, $CDCl_3$)** δ_H: 2.83 (1H, dd, *J* 12.9, 6.3, C(3)$H^AH^B$$NBn_2$), 3.29 (1H, dd, *J* 12.7, 9.5, C(3)$H^AH^B$$NBn_2$), 3.63 (2H, d, *J* 13.6, NCH^AH^BPh), 3.70 (3H, s, COOCH_3), 3.73 (2H, d, *J* 13.8, NCH^AH^BPh), 3.84 (3H, s, Ar-OCH_3), 3.88 (1H, dd, *J* 9.1, 6.4, C(2)*H*), 6.84 – 6.92 (2H, m, ArC*H*), 7.13 – 7.23 (2H, m, ArC*H*), 7.25 – 7.42 (10H, m, ArC*H*); **^{13}C NMR (101 MHz, $CDCl_3$)** δ_C: 50.0 (*C*(2)H), 51.9 ($COOCH_3$), 55.3 (Ar-OCH_3), 57.6 (C(3)H_2), 58.6 (NCH_2Ph), 113.9 (C(2)HAr*C*(3,5)H),

127.0 (NCH$_2$ArC(4)H), 128.2 (NCH$_2$ArC(2,6)H), 129.0 (NCH$_2$ArC(3,5)H), 129.3 (C(2)HArC(2,6)H), 129.5 (C(2)HAr(1)), 139.2 (NCH$_2$ArC(1)), 158.9 (ArC-OCH$_3$), 173.8 (C(1)); **HRMS (ESI$^+$)** $C_{25}H_{28}NO_3$ [M+H]$^+$ found 390.2051, requires 390.2064 (–3.3 ppm).

Methyl (*S*)-3-(dibenzylamino)-2-(p-tolyl)propanoate (12)

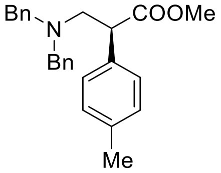

Following **General Procedure 4**, perfluorophenyl 2-(*p*-tolyl)acetate **S12** (63.2 mg, 0.2 mmol), *N*,*N*-dibenzyl-1-methoxymethanamine **5** (72.4 mg, 0.3 mmol), (*R*)-BTM·HCl (2.9 mg, 0.01 mmol) and 4 Å molecular sieves (100 mg) in anhydrous THF (2.0 mL) for 17 hours then anhydrous MeOH (0.5 mL) and DMAP (4.9 mg, 0.04 mmol) for 4 hours gave the crude material that was purified by column chromatography (120:1 Petrol : EtOAc, R_f 0.12) to give the desired compound as a colorless oil (67.9 mg, 91% yield). $[\alpha]_D^{20}$ +18.0 (*c* 1.0, CHCl$_3$); **Chiral HPLC analysis**: Chiralcel OD-H (97:3 hexane:IPA, flow rate 1 mLmin^{-1}, 211 nm, 30 °C) t_R (*S*): 5.1 min, t_R (*R*): 5.7 min, 95:5 er; **IR** ν_{max} (film) 1734 (C=O); **^{1}H NMR (400 MHz, CDCl$_3$)** δ_H: 2.37 (3H, s, Ar-C*H*$_3$), 2.79 (1H, dd, *J* 12.9, 6.1, C(3)*H^A*H^B), 3.29 (1H, dd, *J* 12.9, 9.5, C(3)H^A*H^B*), 3.59 (2H, d, *J* 13.6, C*H^A*H^{B}Ph), 3.67 (3H, s, OC*H*$_3$), 3.71 (2H, d, *J* 13.6, CH^A*H^B*Ph), 3.86 (1H, dd, *J* 9.5, 6.1, C(2)*H*), 7.13 (4H, s, ArC*H*), 7.23 – 7.39 (10H, m, ArC*H*); **^{13}C NMR (101 MHz, CDCl$_3$)** δ_C: 21.1 (Ar-CH$_3$), 50.5 (*C*(2)H), 51.9 (O*C*H$_3$), 57.6 (*C*(3)H$_2$), 58.5 (N*C*H$_2$Ph) 127.0 (NCH$_2$ArC(4)H), 128.1 (C(2)HArC(3,5)H), 128.2 (NCH$_2$ArC(3,5)H), 129.0 (NCH$_2$ArC(2,6)H), 129.2 (CHArC(2,6)H), 134.3 (C(2)HArC(1)), 137.0 (C(2)HArC(4)), 139.2 (NCH$_2$ArC(1)), 173.7(*C*(1)); **HRMS (ESI$^+$)** $C_{25}H_{28}NO_2$ [M+H]$^+$ found 373.2100, requires 374.2115 (–4.0 ppm).

Methyl (*S*)-2-(4-bromophenyl)-3-(dibenzylamino)propanoate (13)

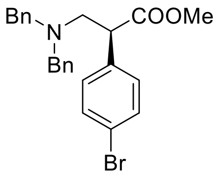

Following **General Procedure 4**, perfluorophenyl 2-(4-bromophenyl)acetate **S13** (76.2 mg, 0.2 mmol), *N*,*N*-dibenzyl-1-methoxymethanamine **5** (72.4 mg, 0.3 mmol), (*R*)-

BTM·HCl (2.9 mg, 0.01 mmol) and 4 Å molecular sieves (100 mg) in anhydrous THF (2.0 mL) for 41 hours then anhydrous MeOH (0.5 mL) and DMAP (4.9 mg, 0.04 mmol) for 4 hours gave the crude material that was purified by column chromatography (120:1 Petrol : EtOAc) to give the desired compound as a white solid (82.2 mg, 94%); **mp** 73–74 °C; $[\alpha]_D^{20}$ -10.1 (*c* 1.0, $CHCl_3$); **Chiral HPLC analysis**: Chiralcel OD-H (97:3 hexane:IPA, flow rate 1 $mLmin^{-1}$, 211 nm, 30 °C) t_R (*S*): 6.2 min, t_R (*R*): 7.6 min, 96:4 er; **IR** ν_{max} (film) 1736 (C=O); **^{1}H NMR (400 MHz, $CDCl_3$)** δ_H: 2.83 (1H, dd, *J* 13.0, 7.1, C(3)H^AH^B), 3.18 (1H, dd, *J* 13.0, 8.5, C(3)H^AH^B), 3.62 (4H, s, NCH_2Ph), 3.66 (3H, s, OCH_3), 3.81 (1H, dd, *J* 8.5, 7.1, C(2)*H*), 7.00 – 7.09 (2H, m, C(2)HArC(2,6)*H*), 7.20 – 7.36 (14H, m, ArC*H*), 7.36 – 7.45 (2H, m, C(2)HArC(3,5)*H*); **^{13}C NMR (101 MHz, $CDCl_3$)** δ 50.2 (*C*(2)H), 52.1 (OCH_3), 57.2 (*C*(3)H_2), 58.7 (NCH_2Ph), 121.3 (Ar*C*(4)-Br), 127.1 (NCH_2Ar*C*(4)H), 128.2 (NCH_2Ar*C*(3,5)H), 128.9 (NCH_2Ar*C*(2,6)H), 130.0 (C(2)HAr*C*(2,6)H), 131.6 (C(2)HAr*C*(3,5)H), 136.4 (C(2)HAr*C*(1)), 139.0 (NCH_2Ar*C*(1)), 173.1 (*C*(1)); **HRMS (ESI$^+$)** $C_{24}H_{25}BrNO_2$ $[M+H]^+$ found 438.1057, requires 438.1063 (–1.4 ppm).

Methyl (*S*)-2-(4-chlorophenyl)-3-(dibenzylamino)propanoate (14)

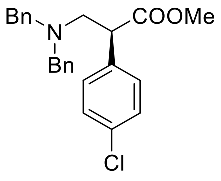

Following **General Procedure 4**, perfluorophenyl 2-(4-chlorophenyl)acetate **S14** (67.3 mg, 0.2 mmol), *N,N*-dibenzyl-1-methoxymethanamine **5** (72.4 mg, 0.3 mmol), (*R*)-BTM·HCl (2.9 mg, 0.01 mmol) and 4 Å molecular sieves (100 mg) in anhydrous THF (2.0 mL) for 24 hours then anhydrous MeOH (0.5 mL) and DMAP (4.9 mg, 0.04 mmol) for 4 hours gave the crude material that was purified by column chromatography (120:1 Petrol : EtOAc) to give the desired compound as a white solid (71.5 mg, 91%); **mp** 71–72 °C; $[\alpha]_D^{20}$ +11.4 (*c* 1.0, $CHCl_3$); **Chiral HPLC analysis**: Chiralcel OD-H (98.5:1.5 hexane:IPA, flow rate 1 $mLmin^{-1}$, 211 nm, 30 °C) t_R (*S*): 7.2 min, t_R (*R*): 9.5 min, 96:4 er; **IR** ν_{max} (film) 1734 (C=O); **^{1}H NMR (400 MHz, $CDCl_3$)** δ_H: 2.84 (1H, dd, *J* 13.0, 7.0, C(3)H^AH^B), 3.21 (1H, dd, *J* 13.0, 8.6, C(3)H^AH^B), 3.64 (4H, s, NCH_2Ph), 3.68 (3H, s, OCH_3), 3.85 (1H, dd, *J* 8.4, 7.2, C(2)*H*), 7.05 – 7.17 (2H, m, ArC*H*), 7.21 – 7.41 (12H, m,

ArC*H*); **^{13}C NMR (101 MHz, $CDCl_3$)** δ 50.1 (*C*(2)H), 52.1 (OCH_3), 57.3 (*C*(3)H_2), 58.7 (NCH_2Ph), 127.1 (NCH_2Ar*C*(4)H), 128.2 (NCH_2Ar*C*(3,5)H), 128.6 (C(2)HAr*C*(3,5)H), 129.0 (NCH_2Ar*C*(2,6)H), 129.7 (C(2)HAr*C*(2,6)H), 133.2 (C(2)HAr*C*(4)-Cl), 135.9 (C(2)HAr*C*(1)), 139.0 (NCH_2Ar*C*(1)), 173.2 (*C*(1)); **HRMS (ESI$^+$)** $C_{24}H_{25}ClNO_2$ $[M+H]^+$ found 394.1559, requires 394.1568 (–2.3 ppm).

Methyl (*S*)-3-(dibenzylamino)-2-(4-fluorophenyl)propanoate (15)

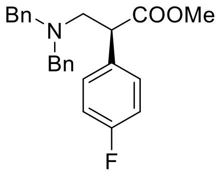

Following **General Procedure 4**, perfluorophenyl 2-(4-fluorophenyl)acetate **S15** (64.0 mg, 0.2 mmol), *N*,*N*-dibenzyl-1-methoxymethanamine **5** (72.4 mg, 0.3 mmol), (*R*)-BTM·HCl (2.9 mg, 0.01 mmol) and 4 Å molecular sieves (100 mg) in anhydrous THF (2.0 mL) for 24 hours then anhydrous MeOH (0.5 mL) and DMAP (4.9 mg, 0.04 mmol) for 4 hours gave the crude material that was purified by column chromatography (120:1 Petrol : EtOAc) to give the desired compound as a colorless oil (67.9 mg, 90%); $[\alpha]_D^{20}$ +2.8 (*c* 1.0, $CHCl_3$); **Chiral HPLC analysis**: Chiralcel OD-H (98.5:1.5 hexane:IPA, flow rate 1 mLmin^{-1}, 211 nm, 30 °C) t_R (*S*): 7.0 min, t_R (*R*): 9.1 min, 96:4 er; **IR** ν_{max} (film) 1734 (C=O); **^{1}H NMR (400 MHz, $CDCl_3$)** δ_H: 2.82 (1H, dd, *J* 12.9, 6.9, C(3)H^AH^B), 3.20 (1H, dd, *J* 12.9, 8.8, C(3)H^AH^B), 3.63 (4H, s, NCH_2Ph), 3.67 (3H, s, OCH_3), 3.85 (1H, dd, *J* 8.7, 6.9, C(2)*H*), 6.93 – 7.04 (2H, m, ArC*H*), 7.09 – 7.20 (2H, m, ArC*H*), 7.21 – 7.38 (10H, m, ArC*H*); **^{13}C NMR (101 MHz, $CDCl_3$)** δ_C: 50.0 (*C*(2)H), 52.0 (OCH_3), 57.4 (*C*(3)H_2), 58.6 (NCH_2Ph), 115.3 (d, *J* 21.3, C(2)HAr*C*(3,5)H), 127.0 (NCH_2ArC(4)H), 128.2 (NCH_2Ar*C*(3,5)H), 128.9 (NCH2Ar*C*(2,6)H), 129.8 (d, *J* 8.0, C(2)HAr*C*(2,6)H), 133.0 (d, *J* 3.1, C(2)HAr*C*(1)), 139.0 (NCH_2Ar*C*(1)), 162.1 (d, *J* 245.6, C(2)HAr*C*(4)-F), 173.4 (*C*(1)); **^{19}F NMR (282 MHz, $CDCl_3$)** δ_F: -115.35; **HRMS (ESI$^+$)** $C_{24}H_{25}FNO_2$ $[M+H]^+$ found 378.1855, requires 378.1864 (–2.4 ppm).

Methyl (*S*)-3-(dibenzylamino)-2-(4-(trifluoromethyl)phenyl)propanoate (16)

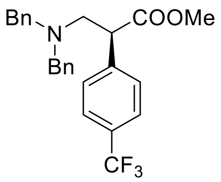

Following **General Procedure 4**, perfluorophenyl 2-(4-(trifluoromethyl)phenyl)acetate **S16** (74.0 mg, 0.2 mmol), *N*,*N*-dibenzyl-1-methoxymethanamine **5** (72.4 mg, 0.3 mmol), (*R*)-BTM·HCl (2.9 mg, 0.01 mmol) and 4 Å molecular sieves (100 mg) in anhydrous THF (2.0 mL) for 16 hours then anhydrous MeOH (0.5 mL) and DMAP (4.9 mg, 0.04 mmol) for 4 hours gave the crude material that was purified by column chromatography (120:1 Petrol : EtOAc) to give the desired compound as a white solid (72.5mg, 85%); **mp** 44–45 °C; $[\alpha]_D^{20}$ -4.4 (*c* 1.0, $CHCl_3$); **Chiral HPLC analysis**: Chiralcel OD-H (98.5:1.5 hexane:IPA, flow rate 1 mLmin^{-1}, 211 nm, 30 °C) t_R (*S*): 7.1 min, t_R (*R*): 9.6 min, 96:4 er; **IR** ν_{max} (film) 1738 (C=O), 1325 (C-O); **^{1}H NMR (400 MHz, $CDCl_3$)** δ_H: 2.91 (1H, dd, *J* 13.0, 7.4, C(3)H^AH^B), 3.24 (1H, dd, *J* 13.0, 8.2, C(3)H^AH^B), 3.62 (2H, d, *J* 13.6, NCH^AH^BPh), 2.68 (2H, d, *J* 13.6, NCH^AH^BPh), 3.70 (3H, s, OCH_3), 3.92 (1H, t, *J* 7.7, C(2)*H*), 7.22 – 7.36 (12H, m, ArC*H*), 7.56 (2H, d, *J* 8.1, ArC*H*); **^{13}C NMR (101 MHz, $CDCl_3$)** δ_C: 50.6 (*C*(2)H), 52.1 (O*C*H_3), 57.2 (*C*(3)H_2), 58.8 (N*C*H_2Ph), 124.2 (q, $^1J_{C\text{-}F}$ = 272.9 Hz, *C*F_3), 125.4 (q, $^3J_{C\text{-}F}$ = 3.7 Hz, C(2)HAr*C*(3,5)H), 127.1 (NCH_2Ar*C*(4)H), 128.3 (NCH_2Ar*C*(3,5)H), 128.7 (C(2)HAr*C*(2,6)H), 128.9 (NCH_2Ar*C*(2,6)H), 129.6 (q, *J* = 32.4 Hz, Ar*C*(4)CF_3), 138.9 (NCH_2Ar*C*(1)), 141.4 (C(2)HAr*C*(1)), 172.8 (*C*(1)); **^{19}F NMR (471 MHz, $CDCl_3$)** δ -62.42; **HRMS (ESI$^+$)** $C_{25}H_{25}F_3NO_2$ $[M+H]^+$ found 428.1821, requires 428.1832 (–2.6 ppm).

Methyl (*S*)-3-(dibenzylamino)-2-(4-nitrophenyl)propanoate (17)

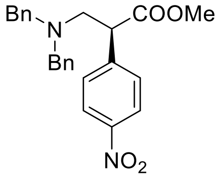

Following **General Procedure 4**, perfluorophenyl 2-(4-nitrophenyl)acetate **S17** (69.4 mg, 0.2 mmol), *N*,*N*-dibenzyl-1-methoxymethanamine **5** (72.4 mg, 0.3 mmol), (*R*)-BTM·HCl (2.9 mg, 0.01 mmol) and 4 Å molecular sieves (100 mg) in anhydrous THF (2.0 mL) for 3 days then anhydrous MeOH (0.5 mL) and DMAP (4.9 mg, 0.04 mmol) for 4 hours gave the crude material that was purified by column chromatography (60:1

Petrol : EtOAc) to give the desired compound as a yellow solid (43.6 mg, 54%); **mp** 68–69 °C; $[\alpha]_D^{20}$ +0.8 (*c* 1.0, $CHCl_3$); **Chiral HPLC analysis**: Chiralcel OD-H (95:5 hexane:IPA, flow rate 1 $mLmin^{-1}$, 211 nm, 30 °C) t_R (*S*): 12.1 min, t_R (*R*): 15.9 min, 64:36 er; **IR** ν_{max} (film) 1736 (C=O), 1520 (N-O); **^{1}H NMR (400 MHz, $CDCl_3$)** δ_H: 2.94 (1H, dd, *J* 13.1, 8.0, C(3)$H^A$$H^B$), 3.17 (1H, dd, *J* 13.1, 7.6, C(3)$H^A$$H^B$), 3.55 (2H, d, *J* 13.5, NC$H^A$$H^B$Ph), 3.67 (3H, s, OC$H_3$), 3.67 (2H, d, *J* 13.4, NC$H^A$$H^B$Ph), 3.92 (1H, t, *J* 7.8, C(2)*H*), 7.14 – 7.22 (4H, m, ArC*H*), 7.24 – 7.32 (8H, m, ArC*H*), 8.07 – 8.16 (2H, m, ArC*H*); **^{13}C NMR (101 MHz, $CDCl_3$)** δ_C: 50.5 (*C*(2)H), 52.3 (OCH_3), 56.9 (*C*(3)H_2), 58.8 (NCH_2Ph), 123.5 (C(2)HAr*C*(3,5)H), 127.2 (NCH_2Ar*C*(4)H), 128.3 (NCH_2Ar*C*(3,5)H), 128.9 (NCH_2Ar*C*(2,6)H), 129.3 (C(2)HAr*C*(2,6)H), 138.7 (NCH_2Ar*C*(1)), 144.8 (C(2)HAr*C*(1)), 147.2 (Ar*C*(4)-NO_2), 172.3 (*C*(1)); **HRMS (ESI$^+$)** $C_{24}H_{25}N_2O_4$ $[M+H]^+$ found 405.1798, requires 405.1809 (–2.7 ppm).

Methyl (*S*)-3-(dibenzylamino)-2-(3,4-dimethoxyphenyl)propanoate (18)

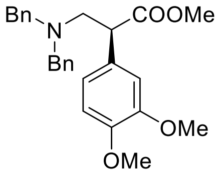

Following **General Procedure 4**, perfluorophenyl 2-(3,4-dimethoxyphenyl)acetate **S18** (69.4 mg, 0.2 mmol), *N*,*N*-dibenzyl-1-methoxymethanamine **5** (72.4 mg, 0.3 mmol), (*R*)-BTM·HCl (2.9 mg, 0.01 mmol) and 4 Å molecular sieves (100 mg) in anhydrous THF (2.0 mL) for 5 days then anhydrous MeOH (0.5 mL) and DMAP (4.9 mg, 0.04 mmol) for 4 hours gave the crude material that was purified by column chromatography (120:1 Petrol : EtOAc) to give the desired compound as a light yellow solid (75.4 mg, 90%); **mp** 50–51 °C; $[\alpha]_D^{20}$ -10.4 (*c* 1.0, $CHCl_3$); **Chiral HPLC analysis**: Chiralpak AD-H (97.5:2.5 hexane:IPA, flow rate 1 $mLmin^{-1}$, 211 nm, 30 °C) t_R (*S*): 18.5 min, t_R (*R*): 26.6 min, 96:4 er; **IR** ν_{max} (film) 1732 (C=O), 1263(C-O); **^{1}H NMR (400 MHz, $CDCl_3$)** δ_H: 2.84 (1H, dd, *J* 12.9, 6.9, C(3)$H^A$$H^B$), 3.19 (1H, dd, *J* 12.9, 8.6, C(3)$H^A$$H^B$), 3.57 – 3.65 (4H, m, N$CH_2$Ph), 3.67 (3H, s, COOC$H_3$), 3.74 – 3.84 (4H, m, ArC(3)-OCH_3 and C(2)*H*), 3.90 (3H, s, ArC(4)-OCH_3), 6.58 – 6.93 (3H, m, ArC*H*), 7.11 – 7.46 (10H, m, ArC*H*); **^{13}C NMR (101 MHz, $CDCl_3$)** δ_C: 50.2 (*C*(2)H), 51.9 (COOCH_3), 55.8 (OCH_3), 55.9 (OCH_3), 57.5 (*C*(2)H_2), 58.6 (NCH_2Ph), 111.0 (C(2)HAr*C*(2)H), 111.1 (C(2)HAr*C*(5)H), 120.4 (C(2)HAr*C*(6)H),

127.0 (NCH$_2$Ar*C*(4)H), 128.1 (NCH$_2$Ar*C*(3,5)H), 128.9 (NCH$_2$Ar*C*(2,6)H), 129.9 (C(2)HAr*C*(1)), 139.2 (NCH$_2$Ar*C*(1)), 148.3 (Ar*C*(4)-OMe), 148.8 (Ar*C*(3)-OMe), 173.8 (*C*(1)); **HRMS (ESI$^+$)** $C_{26}H_{30}NO_4$ [M+H]$^+$ found 420.2160, requires 420.2169 (–2.1 ppm).

Methyl (*S*)-2-(benzo[d][1,3]dioxol-5-yl)-3-(dibenzylamino)propanoate (19)

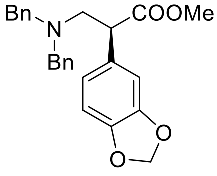

Following **General Procedure 4**, perfluorophenyl 2-(benzo[d][1,3]dioxol-5-yl)acetate **S19** (69.2 mg, 0.2 mmol), *N*,*N*-dibenzyl-1-methoxymethanamine **5** (72.4 mg, 0.3 mmol), (*R*)-BTM·HCl (2.9 mg, 0.01 mmol) and 4 Å molecular sieves (100 mg) in anhydrous THF (2.0 mL) for 4 days then anhydrous MeOH (0.5 mL) and DMAP (4.9 mg, 0.04 mmol) for 4 hours gave the crude material that was purified by column chromatography (80:1 Petrol : EtOAc) to give the desired compound as a colorless oil (71.7 mg, 89%); $[\alpha]_D^{20}$ -11.3 (*c* 1.0, $CHCl_3$); **Chiral HPLC analysis**: Chiralcel OD-H (98.5:1.5 hexane:IPA, flow rate 1.00 mL.min^{-1}, 211 nm, 30 °C): t_R (*S*): 10.9 min, t_R (*R*): 13.4 min, 96:4 er; **IR** ν_{max} (film) 1734 (C=O), 1248 (C-O); **^{1}H NMR (400 MHz, CDCl$_3$)** δ_H: 2.81 (1H, dd, *J* 12.9, 6.4, C(3)H^AH^B), 3.25 (1H, dd, *J* 12.9, 9.2, C(3)H^AH^B), 3.63 (2H, d, *J* 13.6, NCH^AH^BPh), 3.70 (3H, s, OC*H*$_3$), 3.71 (1H, d, *J* 13.4, NCH^AH^BPh), 3.82 (1H, dd, *J* 9.2, 6.3, C(2)*H*), 5.96 (1H, d, *J* 1.4, OCH^AH^BO), 5.97 (1H, d, *J* 1.4, OCH^AH^BO), 6.63 – 6.89 (3H, m, C(2)HArC(2,5,6)*H*), 7.22 – 7.49 (10H, m, ArC*H*); **^{13}C NMR (126 MHz, CDCl$_3$)** δ_C: 50.5 (*C*(2)H), 52.0 (OCH$_3$), 57.6 (*C*(3)H$_2$), 58.6 (NCH$_2$Ph), 101.1 (OCH$_2$O), 108.2 (C(2)HAr*C*(2)H), 108.5 (C(2)HAr*C*(5)H), 121.7 (C(2)HAr*C*(6)H), 127.1 (NCH$_2$Ar*C*(4)H), 128.2 (NCH$_2$Ar*C*(3,5)H), 129.0 (NCH$_2$Ar*C*(2,6)H), 131.1 (C(2)HAr*C*(1)), 139.2 (NCH$_2$Ar*C*(1)), 146.9(C(2)HAr*C*(4)), 147.8 (C(2)HAr*C*(3)), 173.6 (*C*(1)); **HRMS (ESI$^+$)** $C_{25}H_{26}NO_4$ [M+H]$^+$ found 404.1843, requires 404.1856 (–3.2 ppm).

Methyl (*S*)-3-(dibenzylamino)-2-(3-methoxyphenyl)propanoate (20)

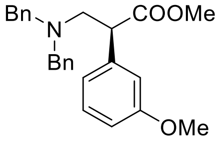

Following **General Procedure 4**, perfluorophenyl 2-(3-methoxyphenyl) acetate **S20**

(66.4 mg, 0.2 mmol), *N*,*N*-dibenzyl-1-methoxymethanamine **5** (72.4 mg, 0.3 mmol), (*R*)-BTM·HCl (2.9 mg, 0.01 mmol) and 4 Å molecular sieves (100 mg) in anhydrous THF (2.0 mL) for 44 hours then anhydrous MeOH (0.5 mL) and DMAP (4.9 mg, 0.04 mmol) for 4 hours gave the crude material that was purified by column chromatography (120:1 Petrol : EtOAc) to give the desired compound as a colorless oil (68.5 mg, 88%); $[\alpha]_D^{20}$ +9.7 (*c* 1.0, $CHCl_3$); **Chiral HPLC analysis**: Chiralcel OD-H (97:3 hexane:IPA, flow rate 1 $mLmin^{-1}$, 211 nm, 30 °C) t_R (*S*): 6.9 min, t_R (*R*): 8.5 min, 95:5 er; **IR** ν_{max} (film) 1734 (C=O), 1261 (C-O); **^{1}H NMR (400 MHz, $CDCl_3$)** δ_H: 2.85 (1H, dd, *J* 12.9, 6.3, C(3)H^AH^B), 3.28 (1H, dd, *J* 12.9, 9.1, C(3)H^AH^B), 3.63 (2H, d, *J* 13.6, NCH^AH^BPh), 3.69 (3H, s), 3.69 (2H, d, *J* 13.6, NCH^AH^BPh), 3.79 (3H, s, Ar-OCH_3), 3.87 (1H, dd, *J* 9.1, 6.3, C(2)*H*), 6.75 – 6.80 (1H, m, ArC*H*), 6.81 – 6.90 (2H, m, ArC*H*), 7.20 – 7.39 (11H, m, ArC*H*); **^{13}C NMR (101 MHz, $CDCl_3$)** δ_C: 50.9 (*C*(2)H), 52.0 ($COOCH_3$), 55.2 (Ar-OCH_3), 57.5 (*C*(3)H^AH^B), 58.6 (NCH_2Ph), 113.0 (C(2)HAr*C*(4)H), 113.7 (C(2)HAr*C*(2)H), 120.7 (C(2)HAr*C*(6)H), 127.0 (NCH_2Ar*C*(4)H), 128.2 (NCH_2Ar*C*(3,5)H), 129.0 (NCH_2Ar*C*(2,6)H), 129.5 (C(2)HAr*C*(5)H), 138.9 (C(2)HAr*C*(1)), 139.2 (NCH_2Ar*C*(1)), 159.7 (Ar*C*(3)-OCH_3), 173.4 (C(1)); **HRMS (ESI$^+$)** $C_{25}H_{28}NO_3$ $[M+H]^+$ found 390.2050, requires 390.2064 (–3.3 ppm).

Methyl (*S*)-2-(3-bromophenyl)-3-(dibenzylamino)propanoate (21)

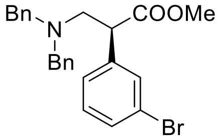

Following **General Procedure 4**, perfluorophenyl 2-(3-bromophenyl)acetate **S21** (76.2 mg, 0.2 mmol), *N*,*N*-dibenzyl-1-methoxymethanamine **5** (72.4 mg, 0.3 mmol), (*R*)-BTM·HCl (2.9 mg, 0.01 mmol) and 4 Å molecular sieves (100 mg) in anhydrous THF (2.0 mL) for 24 hours then anhydrous MeOH (0.5 mL) and DMAP (4.9 mg, 0.04 mmol) for 4 hours gave the crude material that was purified by column chromatography (120:1 Petrol : EtOAc) to give the desired compound as a colorless oil (78.7 mg, 90%); $[\alpha]_D^{20}$ +6.1 (*c* 1.0, $CHCl_3$); **Chiral HPLC analysis**: Chiralcel OD-H (98.5:1.5 hexane:IPA, flow rate 1 $mLmin^{-1}$, 211 nm, 30 °C) t_R (*S*): 7.2 min, t_R (*R*): 8.8 min, 96:4 er; **IR** ν_{max} (film) 1736 (C=O); **^{1}H NMR (500 MHz, $CDCl_3$)** δ_H: 2.88 (1H, dd, *J* 13.0, 7.1, C(3)H^AH^B), 3.22 (1H, dd, *J* 13.0, 8.5, C(3)H^AH^B), 3.63 (2H, d, *J* 13.6, NCH^AH^BPh), 3.67 (2H, d, *J* 13.8,

NCH^A^*H*^*B*Ph), 3.69 (3H, s, OC*H*$_3$), 3.84 (1H, dd, *J* 8.2, 7.4, C(2)*H*), 7.12 – 7.22 (2H, m, ArC*H*), 7.24 – 7.40 (10H, m, ArC*H*), 7.44 – 7.47 (1H, m, ArC*H*); **^{13}C NMR (126 MHz, CDCl$_3$)** δ_C: 50.4 (*C*(2)H), 52.1 (O*C*H$_3$), 57.2 (*C*(3)H$_2$), 58.7 (N*C*H$_2$Ph), 122.5 (Ar*C*-Br), 127.06 (C(2)HAr*C*(6)H), 127.11 (NCH$_2$Ar*C*(4)H), 128.3 (NCH$_2$Ar*C*(3,5)H), 128.9 (NCH$_2$Ar*C*(2,6)H), 130.0 (C(2)HAr*C*(5)H), 130.5 (C(2)HAr*C*(4)H), 131.4 (C(2)HAr*C*(2)H), 139.0 (NCH$_2$Ar*C*(1)), 139.6 (C(2)HAr*C*(1)), 172.9 (*C*(1)); **HRMS (ESI$^+$)** $C_{24}H_{25}BrNO_2$ [M+H]$^+$ found 438.1054, requires 438.1063 (–2.1 ppm).

Methyl (*S*)-3-(dibenzylamino)-2-(2-methoxyphenyl)propanoate (22)

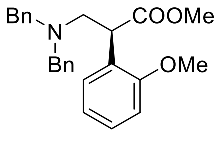

Following **General Procedure 4**, perfluorophenyl 2-(2-methoxyphenyl) acetate **S22** (66.4 mg, 0.2 mmol), *N*,*N*-dibenzyl-1-methoxymethanamine **5** (72.4 mg, 0.3 mmol), (*R*)-BTM·HCl (2.9 mg, 0.01 mmol) and 4 Å molecular sieves (100 mg) in anhydrous THF (2.0 mL) for 4 days then anhydrous MeOH (0.5 mL) and DMAP (4.9 mg, 0.04 mmol) for 4 hours gave the crude material that was purified by column chromatography (120:1 Petrol : EtOAc) to give the desired compound as a colorless oil (63.8 mg, 82%); $[\alpha]_D^{20}$ +11.5 (*c* 1.0, CHCl$_3$); **Chiral HPLC analysis**: Chiralcel OD-H (95:5 hexane:IPA, flow rate 1 mLmin^{-1}, 211 nm, 30 °C) t_R (*S*): 28.9 min, t_R (*R*): 30.6 min, 94:6 er; **IR** ν_{max} (film) 1734 (C=O), 1246 (C-O); **^{1}H NMR (500 MHz, CDCl$_3$)** δ_H: 2.84 (1H, dd, *J* 12.8, 7.0, C(3)H^AH^B), 3.16 (1H, dd, *J* 12.8, 8.3, C(3)H^AH^B), 3.62 (2H, d, *J* 13.8, NCH^AH^{B}Ph), 3.65 (2H, d, *J* 13.8, NCH^AH^BPh), 3.67 (3H, s, COOC*H*$_3$), 3.73 (3H, s, Ar-OC*H*$_3$), 4.45 (1H, dd, *J* 8.2, 7.1, C(2)*H*), 6.87 (1H, dd, *J* 8.2, 0.9, C(2)HArC(3)*H*), 6.92 (1H, td, *J* 7.5, 1.1, C(2)HArC(4)*H*), 7.14 – 7.33 (12H, m, ArC*H*); **^{13}C NMR (101 MHz, CDCl$_3$)** δ_C: 42.8 (C(2)*H*), 51.8 (COOCH$_3$), 55.3 (Ar-OCH$_3$), 56.2 (*C*(3)H$_2$), 58.2 (NCH$_2$Ph), 110.4 (C(2)HAr*C*(3)H), 120.5 (C(2)HAr*C*(5)H), 126.2 (C(2)HAr*C*(1)), 126.8 (CH$_2$Ar*C*(4)H), 128.1 (CH$_2$Ar*C*(3,5)H), 128.2 (C(2)HAr*C*(4)H), 128.87 (C(2)HAr*C*(6)H), 128.9 (CH$_2$Ar*C*(2,6)H), 139.4 (CH$_2$Ar*C*(1)), 156.8 (Ar*C*(2)-OCH$_3$), 173.9 (*C*(1)); **HRMS (ESI$^+$)** $C_{25}H_{28}NO_3$ [M+H]$^+$ found 390.2050, requires 390.2064 (–3.3 ppm).

Methyl (*S*)-2-(2-bromophenyl)-3-(dibenzylamino)propanoate (23)

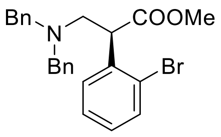

Following **General Procedure 4**, perfluorophenyl 2-(2-bromophenyl)acetate **S23** (76.2 mg, 0.2 mmol), *N*,*N*-dibenzyl-1-methoxymethanamine **5** (72.4 mg, 0.3 mmol), (*R*)-BTM·HCl (2.9 mg, 0.01 mmol) and 4 Å molecular sieves (100 mg) in anhydrous THF (2.0 mL) for 24 hours then anhydrous MeOH (0.5 mL) and DMAP (4.9 mg, 0.04 mmol) for 4 hours gave the crude material that was purified by column chromatography (120:1 Petrol : EtOAc) to give the desired compound as a colorless oil (82.1 mg, 94%); $[\alpha]_D^{20}$ +2.1 (*c* 1.0, $CHCl_3$); **Chiral HPLC analysis**: Chiralcel OD-H (97:3 hexane:IPA, flow rate 1 $mLmin^{-1}$, 211 nm, 30 °C) t_R (*S*): 5.4 min, t_R (*R*): 6.0 min, 95:5 er; **IR** ν_{max} (film) 1736 (C=O); **^{1}H NMR (400 MHz, $CDCl_3$)** δ_H: 2.88 (1H, dd, *J* 12.9, 6.7, C(3)H^AH^B), 3.20 (1H, dd, *J* 12.9, 8.6, C(3)H^AH^B), 3.61 – 3.69 (4H, m, NCH_2Ph), 3.71 (3H, s, OCH_3), 4.60 (1H, dd, *J* 8.6, 6.8, C(2)*H*), 7.11 – 7.18 (1H, m, ArC*H*), 7.20 – 7.37 (12H, m, ArC*H*), 7.56 – 7.63 (1H, m, ArC*H*); **^{13}C NMR (101 MHz, $CDCl_3$)** δ_C: 48.8 (*C*(2)H), 52.1 (OCH_3), 56.8 (*C*(3)H_2), 58.3 (NCH_2Ph), 124.9 (Ar*C*(2)-Br), 127.0 (NCH_2Ar*C*(4)H), 127.6 (C(2)HAr*C*(5)H), 128.2 (NCH_2Ar*C*(3,5)H), 128.7 (C(2)HAr*C*(4)H), 129.0 (NCH_2Ar*C*(2,6)H), 129.4 (C(2)HAr*C*(6)H), 132.9 (C(2)HAr*C*(3)H), 136.8 (C(2)HAr*C*(1)), 139.0 (NCH_2Ar*C*(1)), 173.0 (*C*(1)); **HRMS (ESI$^+$)** $C_{24}H_{25}BrNO_2$ $[M+H]^+$ found 438.1055, requires 438.1063 (–1.8 ppm).

Methyl (*S*)-3-(dibenzylamino)-2-(naphthalen-1-yl)propanoate (24)

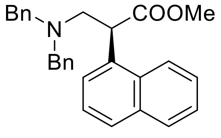

Following **General Procedure 4**, perfluorophenyl 2-(naphthalen-1-yl)acetate **S24** (70.4 mg, 0.2 mmol), *N*,*N*-dibenzyl-1-methoxymethanamine **5** (72.4 mg, 0.3 mmol), (*R*)-BTM·HCl (2.9 mg, 0.01 mmol) and 4 Å molecular sieves (100 mg) in anhydrous THF (2.0 mL) for 3 days then anhydrous MeOH (0.5 mL) and DMAP (4.9 mg, 0.04 mmol) for 4 hours gave the crude material that was purified by column chromatography (80:1 Petrol : EtOAc) to give the desired compound as a colorless oil (67.1 mg, 82%); $[\alpha]_D^{20}$ +28.1 (*c* 1.0, $CHCl_3$); **Chiral HPLC analysis**: Chiralpak IC (99:1 hexane:IPA, flow rate 1

mLmin^{-1}, 211 nm, 30 °C) t_R (*S*): 7.9 min, t_R (*R*): 10.4 min, 94:6 er; **IR** ν_{max} (film) 1732 (C=O); **^{1}H NMR (500 MHz, CDCl$_3$)** δ_H: 2.97 (1H, dd, *J* 13.1, 5.2, C(3)H^AH^B), 3.53 (1H, dd, *J* 13.1, 9.8, C(3)H^AH^B), 3.70 (3H, s, OCH_3), 3.74 (4H, s, NCH_2Ph), 4.69 (1H, dd, *J* 9.8, 5.2, C(2)*H*), 7.26 – 7.38 (10H, m, ArC*H*), 7.40 – 7.54 (4H, m, ArC*H*), 7.75 – 7.85 (2H, m, ArC*H*), 7.86 – 7.91 (1H, m, ArC*H*); **^{13}C NMR (126 MHz, CDCl$_3$)** δ_C: 46.3 (*C*(2)H), 52.1 (OCH$_3$), 57.7 (*C*(3)H$_2$), 59.2 (NCH$_2$Ph), 123.1 (C(2)HArCH), 125.1 (C(2)HArCH), 125.5 (C(2)HArCH), 125.6 (C(2)HArCH), 126.4 (C(2)HArCH), 127.0 (NCH$_2$Ar*C*(4)H), 127.9 (C(2)HArCH), 128.3 (NCH$_2$Ar*C*(3,5)H), 128.9 (C(2)HArCH), 129.0 (NCH$_2$Ar*C*(2,6)H), 131.6 (C(2)HAr*C*(8a)), 133.4 (C(2)HAr*C*(4a)), 133.9 (C(2)HAr*C*(1)), 139.4 (NCH$_2$Ar*C*(1)), 174.0 (*C*(1)); **HRMS (ESI$^+$)** $C_{28}H_{28}NO_2$ [M+H]$^+$ found 410.2100, requires 410.2115 (–3.6 ppm).

Methyl (*S*)-3-(dibenzylamino)-2-(naphthalen-2-yl)propanoate (25)

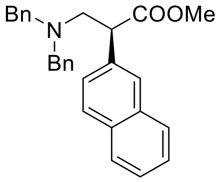

Following **General Procedure 4**, perfluorophenyl 2-(naphthalen-2-yl)acetate **42** (70.4 mg, 0.2 mmol), *N*,*N*-dibenzyl-1-methoxymethanamine **5** (72.4 mg, 0.3 mmol), (*R*)-BTM·HCl (2.9 mg, 0.01 mmol) and 4 Å molecular sieves (100 mg) in anhydrous THF (2.0 mL) for 17 hours then anhydrous MeOH (0.5 mL) and DMAP (4.9 mg, 0.04 mmol) for 4 hours gave the crude material that was purified by column chromatography (80:1 Petrol : EtOAc) to give the desired compound as a colorless oil (76.1 mg, 93%); $[\alpha]_D^{20}$ +12.9 (*c* 1.0, CHCl$_3$); **Chiral HPLC analysis**: Chiralcel OD-H (98.5:1.5 hexane:IPA, flow rate 1 mLmin^{-1}, 211 nm, 30 °C) t_R (*S*): 9.2 min, t_R (*R*): 11.7 min, 96:4 er; **IR** ν_{max} (film) 1732 (C=O); **^{1}H NMR (500 MHz, CDCl$_3$)** δ_H: 3.00 (1H, dd, *J* 13.0, 6.7, C(3)H^AH^B), 3.39 (1H, dd, *J* 13.0, 8.8, C(3)H^AH^B), 3.61 – 3.79 (7H, m, OCH_3 and NCH_2Ph), 4.10 (1H, dd, *J* 8.7, 6.8, C(2)*H*), 7.26 – 7.36 (10H, m, ArC*H*), 7.38 (1H, dd, *J* 8.5, 1.8, ArC*H*), 7.50 – 7.60 (2H, m, ArC*H*), 7.71 (1H, d, *J* 1.1, ArC*H*), 7.83 (2H, t, *J* 5.1, ArC*H*), 7.85 – 7.91 (1H, m, ArC*H*); **^{13}C NMR (126 MHz, CDCl$_3$)** δ_C: 50.9 (*C*(2)H), 52.1 (OCH$_3$), 57.4 (*C*(3)H$_2$), 58.7 (NCH$_2$Ph), 125.9 (C(2)HArCH), 126.1 (C(2)HArCH), 126.4 (C(2)HArCH), 127.0 (NCH$_2$Ar*C*(4)H), 127.1 (C(2)HArCH), 127.7 (C(2)HArCH), 128.0 (C(2)HArCH), 128.2 (C(2)HArCH),

128.2 (NCH$_2$Ar*C*(3,5)H), 129.0 (NCH$_2$Ar*C*(2,6)H), 132.8 (C(2)HAr*C*(4a)), 133.4 (C(2)HAr*C*(8a)), 134.9 (C(2)HAr*C*(2)), 139.2 (NCH$_2$Ar*C*(1)), 173.6 (*C*(1)); **HRMS (ESI$^+$)** $C_{28}H_{28}NO_2$ [M+H]$^+$ found 410.2103, requires 410.2115 (–2.9 ppm).

Methyl (*S*)-3-(dibenzylamino)-2-(thiophen-2-yl)propanoate (26)

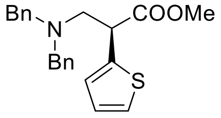

Following **General Procedure 4**, perfluorophenyl 2-(thiophen-2-yl)acetate **S26** (61.6 mg, 0.2 mmol), *N,N*-dibenzyl-1-methoxymethanamine **5** (72.4 mg, 0.3 mmol), (*R*)-BTM·HCl (2.9 mg, 0.01 mmol) and 4 Å molecular sieves (100 mg) in anhydrous THF (2.0 mL) for 5 hours then anhydrous MeOH (0.5 mL) and DMAP (4.9 mg, 0.04 mmol) for 4 hours gave the crude material that was purified by column chromatography (100:1 Petrol : EtOAc) to give the desired compound as a yellow oil (68.6mg, 94%); $[\alpha]_D^{20}$ +8.7 (*c* 1.0, $CHCl_3$); **Chiral HPLC analysis**: Chiralcel OD-H (98.5:1.5 hexane:IPA, flow rate 1 mLmin^{-1}, 211 nm, 30 °C) t_R (*S*): 7.7 min, t_R (*R*): 8.7 min, 94:6 er; **IR** ν_{max} (film) 1734 (C=O); **^{1}H NMR (400 MHz, CDCl$_3$)** δ_H: 2.84 (1H, dd, *J* 12.8, 5.8, C(3)*H*^{A}H^B), 3.29 (1H, dd, *J* 12.8, 9.9, C(3)H^{A}H^B), 3.55 (2H, d, *J* 13.6, NC*H*^{A}H^{B}Ph), 3.69 (3H, s, OC*H*$_3$), 3.74 (2H, d, *J* 13.6, NCH^A*H*^{B}Ph), 4.15 (1H, dd, *J* 9.8, 5.7, C(2)*H*), 6.83 – 6.91 (1H, m, C(2)HArC(3)*H*), 6.91 – 6.98 (1H, m, C(2)HArC(4)*H*), 7.17 – 7.23 (1H, m, C(2)HArC(5)*H*), 7.23 – 7.39 (10H, m, NCH$_2$ArC*H*); **^{13}C NMR (101 MHz, CDCl$_3$)** δ_C: 46.3 (*C*(2)H), 52.1 (OCH$_3$), 58.4 (NCH$_2$Ph), 58.6 (*C*(3)H$_2$), 124.6 (C(2)HAr*C*(5)H), 125.6 (C(2)HAr*C*(3)H), 126.6 (C(2)HArC(4)H), 127.0 (NCH$_2$Ar*C*(4)H), 128.2 (NCH$_2$Ar*C*(3,5)H), 129.0 (NCH$_2$Ar*C*(2,6)H), 138.9 (NCH$_2$Ar*C*(1)), 139.2 (C(2)HAr*C*(2)), 172.7 (*C*(1)); **HRMS (ESI$^+$)** $C_{22}H_{24}NO_2S$ [M+H]$^+$ found 366.1511, requires 366.1522 (–3.0 ppm).

Methyl (S,E)-2-((dibenzylamino)methyl)pent-3-enoate (27)

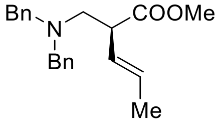

Following **General Procedure 4**, perfluorophenyl (E)-pent-3-enoate **S27** (53.2 mg, 0.2 mmol), *N,N*-dibenzyl-1-methoxymethanamine **5** (72.4 mg, 0.3 mmol), (*R*)-BTM·HCl (2.9 mg, 0.01 mmol) and 4 Å molecular sieves (100 mg) in anhydrous THF (2.0 mL) for

24 hours then anhydrous MeOH (0.5 mL) and DMAP (4.9 mg, 0.04 mmol) for 4 hours gave the crude material that was purified by column chromatography (150:1 Petrol : EtOAc) to give the desired compound as a colorless oil (32.9 mg, 51%); $[\alpha]_D^{20}$ +21.4 (*c* 1.8, $CHCl_3$); **Chiral HPLC analysis**: Chiralcel OJ-H (97:3 hexane:IPA, flow rate 1 $mLmin^{-1}$, 220 nm, 30 °C) t_R (*S*): 11.5 min, t_R (*R*): 15.9 min, 94:6 er; **IR** ν_{max} (film) 1736 (C=O); **^{1}H NMR (500 MHz, $CDCl_3$)** δ_H: 1.69 (3H, dd, *J* 6.4, 1.5, C(5)H_3), 2.51 (1H, dd, *J* 12.7, 6.1, C(2)HC$H^A$$H^B$), 2.93 (1H, dd, *J* 12.7, 9.6, C(2)HC$H^A$$H^B$), 3.37 (1H, td, *J* 9.1, 6.2, C(2)*H*), 3.45 (2H, d, *J* 13.6, NC$H^A$$H^B$Ph), 3.66 (3H, s, OC$H_3$), 3.73 (2H, d, *J* 13.6, NC$H^A$$H^B$Ph), 5.26 – 5.42 (1H, m, C(4)*H*), 5.51 – 5.67 (1H, m, C(3)*H*), 7.23 – 7.30 (2H, m, ArC(4)*H*), 7.30 – 7.38 (8H, m, ArC*H*); **^{13}C NMR (126 MHz, $CDCl_3$)** δ_C: 18.0 (*C*(5)H_3), 48.8 (*C*(2)H), 51.6 (OCH_3), 56.4 (C(2)HCH_2), 58.3 (NCH_2Ph), 126.9 (NCH_2Ar*C*(4)H), 127.1 (*C*(3)H), 128.1 (NCH_2ArC(3,5)H), 128.7 (*C*(4)H), 129.0 (NCH_2ArC(2,6)H), 139.2 (NCH_2Ar*C*(1)), 173.9 (*C*(1)); **HRMS (ESI$^+$)** $C_{21}H_{26}NO_2$ $[M+H]^+$ found 324.1946, requires 324.1958 (–3.7 ppm).

Methyl (*S*)-3-(benzyl(4-fluorobenzyl)amino)-2-phenylpropanoate (28)

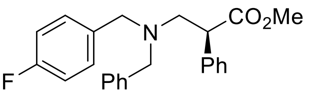

Following **General Procedure 4**, perfluorophenyl 2-phenylacetate **8** (60.4 mg, 0.2 mmol), *N*-benzyl-*N*-(4-fluorobenzyl)-1-methoxymethanamine **S28** (77.7 mg, 0.3 mmol), (*R*)-BTM·HCl (2.9 mg, 0.01 mmol) and 4 Å molecular sieves (100 mg) in anhydrous THF (2.0 mL) for 24 hours then anhydrous MeOH (0.5 mL) and DMAP (4.9 mg, 0.04 mmol) for 4 hours gave the crude material that was purified by column chromatography (100:1 Petrol : EtOAc) to give the desired compound as a colorless oil (58.1 mg, 77%); $[\alpha]_D^{20}$ +8.4 (*c* 1.2, $CHCl_3$); **Chiral HPLC analysis**: Chiralcel OD-H (98.5:1.5 hexane:IPA, flow rate 1 $mLmin^{-1}$, 211 nm, 30 °C) t_R (*S*): 6.7 min, t_R (*R*): 8.0 min, 95:5 er; **IR** ν_{max} (film) 1732 (C=O), 1219 (C-O);**^{1}H NMR (400 MHz, $CDCl_3$)** δ_H: 2.83 (1H, dd, *J* 12.9, 6.5, C(3)$H^A$$H^B$), 3.27 (1H, dd, *J* 12.9, 9.1, C(3)$H^A$$H^B$), 3.56 (1H, d, *J* 13.6, NC$H^A$$H^B$Ph), 3.61 (1H, d, *J* 10.8, NC$H^A$$H^B$$Ar_F$), 3.64 (1H, d, *J* 10.7, NC$H^A$$H^B$$Ar_F$), 3.68 (3H, s, OC$H_3$), 3.69 (1H, d, *J* 13.6, N$H^A$$H^B$Ph), 3.89 (1H, dd, *J* 9.1, 6.5, C(2)*H*), 6.94 – 7.06 (2H, m, N$CH_2$$Ar_F$C(3,5)*H*), 7.18 – 7.38 (12H, m, ArC*H*); **^{13}C NMR (101 MHz, $CDCl_3$)** δ_C: 50.8 (*C*(2)H), 52.0 (OCH_3), 57.4 (NCH_2Ph), 57.8 (N$CH_2$$Ar_F$), 58.5 (*C*(3)$H_2$), 114.9 (d, *J* 21.2,

NCH$_2$Ar$_F$*C*(3,5)H), 127.1 (NCH$_2$Ph*C*(4)H), 127.4 (C(2)HPh*C*(4)H), 128.3 (NCH$_2$Ph*C*(3,5)H and C(2)HPh*C*(3,5)H), 128.6 (NCH$_2$Ph*C*(2,6)H), 128.9 (C(2)HPh*C*(2,6)H), 130.4 (d, *J* 7.9, NCH$_2$Ar$_F$*C*(2,6)H), 134.8 (d, *J* 3.0, NCH$_2$Ar$_F$*C*(1)), 137.3 (C(2)HPh*C*(1)), 139.0 (NCH$_2$Ph*C*(1)), 162.0 (d, *J* 244.5, NCH$_2$Ar$_F$*C*(4)), 173.5 (*C*(1)); **^{19}F NMR (377 MHz, CDCl$_3$)** δ_F: -116.00; **HRMS (ESI$^+$)** $C_{24}H_{25}NO_2F$ $[M+H]^+$ found 378.1854, requires 378.1864 (–2.6 ppm).

Methyl (*S*)-3-(benzyl(4-bromobenzyl)amino)-2-phenylpropanoate (29)

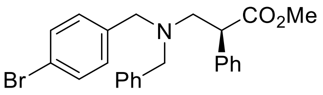

Following **General Procedure 4**, perfluorophenyl 2-phenylacetate **8** (60.4 mg, 0.2 mmol), *N*-benzyl-*N*-(4-bromobenzyl)-1-methoxymethanamine **S29** (95.7 mg, 0.3 mmol), (*R*)-BTM·HCl (2.9 mg, 0.01 mmol) and 4 Å molecular sieves (100 mg) in anhydrous THF (2.0 mL) for 24 hours then anhydrous MeOH (0.5 mL) and DMAP (4.9 mg, 0.04 mmol) for 4 hours gave the crude material that was purified by column chromatography (100:1 Petrol : EtOAc) to give the desired compound as a colorless oil (61.2 mg, 70%); $[\alpha]_D^{20}$ +10.4 (*c* 1.3, CHCl$_3$); **Chiral HPLC analysis**: Chiralcel OD-H (98.5:1.5 hexane:IPA, flow rate 1 mLmin^{-1}, 211 nm, 30 °C) t_R (*S*): 7.7 min, t_R (*R*): 9.2 min, 95:5 er; **IR** ν_{max} (film) 1732 (C=O); **^{1}H NMR (500 MHz, CDCl$_3$)** δ_H: 2.83 (1H, dd, *J* 12.9, 6.5, C(3)H^AH^B), 3.25 (1H, dd, *J* 12.6, 9.3, C(3)H^AH^B), 3.53 (1H, d, *J* 13.8, NCH^AH^BPh), 3.59 (1H, s, NCH^AH^BAr$_{Br}$), 3.61 (1H, s, NCH^AH^BAr$_{Br}$), 3.63 – 3.70 (4H, m, OCH_3 and NCH^AH^BPh), 3.87 (1H, t, *J* 7.7, C(2)*H*), 7.11 (2H, d, *J* 7.8, NCH$_2$Ar$_{Br}$C(2,6)*H*), 7.18 – 7.37 (10H, m, NCH$_2$PhC*H* and C(2)HPhC*H*), 7.42 (2H, d, *J* 7.7, NCH$_2$Ar$_{Br}$C(3,5)*H*); **^{13}C NMR (126 MHz, CDCl$_3$)** δ_C: 50.7 (*C*(2)H), 52.0 (OCH$_3$), 57.4 (*C*(3)H$_2$), 57.9 (NCH$_2$Ar), 58.5 (NCH$_2$Ar), 120.7 (Ar*C*(4)-Br), 127.1 (C(2)HPh*C*(4)H), 127.4 (NCH$_2$Ph*C*(4)H), 128.23 (NCH$_2$Ph*C*(2,6)H), 128.25 (NCH$_2$Ph*C*(3,5)H), 128.6 (C(2)HPh*C*(3,5)H), 128.9 (C(2)HPh*C*(2,6)H), 130.6 (NCH$_2$Ar$_{Br}$*C*(2,6)H), 131.2 (NCH$_2$Ar$_{Br}$*C*(3,5)H), 137.2 (C(2)HPh*C*(1)), 138.3 (NCH$_2$Ar$_{Br}$*C*(1)), 138.8 (NCH$_2$Ph*C*(1)), 173.4 (*C*(1)); **HRMS (ESI$^+$)** $C_{24}H_{25}NO_2Br$ $[M+H]^+$ found 438.1048, requires 438.1063 (–3.5 ppm).

Methyl (*S*)-3-(benzyl(4-methoxybenzyl)amino)-2-phenylpropanoate (30)

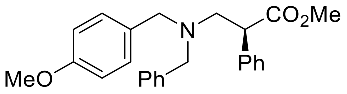

Following **General Procedure 4**, perfluorophenyl 2-phenylacetate **8** (60.4 mg, 0.2 mmol), *N*-benzyl-1-methoxy-*N*-(4-methoxybenzyl)methanamine **S30** (95.7 mg, 0.3 mmol), (*R*)-BTM·HCl (2.9 mg, 0.01 mmol) and 4 Å molecular sieves (100 mg) in anhydrous THF (2.0 mL) for 20 hours then anhydrous MeOH (0.5 mL) and DMAP (4.9 mg, 0.04 mmol) for 4 hours gave the crude material that was purified by column chromatography (100:1 Petrol : EtOAc) to give the desired compound as a colorless oil (56.0 mg, 72%); $[\alpha]_D^{20}$ +9.2 (*c* 0.7, $CHCl_3$); **Chiral HPLC analysis**: Chiralcel OD-H (98.5:1.5 hexane:IPA, flow rate 1 $mLmin^{-1}$, 211 nm, 30 °C) t_R (*S*): 9.1 min, t_R (*R*): 10.4 min, 95:5 er; **IR** ν_{max} (film) 1734 (C=O); **^{1}H NMR (300 MHz, $CDCl_3$)** δ_H: 2.81 (1H, dd, *J* 12.9, 6.4, C(3)H^AH^B), 3.26 (1H, dd, *J* 12.9, 9.3, C(3)H^AH^B), 3.49 – 3.73 (4H, m, NCH_2Ar), 3.67 (3H, s, $COOCH_3$), 3.83 (3H, s, Ar-OCH_3), 3.88 (1H, dd, *J* 9.2, 6.4, C(2)*H*), 6.81 – 6.94 (2H, m, $NCH_2Ar_{MeO}C(3,5)H$), 7.07 – 7.42 (12H, m, NCH_2ArCH); **^{13}C NMR (101 MHz, $CDCl_3$)** δ_C: 50.8 (*C*(2)H), 51.9 ($COOCH_3$), 55.3 (Ar-OCH_3), 57.3 (NCH_2Ar), 57.8 (NCH_2Ar), 58.3 (*C*(3)H_2), 113.5 ($NCH_2Ar_{MeO}C(3,5)H$), 126.9 (C(2)HPh*C*(4)H), 127.3 ($NCH_2PhC(4)H$), 128.1 ($NCH_2PhC(3,5)H$), 128.3 ($NCH_2PhC(2,6)H$), 128.5 (C(2)HPh*C*(3,5)H), 128.9 (C(2)HPh*C*(2,6)H), 130.1 ($NCH_2Ar_{MeO}C(2,6)H$), 131.1 ($NCH_2Ar_{MeO}C(1)$), 137.4 (C(2)HPh*C*(1)), 139.3 ($NCH_2PhC(1)$), 158.6 ($NCH_2Ar_{MeO}C(4)H$), 173.6 (*C*(1)); **HRMS (ESI$^+$)** $C_{25}H_{28}NO_3$ $[M+H]^+$ found 390.2052, requires 390.2064 (–3.0 ppm).

Ethyl (*S*)-3-(diallylamino)-2-phenylpropanoate (31)

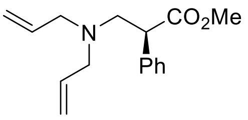

Following **General Procedure 4**, perfluorophenyl 2-phenylacetate **8** (60.4 mg, 0.2 mmol), *N*-allyl-*N*-(methoxymethyl)prop-2-en-1-amine **S31** (42.3 mg, 0.3 mmol), (*R*)-BTM·HCl (2.9 mg, 0.01 mmol) and 4 Å molecular sieves (100 mg) in anhydrous THF (2.0 mL) for 20 hours then anhydrous MeOH (0.5 mL) and DMAP (4.9 mg, 0.04 mmol) for 4 hours gave the crude material that was purified by column chromatography (120:1 Petrol : EtOAc) to give desired compound as a colorless oil (41.4 mg, 80% yield). $[\alpha]_D^{20}$ –13.1 (*c* 0.6, $CHCl_3$); **Chiral HPLC analysis**: Chiralcel OD-H (98.5:1.5 hexane:IPA,

flow rate 0.3 mLmin^{-1}, 211 nm, 30 °C) t_R (*S*): 13.4 min, t_R (*R*): 14.7 min, 94:6 er; **IR** ν_{max} (film) 1736 (C=O), 1643 (C=C); **^{1}H NMR (300 MHz, CDCl$_3$)** δ_H: 2.67 (1H, dd, *J* 13.0, 5.2, C(3)H^AH^B), 3.09 (1H, d, *J* 14.2, NCH_2CH=CH$_2$), 3.11 (2H, d, *J* 14.1, NCH_2CH=CH$_2$), 3.23 (2H, d, *J* 14.1, NCH_2CH=CH$_2$), 3.25 (1H, d, *J* 14.3, NCH_2CH=CH$_2$), 3.28 (2H, dd, *J* 12.9, 10.1, C(3)H^AH^B), 3.71 (3H, s, OCH_3), 3.88 (1H, dd, *J* 10.2, 5.2, C(2)*H*), 5.08 – 5.25 (4H, m, CH_2=CH), 5.84 (2H, ddt, *J* 17.0, 10.2, 6.4, CH$_2$=C*H*CH$_2$), 7.17 – 7.38 (5H, m, ArC*H*); **^{13}C NMR (126 MHz, CDCl$_3$)** δ_C: 50.8 (*C*(2)H), 51.9 (OCH$_3$), 57.0 (*C*(3)H$_2$), 57.3 (CH$_2$=CHCH$_2$), 117.4 (CH$_2$=CH), 127.4 (C(2)HAr*C*(4)H), 128.1 (C(2)HAr*C*(3,5)H), 128.6 (C(2)HAr*C*(2,6)H), 135.7 (CH$_2$=CH), 137.5 (C(2)HAr*C*(1)), 173.8 (*C*(1)); **HRMS (ESI$^+$)** $C_{16}H_{22}NO_2$ [M+H]$^+$ found 260.1636, requires 260.1645 (–3.5 ppm).

N-Benzyl-N-methyl-2-phenylacetamide (37)

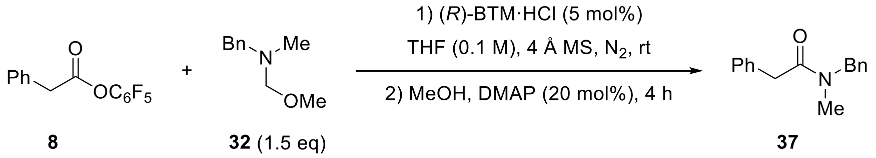

Following **General Procedure 4**, perfluorophenyl 2-phenylacetate **8** (60.4 mg, 0.2 mmol), *N*-benzyl-1-methoxy-*N*-methylmethanamine **32** (0.3 mmol), (*R*)-BTM·HCl (2.9 mg, 0.01 mmol) and 4 Å molecular sieves (100 mg) in anhydrous THF (2.0 mL) for 24 hours. Then the reaction was quenched by adding anhydrous MeOH (0.5 mL) and DMAP (4.9 mg, 0.04 mmol) and the resulted solution was stirred at room temperature for 4 hours. The solvent was removed *in vacuo* and the residue was purified through silica to give *N*-benzyl-*N*-methyl-2-phenylacetamide (containing rotamers, 30% yield) as the major product, without formation of the desire β-amino ester. When only (*R*)-BTM was used as the catalyst, the amide was also formed in 31% yield. **^{1}H NMR (300 MHz, CDCl$_3$)** δ_H: 2.97 (3H), 3.81 (2H), 4.61 (2H), 7.03 – 7.18 (1H, m), 7.22 – 7.52 (9H, m).

2-Phenyl-1-(piperidin-1-yl)ethan-1-one (38)

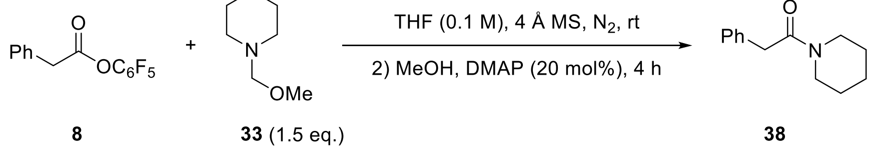

Following **General Procedure 4**, perfluorophenyl 2-phenylacetate **8** (60.4 mg, 0.2 mmol), 1-(methoxymethyl)piperidine **33** (0.3 mmol), (*R*)-BTM·HCl (2.9 mg, 0.01 mmol)

and 4 Å molecular sieves (100 mg) in anhydrous THF (2.0 mL) for 24 hours. Then the reaction was quenched by adding anhydrous MeOH (0.5 mL) and DMAP (4.9 mg, 0.04 mmol) and the resulted solution was stirred at room temperature for 4 hours. The solvent was removed *in vacuo* and the residue was purified through silica to give 2-phenyl-1-(piperidin-1-yl)ethan-1-one **38** (31% yield) as the major product, without formation of the desire β-amino ester. When only (*R*)-BTM was used as the catalyst, the amide was also formed in 39% yield. **^{1}H NMR (400 MHz, $CDCl_3$)** δ_H: 1.24 – 1.43 (2H, m), 1.43 – 1.67 (4H, m), 3.38 (2H, t, *J* 5.6), 3.58 (2H, t, *J* 5.5), 3.74 (2H, s), 7.19 – 7.39 (5H, m).

4.5 X-ray crystal structure

X-ray diffraction data were collected at 173 K using a Rigaku MM-007HF High Brilliance RA generator/confocal optics with XtaLAB P100 diffractometer [Cu Kα radiation (λ = 1.54187 Å)]. Data for (*S*)-**13** were collected using CrystalClear[14] and processed (including correction for Lorentz, polarization and absorption effects) using CrysAlisPro.[15] The structure was solved using intrinsic phasing methods (SHELXT[16]) and refined by full-matrix least-squares against F^2 (SHELXL-2018/3[17]). Non-hydrogen atoms were refined anisotropically, and all hydrogen atoms were refined using a riding model. All calculations were performed using the Oelx2[18]interface.

	(*S*)-**13**
CCDC	1988076
empirical formula	$C_{24}H_{24}BrNO_2$
fw	438.35
crystal description	colourless prism
crystal size [mm]	0.45 × 0.3 × 0.24
space group	$P4_12_12$
a [Å]	8.70727(5)
b [Å]	8.70727(5)
c [Å]	55.7266(6)
vol [Å]^3	4225.00(7)
α [°]	90
β [°]	90
γ [°]	90
Z	8
ρ (calc) [g/cm^3]	1.378
μ [mm^{-1}]	2.794
F(000)	1808.0
reflections collected	44540
independent reflections (R_{int})	3854 (0.0296)
data/parameters	3854/254
GOF on F^2	1.224
R_1 [$I > 2\sigma(I)$]	0.0494
wR_2 (all data)	0.1285
largest diff. peak/hole [e/Å^3]	0.57, −1.25
Flack parameter	0.001(4)

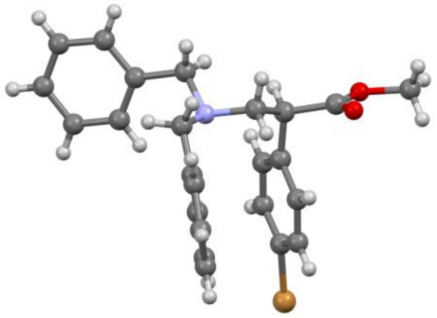

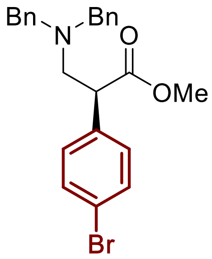

5 Target applications

5.1 Synthesis of (*S*)-Venlafaxine·HCl

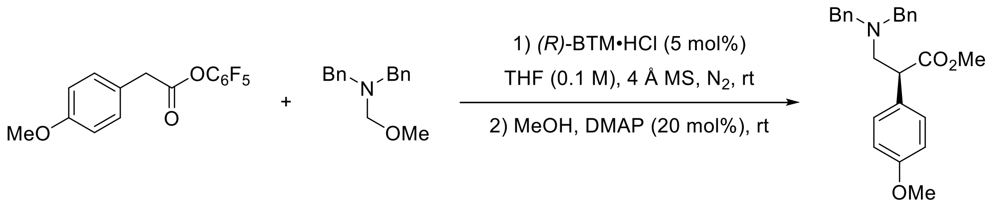

S11 **5** **11**

Following **General Procedure 4**, *N,N*-dibenzyl-1-methoxymethanamine **5** (1.08 g, 4.5 mmol), perfluorophenyl 2-(4-methoxyphenyl)acetate **S11** (996 mg, 3.0 mmol), (*R*)-BTM•HCl (43.2 mg, 0.15 mmol) and 4 Å molecular sieves (1.5 g) in anhydrous THF (30 mL) for 5 days then anhydrous MeOH (8 mL) and DMAP (73 mg, 0.6 mmol) for 4 hours gave the crude material that was purified by column chromatography (100:1 Petrol : EtOAc) to give the desired compound **11** as a colorless oil (1.1 g, 94% yield, 94:6 er).

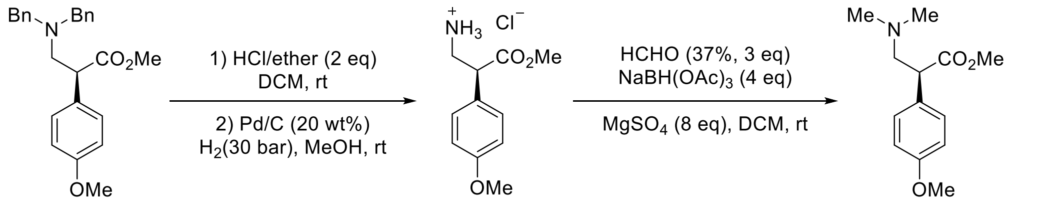

11 **A39** **39**

Methyl (*S*)-3-(dibenzylamino)-2-(4-methoxyphenyl)propanoate **11** (1.1 g, 2.8 mmol, 94:6 er) was dissolved in CH_2Cl_2 and HCl in ether (2 M, 2.8 mL, 2 equiv) was added. The reaction was stirred at rt for 30 min. The solvent was removed and the residue was transferred to the test tube for autoclave, which was then charged with N_2. Pd/C (240 mg, 20 wt%) was carefully added to the test tube under a N_2 atmosphere, followed by anhydrous MeOH (20 mL) (Caution: fire hazard). Then the autoclave was carefully filled with H_2 (30 bar) and the reaction was stirred at rt for 40 h before filtration and evaporation to give **A39** as a white solid (quantitative) which was used for further reactions without purification. (*S*)-3-Methoxy-2-(4-methoxyphenyl)-3-oxopropan-1-aminium chloride **A39** (686 mg, 2.8 mmol) was redissolved in CH_2Cl_2 (20 mL, 0.14 M). $MgSO_4$ (2.7 g, 22.4 mmol) was added, followed by aqueous formaldehyde (37 wt% in H_2O, 680 mg, 8.4 mmol). The resulting mixture was stirred for 10 min, before addition of $NaBH(OAc)_3$ (2.37 g, 11.2 mmol). The reaction was stirred at room temperature for

24 hours before quenching with sat. aq. $NaHCO_3$ (10 mL). The mixture was extracted with CH_2Cl_2 (10 mL × 2). The organic layers were combined and treated with 1 M HCl (10 mL) and then extracted with 1 M HCl (10 mL × 3). The aqueous layers were combined and washed with Et_2O (10 mL × 2) and then basified with solid $NaHCO_3$ to pH > 8. The resulting solution was extracted with EtOAc (30 mL × 2). The EtOAc solutions were combined, dried over $MgSO_4$, filtered and concentrated to dryness *in vacuo* to give product **39** as an off white solid (555 mg, 83% yield, 93:7 er) with spectroscopic data in accordance with literature.[19] $[\alpha]_D^{20}$ –37.1 (*c* 1.0, $CHCl_3$) {Lit.[19] $[\alpha]_D^{20}$ -34.7 (*c* 1.69, $CHCl_3$)}; **mp** 44–45 °C {Lit.[19] 41-43 °C}; **Chiral HPLC analysis**: Chiralcel OD-H (98.5:1.5 hexane:IPA, flow rate 1 mLmin^{-1}, 211 nm, 30 °C) t_R (*S*): 7.1 min, t_R (*R*): 8.6 min, 93:7 er; **^{1}H NMR (400 MHz, $CDCl_3$)** δ_H: 2.29 (6H, s, N(C*H*$_3$)$_2$), 2.44 (1H, dd, *J* 12.3, 5.3, C(3)*H*^{A}H^B), 3.13 (1H, dd, *J* 12.3, 10.1, C(3)H^A*H*^{B}), 3.70 (3H, s, COOC*H*$_3$), 3.78 (1H, dd, *J* 10.1, 5.3, C(2)*H*), 3.81 (3H, s, Ar-OC*H*$_3$), 6.83 – 6.91 (2H, m, C(2)HArC(3,5)*H*), 7.23 – 7.28 (2H, m, C(2)HArC(2,6)*H*); **^{13}C NMR (101 MHz, $CDCl_3$)** δ_C: 45.7 (N(CH_3)$_2$), 49.4 (*C*(2)H), 52.1 ($COOCH_3$), 55.3 (Ar-OCH_3), 63.0 (*C*(3)H_2), 114.1 (C(2)HAr*C*(3,5)H), 128.9 (C(2)HAr*C*(2,6)H), 129.5 (C(2)HAr*C*(1)), 158.9 (Ar*C*(4)-OMe), 174.0 (*C*(1)); **HRMS (ESI$^+$)** $C_{13}H_{20}NO_3$ $[M+H]^+$ found 238.1435, requires 238.1438 (–1.2 ppm).

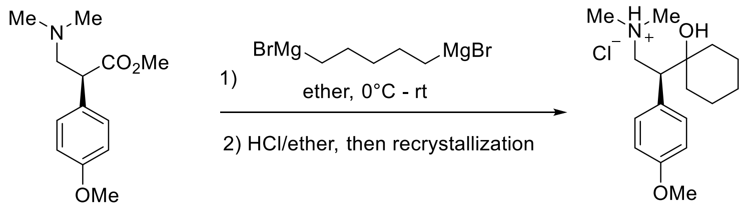

Following a literature procedure,[19] magnesium turnings (1.14 g, 47 mmol) was added to anhydrous Et_2O (60 mL) and 1,5-dibromopentane (4.10 g, 18 mmol) was added. The mixture was heated to reflux for 3 hours until most of the magnesium was consumed. The formed Grignard reagent (5 mL, bottom layer of above reaction mixture) and methyl (*S*)-3-(dimethylamino)-2-(4-methoxyphenyl)propanoate **39** (120 mg, 0.5 mmol, 93:7 er) in Et_2O (5 mL) were added to Et_2O (70 mL) cooled to 0 °C simultaneously by a syringe pump over 0.5 h. The reaction was then allowed to warm to room temperature and stirred at room temperature overnight. 1 M Aqueous HCl (15 mL) was added to quench the reaction. The organic layer was separated and extracted with 1 M HCl (2 ×

15 mL). The combined aqueous layers were then extracted with Et_2O (2 × 15 mL). Sat. aq. $NaHCO_3$ solution was used to adjust pH of the aqueous solution to pH > 8. The aqueous solution was extracted with EtOAc (4 × 50 mL), and the combined organic layers were washed with brine (100 mL), dried over $MgSO_4$, filtered and concentrated to dryness *in vacuo*. The residue was dissolved in Et_2O (4 mL) and HCl in ether (2 M, 2 mL) was added. The salt was filtered and washed with Et_2O to give the title compound **4** as the HCl salt, which was recrystallized from CH_2Cl_2/ether to give **4** as a white solid (94 mg, 60% yield, 98.5:1.5 er) with spectroscopic data in accordance with literature.[19] $[\alpha]_D^{20}$ –4.0 (*c* 0.5, EtOH) {Lit.[19] $[\alpha]_D^{20}$ -4.3 (*c* 1.07, EtOH)}; **Chiral HPLC analysis**: Chiralpak AD-H (98.9:1:0.1 hexane:IPA:TEA, flow rate 0.4 mLmin^{-1}, 274 nm, 30 °C) t_R (*R*): 22.8 min, t_R (*S*): 27.2 min, 1:99 er; **^{1}H NMR (400 MHz, $CDCl_3$)** δ_H: 0.71 – 1.78 (10H, m, (CH_2)$_5$), 2.63 (3H, s, NCH^A_3), 2.81 (3H, s, NCH^B_3), 3.12 – 3.27 (1H, m, C(1)H^AH^B), 3.34 (1H, s, C(2)*H*), 3.68 (1H, s, O*H*), 3.79 (3H, s, OCH_3), 4.05 (1H, d, *J* 10.7, C(1)H^AH^B), 6.85 (2H, d, *J* 8.4, CHArC(3,5)*H*), 7.13 (2H, d, *J* 8.5, CHArC(2,6)*H*), 11.43 (1H, s, N*H*); **^{13}C NMR (101 MHz, $CDCl_3$)** δ 21.2 ((CH_2)$_5$), 21.6 ((CH_2)$_5$), 25.3 ((CH_2)$_5$), 31.4 ((CH_2)$_5$), 36.6 ((CH_2)$_5$), 42.7 (N(CH_3)$_2$), 45.1 (N(CH_3)$_2$), 52.5 (*C*(2)H), 55.3 (OCH_3), 60.3 (C(1)H_2), 73.6 (*C*-OH), 114.1 (Ar*C*(3,5)H), 130.2 (Ar*C*(2,6)H), 131.3 (Ar*C*(1)), 158.9 (Ar*C*(4)); **HRMS (ESI$^+$)** $C_{17}H_{28}NO_2$ [M-Cl]$^+$ found 278.2109, requires 278.2115 (–3.5 ppm).

5.2 Synthesis of (*S*)-Nakinadine B

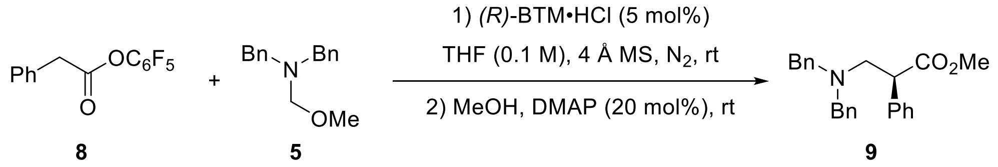

Following **General Procedure 4**, *N,N*-dibenzyl-1-methoxymethanamine **5** (1.45 g, 6.0 mmol), perfluorophenyl 2-phenylacetate **8** (1.21 g, 4.0 mmol), (*R*)-BTM•HCl (57.6 mg, 0.2 mmol) and 4 Å molecular sieves (2.0 g) in anhydrous THF (40 mL) for 64 hours then anhydrous MeOH (10 mL) and DMAP (98 mg, 0.8 mmol) for 4 hours gave the crude material that was purified by column chromatography (100:1 Petrol : EtOAc) to give the desired compound **9** as a colorless oil (1.25 g, 87% yield, 95:5 er).

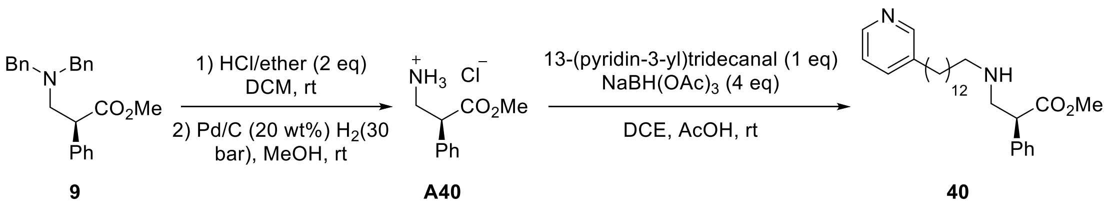

Methyl (*S*)-3-(dibenzylamino)-2-phenylpropanoate **9** (359 mg, 1.0 mmol, 95:5 er) was dissolved in CH_2Cl_2 (5 mL) and HCl in ether (2 M, 1 mL, 2 equiv) was added. The mixture was stirred at rt for 30 min. The solvent was removed and the residue was transferred to the test tube for autoclave, which was then charged with N_2. Pd/C (80 mg, 20 wt%) was carefully added to the test tube under a N_2 atmosphere, followed by MeOH (10 mL) (Caution: fire hazard). Then the autoclave was carefully filled with H_2 (30 bar) and the reaction was stirred at room temperature for 40 h before filtration and evaporation to give **A40** as a pure white solid (quantitative) which was used for further reactions without purification. (*S*)-3-Methoxy-3-oxo-2-phenylpropan-1-aminium chloride **A40** (108 mg, 0.5 mmol) was dissolved in DCE (8 mL), then 13-(pyridin-3-yl)tridecanal[20] (137 mg, 0.5 mmol) and AcOH (0.5 mL) were added. $NaBH(OAc)_3$ (318 mg, 1.5 mmol) was added in portions and the reaction was stirred at rt for 10 h before quenching with sat. aq. $NaHCO_3$ (10 mL). The mixture was extracted with EtOAc (3 × 10 mL), dried over Na_2SO_4, filtered and concentrated to dryness *in vacuo*. The crude material was purified by column chromatography (Petrol to 1:2 Petrol : EtOAc) gave desired product **40** as a white solid (160 mg, 73% yield, 95:5 er). **mp** 35–36 °C; $[\alpha]_D^{20}$ –35.5 (*c* 0.9, $CHCl_3$); **Chiral HPLC analysis**: Chiralcel OD-H (96:3:1 hexane:IPA:TEA, flow rate 1 mLmin^{-1}, 274 nm, 30 °C) t_R (*S*): 30.6 min, t_R (*R*): 37.2 min, 95:5 er; **IR** ν_{max} (film) 2924 (C-H), 1734 (C=O); **^{1}H NMR (400 MHz, $CDCl_3$)** δ_H: 1.19 – 1.39 (18H, m, C(3′)H_2–C(11′)H_2), 1.39 – 1.51 (2H, m, C(2′)H_2), 1.56 – 1.68 (2H, m, C(12′)H_2), 1.74 (1H, s, N*H*), 2.47 – 2.74 (4H, m, C(1′)H_2 and C(13′)H_2), 2.91 (1H, dd, *J* 12.1, 6.6, C(3)H^AH^B), 3.29 (1H, dd, *J* 12.1, 8.6, C(3)H^AH^B), 3.68 (3H, s, OCH_3), 3.85 (1H, dd, *J* 8.5, 6.6, C(2)*H*), 7.21 (1H, ddd, *J* 7.8, 4.8, 0.7, PyC(5)*H*), 7.27 – 7.40 (5H, m, PhC*H*), 7.50 (1H, ddd, *J* 7.8, 2.2, 1.7, PyC(4)*H*), 8.38 – 8.47 (2H, m, PyC(2,6)*H*); **^{13}C NMR (101 MHz, $CDCl_3$)** δ_C: 27.2, 29.2, 29.4, 29.5, 29.6, 29.6, 29.6, 29.9, (*C*(2′)H_2–*C*(11′)H_2), 31.2 (*C*(12′)H_2), 33.0 (*C*(13′)H_2), 49.7 (*C*(1′)H_2), 51.9 (*C*(2)H), 52.1 (OCH_3), 52.7 (*C*(3)H_2), 123.2 (Py*C*(5)H), 127.5 (Ph*C*(4)H), 128.0 (Ph*C*(3,5)H), 128.8 (Ph*C*(2,6)H), 135.8 (Py*C*(4)H), 137.3 (Ph*C*(1)), 138.0

(PyC(3)), 147.2 (PyC(6)H), 150.0 (PyC(2)H), 173.7 (C(1)); **HRMS (ESI+)** $C_{28}H_{43}N_2O_2$ [M+H]+ found 439.3307, requires 439.3319 (–2.7 ppm). **Note**: spontaneous racemisation of **40** occurred upon storing on the bench top for weeks.

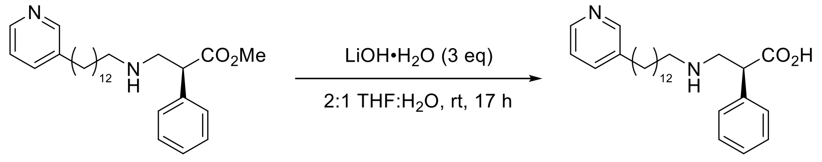

40 **1**

LiOH•H_2O (25.2 mg, 0.6 mmol) was added to the solution of ethyl (*S*)-2-phenyl-3-((13-(pyridin-3-yl)tridecyl)amino)propanoate **40** (88 mg, 0.2 mmol, 95:5 er) in THF/H_2O (2:1, 3 mL) and the resulting mixture was stirred at rt for 17 h before quenching with 1 M aq. HCl (3 mL). The solution was then neutralized by addition of sat. aq. $NaHCO_3$ (10 mL) and extracted with CH_2Cl_2 (3 × 10 mL), dried over Na_2SO_4, filtered and concentrated to dryness *in vacuo*. The crude material was purified by column chromatography (CH_2Cl_2 to 2:1 CH_2Cl_2:MeOH) to give title compound **1** as a white solid (58.4 mg, 69% yield, 93:7 er) with spectroscopic data in accordance with the literature.[21] $[\alpha]_D^{20}$ –14.2 (*c* 0.6, $CHCl_3$) {Lit.[21] $[\alpha]_D^{20}$ -6.3 (*c* 1.0, $CHCl_3$)}; **1H NMR (500 MHz, CDCl3)** δ_H: 0.99 – 1.40 (18H, m, C(3′)H_2–C(11′)H_2), 1.44 – 1.70 (4H, m, C(2′)H_2 and C(12′)H_2), 2.57 – 2.61 (2H, m, C(13′)H_2), 2.75 (3H, s, C(3)H^AH^B and NC(1′)H_2), 3.52 (1H, s, C(3)H^AH^B), 4.08 (1H, d, *J* 10.5, C(2)*H*), 7.12 – 7.25 (4H, m, ArC*H*), 7.30 – 7.37 (2H, m, C(1)HPhC(2,6)*H*), 7.48 (1H, dt, *J* 7.8, 1.9, PyC(4)*H*), 8.37 – 8.49 (2H, m, PyC(2,6)*H*), 9.76 (1H, br s, N*H*); **13C NMR (126 MHz, CDCl3)** δ_C: 25.5 (C(12′)H_2), 26.9, 29.2, 29.4, 29.5, 29.59, 29.63, 29.7, (C(3′)H_2 –C(11′)H_2), 31.2 (C(2′)H_2), 33.0 (C(13′)H_2), 47.8 (C(1′)H_2), 50.9 (C(3)H_2), 51.4 (C(2)H), 123.2 (PyC(5)H), 127.1 (PhC(4)H), 128.2 (PhC(3,5)H), 128.7 (PhC(2,6)H), 135.8 (PyC(4)H), 138.0 (PyC(3)), 138.8 (PhC(1)), 147.1 (PyC(6)H), 149.9 (PyC(2)H), 176.9 (C(1)); **HRMS (ESI+)** $C_{27}H_{41}N_2O_2$ [M+H]+ found 425.3157, requires 425.3163 (–1.3 ppm). To determine the enantiomeric excess of compound **1**, it was converted into its methyl ester **40**, by treating it with thionyl chloride in MeOH. **Chiral HPLC analysis** (determined by converting into **40**): Chiralcel OD-H (96:3:1 hexane:IPA:TEA, flow rate 1 mLmin^{-1}, 274 nm, 30 °C) t_R (*S*): 30.5 min, t_R (*R*): 36.7 min, 93:7 er.

6 Mechanistic Investigation

6.1 Reaction monitoring by ^{19}F NMR

In support of temporal reaction monitoring mentioned in the paper, in the beginning of mechanistic investigation section

^{19}F NMR was adopted to monitor the reaction with fluorinated substrates and catalyst in a NMR tube. A sealed capillary containing C_6D_6 was added to the NMR tube so that the field frequency could be locked to C_6D_6. Fluorobenzene was used as an internal reference for chemical shift and integration. Parameters used for ^{19}F NMR: 80 ppm sweep width (−100 to −180 ppm), number scans (ns) = 2, spectral centre (o1p) = −140 ppm, dummy scans (ds) = 2, d1 relaxation delay = 30 s.

Preparation of fluorinated product

As the β-amino perfluorophenyl ester product was not suitable for isolation, to locate its signal on a ^{19}F NMR spectrum, the ester was synthesized following the **General Procedure 4** using without adding MeOH and DMAP, and characterized in situ.

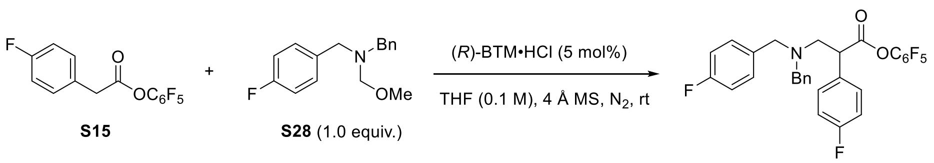

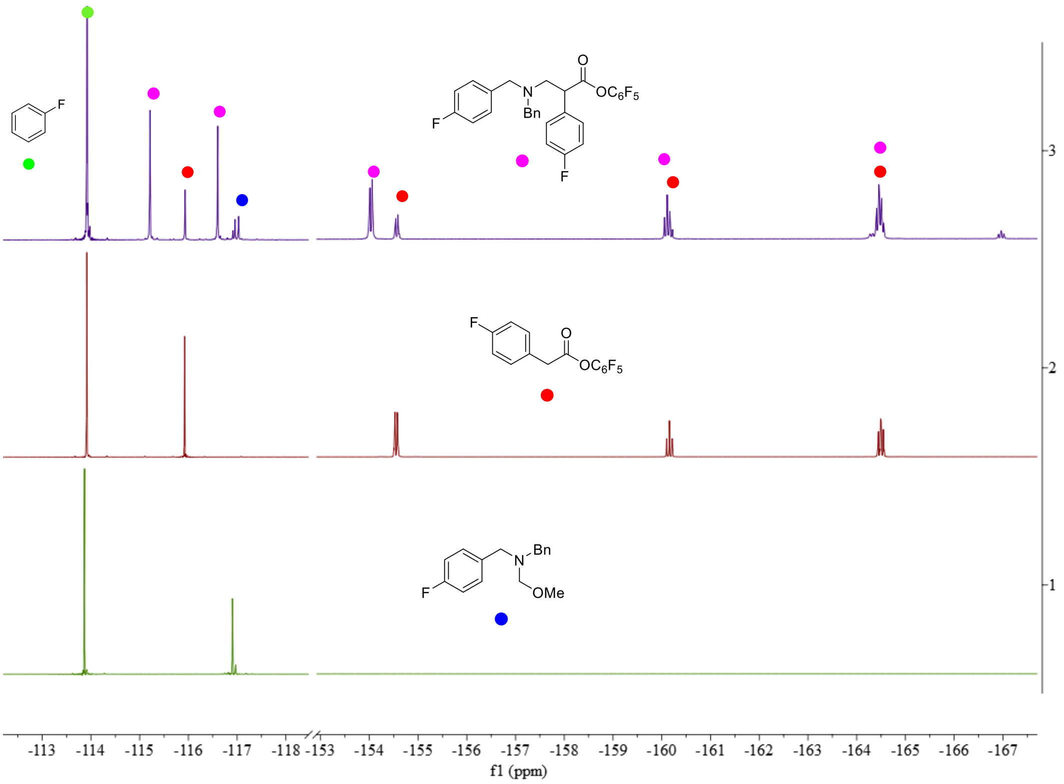

Problems with reaction monitoring

The reaction in THF was found to be heterogeneous, with much precipitation formed thoughout the reaction process. While this solubility problem could be solved by using a THF/DMF (4:1 v/v) mixed solvent, multiple species were observed (including iminium formation and subsequent hydrolysis), together with unknown species generated during the process. Due to the complexity of this process we did not attempt to probe reaction orders using this method.

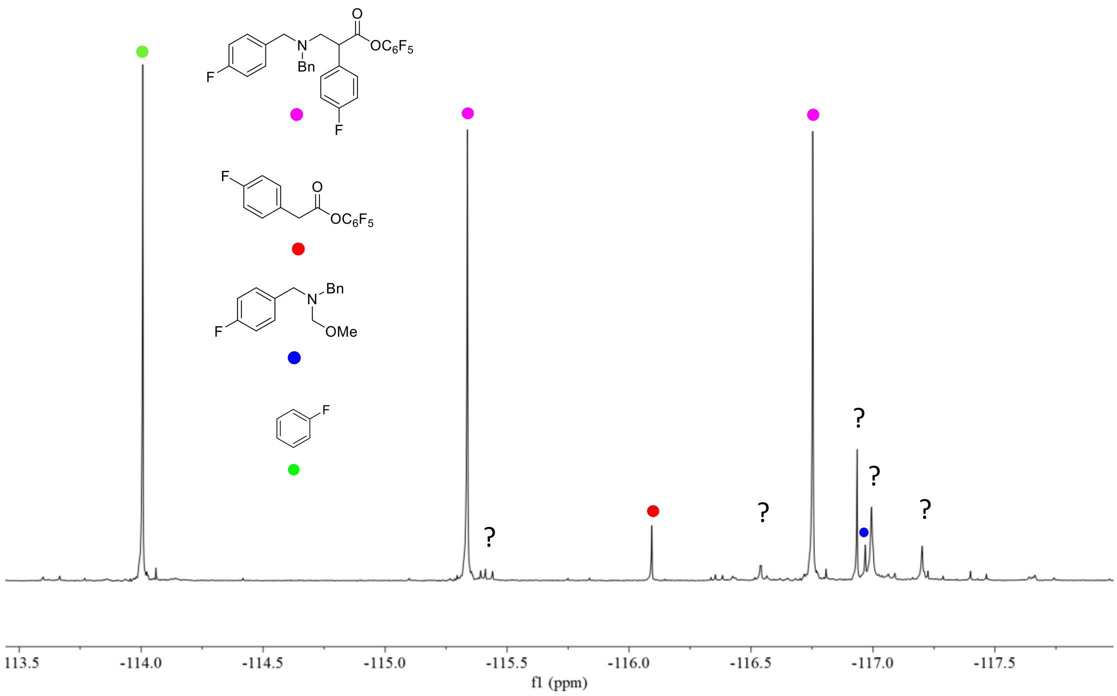

6.2 DKR vs enantioselective aminomethylation

See manuscript Table 3A

As the β-amino perfluorophenyl ester **41** could not be isolated on silica or Al_2O_3, it was generated in racemic form in situ by using racemic catalyst and used for further reaction directly.

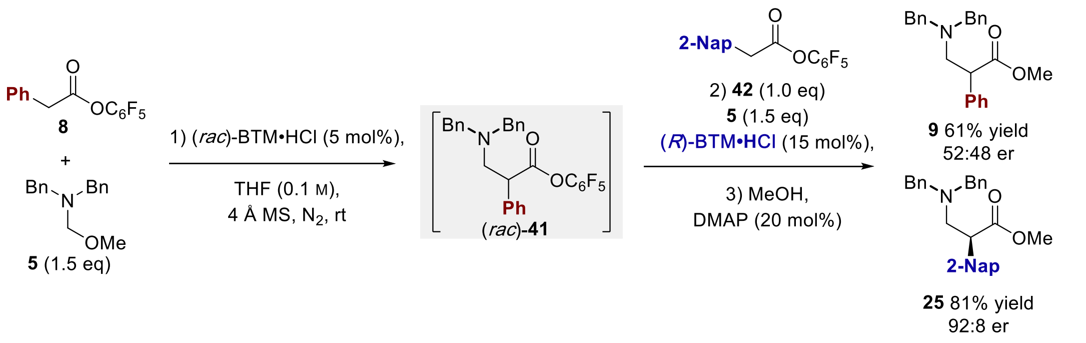

Following **General Procedure 4**, the mixture of perfluorophenyl 2-phenylacetate **8** (60.4 mg, 0.2 mmol), *N,N*-dibenzyl-1-methoxymethanamine **5** (72.3 mg, 0.3 mmol), (±)-BTM•HCl (5.8 mg, 0.02 mmol, 10 mol%) and 4 Å molecular sieves (100 mg) in anhydrous THF (2 mL, 0.1 M) was stirred at room temperature until full conversion of perfluorophenyl 2-phenylacetate **5** was observed (24 h). Then enantiopure (*R*)-BTM•HCl (8.6 mg, 0.03 mmol, 15 mol%) was added, followed by perfluorophenyl 2-(naphthalen-2-yl)acetate **42** (70.4 mg, 0.2 mmol) and *N,N*-dibenzyl-1-methoxymethanamine **5** (72.3 mg, 0.3 mmol). The new mixture was stirred continuously until full consumption of perfluorophenyl 2-(naphthalen-2-yl)acetate **42** was observed (another 24 h). Anhydrous MeOH (0.5 mL) and DMAP (4.9 mg, 0.04 mmol) were added and the reaction was stirred for another 4 h, then concentrated and subjected to column chromatography to give methyl 3-(dibenzylamino)-2-phenylpropanoate **9** (43.8 mg, 61% yiled, 52:48 er) and methyl 3-(dibenzylamino)-2-(naphthalen-2-yl)propanoate **25** (66.2 mg, 81% yield, 92:8 er).

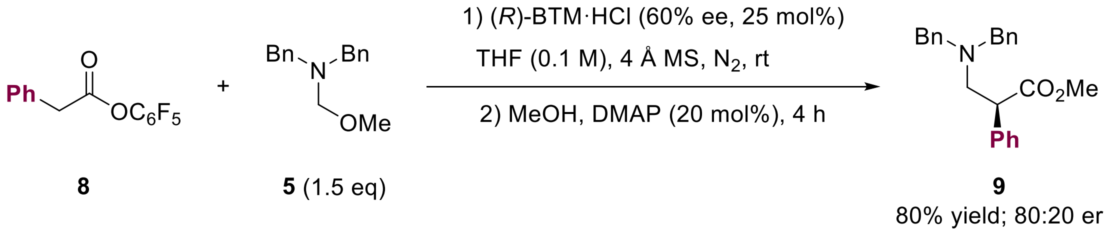

Following **General Procedure 4**, perfluorophenyl 2-phenylacetate **8** (60.4 mg, 0.2 mmol), *N,N*-dibenzyl-1-methoxymethanamine **5** (72.3 mg, 0.3 mmol), scalemic (*R*)-BTM•HCl (60% ee, 14.4 mg, 0.05 mmol, 25 mol%) and 4 Å molecular sieves (100 mg) in anhydrous THF (2 mL, 0.1 M) for 24 h then anhydrous MeOH (0.5 mL) and DMAP (4.9 mg, 0.04 mmol) for 4 h gave the crude material that was purified by column chromatography (120:1 Petrol : EtOAc) to give methyl 3-(dibenzylamino)-2-phenylpropanoate **9** (57.4 mg, 80% yield, 80:20 er).

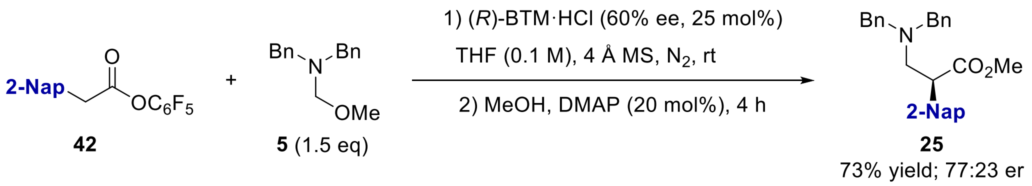

Following **General Procedure 4**, perfluorophenyl 2-(naphthalen-2-yl)acetate **42** (70.4 mg, 0.2 mmol), *N,N*-dibenzyl-1-methoxymethanamine **5** (72.3 mg, 0.3 mmol), scalemic (*R*)-BTM•HCl (60% ee, 14.4 mg, 0.05 mmol, 25 mol%) and 4 Å molecular sieves (100 mg) in anhydrous THF (2 mL, 0.1 M) for 24 h then anhydrous MeOH (0.5 mL) and DMAP (4.9 mg, 0.04 mmol) for 4 h gave the crude material that was purified by column chromatography (80:1 Petrol : EtOAc) to give methyl 3-(dibenzylamino)-2-(naphthalen-2-yl)propanoate **25** (59.7 mg, 73% yield, 77:23 er).

6.3 Relationship between catalyst and product ee

See manuscript Table 3B

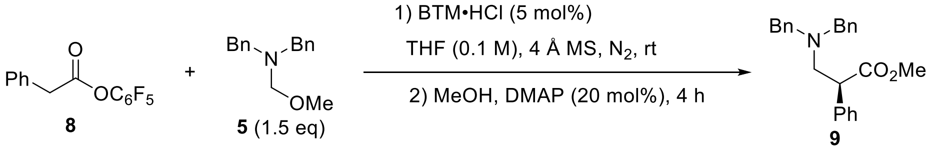

ee of BTM•HCl	ee of product **9**
0	0
17	15
39	36
59	61
79	71
100	92

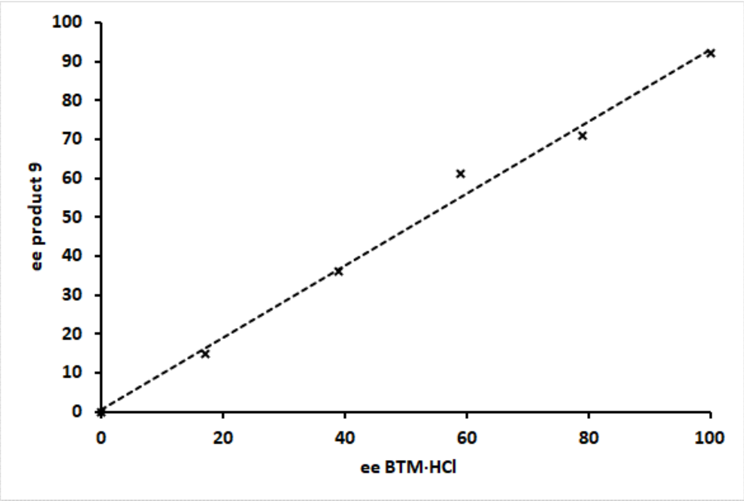

Following **General Procedure 4**, the mixture of perfluorophenyl 2-phenylacetate **8** (60.4 mg, 0.2 mmol), *N*,*N*-dibenzyl-1-methoxymethanamine **5** (72.3 mg, 0.3 mmol), scalemic (*R*)-BTM•HCl (2.8mg, 5 mol%) with known enantiopurity and 4 Å molecular sieves (100 mg) in anhydrous THF (2 mL, 0.1 M) was stirred at room temperature until full conversion of perfluorophenyl 2-phenylacetate. Anhydrous MeOH (0.5 mL) and DMAP (4.9 mg, 0.04 mmol) was added and the reaction was continued for another 4 h, then concentrated and subjected to column chromatography to give methyl 3-(dibenzylamino)-2-phenylpropanoate **9**, with enantiopurity determined by chiral HPLC. A linear correlation between the ee of product and catalyst was observed.

6.4 Acyl ammonium as a precursor

See manuscript Table 3C

Synthesis of acylated BTM

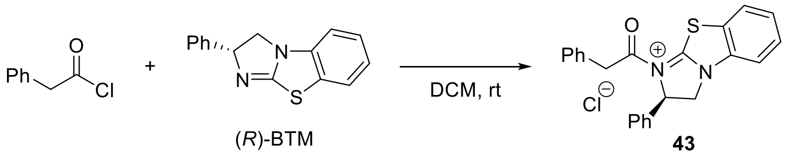

Following the procedure of Smith and co-workers.[3] Phenylacetyl chloride (72 μL, 0.55 mmol, 1.1 equiv.) was added dropwise to a solution of (*R*)-BTM (126 mg, 0.5 mmol, 1 equiv.) in CH_2Cl_2 (10 mL) at rt and the reaction was allowed to stir for 30 minutes then concentrated to half volume in *vacuo*. Et_2O was added and the precipitate filtered and washed with Et_2O to give acylated BTM **43** as a pale-yellow solid with spectroscopic data in accordance with the literature. **^{1}H NMR (400 MHz, DMSO-*d6*)** δ_H: 3.34 (1H, d, *J* 17.1, PhCH^AH^B), 4.12 (1H, d, *J* 17.1, PhC$H^A$$H^B$), 4.86 (1H, dd, *J* 11.7, 6.3, C(3)H^AH^B), 5.46 (1H, t, *J* 11.1, C(3)$H^A H^B$), 6.75 (1H, dd, *J* 10.5, 6.3, C(2)*H*), 6.95 – 7.08 (2H, m, Ar*H*), 7.19 – 7.31 (3H, m, Ar*H*), 7.44 – 7.59 (3H, m, Ar*H*), 7.60 – 7.73 (1H, m, Ar*H*), 7.76 – 7.85 (3H, m, Ar*H*), 7.95 (1H, d, *J* 8.1, ArC(5)*H*), 8.40 (1H, d, *J* 8.2, ArC(8)*H*). As NMR investigation of this compound was in CD_2Cl_2, its ^{1}H NMR spectrum is also provided here: **^{1}H NMR (400 MHz, CD_2Cl_2)** δ_H: 3.45 (1H, d, *J* 17.6, PhCH^AH^B), 4.46 (1H, d, *J* 17.5, PhCH^AH^BC), 4.70 (1H, dd, *J* 11.1, 5.3, C(3)H^AH^B), 5.96 (1H, t, *J* 10.9, C(2)*H*), 7.04 – 7.16 (2H, m), 7.21 – 7.31 (3H, m), 7.49 – 7.66 (7H, m), 7.69 – 7.80 (2H, m), 8.10 – 8.18 (1H, m).

Acylated BTM as a precatalyst

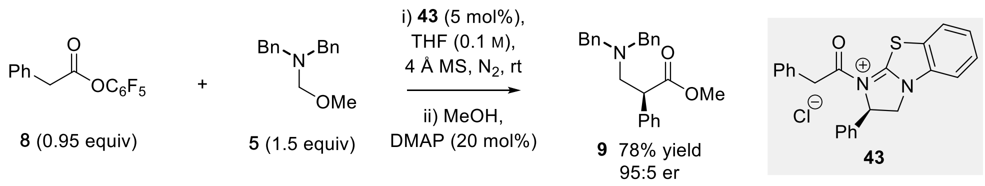

Hemiaminal ether **5** (72.3 mg, 0.3 mmol) was added to a mixture of ester **8** (57.4 mg, 0.19 mmol), acyl ammonium **43** (4.1 mg, 5 mol%) and 4 Å MS (100 mg) in anhydrous THF (2.0 mL). The resulting mixture was stirred at room temperature until full consumption of ester **8** (24 hours). Then anhydrous MeOH (0.5 mL) and DMAP (4.9 mg, 0.04 mmol) were added and the mixture was stirred at room temperature for another 4 hours. The crude mixture was then concentrated to dryness *in vacuo*, and the residue was purified by column chromatography to give desired product **9** (56.0 mg, 78% yield, 95:5 er).

6.5 Addition of Brønsted bases

See manuscript Table 3D

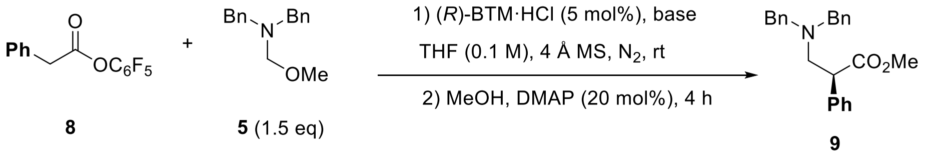

entry	Brønsted base	Time (h)	Yield (%)	Er
1	No base	24	81	96:4
2	Bn_2NEt (1.0 equiv.)	24	87	96:4
3	Bn_2NH (30 mol%)	24	74	95:5
4	iPr_2NEt (1.0 equiv.)	96	22	89:11

The mixture of perfluorophenyl 2-phenylacetate **8** (60.4 mg, 0.2 mmol), *N*,*N*-dibenzyl-1-methoxymethanamine **5** (72.3 mg, 0.3 mmol), (*R*)-BTM•HCl (2.9 mg, 0.01 mmol, 5 mol%), Brønsted base (see the table above) and 4 Å molecular sieves (100 mg) in anhydrous THF (2 mL, 0.1 M) was stirred at room temperature until full conversion of perfluorophenyl 2-phenylacetate **8** (24 h). MeOH (0.5 mL) and DMAP (4.9 mg, 0.04 mmol) were added and the reaction was stirred at room temperature for another 4 h, then concentrated and subjected to column chromatography to give desired product **9**.

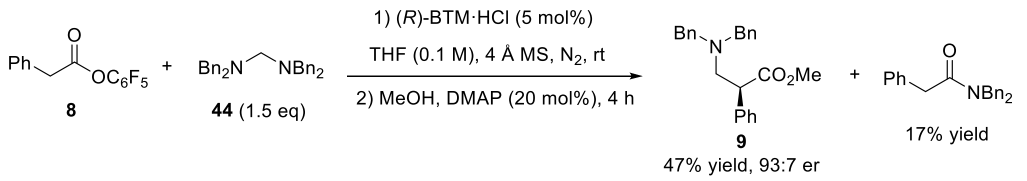

When dibenzylamine was used as an additive base, the reaction outcome only showed very slight erosion in er (entry 3). So, iminium precursor *N,N,N',N'*-tetrabenzylmethanediamine **44**,[12] which produces dibenzylamine while generating the iminium intermediate, was tested. The desired β-amino ester **9** was obtained but with only 47% yield and 93:7 er, together with some amide formation resulting from the reaction of ester substrate **8** and released dibenzylamine.

6.6 BTM alkylation

See manuscript Table 3E

Iminium salt preparation

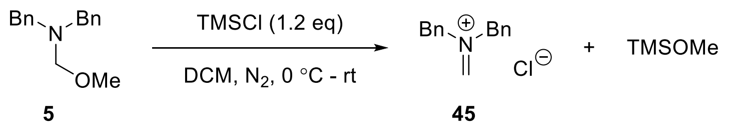

Following a modified procedure of Maulide and co-workers,[12] TMSCl (3.6 mmol) in anhydrous CH_2Cl_2 (2 mL) was added dropwise to a solution of hemiaminal ether **5** (3 mmol) in anhydrous CH_2Cl_2 (4 mL) at 0 °C under a N_2 atmosphere, then the mixture was stirred at room temperature for an hour before removal of CH_2Cl_2, TMSCl and TMSOMe in *vacuo* to give iminium salt **45** as a white and hydroscopic solid which was used without further purification. **^{1}H NMR (500 MHz, CD_2Cl_2)** δ_H: 4.13 (4H, s, NCH_2Ph), 6.08 (2H, s, Bn_2NCH_2), 7.34 – 7.47 (10H, m, ArC*H*); **^{13}C NMR (126 MHz, CD_2Cl_2)** δ_C: 56.9 (NCH_2Ph), 98.6 (Bn_2NCH_2), 128.2 (Ar*C*(4)H), 128.8 (Ar*C*(3,5)H), 129.4 (Ar*C*(2,6)H), 135.3 (Ar*C*(1)); **HRMS (ESI$^+$)** $C_{15}H_{16}N$ $[M-Cl]^+$ found 210.1273, requires 210.1277 (-2.0 ppm).

Iminium salt used directly

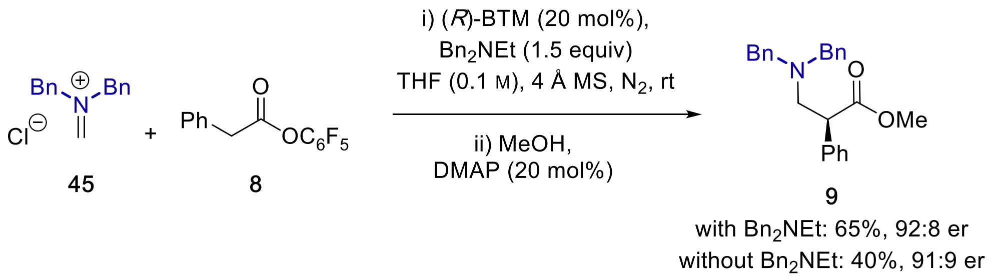

Approximately following **General Procedure 4**, a mixture of perfluorophenyl 2-phenylacetate **8** (60.4 mg, 0.2 mmol), iminium ion **45** (73.8 mg, 0.3 mmol), (*R*)-BTM (11.5 mg, 0.04 mmol) and 4 Å MS (100 mg) in anhydrous THF (2 mL) was stirred at room temperature until the full conversion of perfluorophenyl 2-phenylacetate **8** (24 h). Then the reaction was treated with anhydrous MeOH and DMAP for 4 hours before being subjected to column chromatography to give desired product **9** (28.7 mg, 40% yield, 91:9 er). If dibenzylethylamine (1.5 equiv.) was added to accelerate the productive pathway, the desired product **9** could be obtained with a better outcome (46.7 mg, 65% yield, 92:8 er).

Formation of alkylated BTM

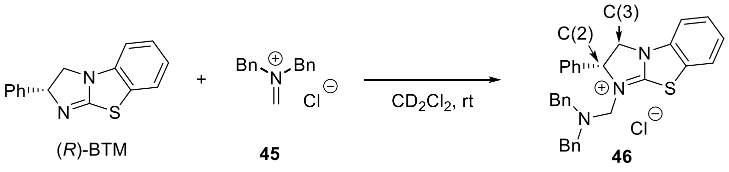

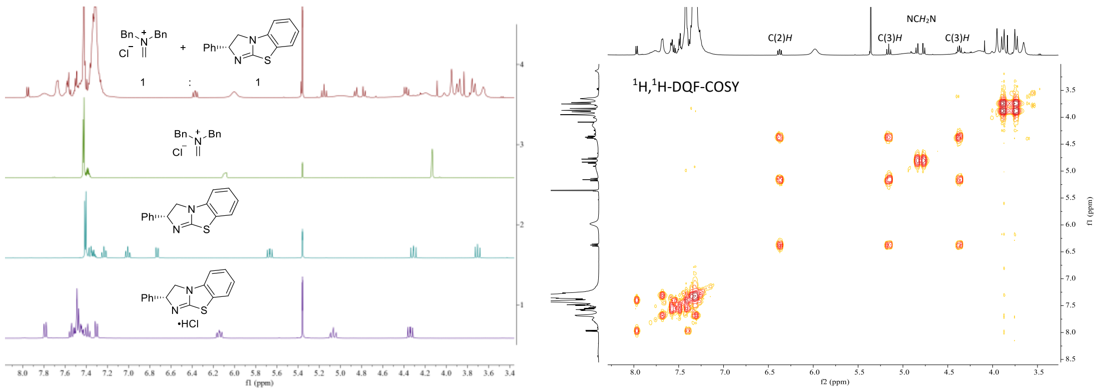

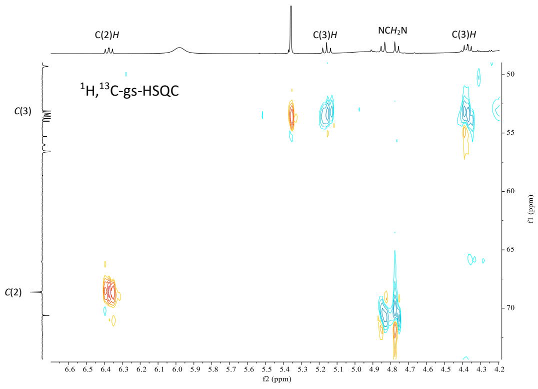

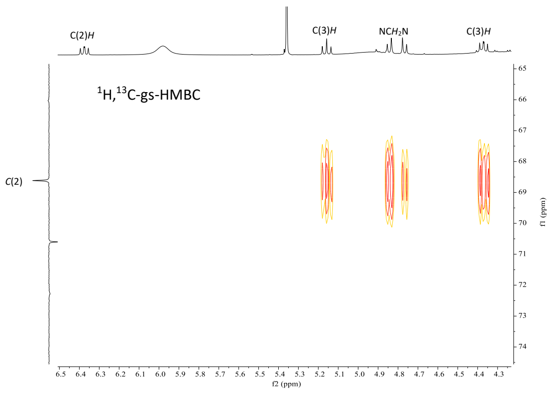

(*R*)-BTM (0.1 mmol) and iminium ion (0.1 mmol) were dissolved in anhydrous CD_2Cl_2 (1 mL). The mixture was then analysed by NMR: (list experiments) Alkylated BTM was identified alongside some other unidentified species. The formation of alkylated BTM was confirmed by confirming connectivity as outlined below.

From **^{1}H,^{1}H-DQF-COSY** NMR spectrum, the three protons [δ_H: 4.38 (dd, *J* 10.7, 8.9), 5.15 (t, *J* 10.2), 6.37 (dd, *J* 11.0, 8.9)] at C(2) and C(3) of the BTM scaffold can be identified. The crosspeak at δ_H: 6.37 and δ_C: 68.8 on the **^{1}H,^{13}C-gs-HSQC** NMR spectrum, enables the assignment of carbon signal for *C*(2)H (68.6 ppm). The signals associated with the proposed N*C*H_2N environment can also be identified: H [δ_H: 4.78 (d, *J* 10.3)], H [δ_H: 4.86 (d, *J* 10.2)] and C (δ_C: 70.6), with the blue **^{1}H,^{13}C-gs-HSQC** crosspeak indicating their connectivity. On **^{1}H,^{13}C-gs-HMBC** NMR spectrum, the correlation between H (4.78 ppm and 4.86 ppm) and C(2) (δ_C: 68.6) indicates through bond coupling and so connectivity of CH_2 (δ_C:70.6) and C(2).

Notably the diagnostic diastereotopic NC*H*$_2$NBn$_2$ signals [δH: 4.78 (d, *J* 10.3) and 4.86 (d, *J* 10.2)] correlates to C(2) (δC: 68.6) while the NC*H*$_2$N protons also appear as inequivalent doublets indicative of their connectivity to stereogenic centre-containing BTM.

Alkylated BTM as precatalysts
See manuscript reference [37]

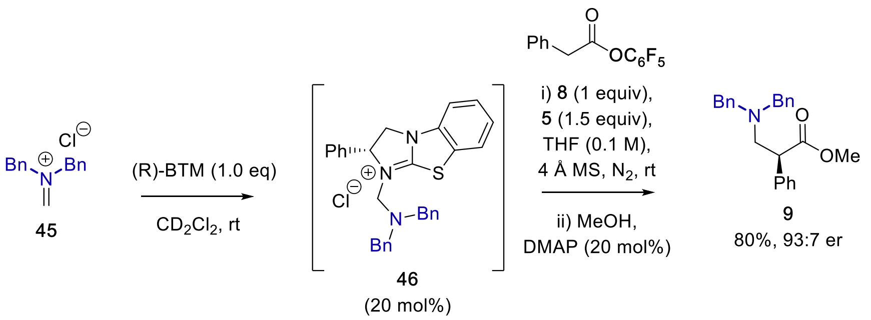

(R)-BTM (100.8 mg, 0.4 mmol) and *N,N*-dibenzyliminium chloride **45** (98.4 mg, 0.4 mmol) were dissolved in CD_2Cl_2 (1 mL) and the resulting solution was stirred at room temperature for 30 minutes. Then the solution was characterized by ^1H NMR to confirm the formation of alkylated BTM **46**. The solution was used as a stock solution of alkylated BTM **46** (0.4 M in CD_2Cl_2).

The mixture of perfluorophenyl 2-phenylacetate **8** (60.4 mg, 0.2 mmol), hemiaminal ether **5** (72.3 mg, 0.3 mmol) and 4 Å MS (100 mg) in anhydrous THF (2 mL) was stirred at room temperature and under a N_2 atmosphere. Then alkylated BTM **46** (100 μL, 0.4 M in CD_2Cl_2) was added and the reaction was stirred at room temperature until full conversion of perfluorophenyl 2-phenylacetate **8** (15 h). Anhydrous MeOH (0.5 mL) and DMAP (4.9 mg, 0.04 mmol) were added and the reaction was stirred at room temperature for another 4 h. The solvent was removed in *vacuo* and the residue was purified by column chromatography to give desired product **9** (57.4 mg, 80% yield, 93:7 er).

6.7 Chiral counterion control of enantioselectivity

See manuscript Table 3F

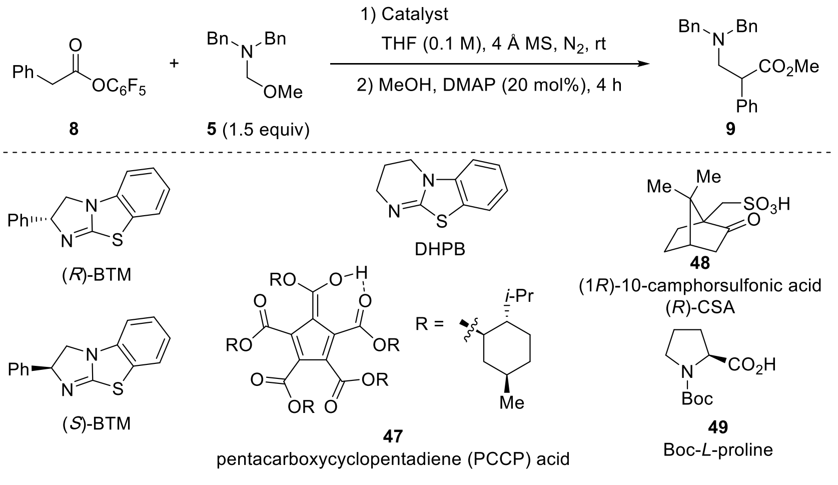

entry	Catalyst	Time (h)	Yield (%)[b]	er[c]
1	(*R*)-BTM (20 mol%) + **47**[d] (20 mol%)	24	81	68:32
2	(*S*)-BTM (20 mol%) + **47** (20 mol%)	24	78	24:76
3	DHPB (20 mol%) + **47** (20 mol%)	24	49	53:47
4	(*R*)-BTM (20 mol%) + **48** (20 mol%)	24	76	77:23
5	(*S*)-BTM (20 mol%) + **48** (20 mol%)	24	81	24:76
6	DHPB (20 mol%) + **48** (20 mol%)	24	68	50:50
7	(*R*)-BTM (20 mol%) + **49** (20 mol%)	24	56	85:15
8	(*S*)-BTM (20 mol%) + **49** (20 mol%)	24	53	18:82
9	DHPB (20 mol%) + **49** (20 mol%)	24	39	50:50

[a] **5** (0.3 mmol) was added to a stirred mixture of **8** (0.2 mmol), catalyst, and 4 Å molecular sieves (MS, 100 mg) in anhydrous THF (2 mL, 0.1 M) at rt under a N_2 atmosphere for up to 24 hours before treatment with MeOH (0.5 mL) and DMAP (20 mol%) for 4 h. [b] Isolated yield. [c] Determined by HPLC analysis on a chiral stationary phase. [d] **47** was synthesized according to Lambert's procedure.[22]

6.8 Substrate control of stereoselectivity

See manuscript Table 3G

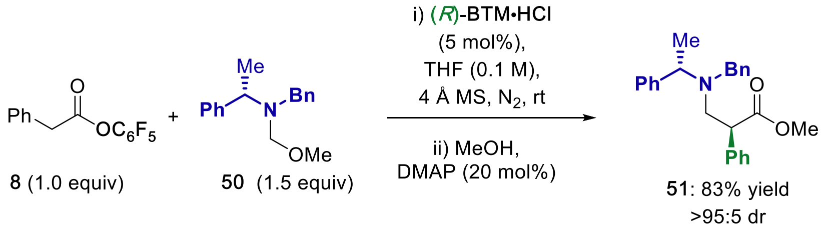

Following **General Procedure 4**, perfluorophenyl 2-phenylacetate **8** (60.4 mg, 0.2 mmol), (*S*)-*N*-benzyl-*N*-(methoxymethyl)-1-phenylethan-1-amine **50** (76.5 mg, 0.3 mmol), (*R*)-BTM·HCl (2.9 mg, 0.01 mmol) and 4 Å molecular sieves (100 mg) in anhydrous THF (2.0 mL) for 40 hours then anhydrous MeOH (0.5 mL) and DMAP (4.9 mg, 0.04 mmol) for 4 hours gave the crude material that was purified by column chromatography (120:1 Petrol : EtOAc) to give desired compound **51** as a colorless oil (61.9 mg, 83% yield, dr >95:5). Diastereoselectivity was determined by comparing ^{1}H NMR signal integrations of the two diastereomers. $[\alpha]_D^{20}$ –11.6 (*c* 1.2, $CHCl_3$); **IR** ν_{max} (film) 1734 (C=O); **^{1}H NMR (400 MHz, $CDCl_3$)** δ_H: 1.38 (1H, d, *J* 6.9, CHC*H*$_3$), 2.93 (1H, dd, *J* 13.1, 6.0, C(3)*H*^{A}H^B), 3.25 (1H, dd, *J* 13.1, 9.4, C(3)H^A*H*^{B}), 3.58 (3H, s, OC*H*$_3$), 3.64 (2H, s, NC*H*$_2$Ph), 3.79 (1H, dd, *J* 9.3, 6.0, C(2)*H*), 4.00 (1H, q, *J* 6.9, C*H*CH$_3$), 7.22 – 7.42 (15H, m, ArC*H*); **^{13}C NMR (101 MHz, $CDCl_3$)** δ_C: 13.7 (CHCH$_3$), 51.5 (*C*(2)H), 51.8 (OCH$_3$), 54.2 (*C*(3)H$_2$), 54.8 (NCH$_2$Ph), 57.9 (*C*HMePh), 126.8 (NCHMePh*C*(4)H), 126.9 (NCH$_2$Ph*C*(4)H), 127.3 (C(2)HPhC(4)H), 128.0 (ArCH), 128.1 (ArCH), 128.2 (ArCH),

128.3 (Ar*C*H), 128.5 (C(2)HPh*C*(3,5)H), 128.8 (C(2)HPh*C*(2,6)H), 137.6 (C(2)HPh*C*(1)), 140.2 (NCH_2Ph*C*(1)), 142.9 (CHMePh*C*(1)), 173.5 (*C*(1)); **HRMS (ESI$^+$)** $C_{25}H_{28}NO_2$ $[M+H]^+$ found 374.2103, requires 374.2115 (–3.1 ppm).

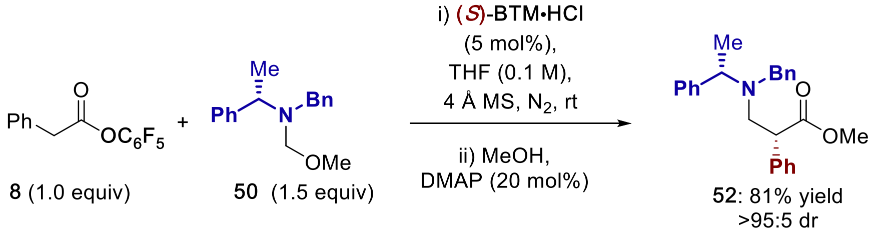

Following **General Procedure 4**, perfluorophenyl 2-phenylacetate **8** (60.4 mg, 0.2 mmol), (*S*)-*N*-benzyl-*N*-(methoxymethyl)-1-phenylethan-1-amine **50** (76.5 mg, 0.3 mmol), (*S*)-BTM·HCl (2.9 mg, 0.01 mmol) and 4 Å molecular sieves (100 mg) in anhydrous THF (2.0 mL) for 40 hours then anhydrous MeOH (0.5 mL) and DMAP (4.9 mg, 0.04 mmol) for 4 hours gave the crude material that was purified by column chromatography (120:1 Petrol : EtOAc) to give desired compound **52** as a colorless oil (60.4 mg, 81% yield, dr >95:5). Diastereoselectivity was determined by comparing ^{1}H NMR signal integrations of the two diastereomers. $[\alpha]_D^{20}$ –12.4 (*c* 1.2, $CHCl_3$); **IR** ν_{max} (film) 1734 (C=O); **^{1}H NMR (300 MHz, $CDCl_3$)** δ_H: 1.46 (1H, d, *J* 6.9, $CHCH_3$), 2.69 (1H, dd, *J* 13.1, 5.9, C(3)H^AH^B), 3.45 (1H, dd, *J* 13.1, 9.5, C(3)H^AH^B), 3.53 – 3.71 (3H, m, NCH_2Ph and C(2)*H*), 3.73 (3H, s, OCH_3), 3.98 (1H, q, *J* 6.8, *C*HMePH), 7.12 – 7.22 (2H, m, ArC*H*), 7.23 – 7.42 (13H, m, ArC*H*); **^{13}C NMR (101 MHz, $CDCl_3$)** δ_C: 14.4 ($CHCH_3$), 51.6 (*C*(2)H), 51.9 (OCH_3), 54.1 (*C*(3)H_2), 54.9 (NCH_2Ph), 57.9 (*C*HMePh), 126.8 (NCHMePh*C*(4)H), 126.9 (NCH_2Ph*C*(4)H), 127.3 (C(2)HPh*C*(4)H), 128.0 (ArCH), 128.1 (ArCH), 128.20 (ArCH), 128.23 (ArCH), 128.5 (C(2)HPh*C*(3,5)H), 128.8 (C(2)HPh*C*(2,6)H), 137.3 (C(2)HPh*C*(1)), 140.2 (NCH_2Ph*C*(1)), 142.2 (CHMePh*C*(1)), 173.9 (*C*(1)); **HRMS (ESI$^+$)** $C_{25}H_{28}NO_2$ $[M+H]^+$ found 374.2105, requires 374.2115 (–2.6 ppm).

7 References

[1] Schwarz, K. J.; Amos, J. L.; Klein, J. C.; Do, D. T.; Snaddon, T. N. *J. Am. Chem. Soc.* **2016**, *138*, 5214.

[2] Yu, J.; Chen, L.; Sun, J. *Org. Lett.* **2019**, *21*, 1664.

[3] Young, C. M.; Stark, D. G.; West, T. H.; Taylor, J. E.; Smith, A. D. *Angew. Chem., Int. Ed.* **2016**, *55*, 14394.

[4] Damakaci, F.; DeShong, P. *J. Am. Chem. Soc.* **2003**, *125*, 4408.

[5] Jiang, X.; Beiger, J. J.; Hartwig, J. F. *J. Am. Chem. Soc.* **2017,** *139*, 87.

[6] Schwarz, K. J.; Pearson, C. M.; Cintron-Rosado, G. A.; Liu, P.; Snaddon, T. N. *Angew. Chem., Int. Ed.* **2018,** *57*, 7800-7803.

[7] Vicuron Pharmaceuticals Inc. Ramoplanin derivatives possessing antibacterial activity. U.S. Patent 2006211603, September 21, 2006.

[8] Song, J.; Zhang, Z. J.; Chen, S. S.; Fan, T.; Gong, L. Z. *J. Am. Chem. Soc.* **2018,** *140,* 3177.

[9] Hutchings-Goetz, L.; Yang, C.; Snaddon, T. N. *ACS Catal.* **2018**, *8*, 10537.

[10] Szostak, M.; Spain, M.; Procter, D. J., *J. Am. Chem. Soc.* **2014,** *136*, 8459.

[11] Sakai, N.; Shimamura, K.; Ikeda, R.; Konakahara, T. *J. Org. Chem.* **2010**, *75*, 3923.

[12] Kaiser, D.; Tona, C.; Gonçalves, C. R.; Shaaban, S.; Oppedisano, A.; Manlide, N. *Angew. Chem., Int. Ed.* **2019**, *58*, 14639.

[13] Meyer, D.; Marti, R.; Seebach, D. *Eur. J. Org. Chem.* **2015**, *2015*, 4883.

[14] CrystalClear-SM Expert v2.1. Rigaku Americas, The Woodlands, Texas, USA, and Rigaku Corporation, Tokyo, Japan, 2015

[15] CrysAlisPro v1.171.38.46. Rigaku Oxford Diffraction, Rigaku Corporation, Oxford, U.K. 2015

[16] Sheldrick, G. M. *Acta Crystallogr., Sect. A.* **2015**, *71*, 3–8.

[17] Sheldrick, G. M. *Acta Crystallogr., Sect. C.* **2015**, *71*, 3–8.

[18] Dolomanov, O.V.; Bourhis, L.J.; Gildea, R.J.; Howard, J.A.K.; Puschmann, H. *J. Appl. Cryst.* **2009**, *42*, 339-341.

[19] Davies, H. M.; Ni, A. *Chem. Commun.* **2006**, 3110.

[20] Garg, Y.; Pandey, S. K. *RSC Adv.* **2016**, *6*, 25913.

[21] Davies, S. G.; Lee, J. A.; Roberts, P. M.; Shah, R. S.; Thomson, J. E. *Chem. Commun.* **2012**, *48*, 9236.

[22] Radtke, M. A.; Dudley, C. C.; O'Leary, J. M.; Lambert, T. H. *Synthesis*, **2019**, *51*, 1135.

Appendix I: NMR Spectra

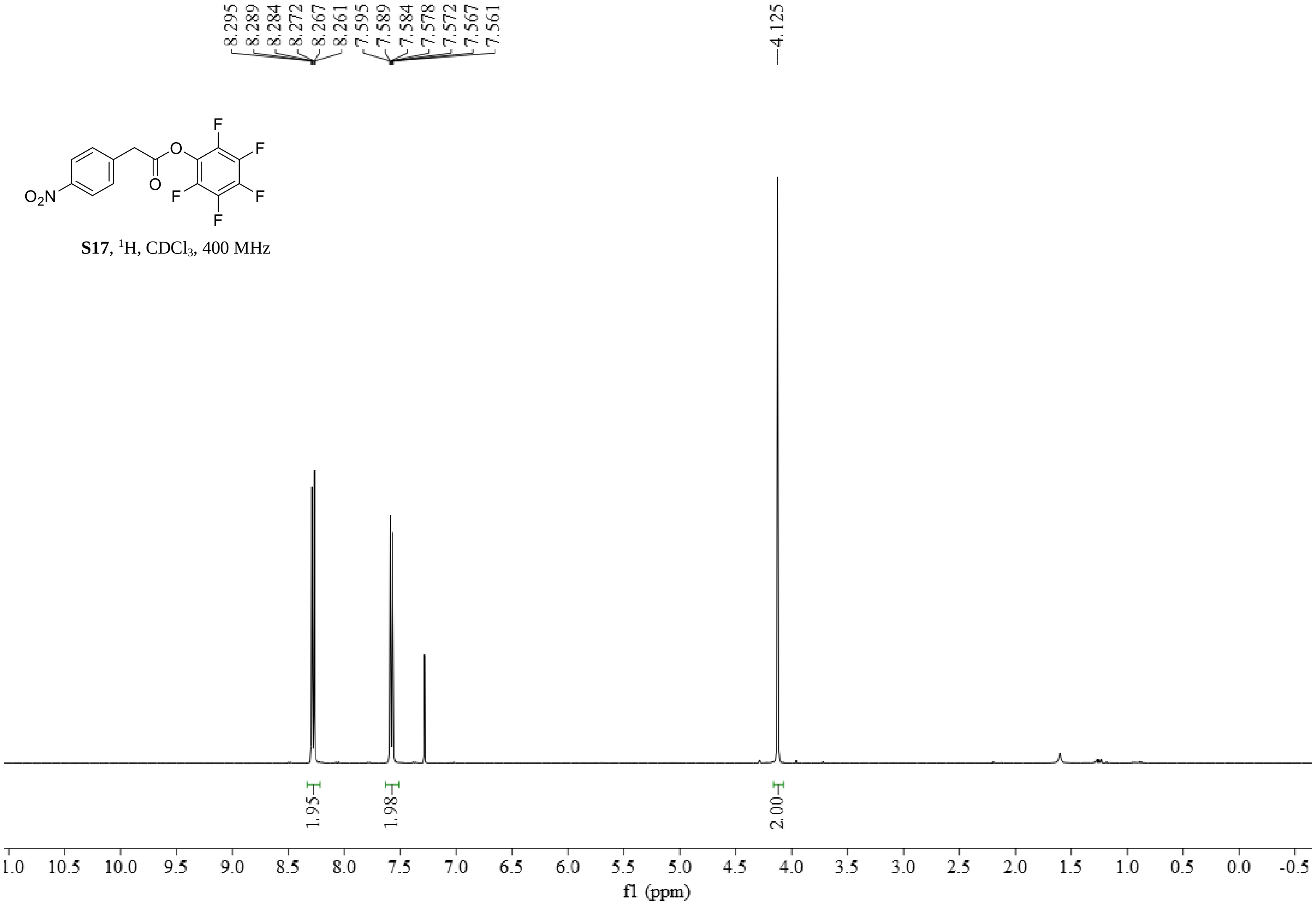

S17, ^{1}H, $CDCl_3$, 400 MHz

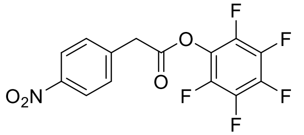

S17, ^{19}F, $CDCl_3$, 377 MHz

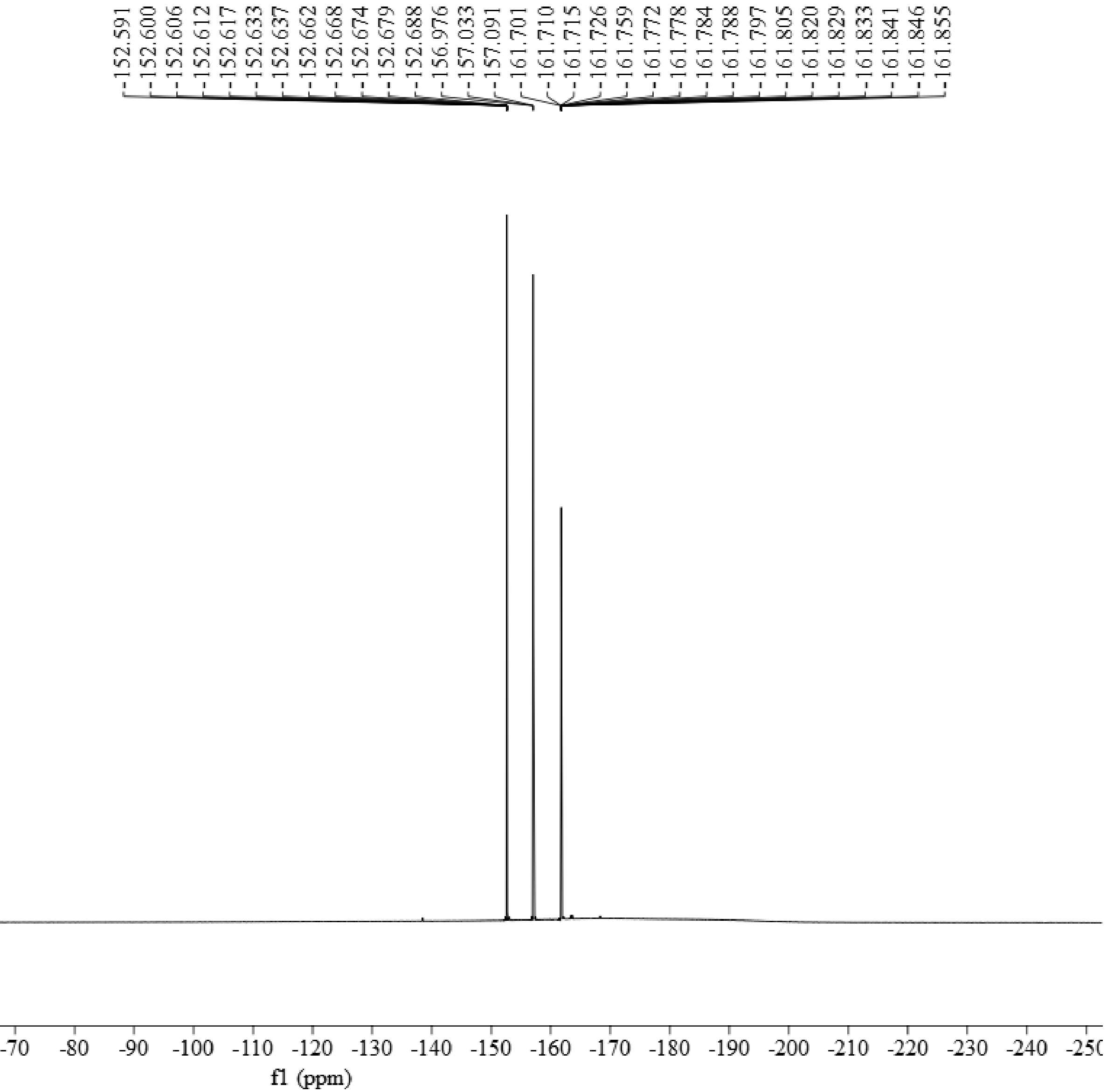

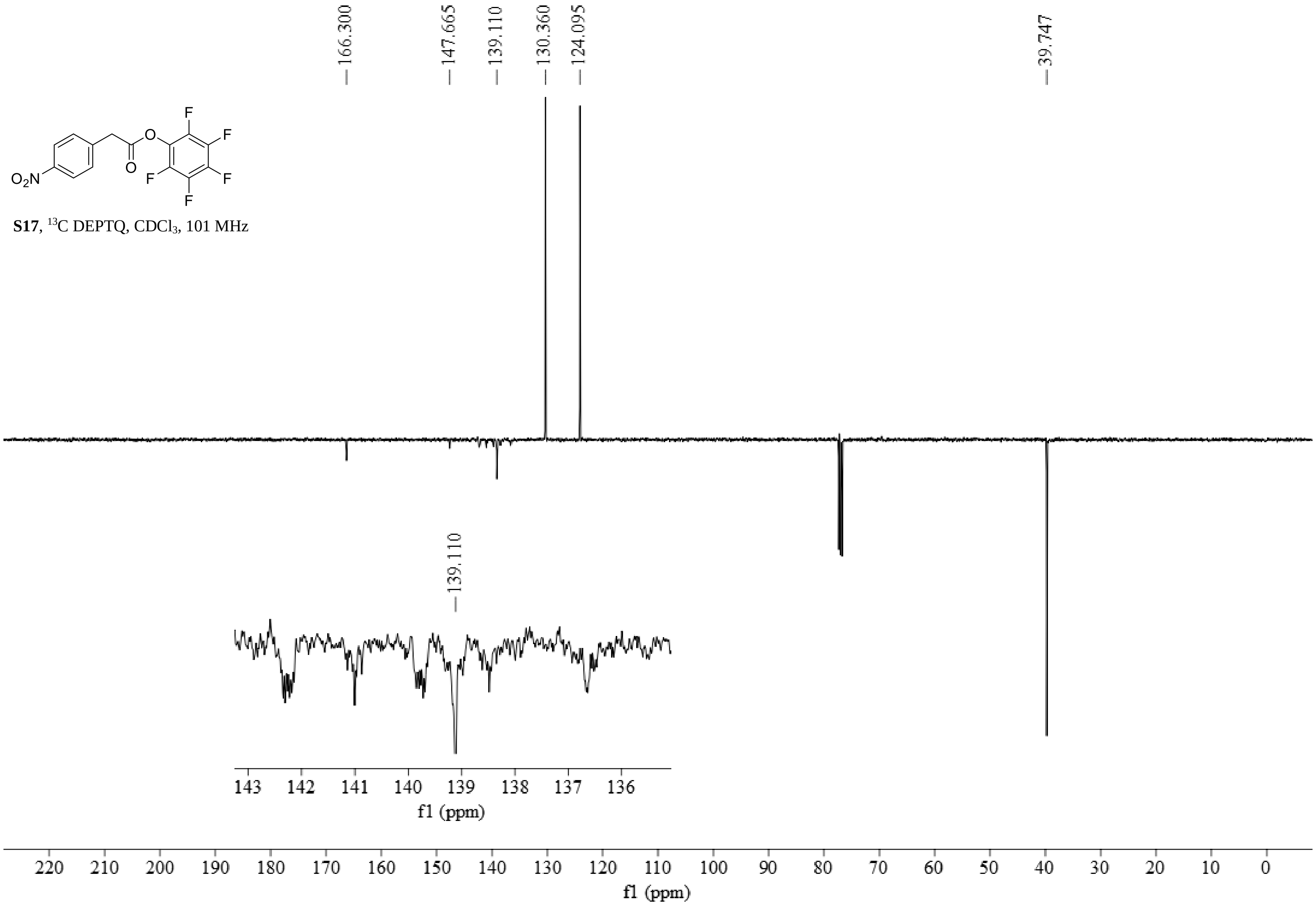

S17, ^{13}C DEPTQ, $CDCl_3$, 101 MHz

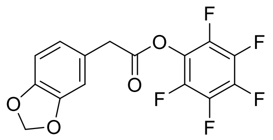

S19, ^{1}H, $CDCl_3$, 500 MHz

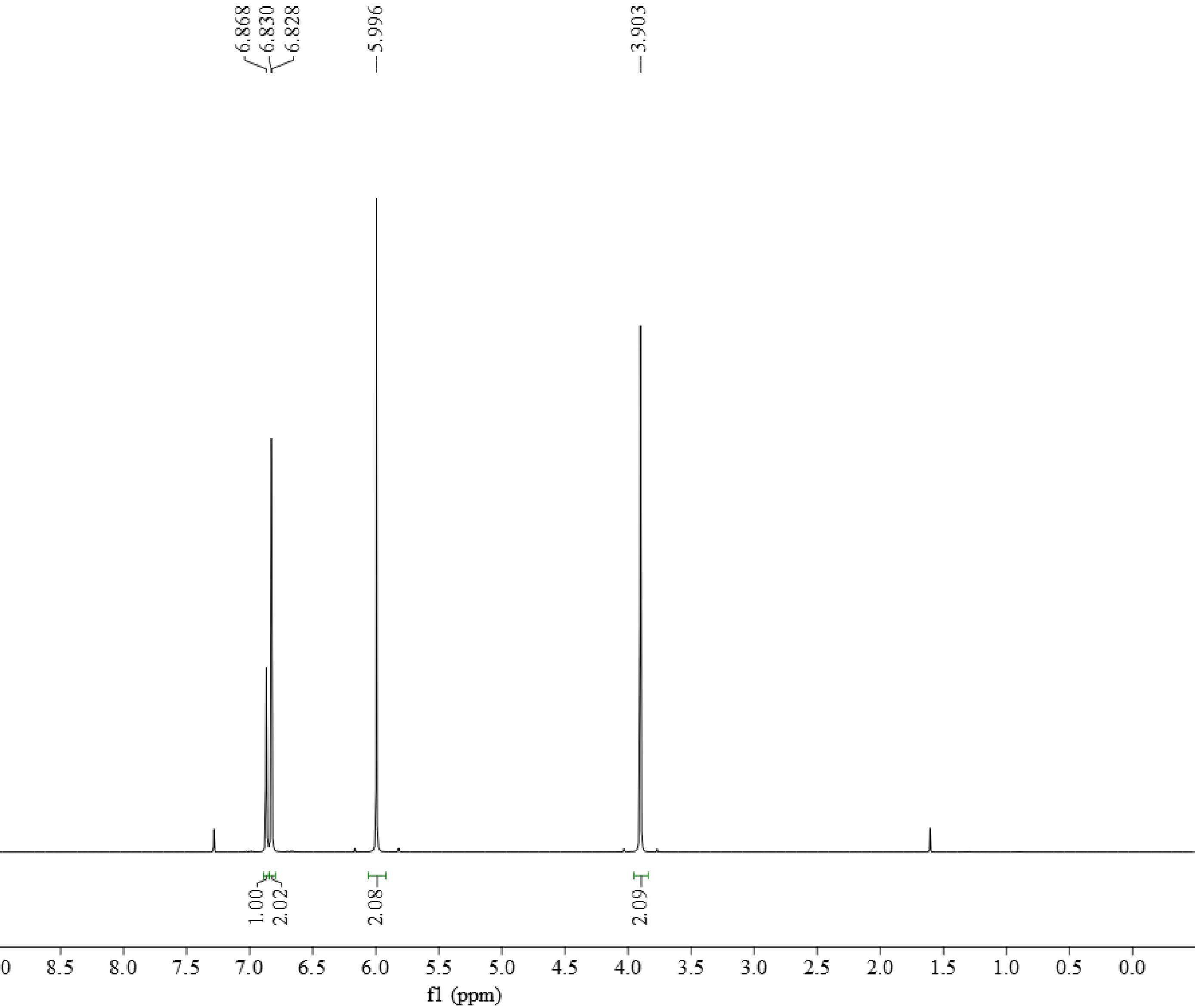

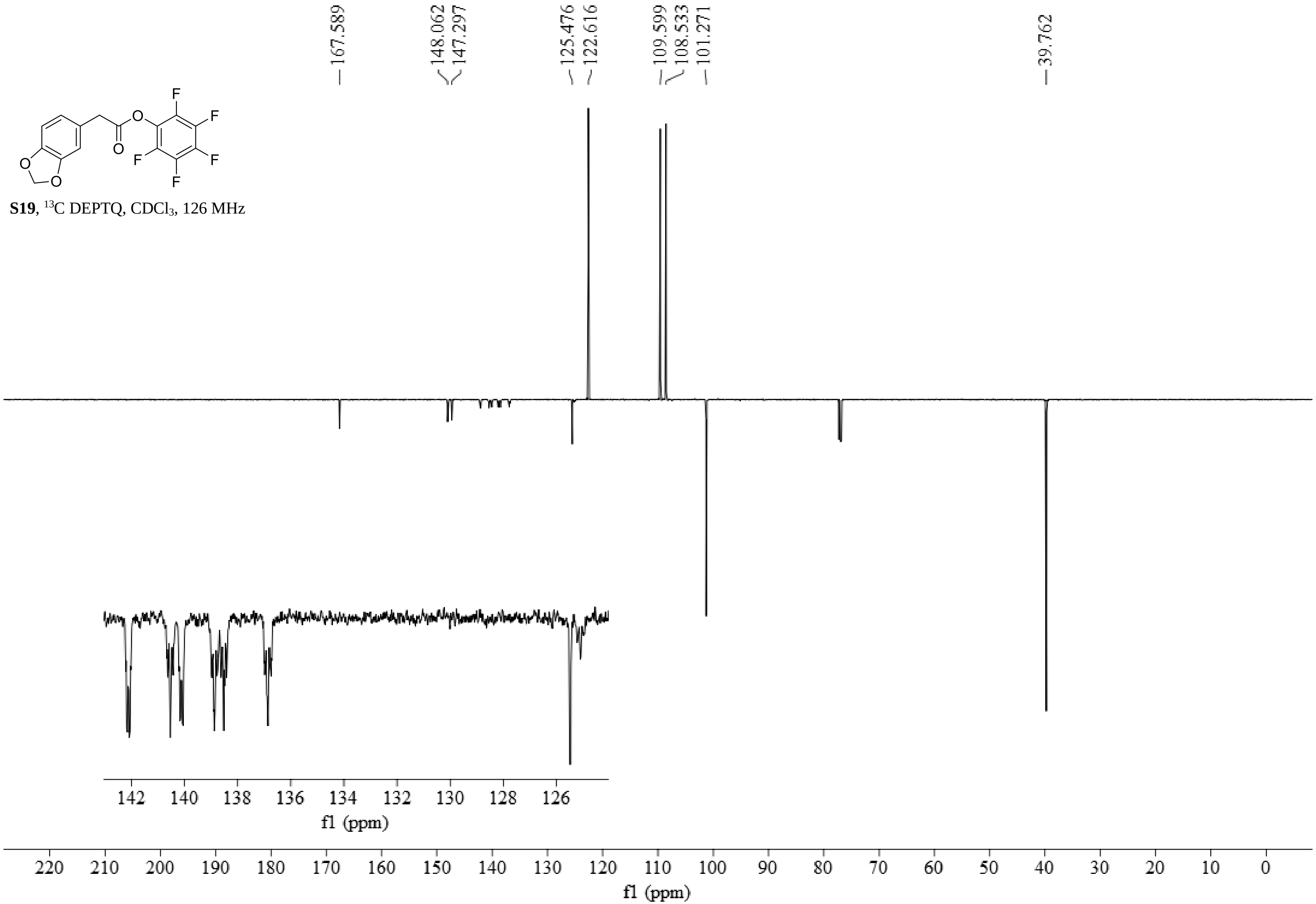

S19, ^{13}C DEPTQ, $CDCl_3$, 126 MHz

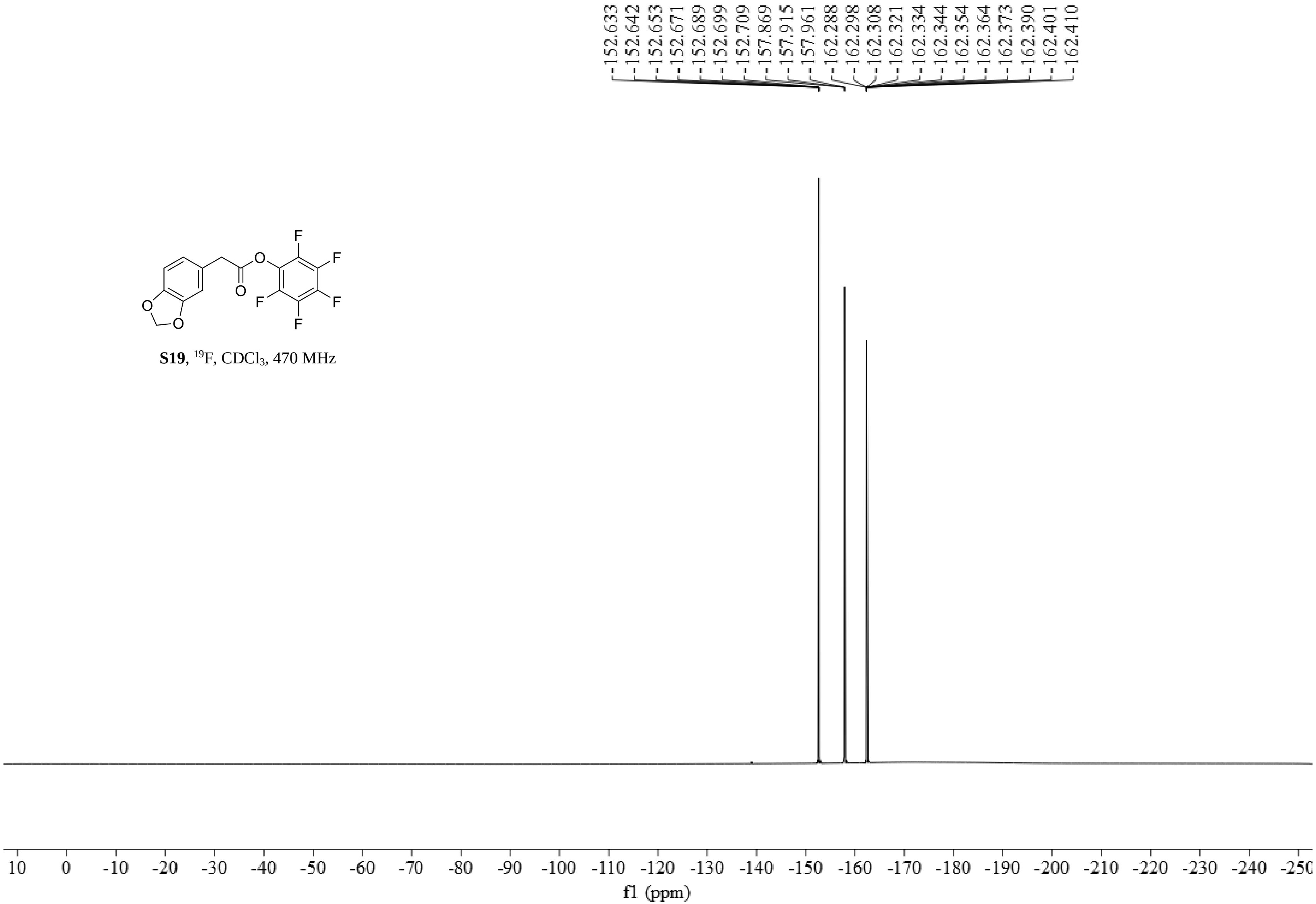

S19, ^{19}F, $CDCl_3$, 470 MHz

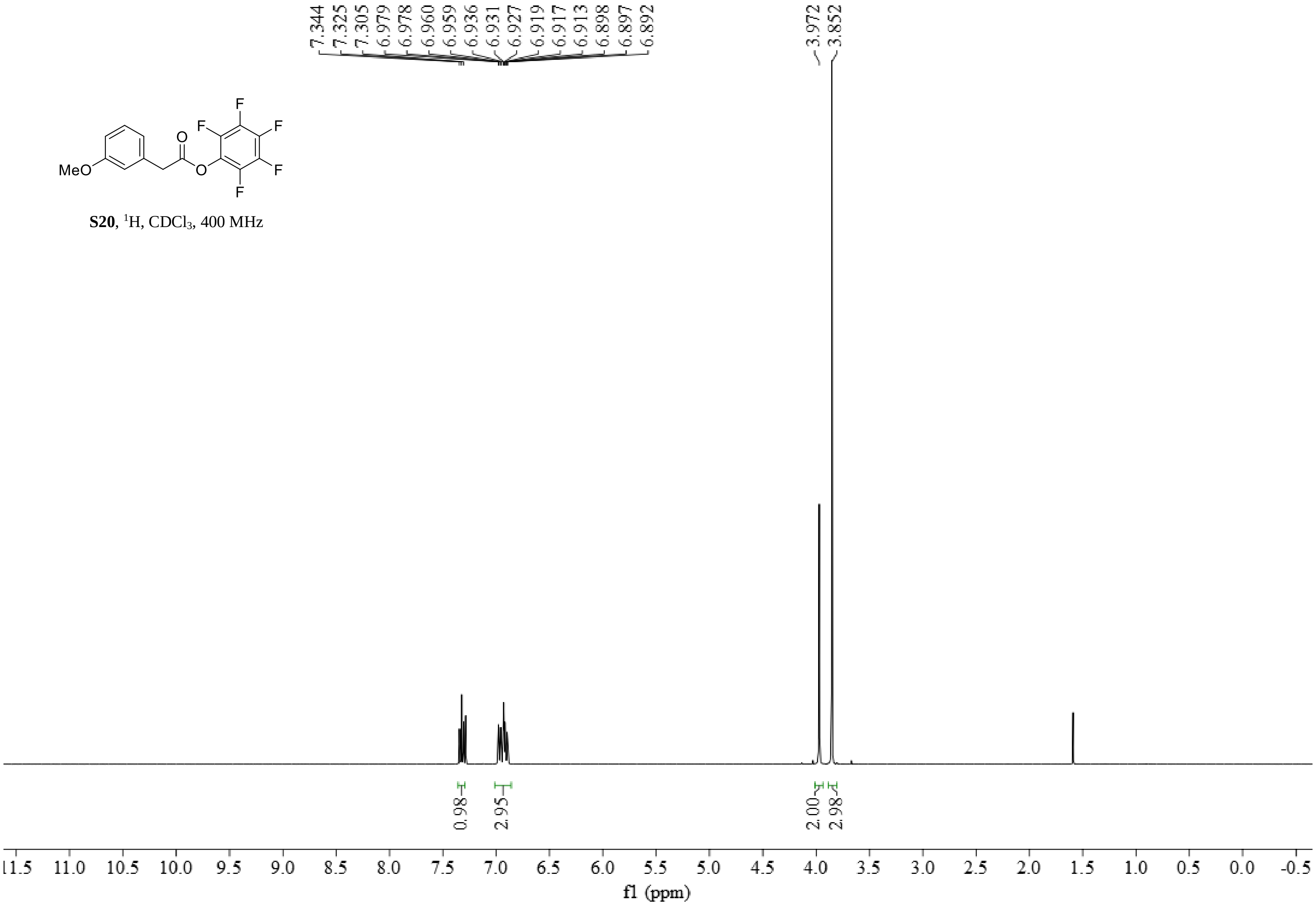

S20, ^{1}H, $CDCl_3$, 400 MHz

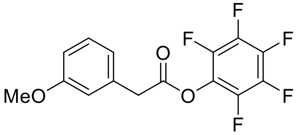

S20, ^{19}F, $CDCl_3$, 376 MHz

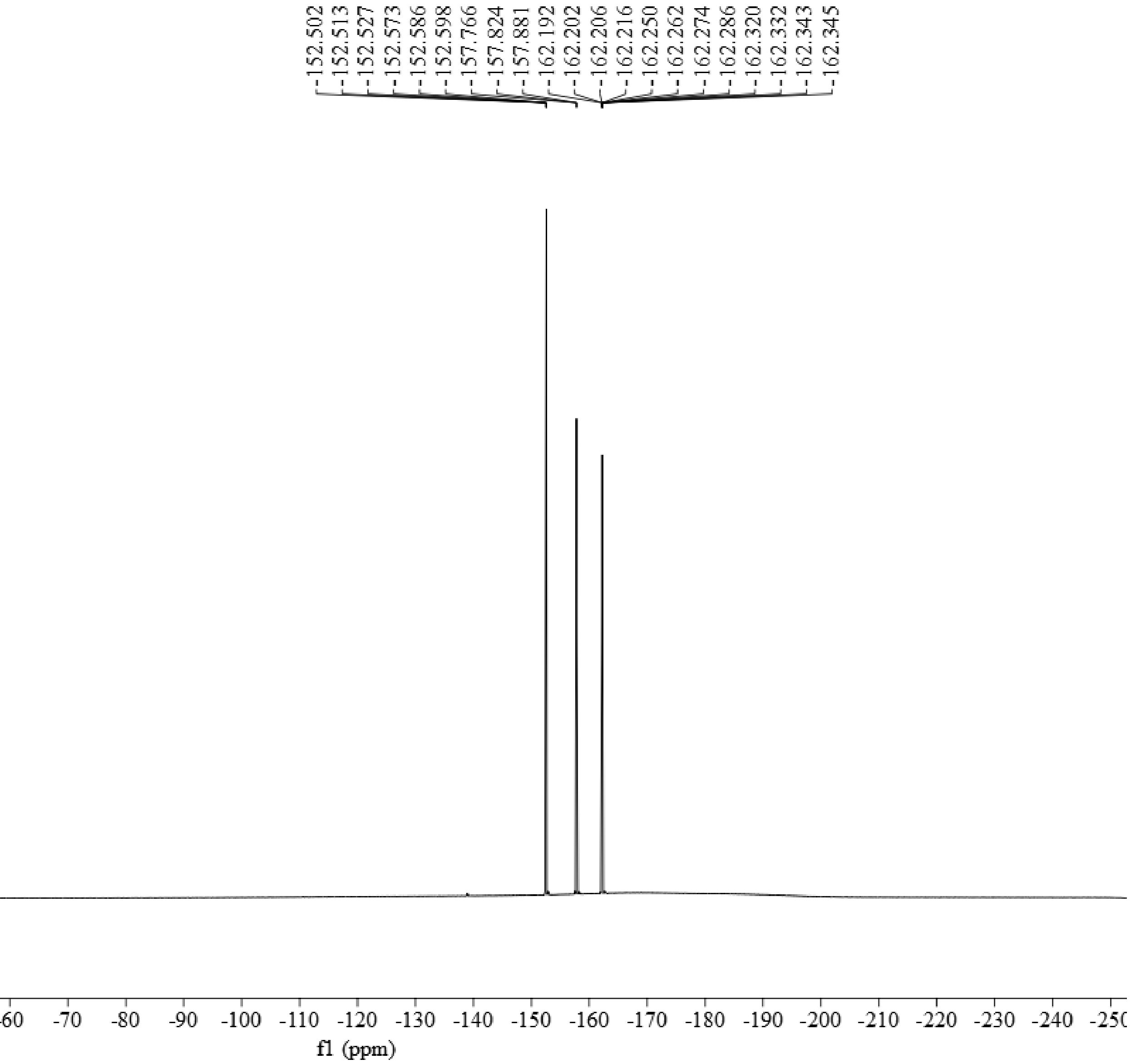

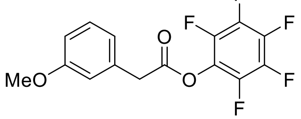

S20, ^{13}C DEPTQ, $CDCl_3$, 101 MHz

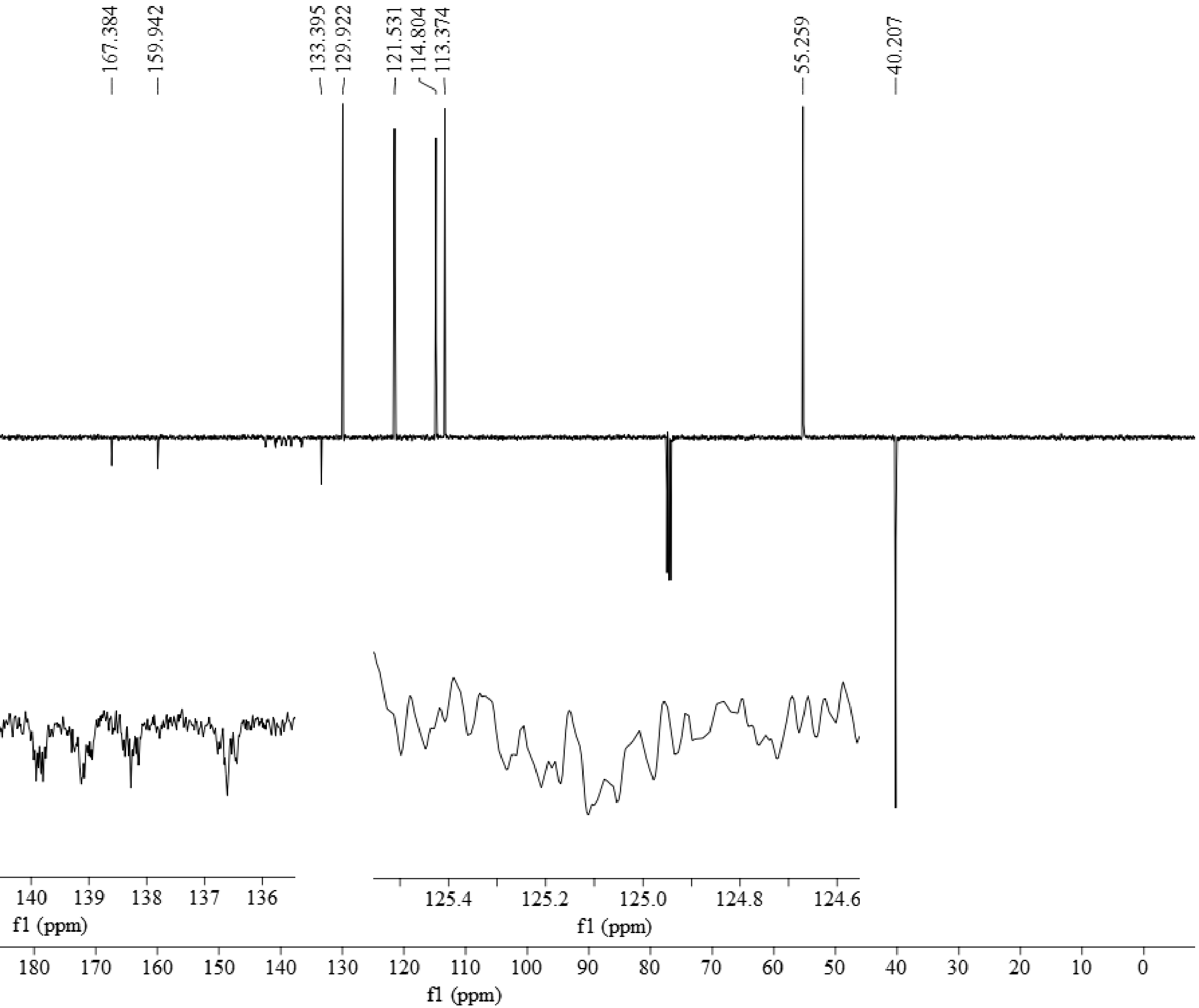

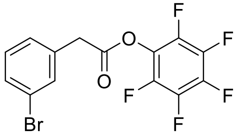

S21, ^{1}H, $CDCl_3$, 400 MHz

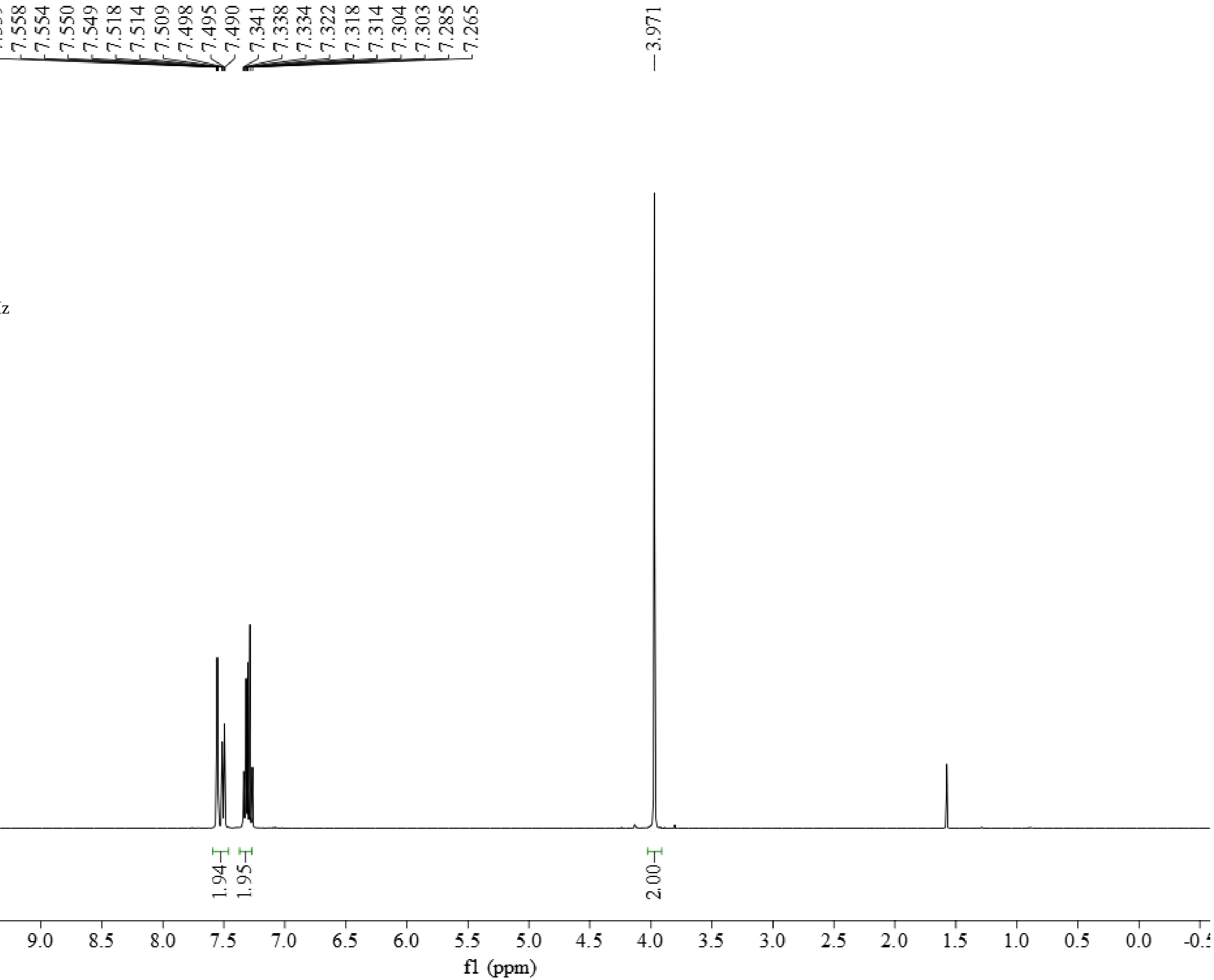

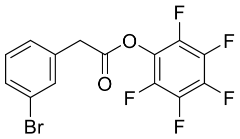

S21, ^{19}F, $CDCl_3$, 376 MHz

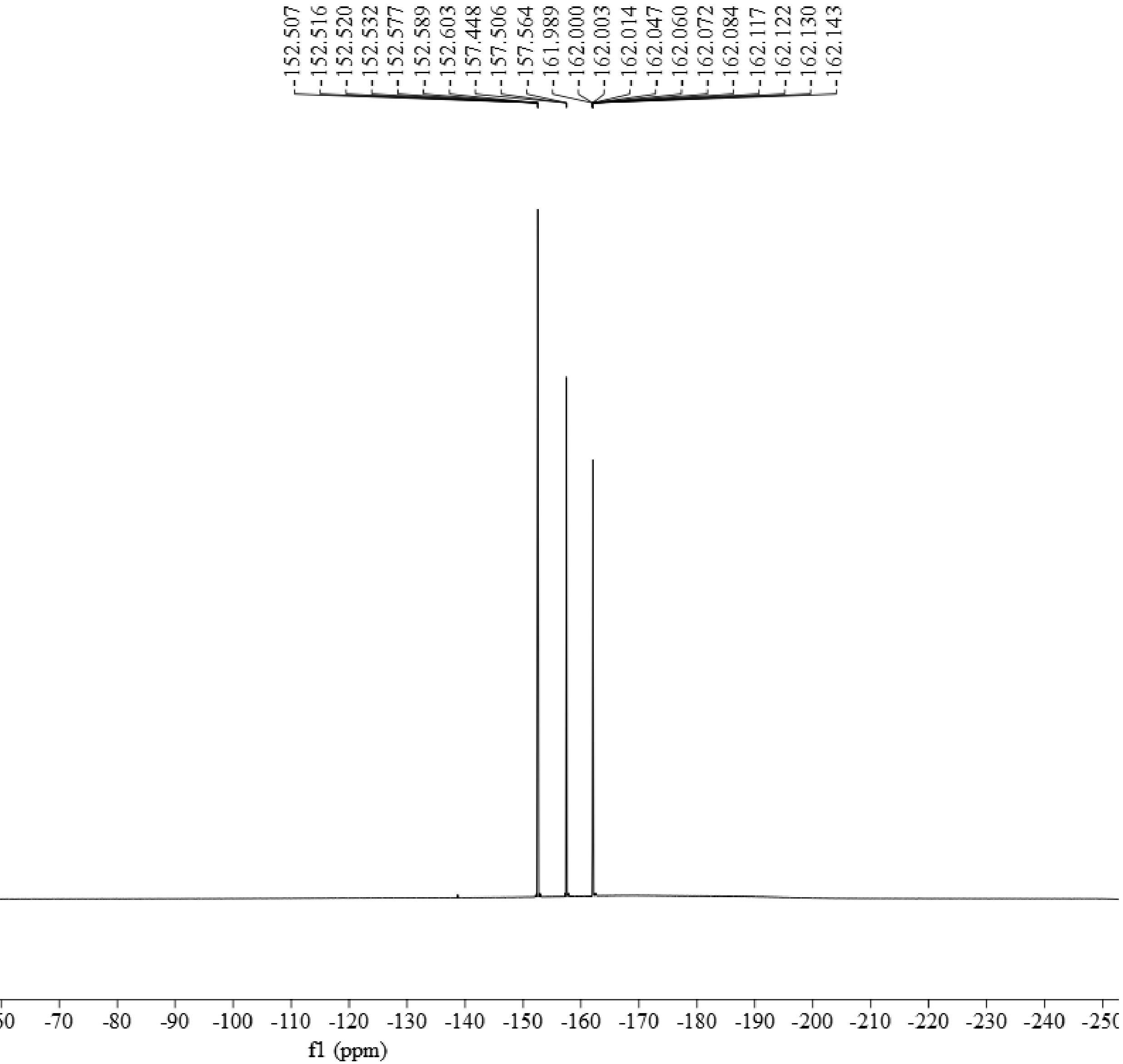

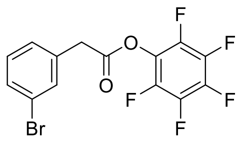

S21, ^{13}C DEPTQ, $CDCl_3$, 101 MHz

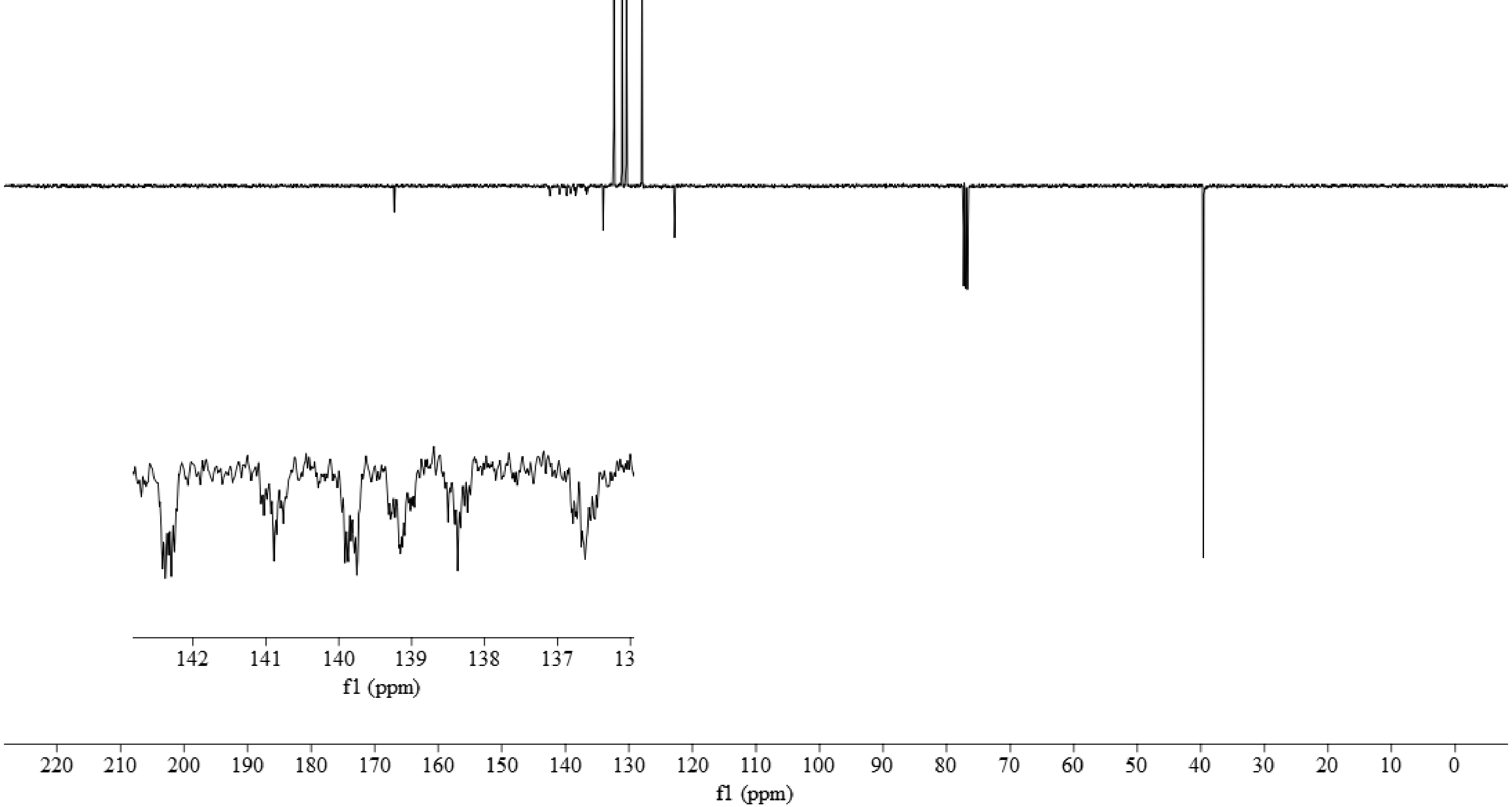

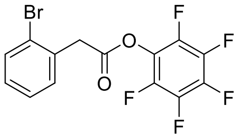

S23, ^{1}H, $CDCl_3$, 400 MHz

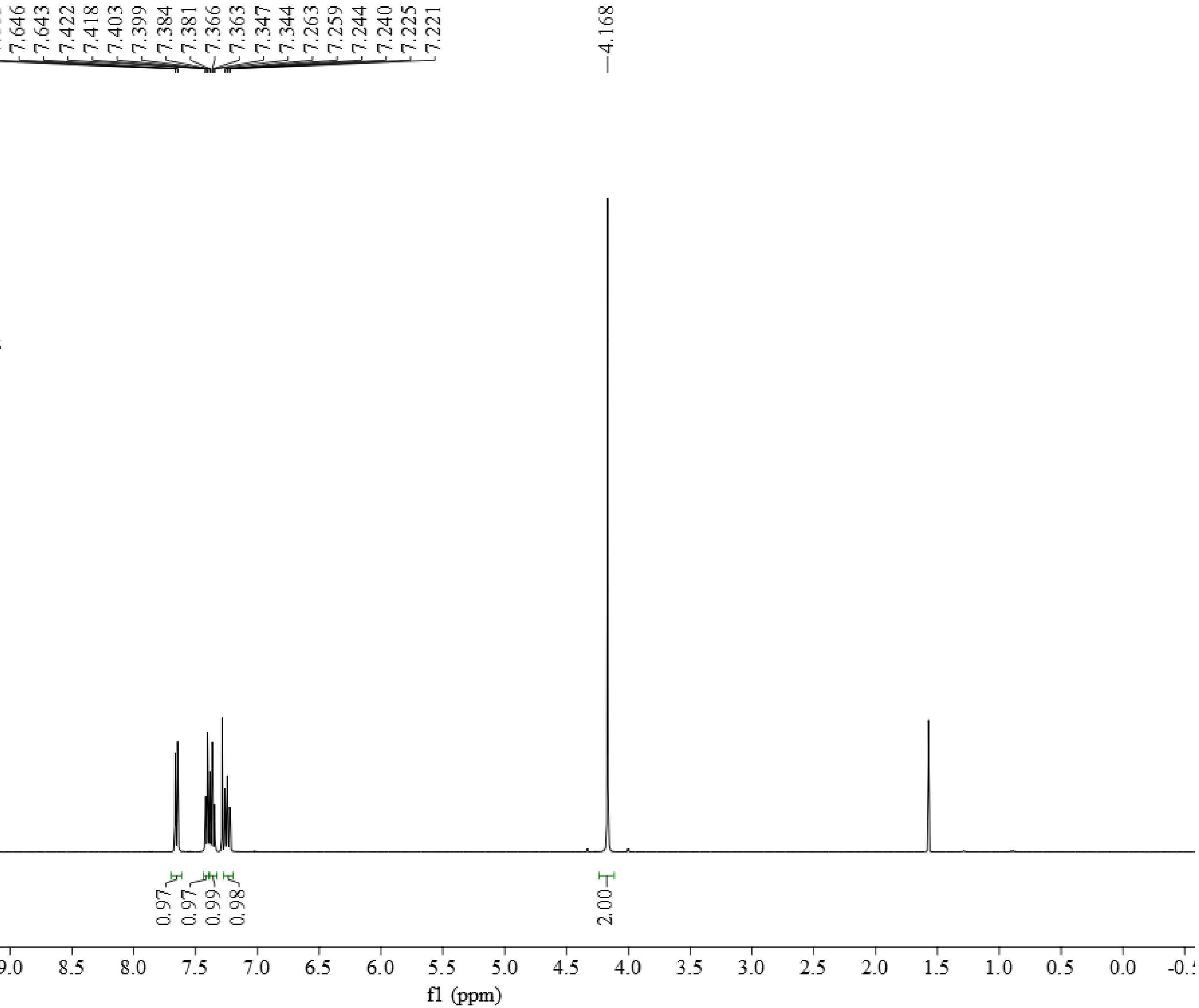

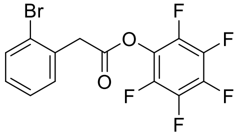

S23, ^{19}F, $CDCl_3$, 376 MHz

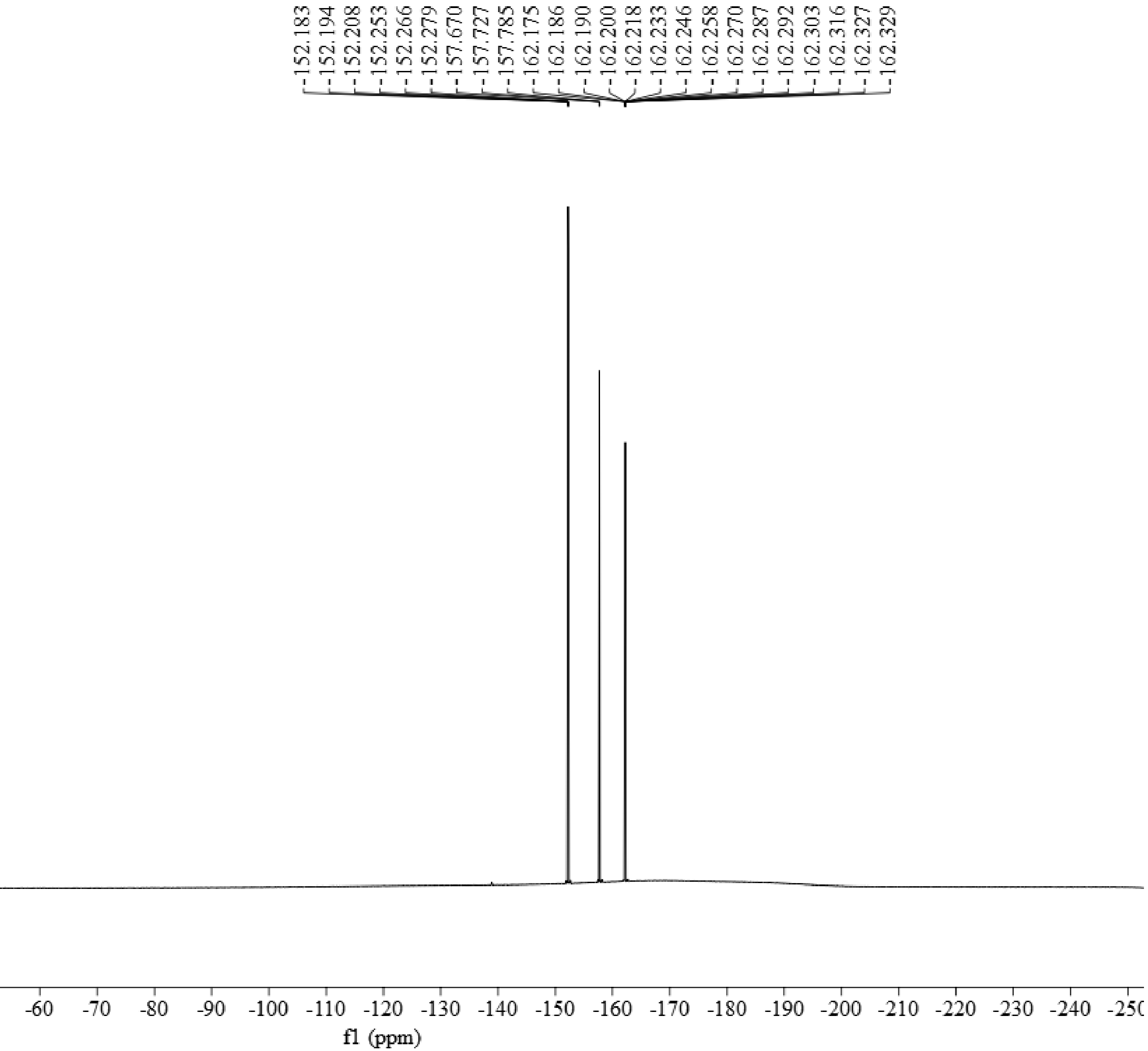

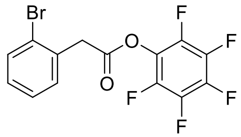

S23, ^{13}C DEPTQ, $CDCl_3$, 101 MHz

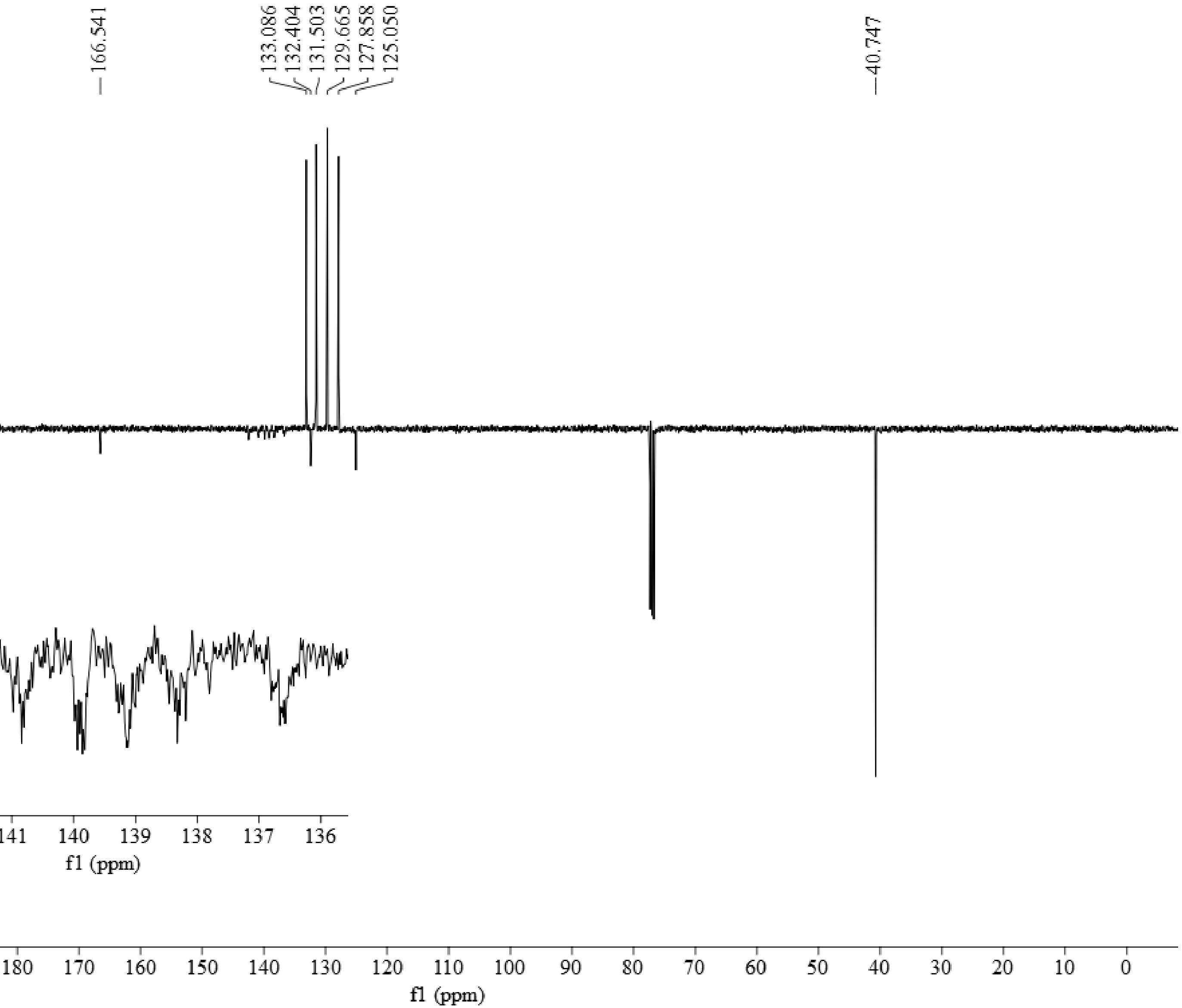

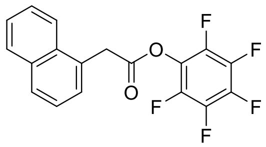

S24, ^{1}H, $CDCl_3$, 500 MHz

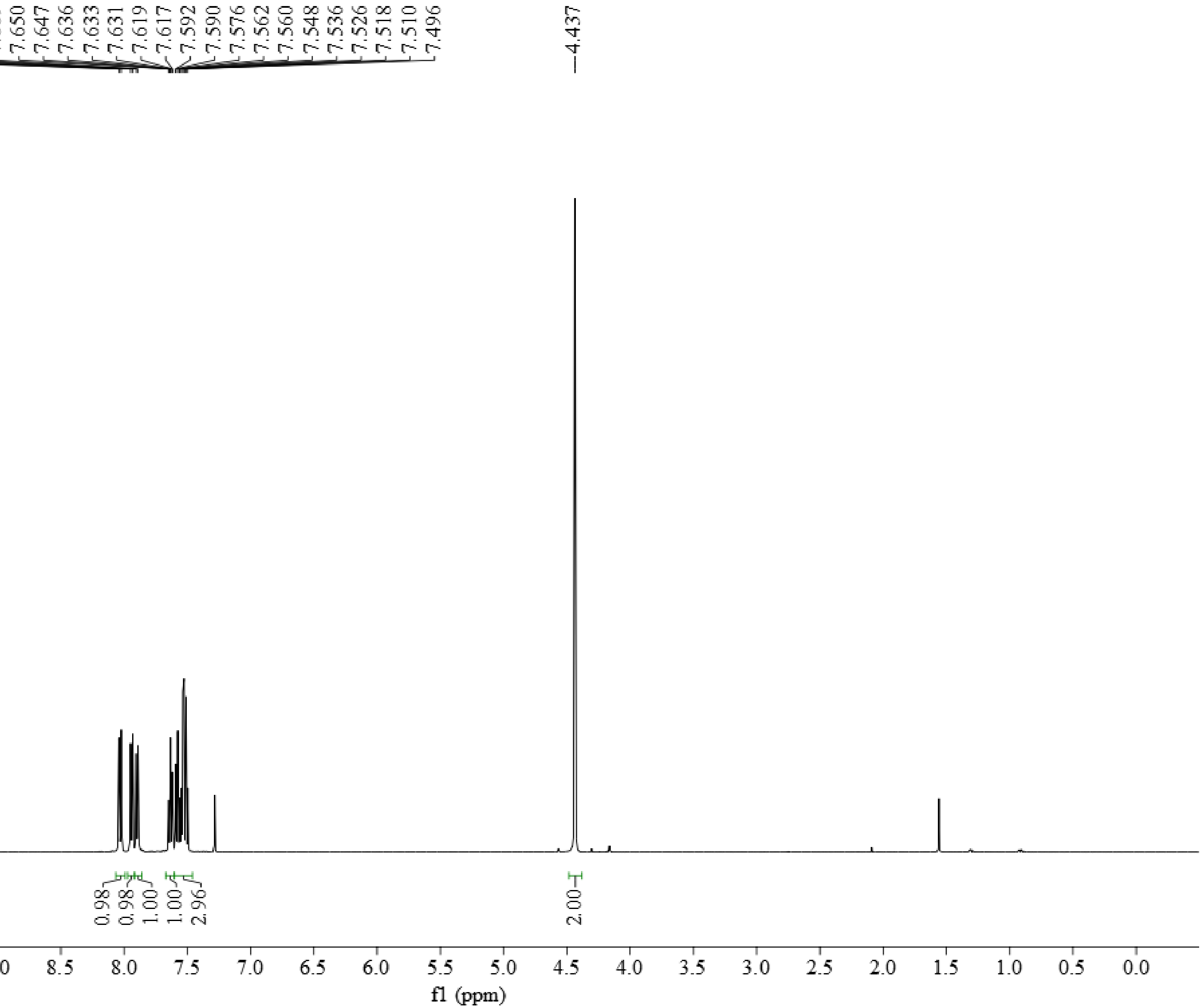

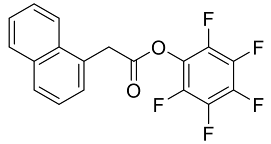

S24, ^{19}F, $CDCl_3$, 470 MHz

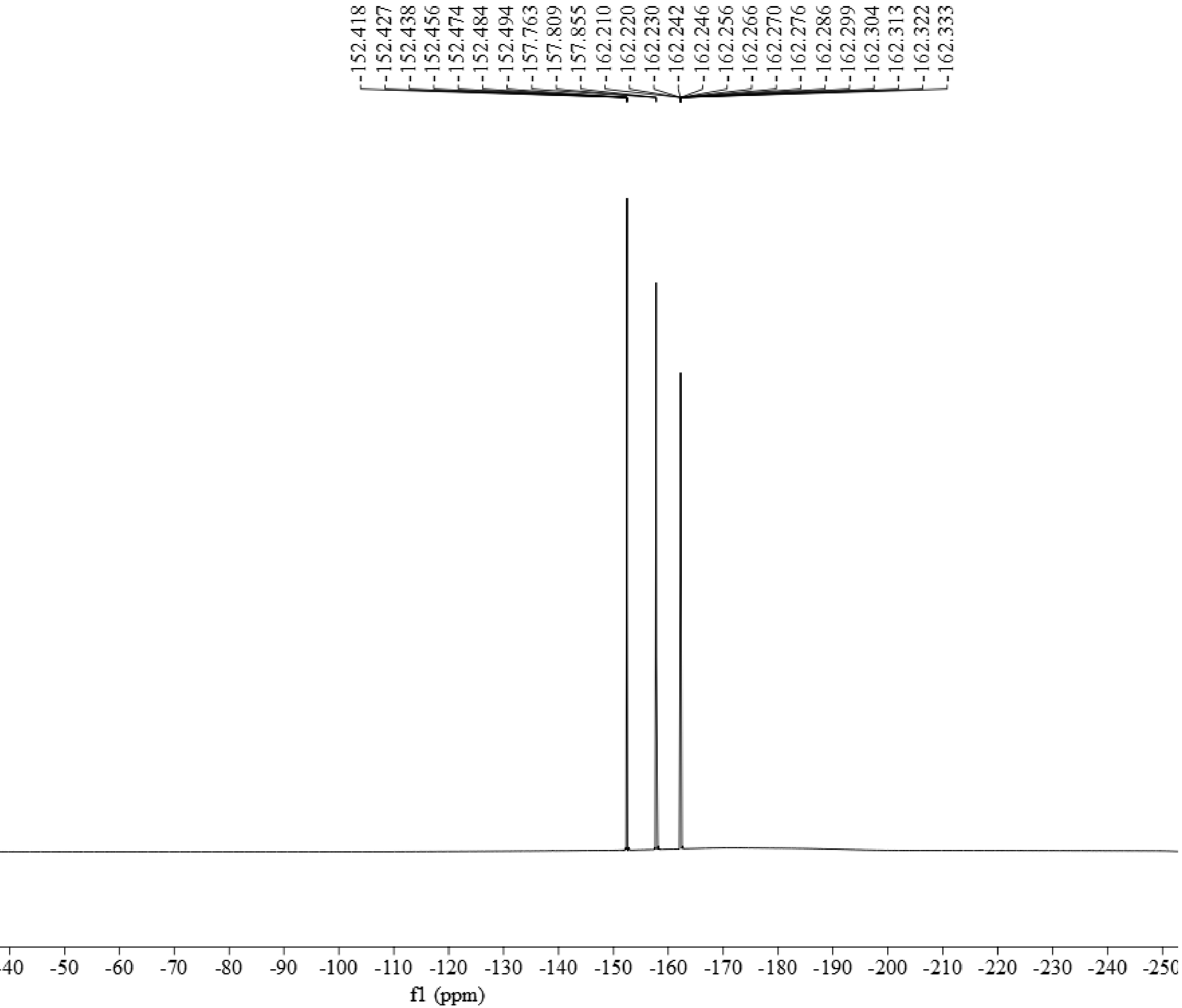

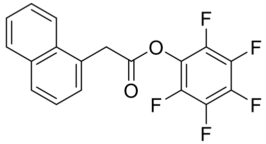

S24, ^{13}C DEPTQ, $CDCl_3$, 126 MHz

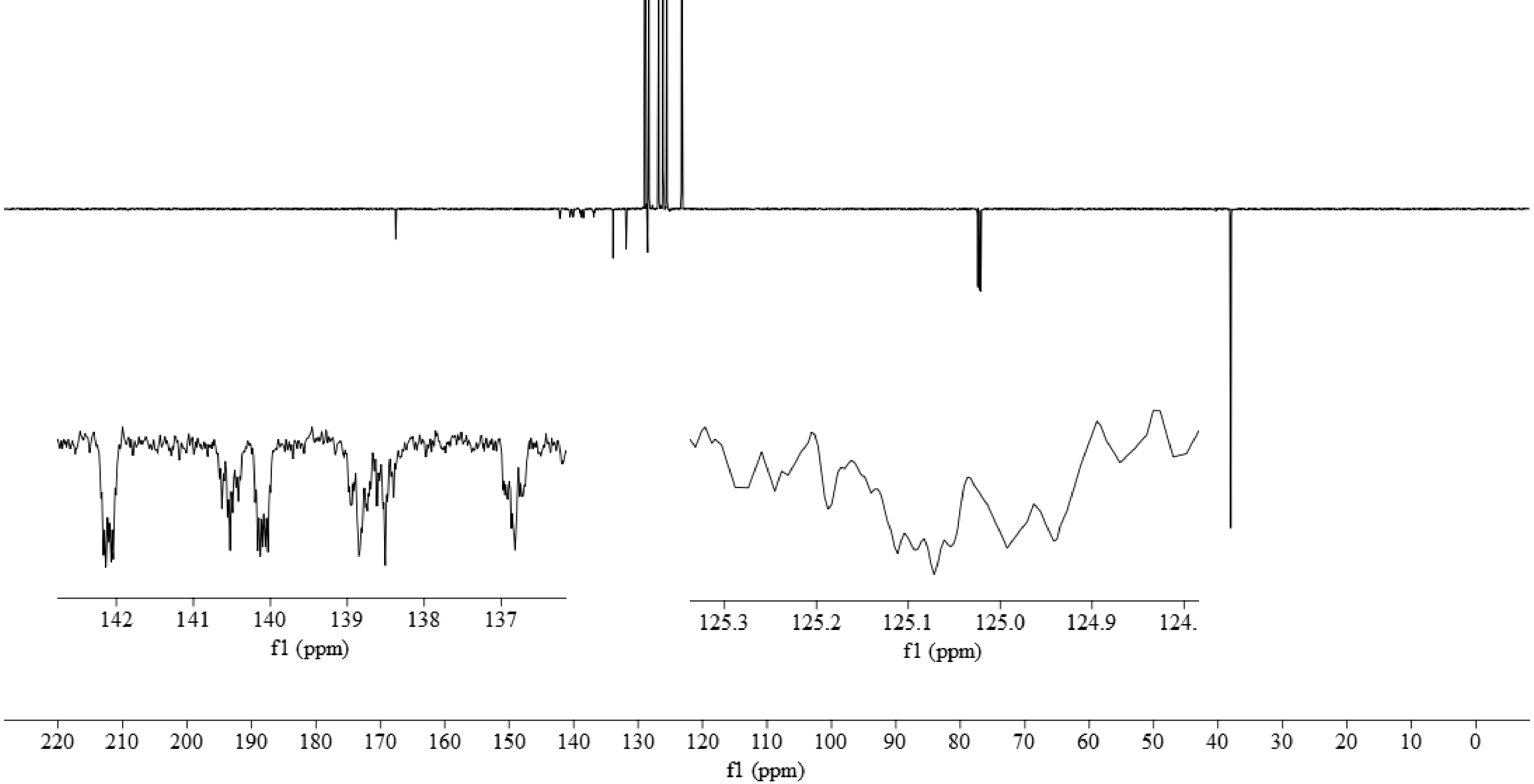

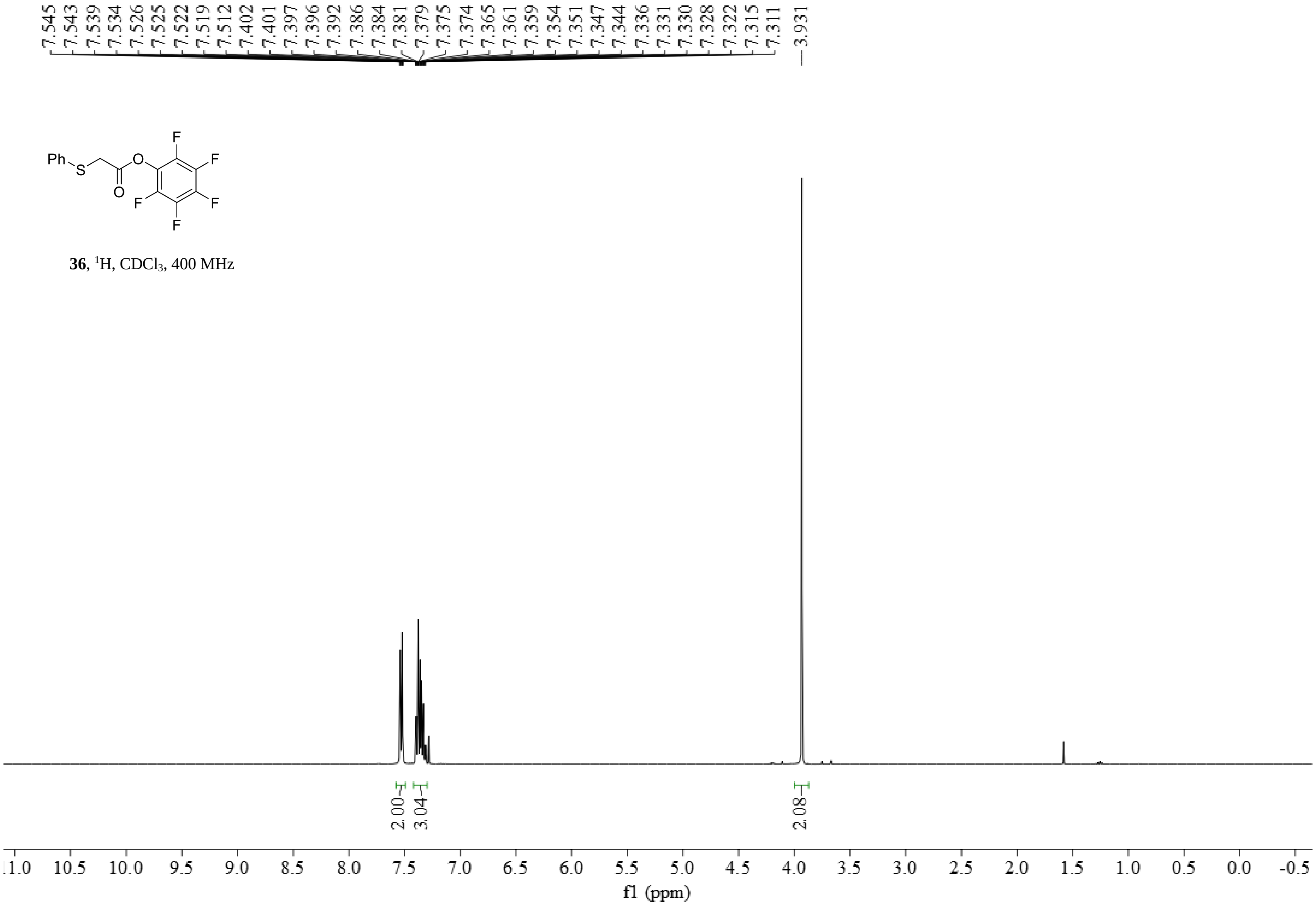

36, ^{1}H, $CDCl_3$, 400 MHz

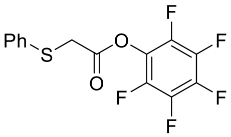

36, ^{13}C, $CDCl_3$, 101 MHz

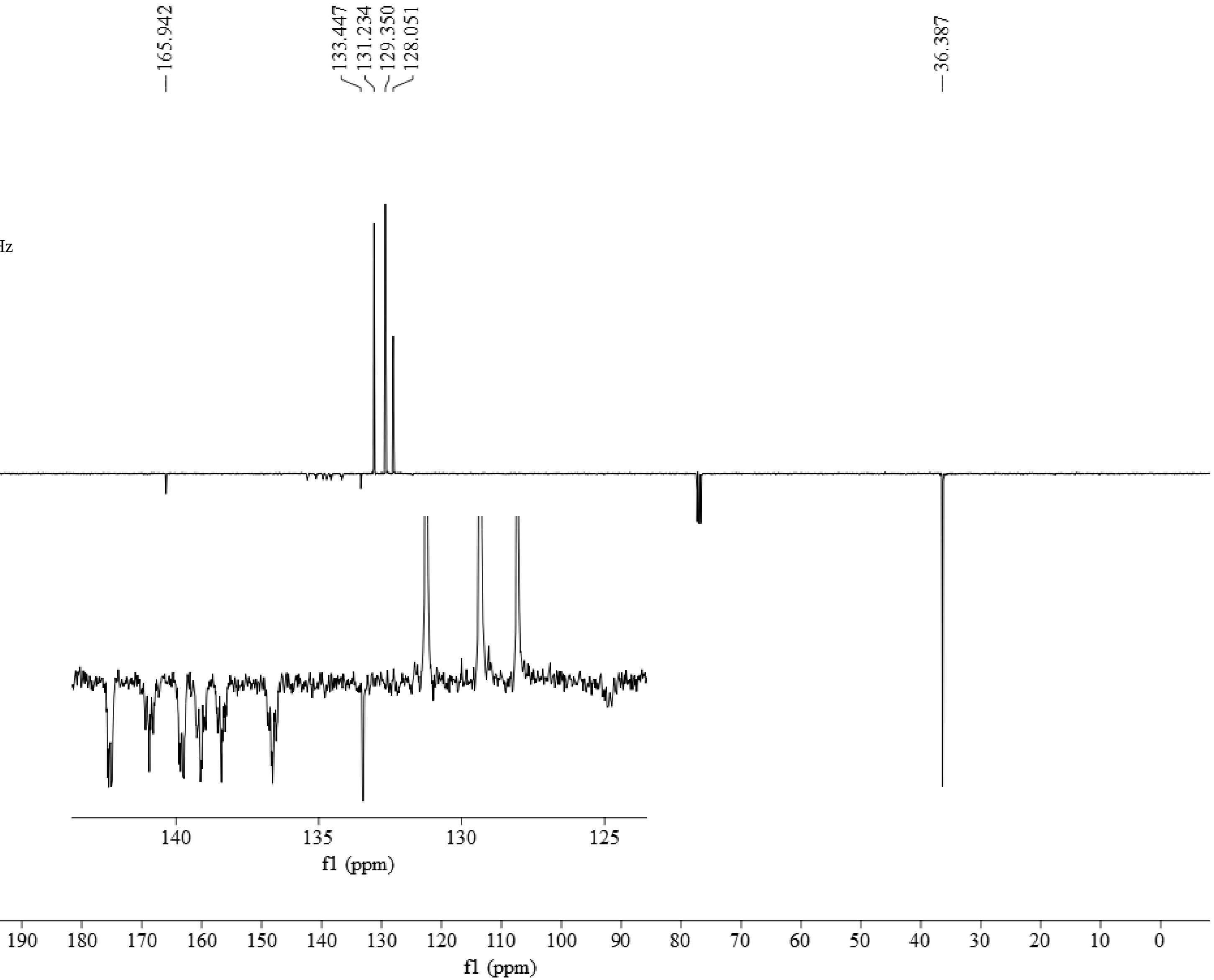

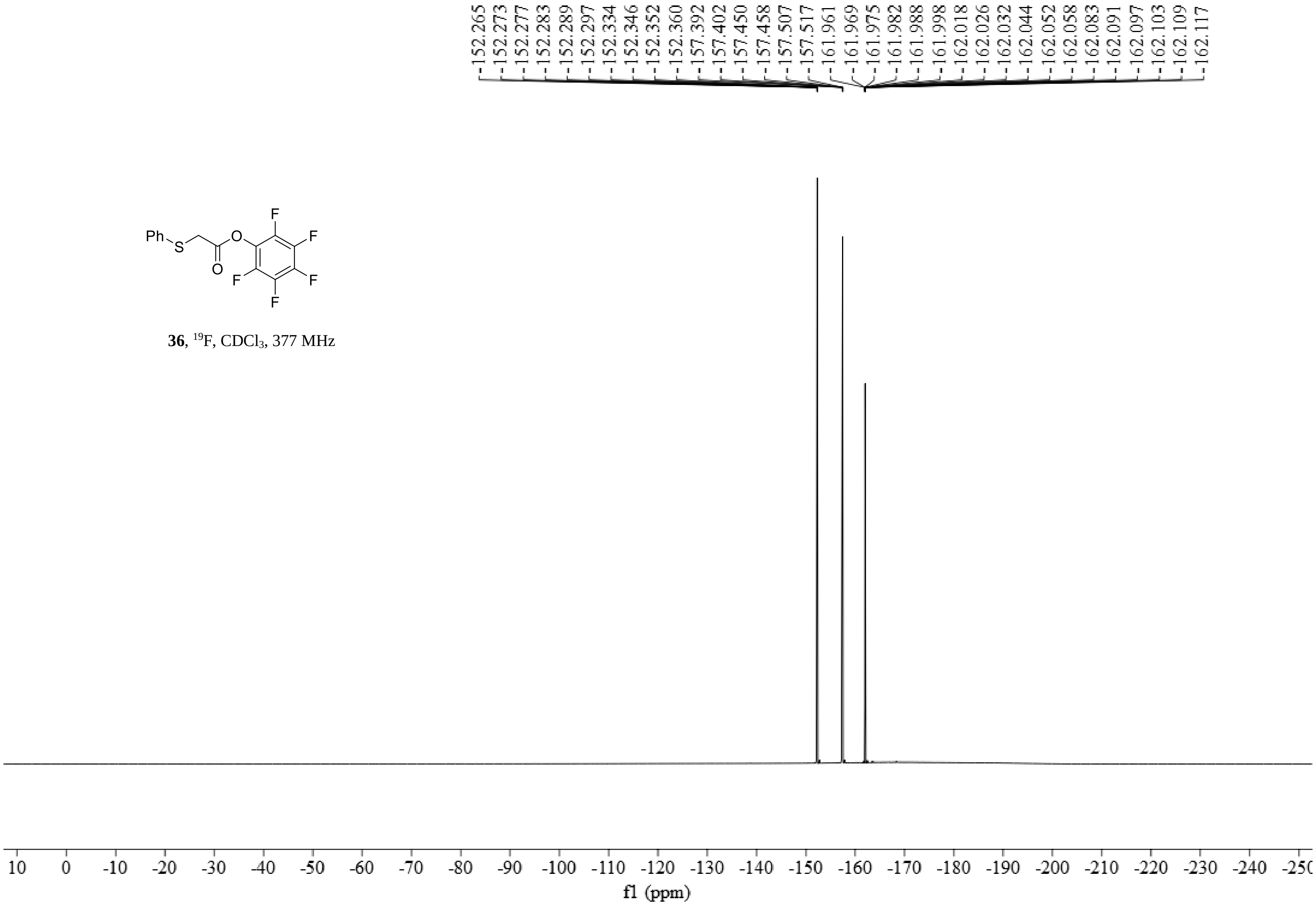
-152.265
-152.273
-152.277
-152.283
-152.289
-152.297
-152.334
-152.346
-152.352
-152.360
-157.392
-157.402
-157.450
-157.458
-157.507
-157.517
-161.961
-161.969
-161.975
-161.982
-161.988
-161.998
-162.018
-162.026
-162.032
-162.044
-162.052
-162.058
-162.083
-162.091
-162.097
-162.103
-162.109
-162.117
Ph
S
O
O
F
F
F
F
F
36, ^{19}F, $CDCl_3$, 377 MHz
10 0 -10 -20 -30 -40 -50 -60 -70 -80 -90 -100 -110 -120 -130 -140 -150 -160 -170 -180 -190 -200 -210 -220 -230 -240 -250
f1 (ppm)

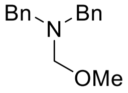

5, ^{1}H, $CDCl_3$, 400 MHz

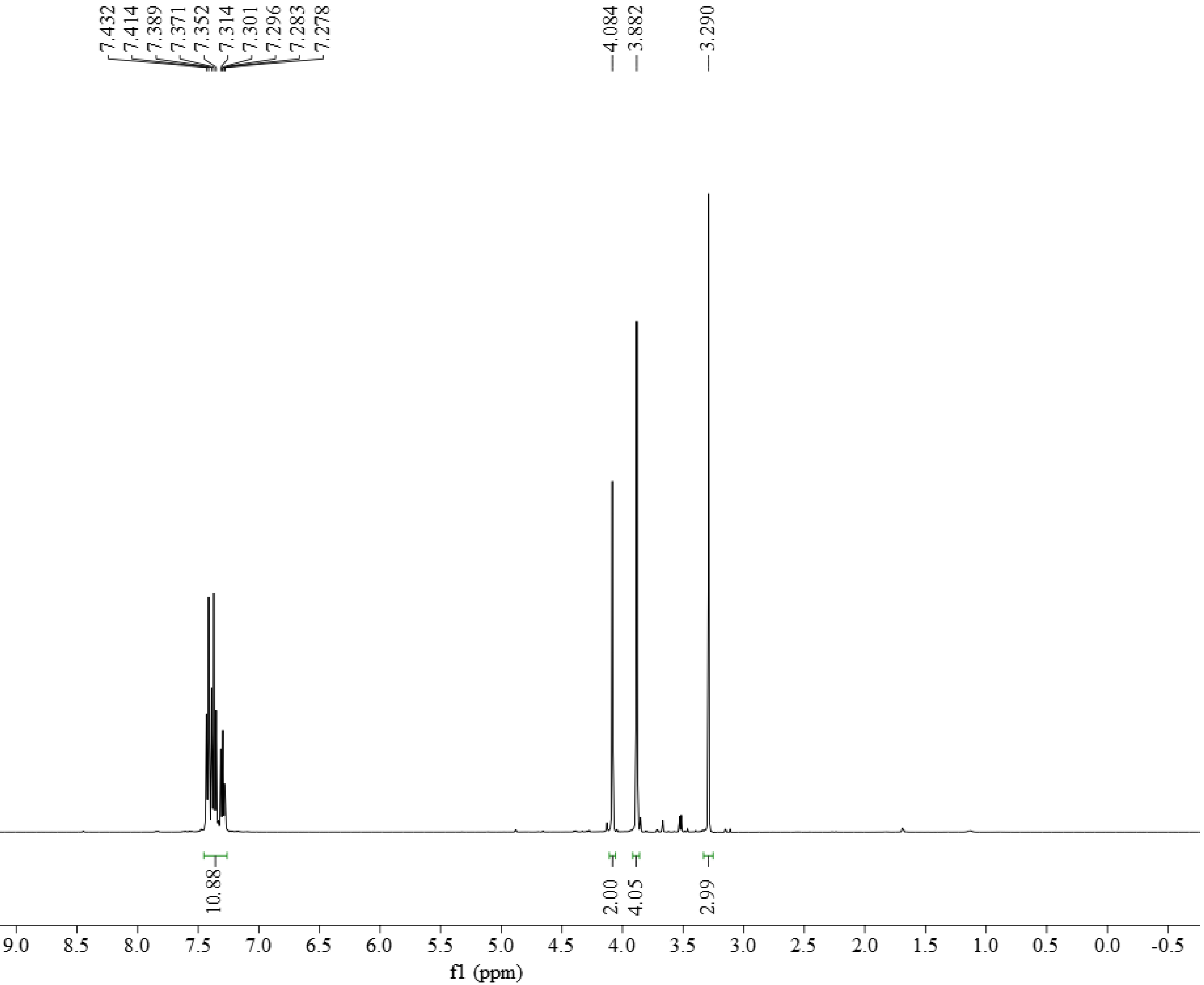

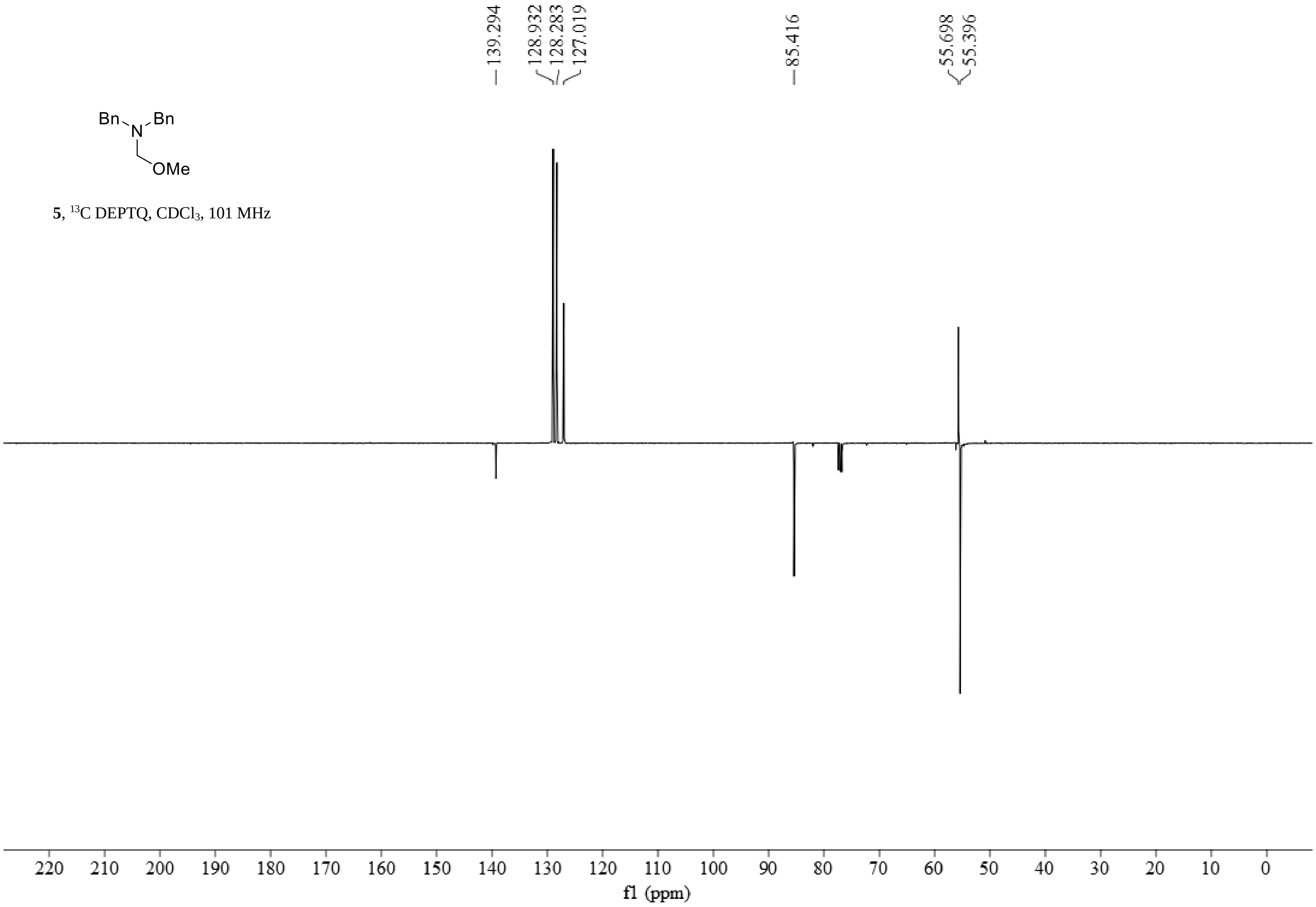

5, ^{13}C DEPTQ, $CDCl_3$, 101 MHz

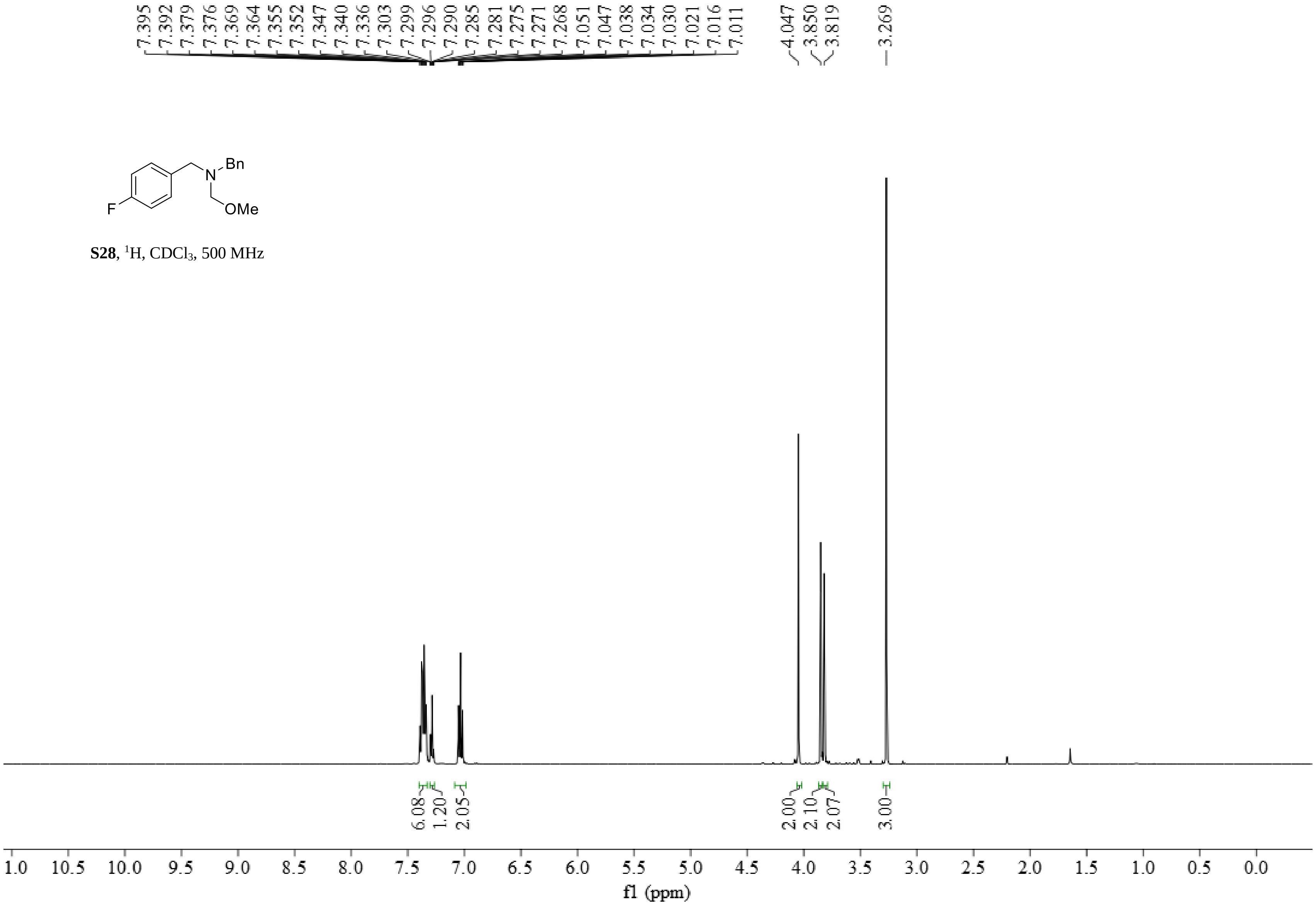

S28, ^{1}H, $CDCl_3$, 500 MHz

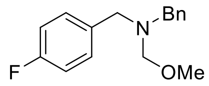

S28, ^{19}F, $CDCl_3$, 470 MHz

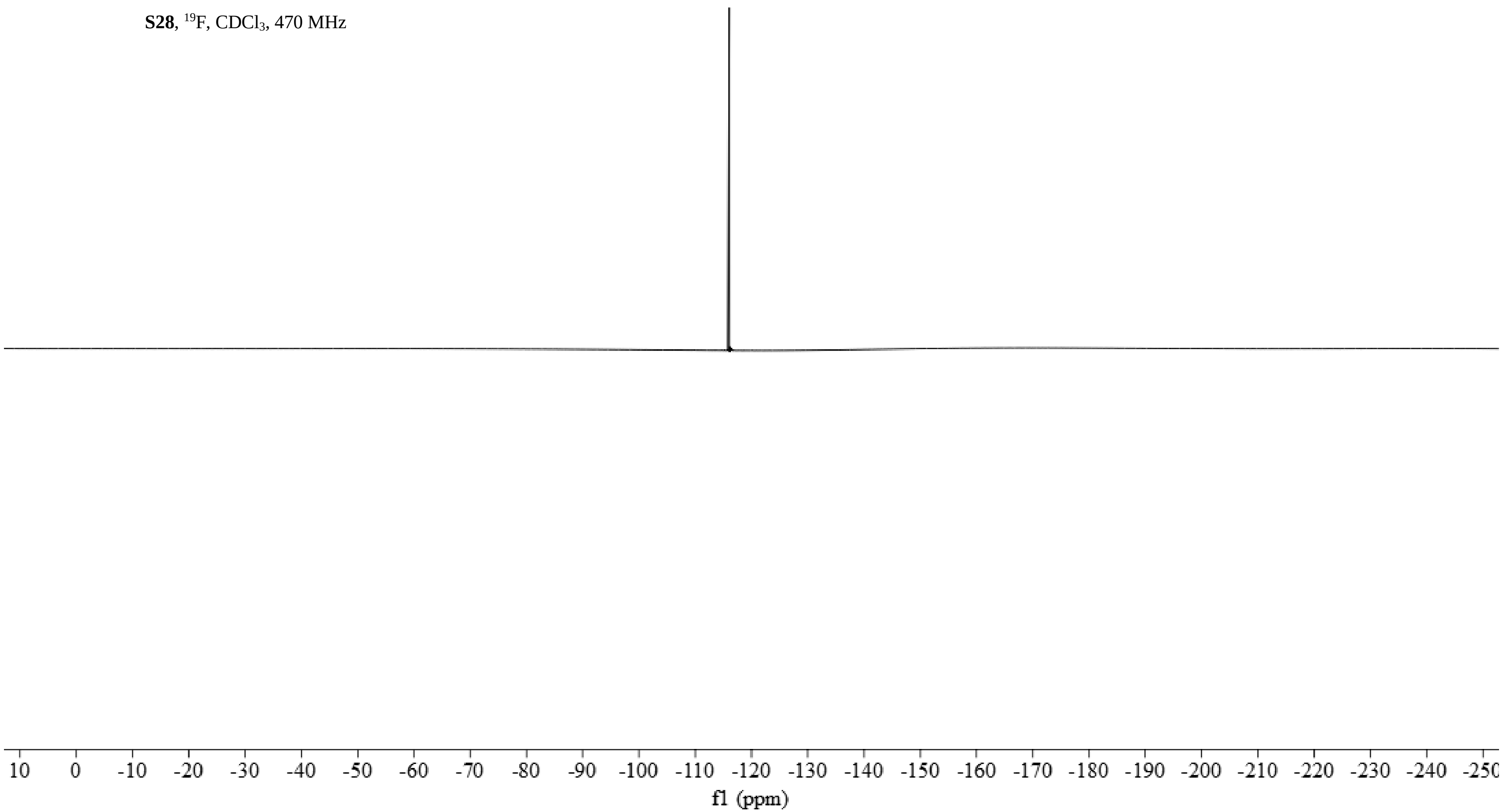

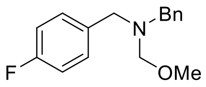

S28, ^{13}C DEPTQ, $CDCl_3$, 126 MHz

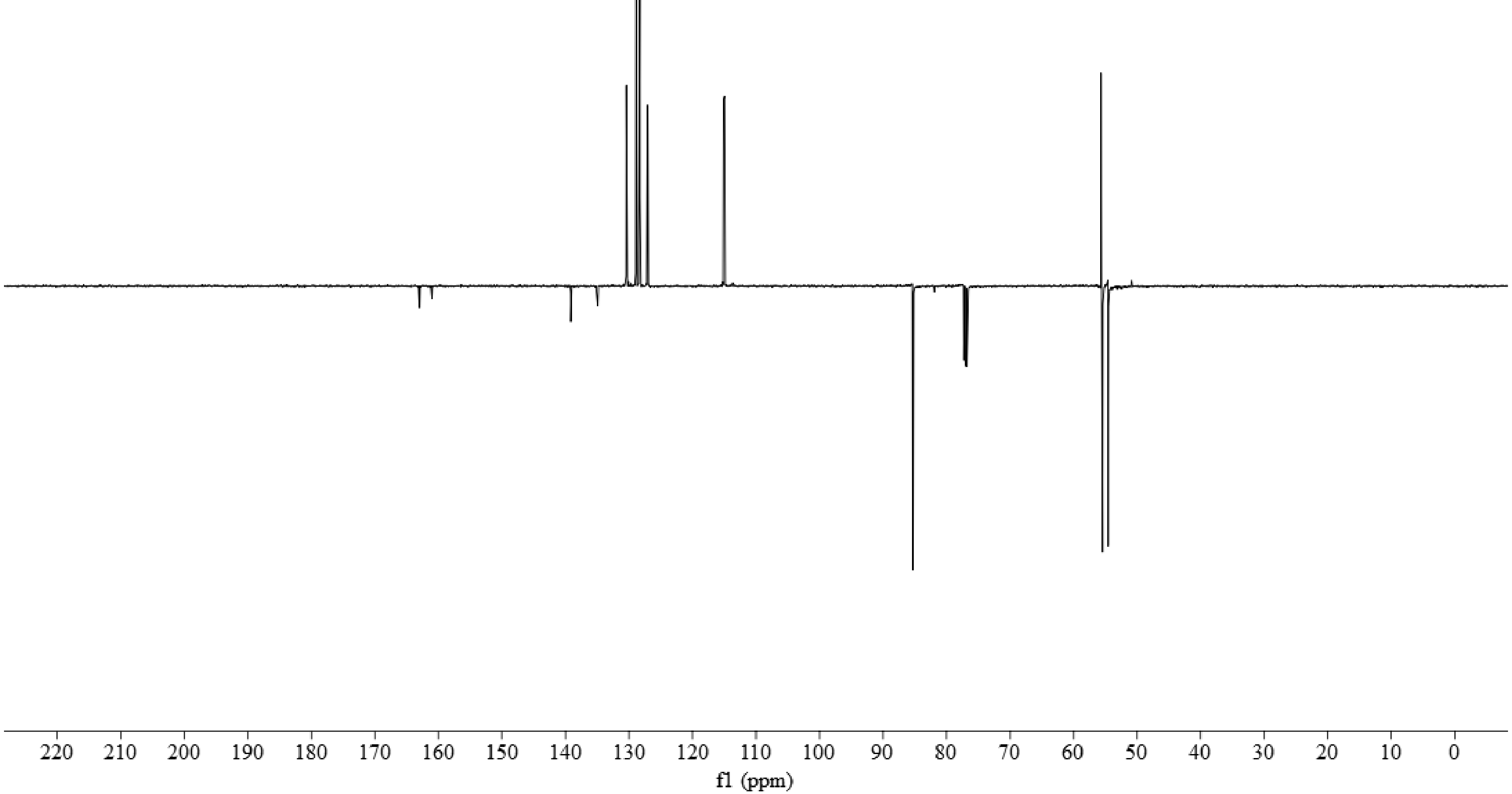

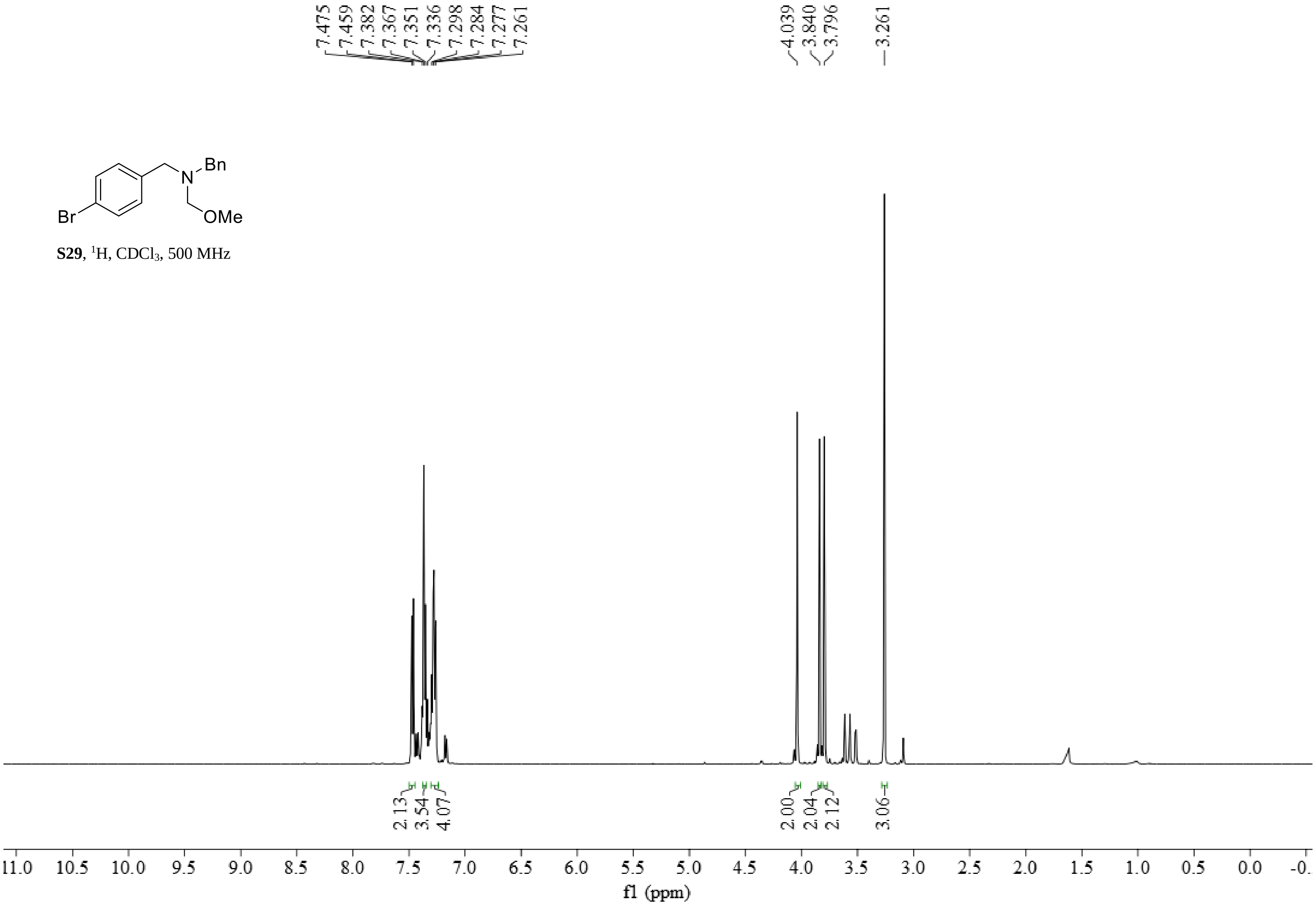

S29, ^{1}H, $CDCl_3$, 500 MHz

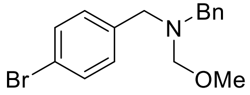

S29, ^{13}C DEPTQ, $CDCl_3$, 126 MHz

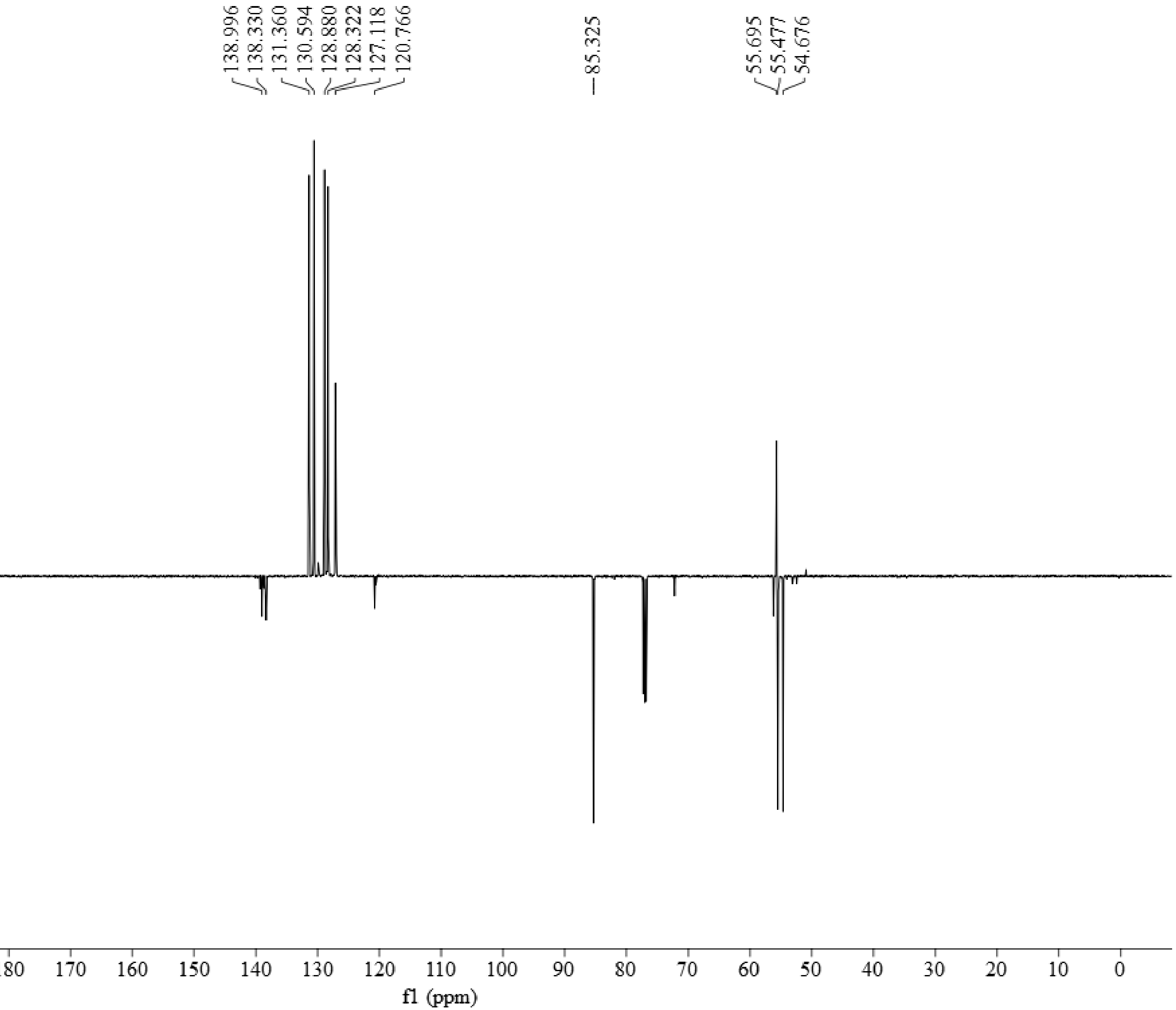

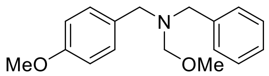

S30, ^{1}H, $CDCl_3$, 500 MHz

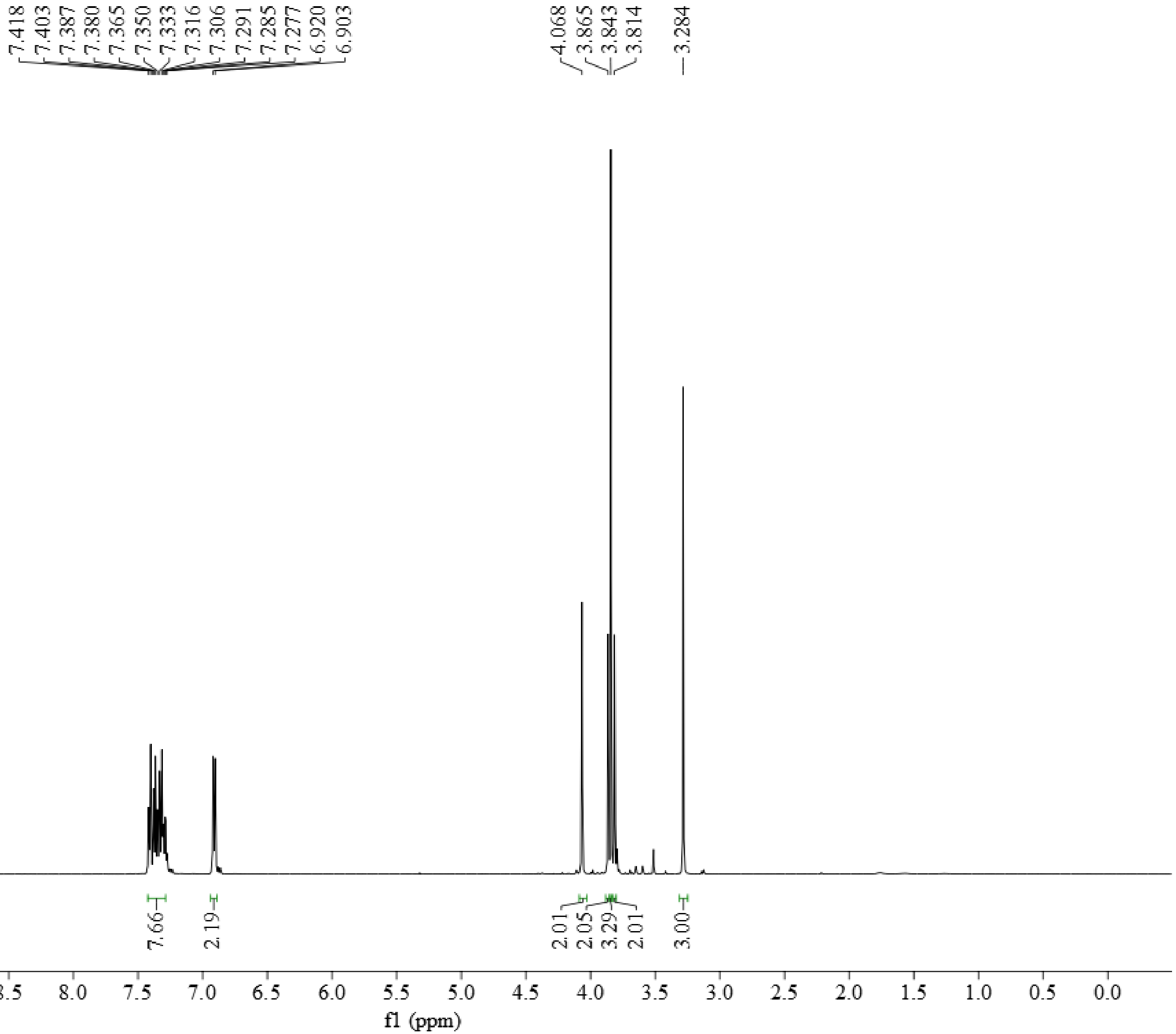

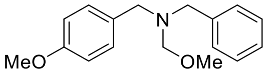

S30, ^{13}C DEPTQ, $CDCl_3$, 126 MHz

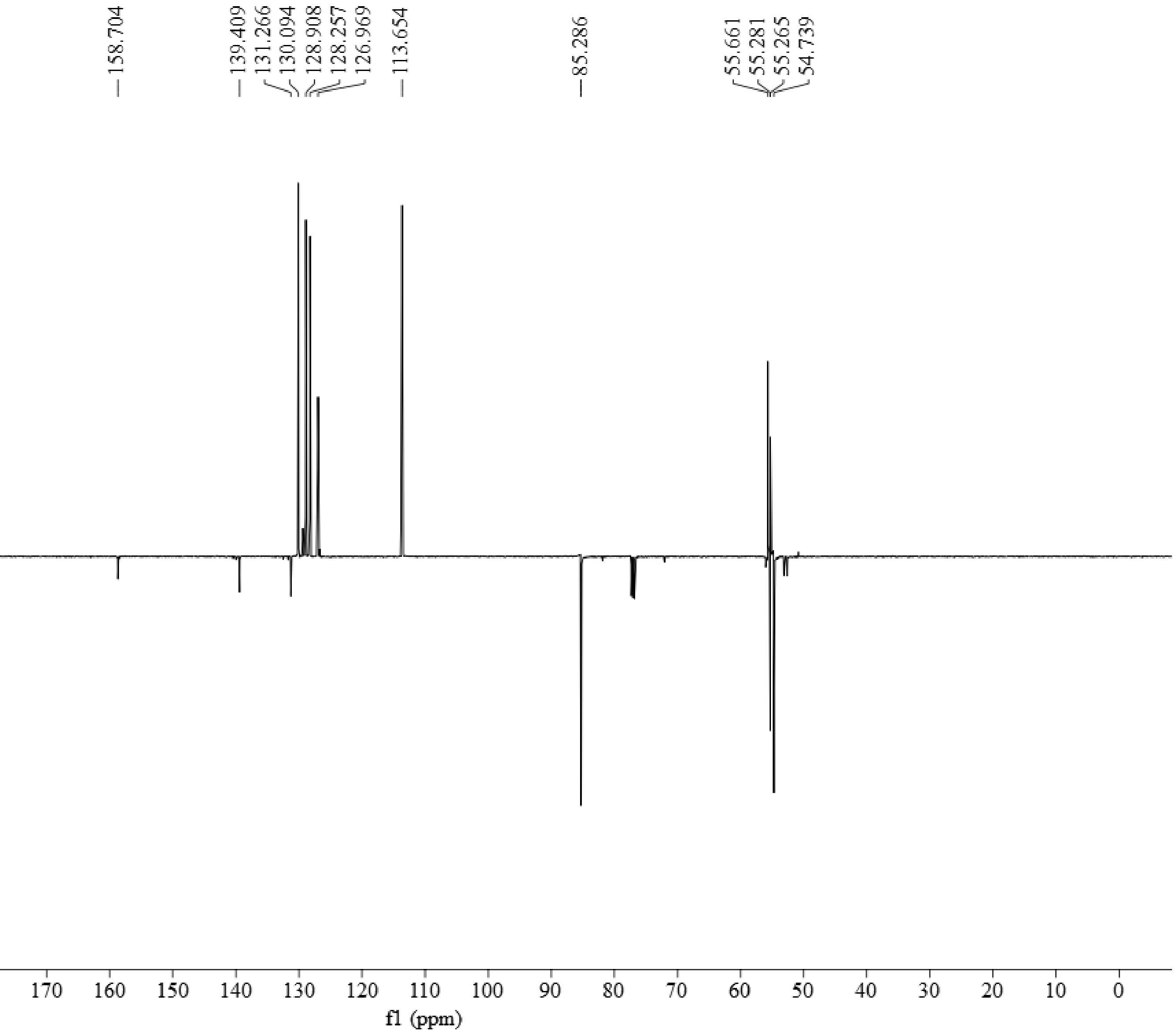

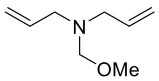

S31, ^{1}H, $CDCl_3$, 400 MHz

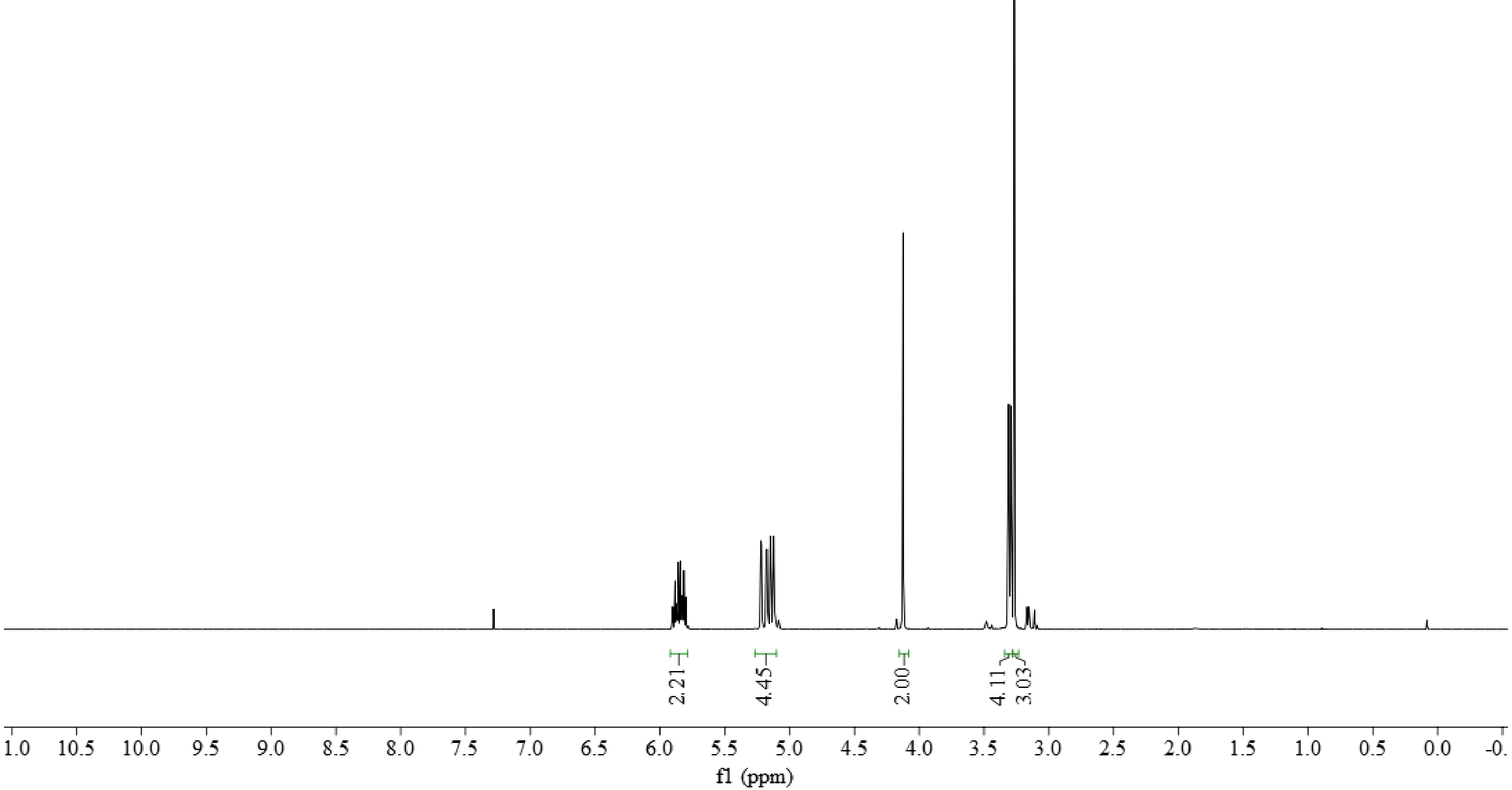

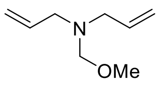

S31, ^{13}C DEPTQ, $CDCl_3$, 126 MHz

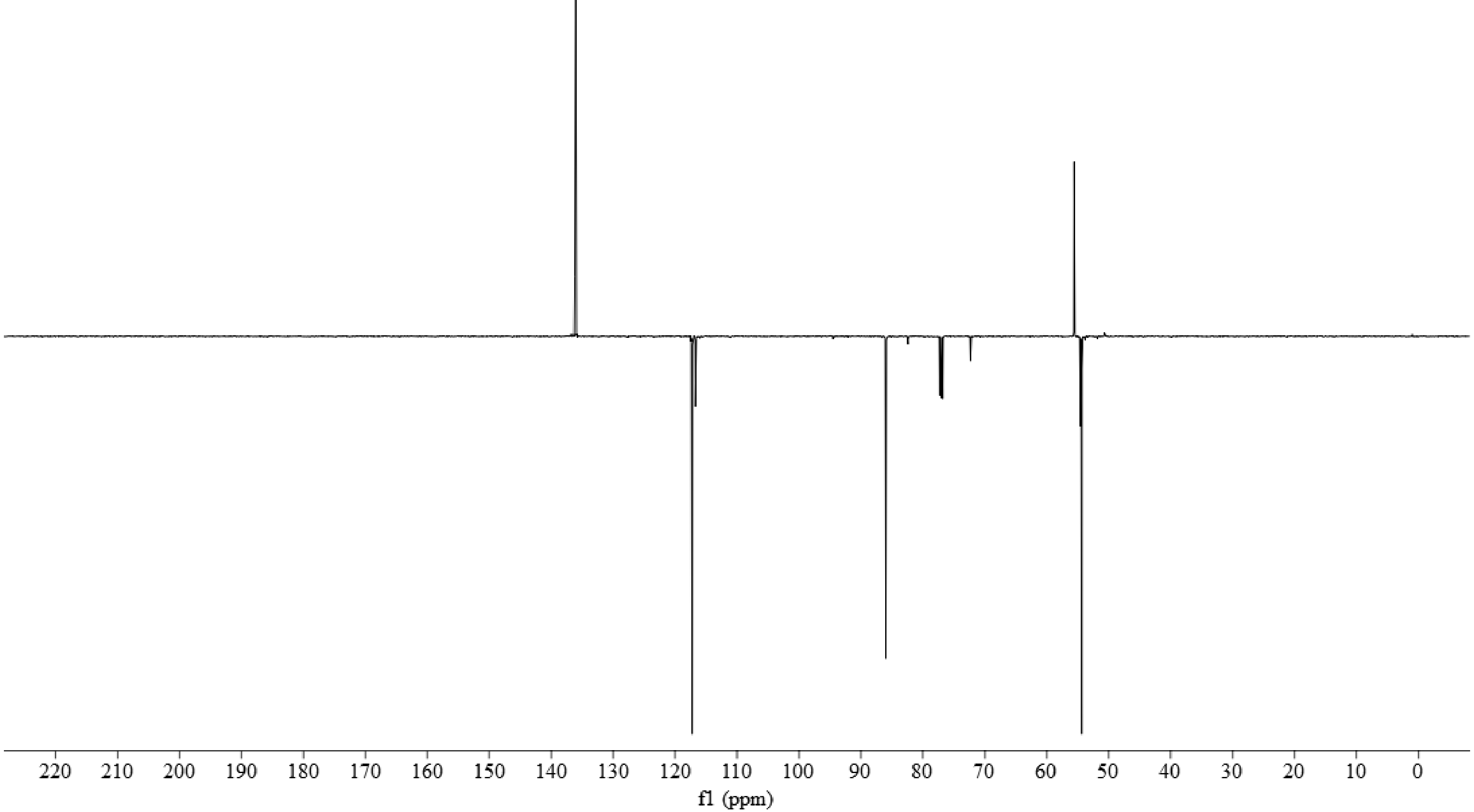

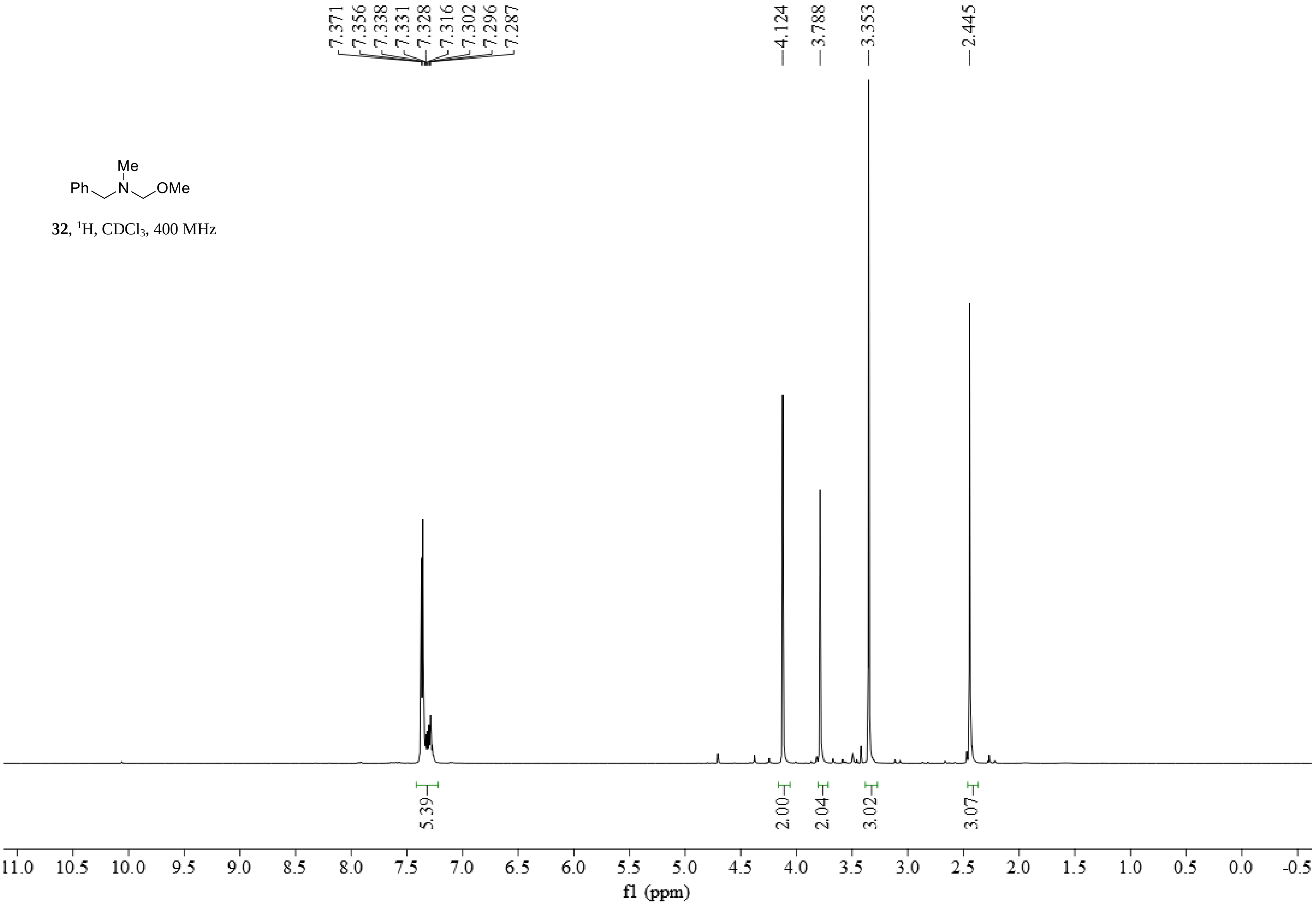
Me
Ph
N
OMe
32, 1H, CDCl3, 400 MHz
7.371
7.356
7.338
7.331
7.328
7.316
7.302
7.296
7.287
4.124
3.788
3.353
2.445
5.39
2.00
2.04
3.02
3.07
11.0 10.5 10.0 9.5 9.0 8.5 8.0 7.5 7.0 6.5 6.0 5.5 5.0 4.5 4.0 3.5 3.0 2.5 2.0 1.5 1.0 0.5 0.0 -0.5
f1 (ppm)

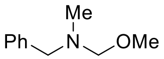

32, ^{13}C DEPTQ, $CDCl_3$, 400 MHz

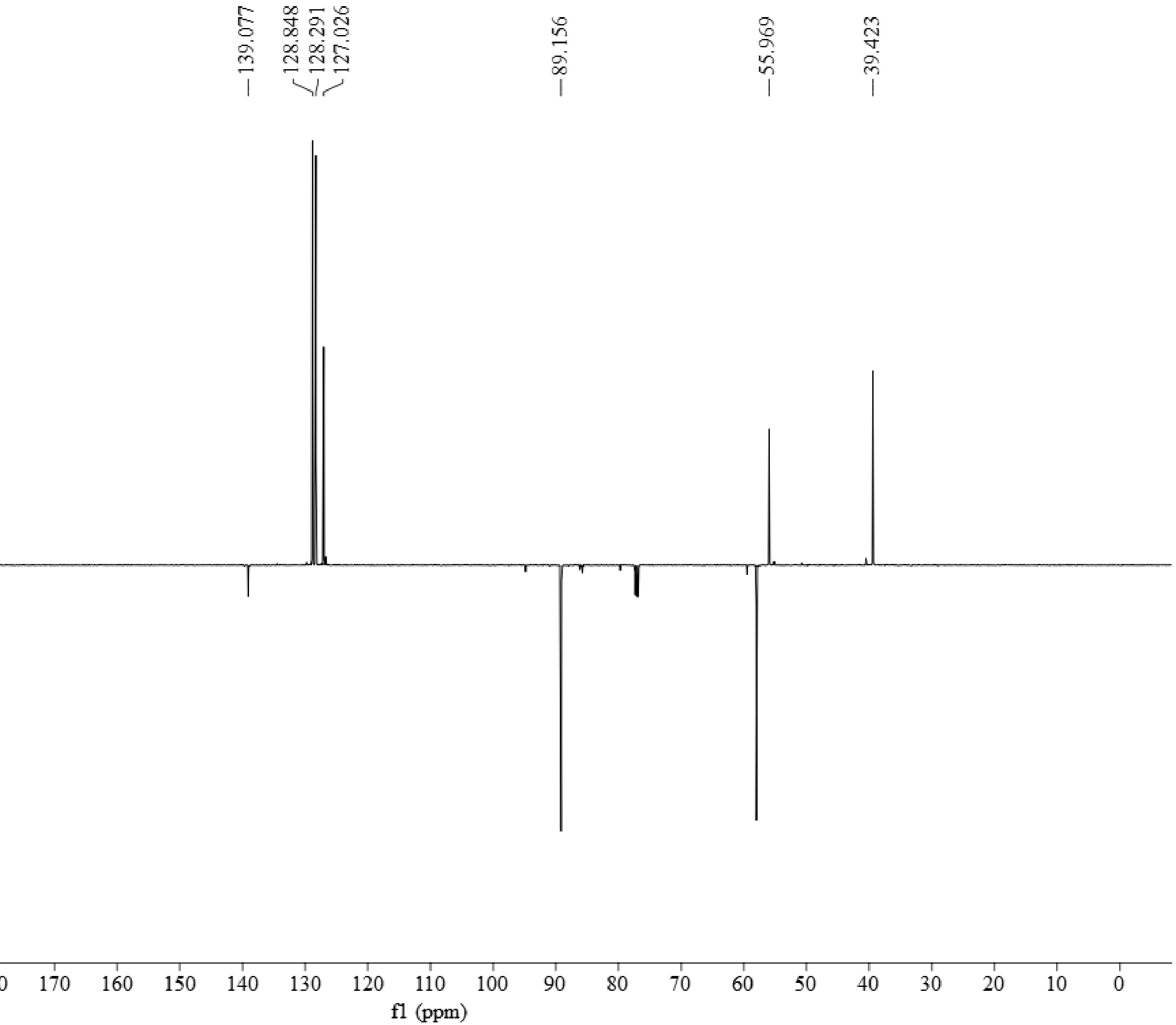

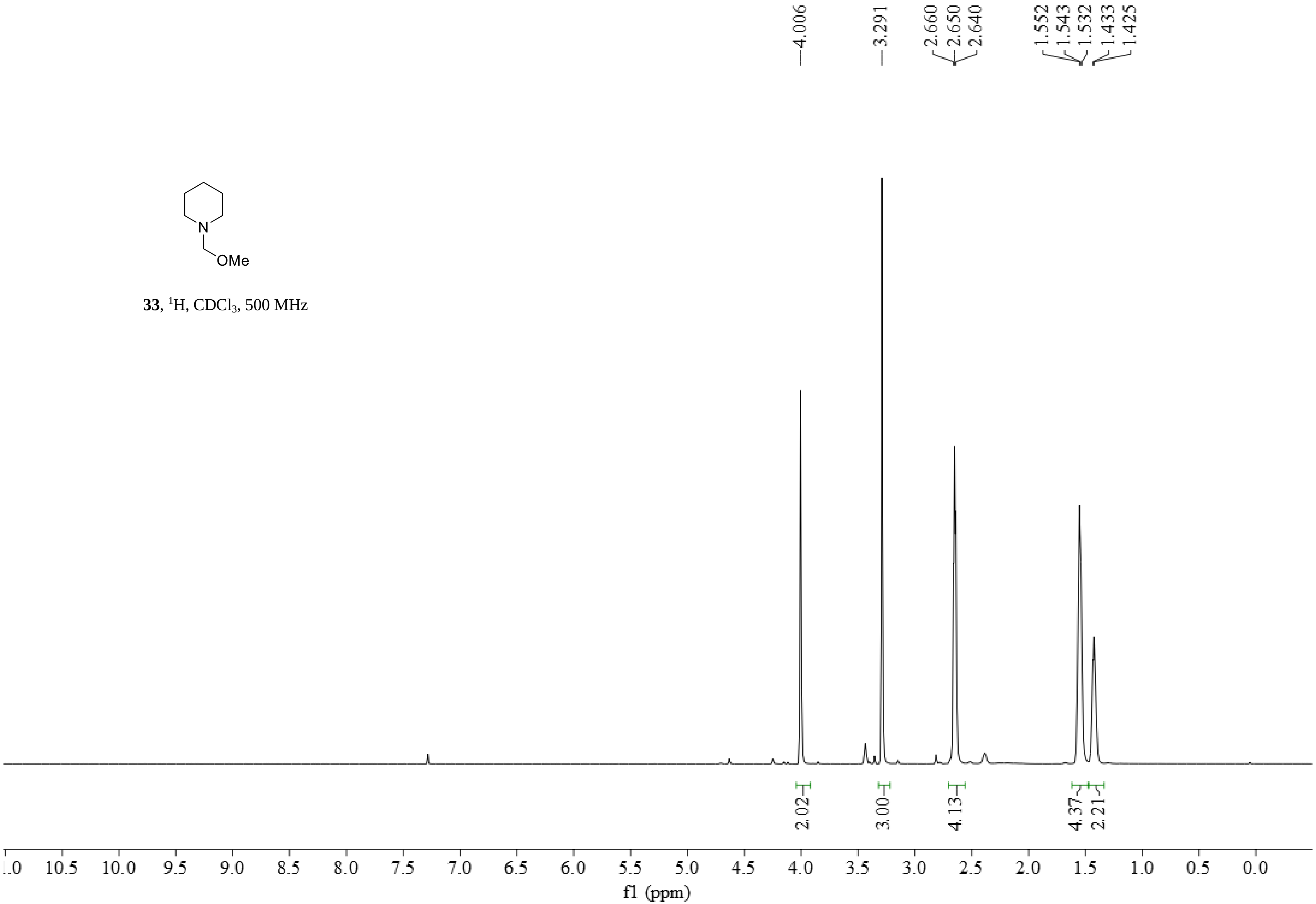

33, ^{1}H, $CDCl_3$, 500 MHz

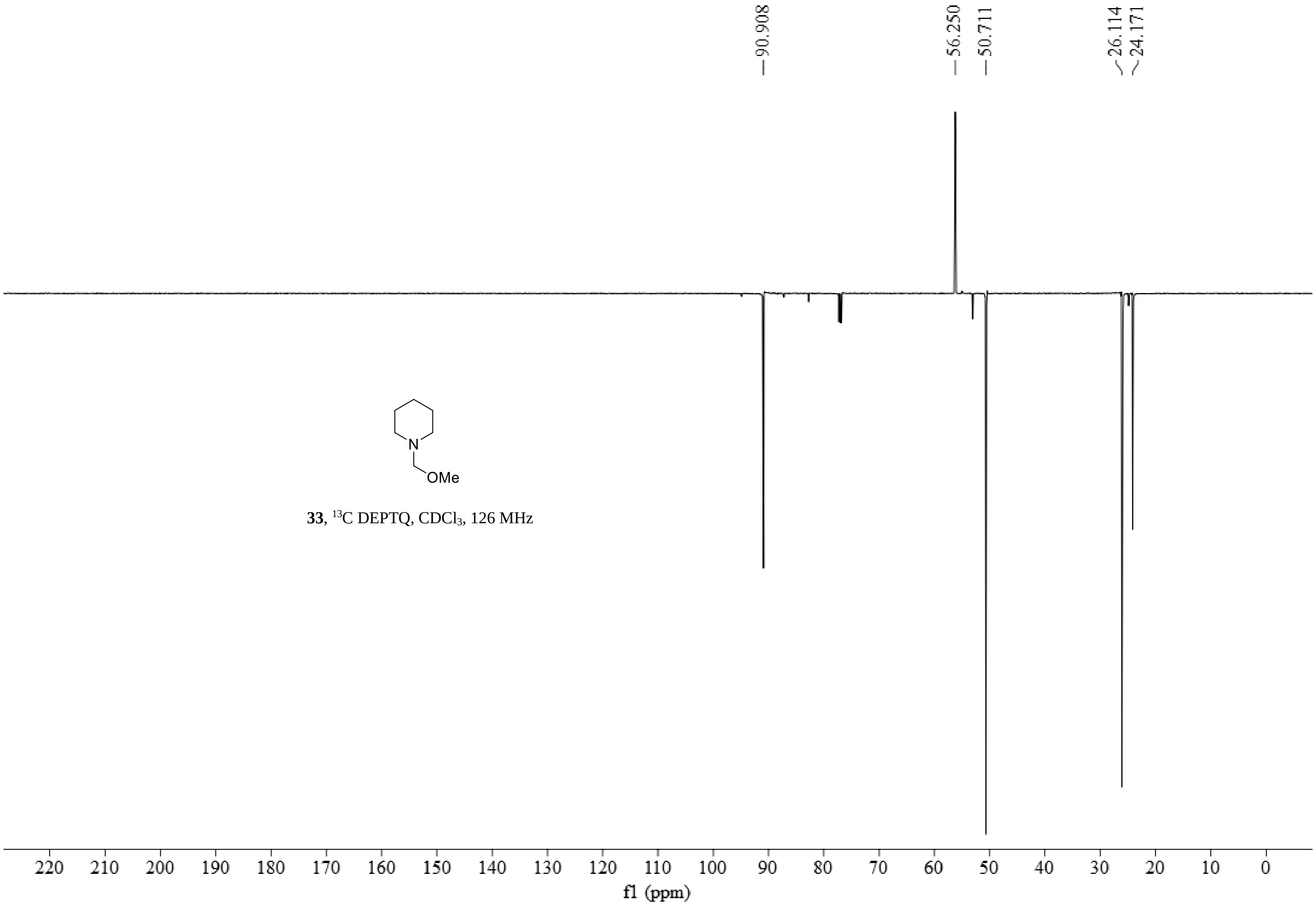

33, ^{13}C DEPTQ, $CDCl_3$, 126 MHz

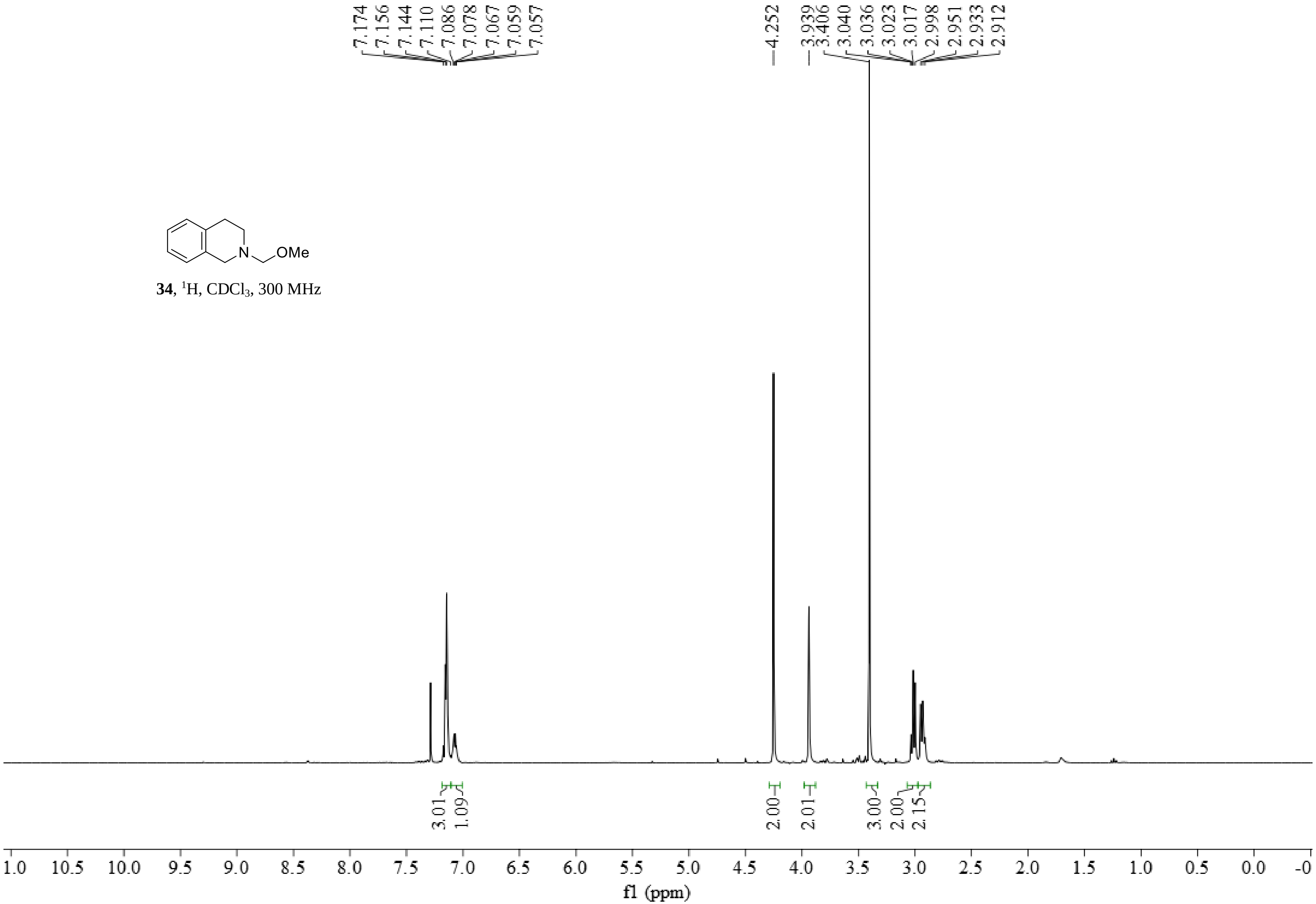

34, ^{1}H, $CDCl_3$, 300 MHz

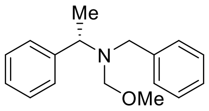

50, ^{1}H, $CDCl_3$, 400 MHz

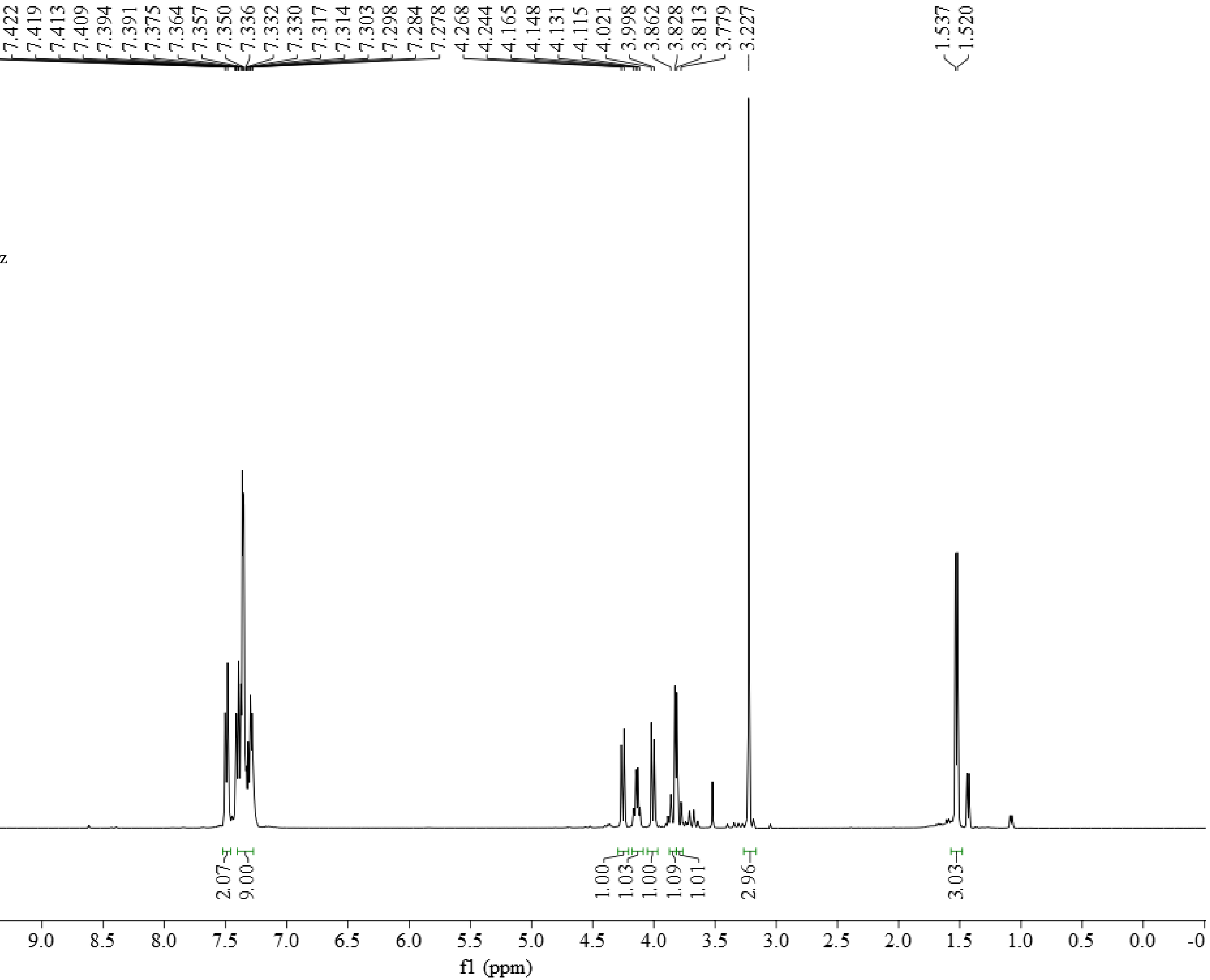

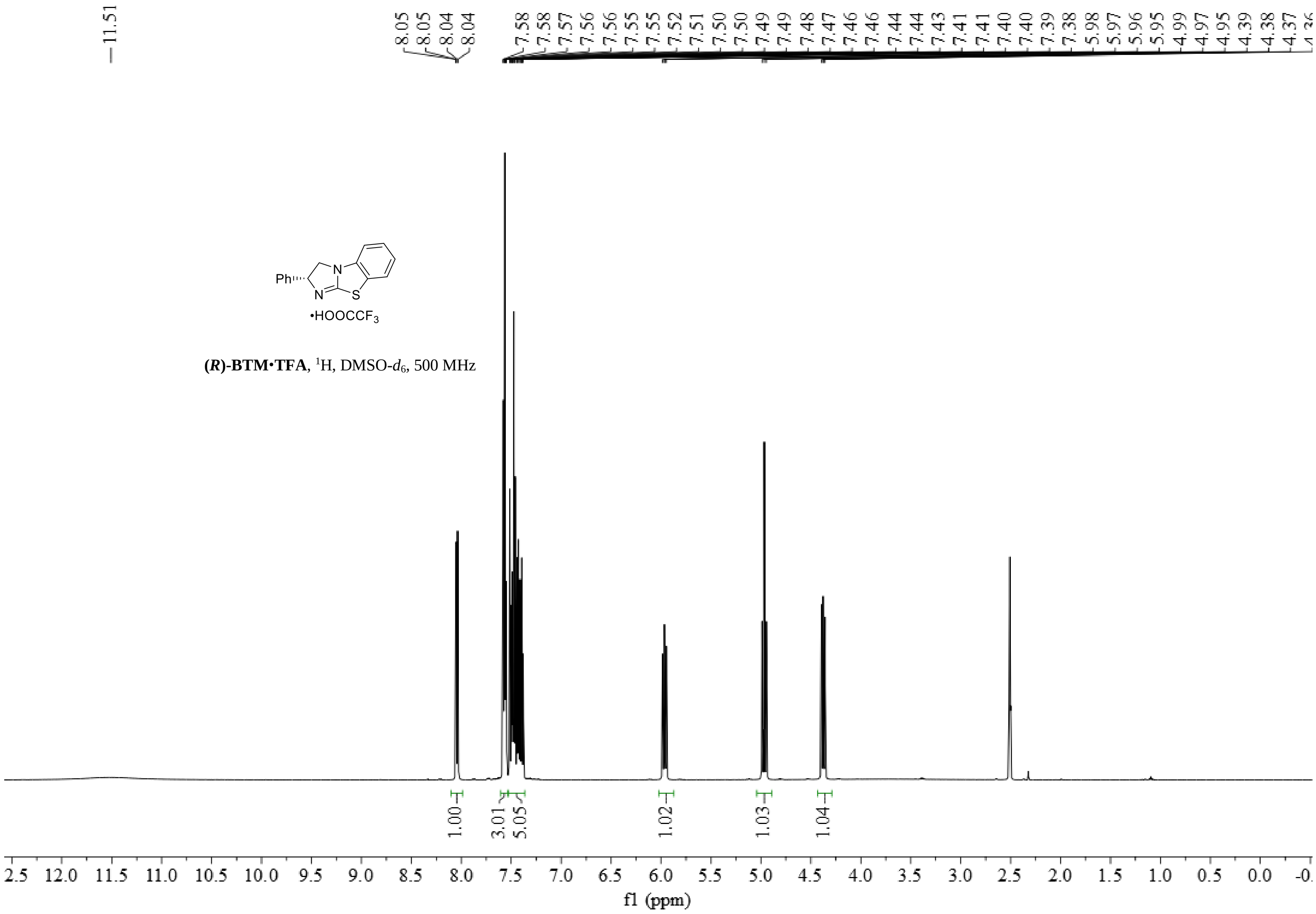

(***R***)**-BTM•TFA**, ^{1}H, DMSO-d_6, 500 MHz

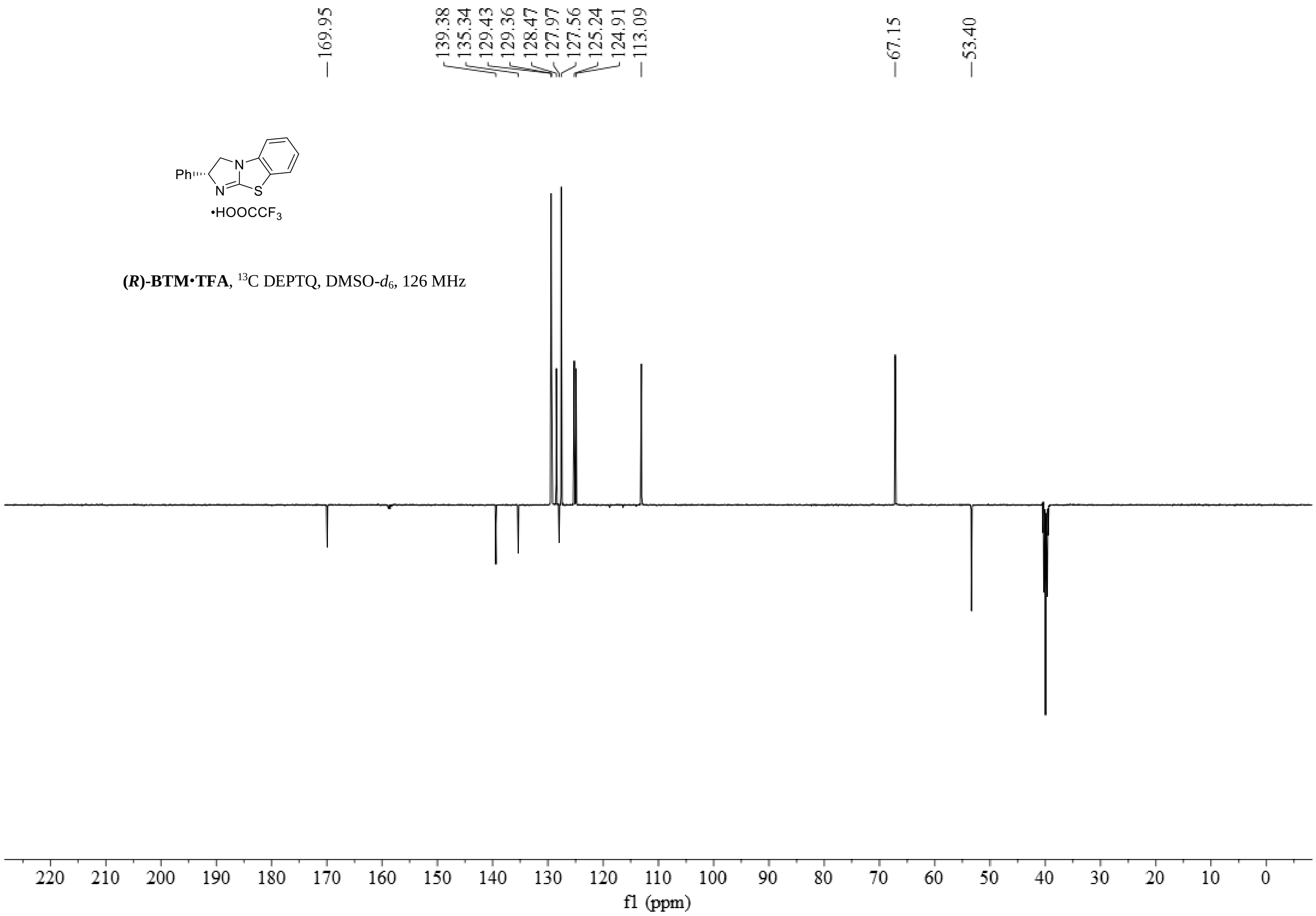

(*R*)-BTM·TFA, ^{13}C DEPTQ, DMSO-d_6, 126 MHz

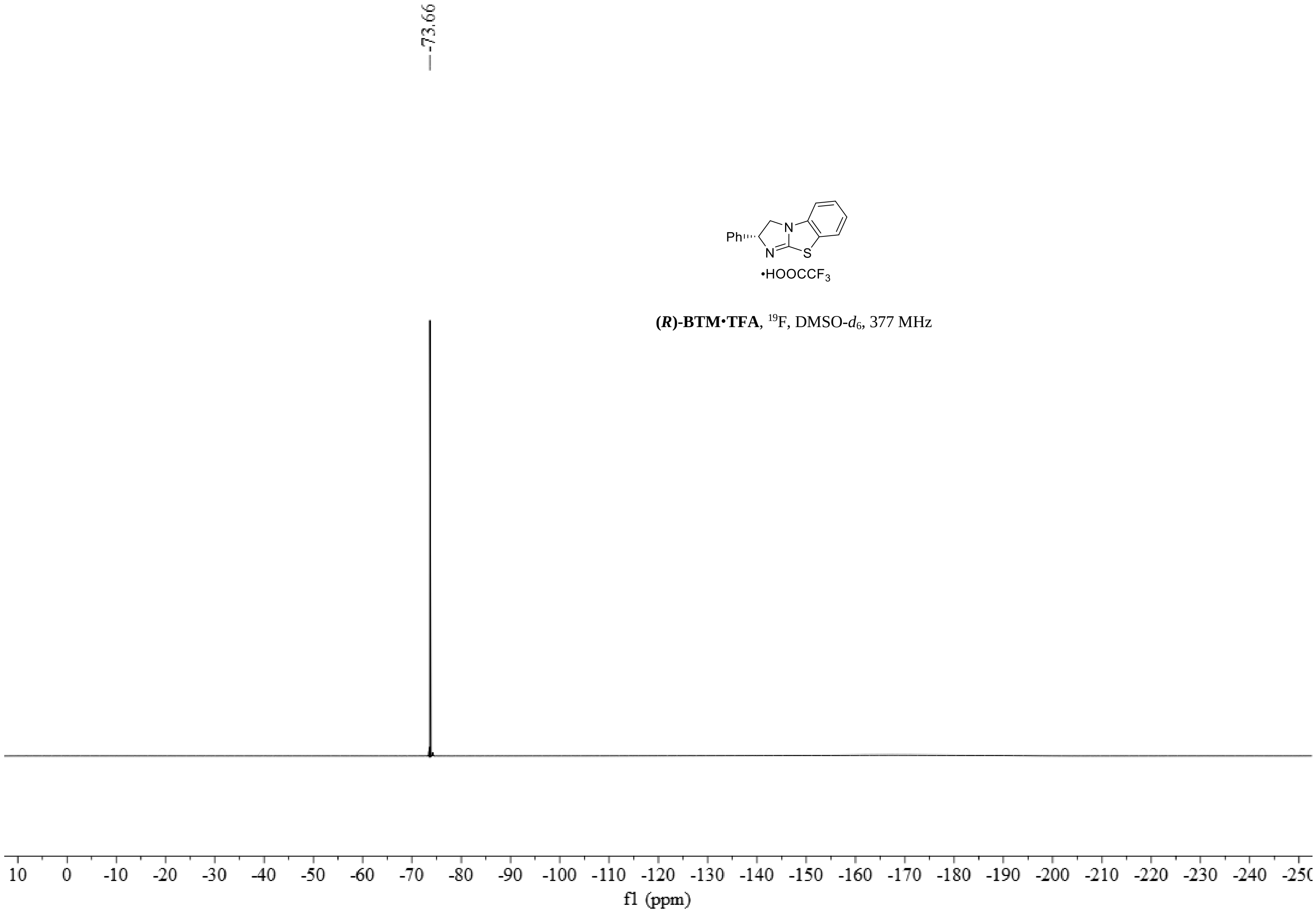

(***R***)**-BTM•TFA**, ^{19}F, DMSO-d_6, 377 MHz

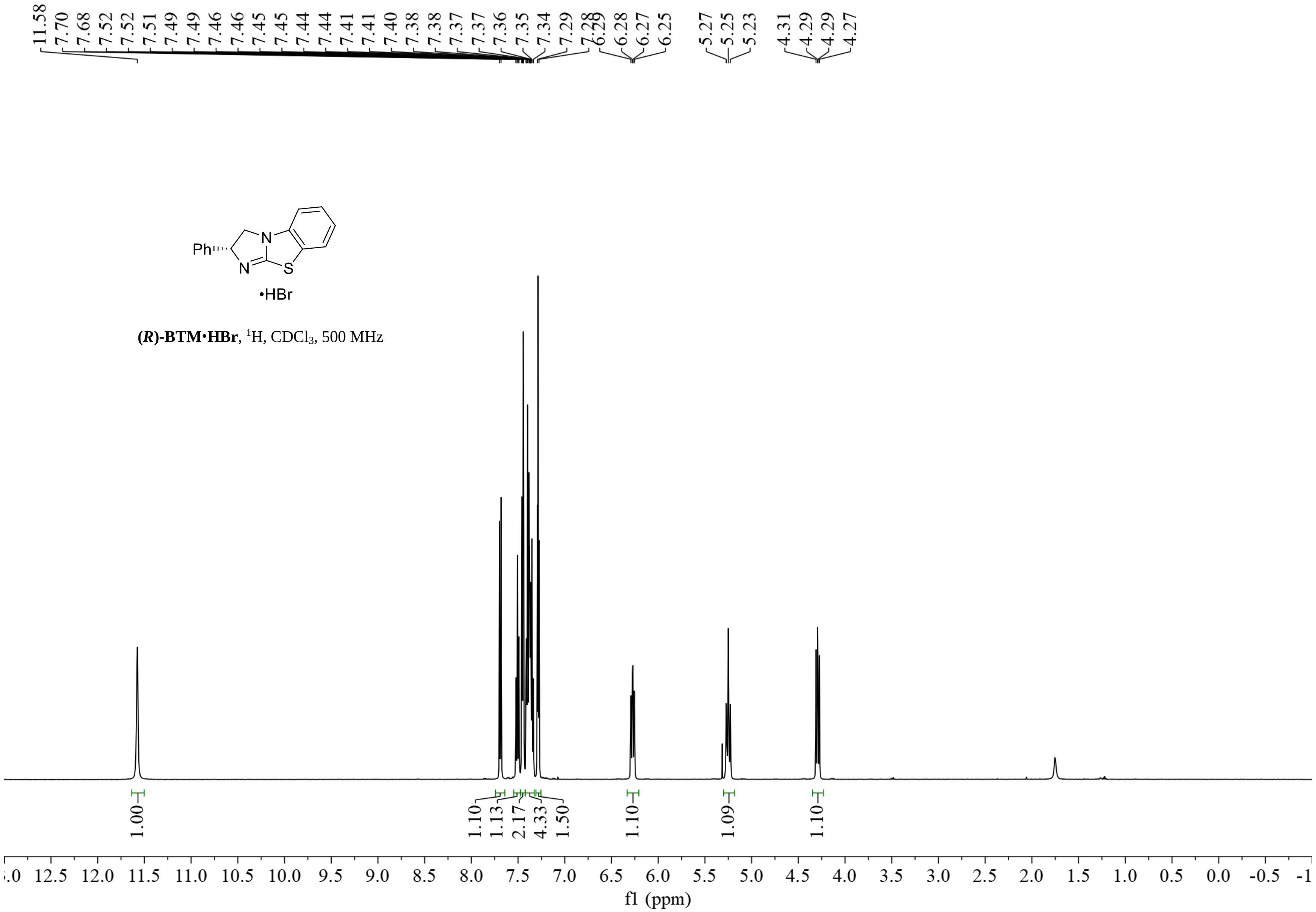
11.58 7.70 7.68 7.52 7.52 7.51 7.49 7.49 7.46 7.46 7.45 7.45 7.44 7.44 7.41 7.41 7.40 7.38 7.38 7.37 7.37 7.36 7.35 7.34 7.29 7.28 6.29 6.28 6.27 6.25 5.27 5.25 5.23 4.31 4.29 4.29 4.27
Ph
N
N
S
•HBr
(R)-BTM•HBr, 1H, CDCl3, 500 MHz
1.00
1.10
1.13
2.17
4.33
1.50
1.10
1.09
1.10
f1 (ppm)

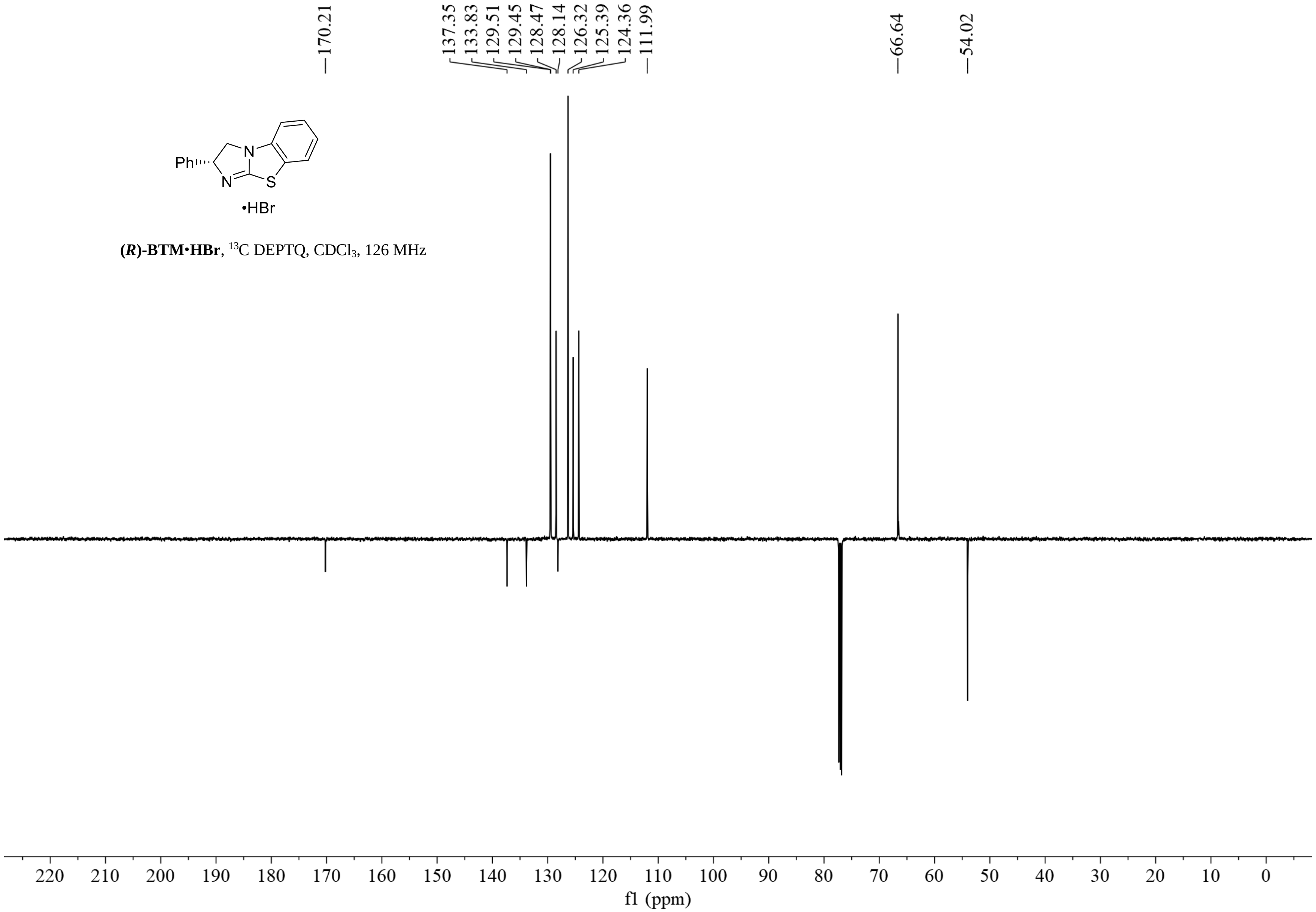

(*R*)-BTM•HBr, ^{13}C DEPTQ, $CDCl_3$, 126 MHz

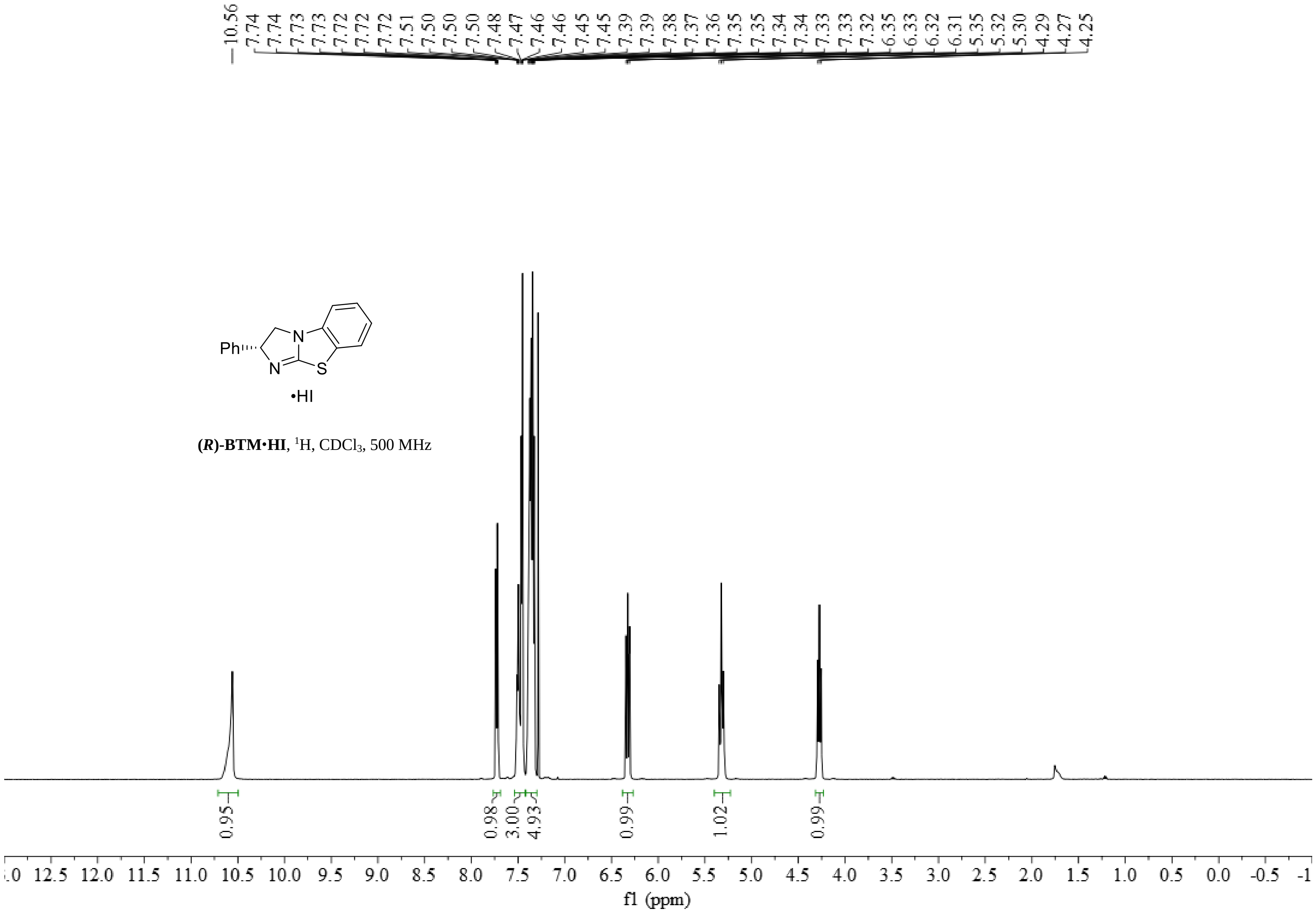

10.56
7.74
7.74
7.73
7.73
7.72
7.72
7.72
7.51
7.50
7.50
7.50
7.48
7.47
7.46
7.46
7.45
7.45
7.39
7.39
7.38
7.37
7.37
7.36
7.35
7.35
7.34
7.34
7.33
7.33
7.32
6.35
6.33
6.32
6.31
5.35
5.32
5.30
4.29
4.27
4.25
Ph
N
N
S
•HI
(*R*)-BTM•HI, ¹H, CDCl3, 500 MHz
0.95
0.98
3.00
4.93
0.99
1.02
0.99
12.5 12.0 11.5 11.0 10.5 10.0 9.5 9.0 8.5 8.0 7.5 7.0 6.5 6.0 5.5 5.0 4.5 4.0 3.5 3.0 2.5 2.0 1.5 1.0 0.5 0.0 -0.5
f1 (ppm)

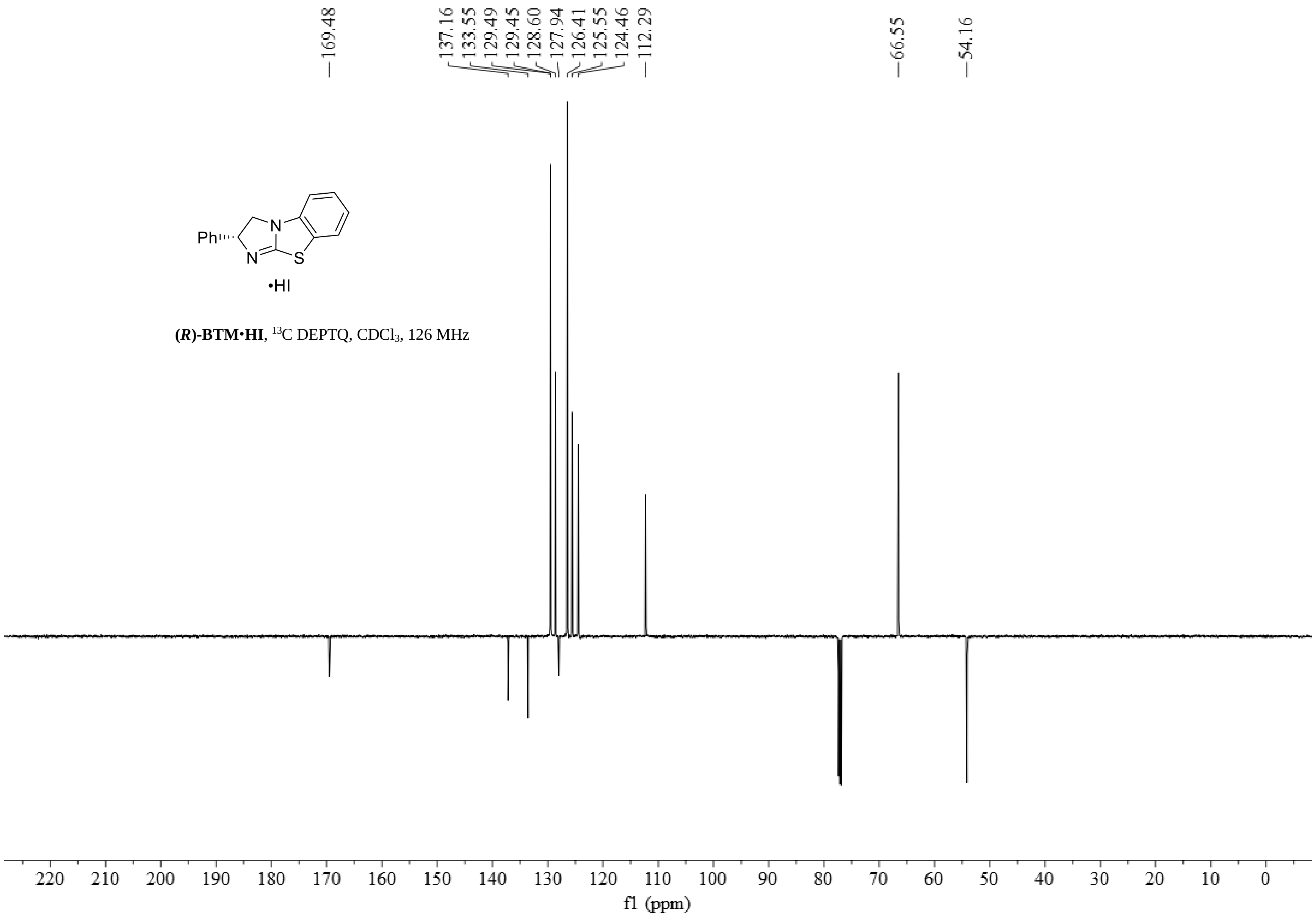

169.48
137.16
133.55
129.49
129.45
128.60
127.94
126.41
125.55
124.46
112.29
66.55
54.16
Ph
N
N
S
•HI
(*R*)-BTM•HI, 13C DEPTQ, CDCl3, 126 MHz
220 210 200 190 180 170 160 150 140 130 120 110 100 90 80 70 60 50 40 30 20 10 0
f1 (ppm)

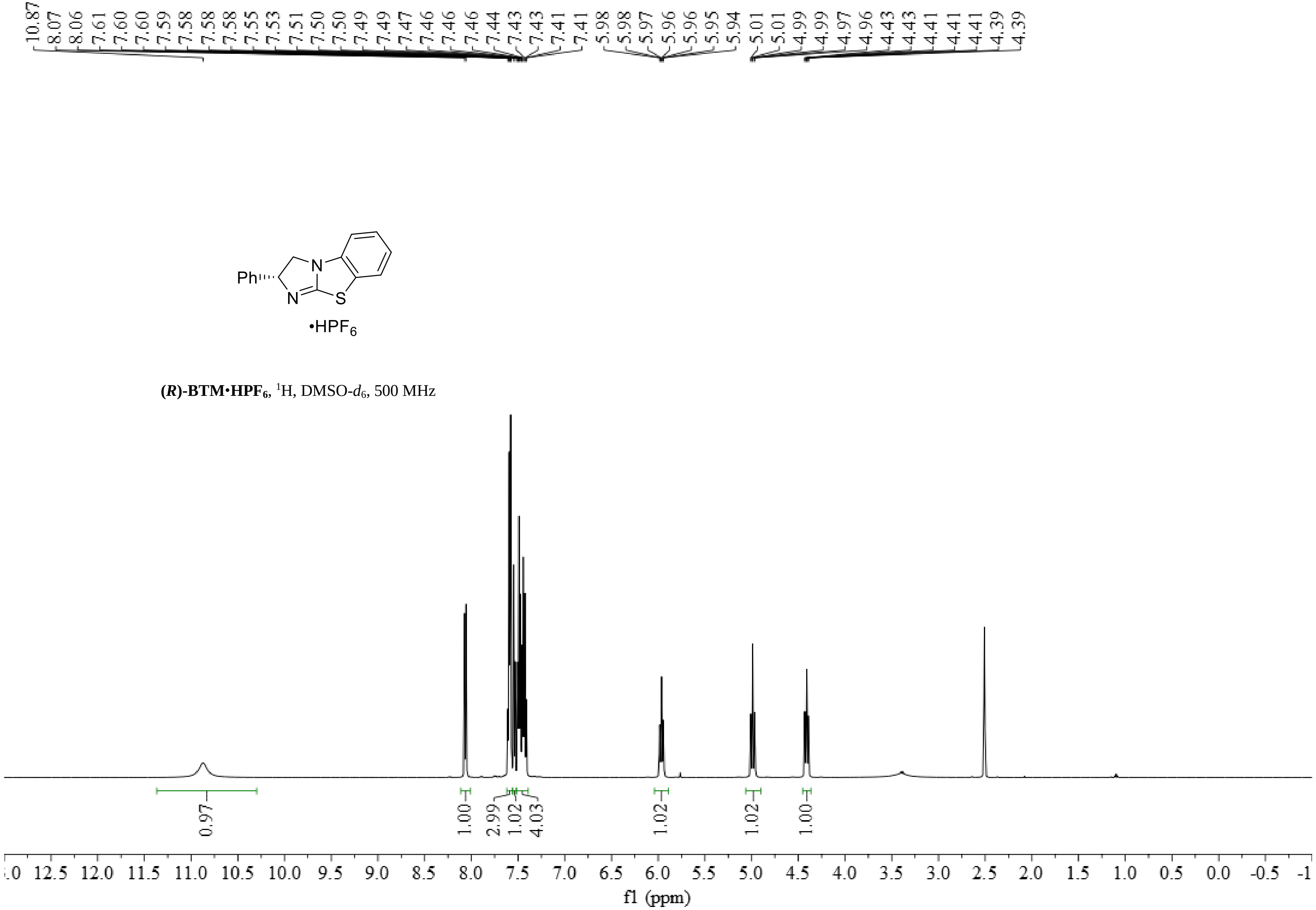

(***R***)-**BTM•HPF$_6$**, ^{1}H, DMSO-d_6, 500 MHz

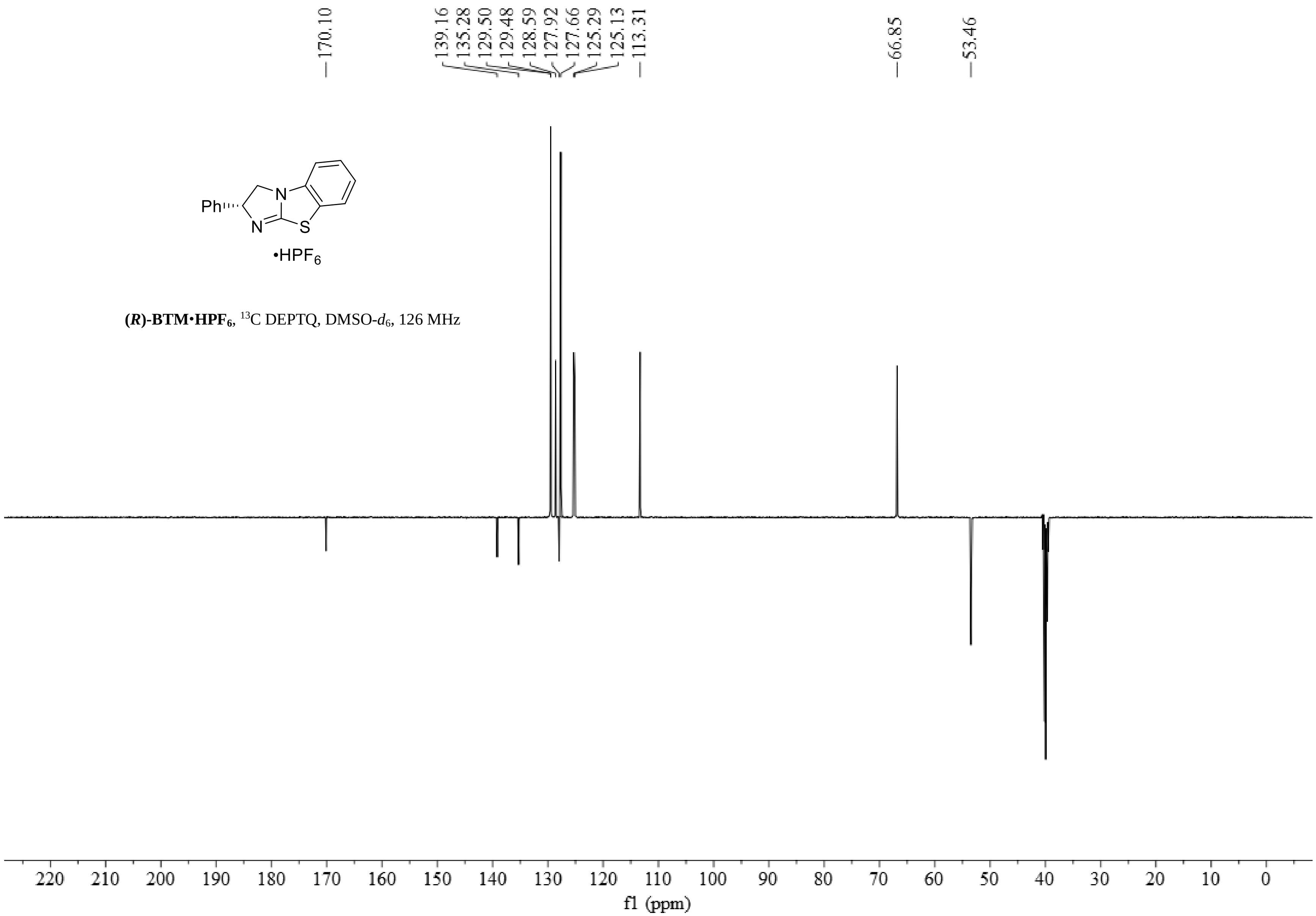

(*R*)-BTM•HPF$_6$, ^{13}C DEPTQ, DMSO-d_6, 126 MHz

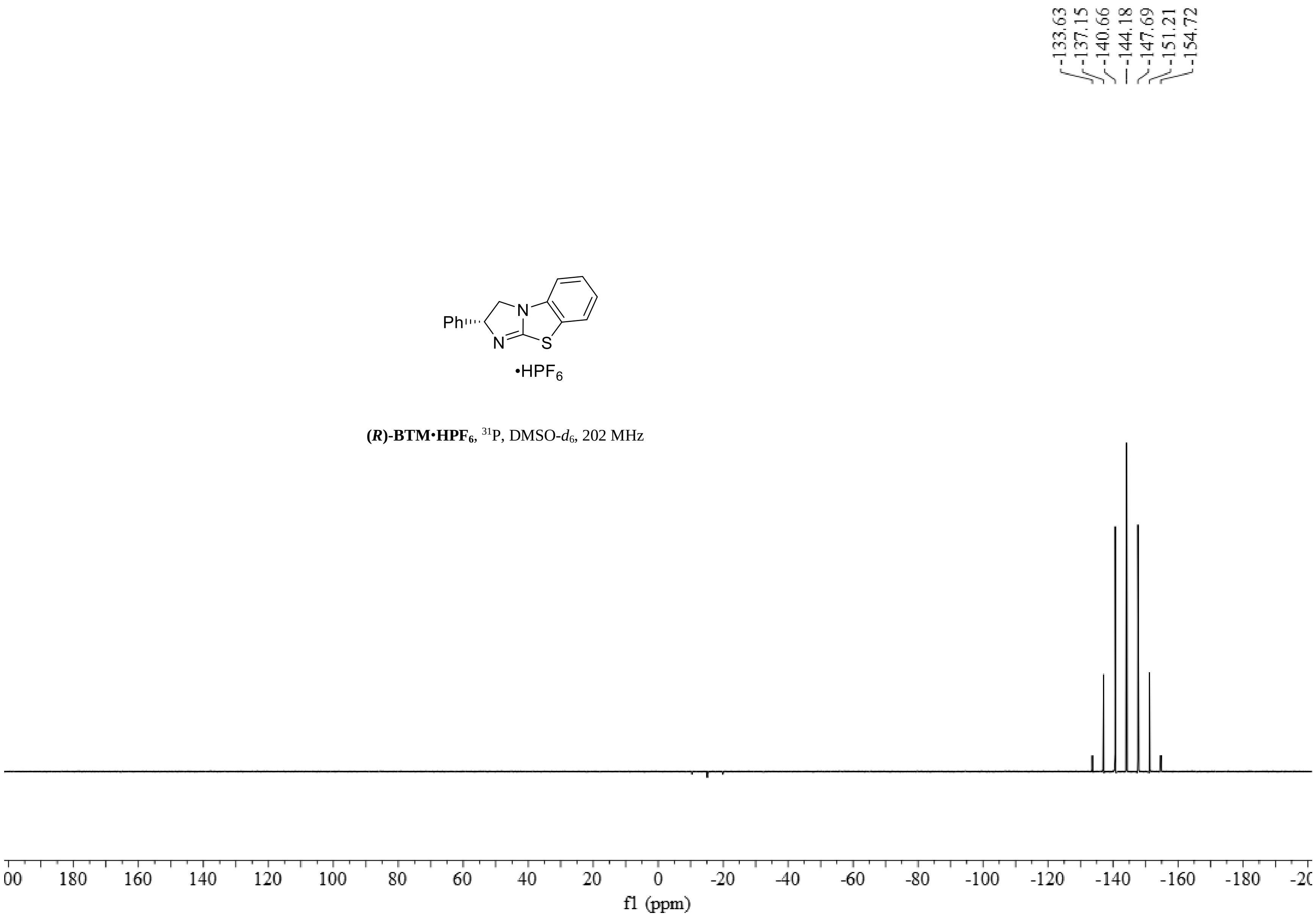

(*R*)-BTM•HPF$_6$, ^{31}P, DMSO-d_6, 202 MHz

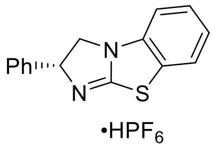

(*R*)-BTM•HPF$_6$, ^{19}F, DMSO-d_6, 471 MHz

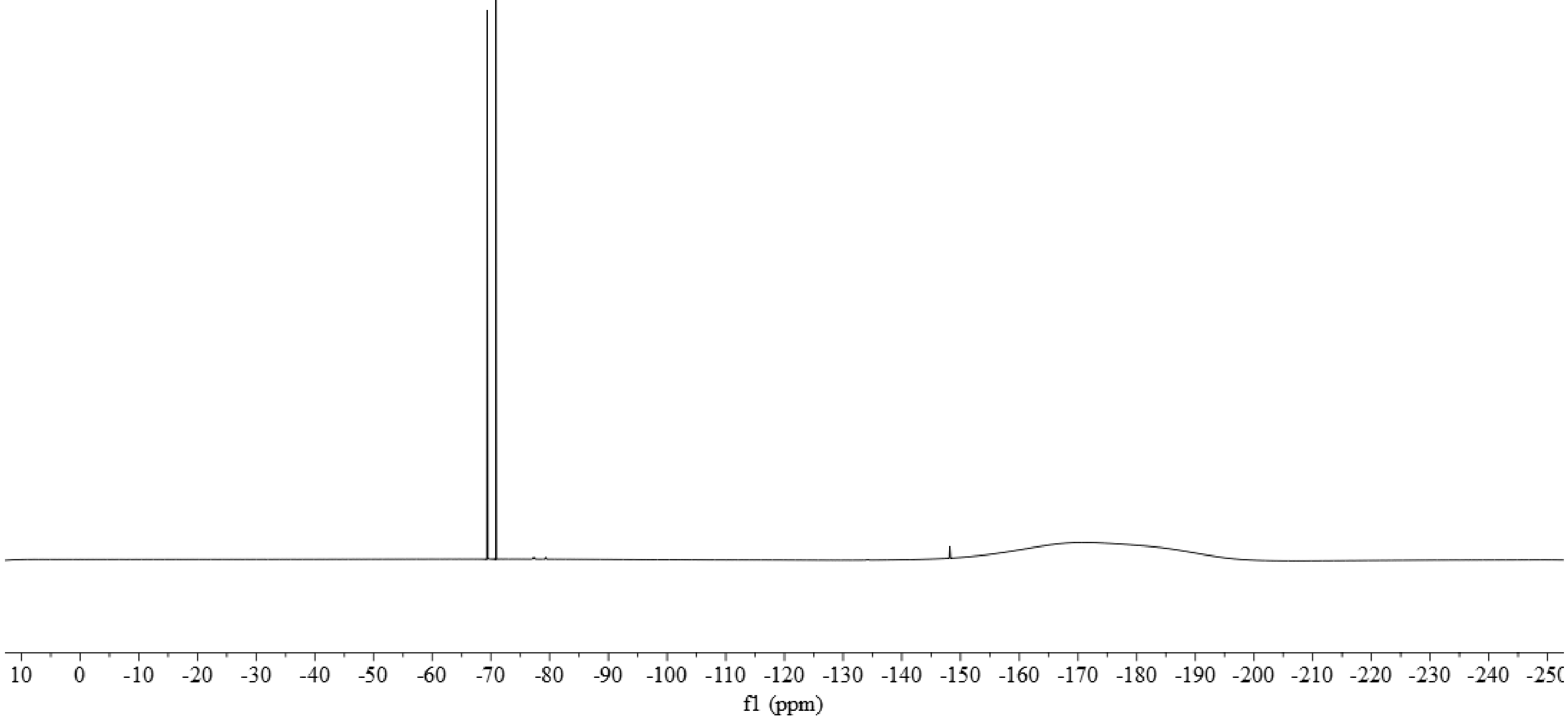

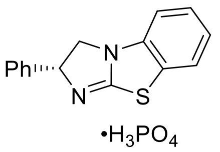

(***R***)**-BTM•H**$_3$**PO**$_4$, ^{1}H, DMSO-d_6, 400 MHz

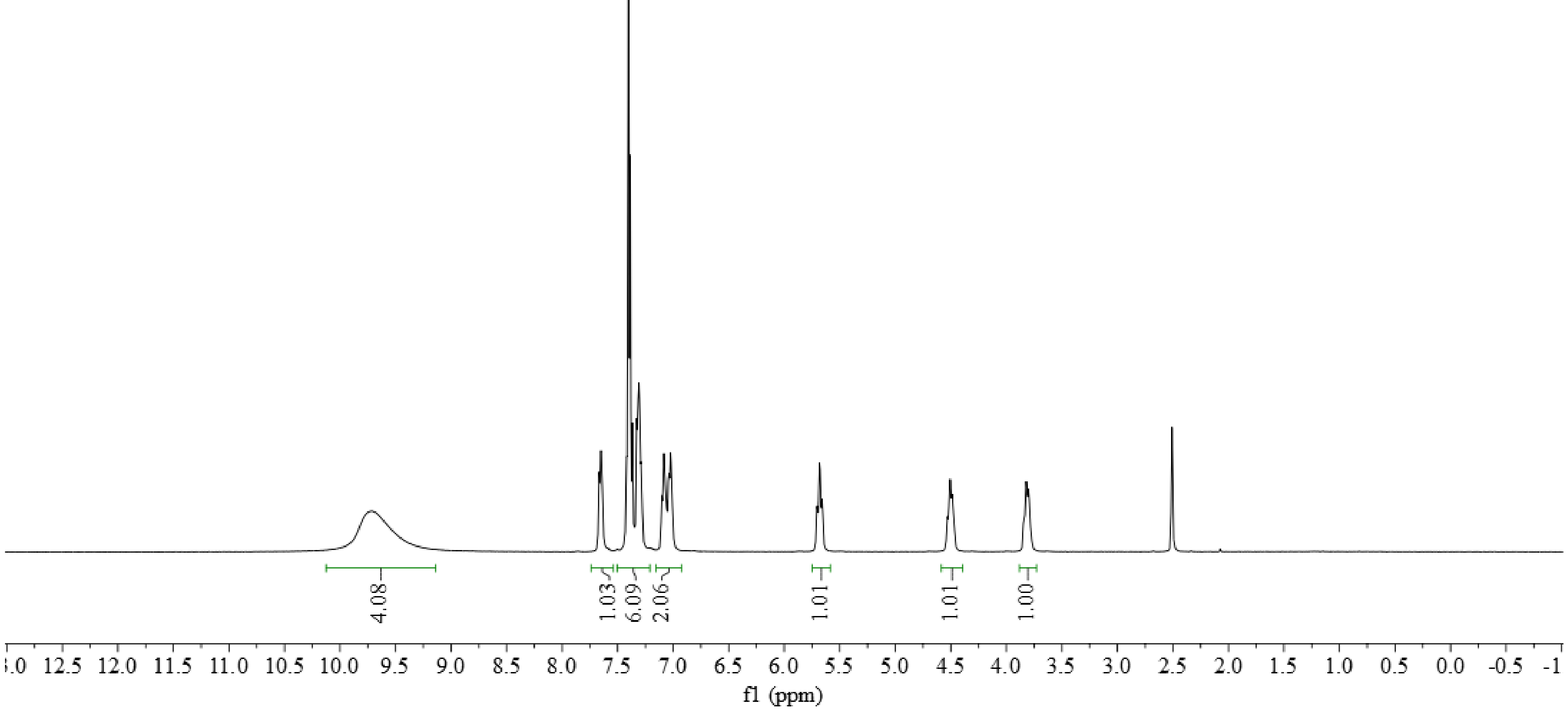

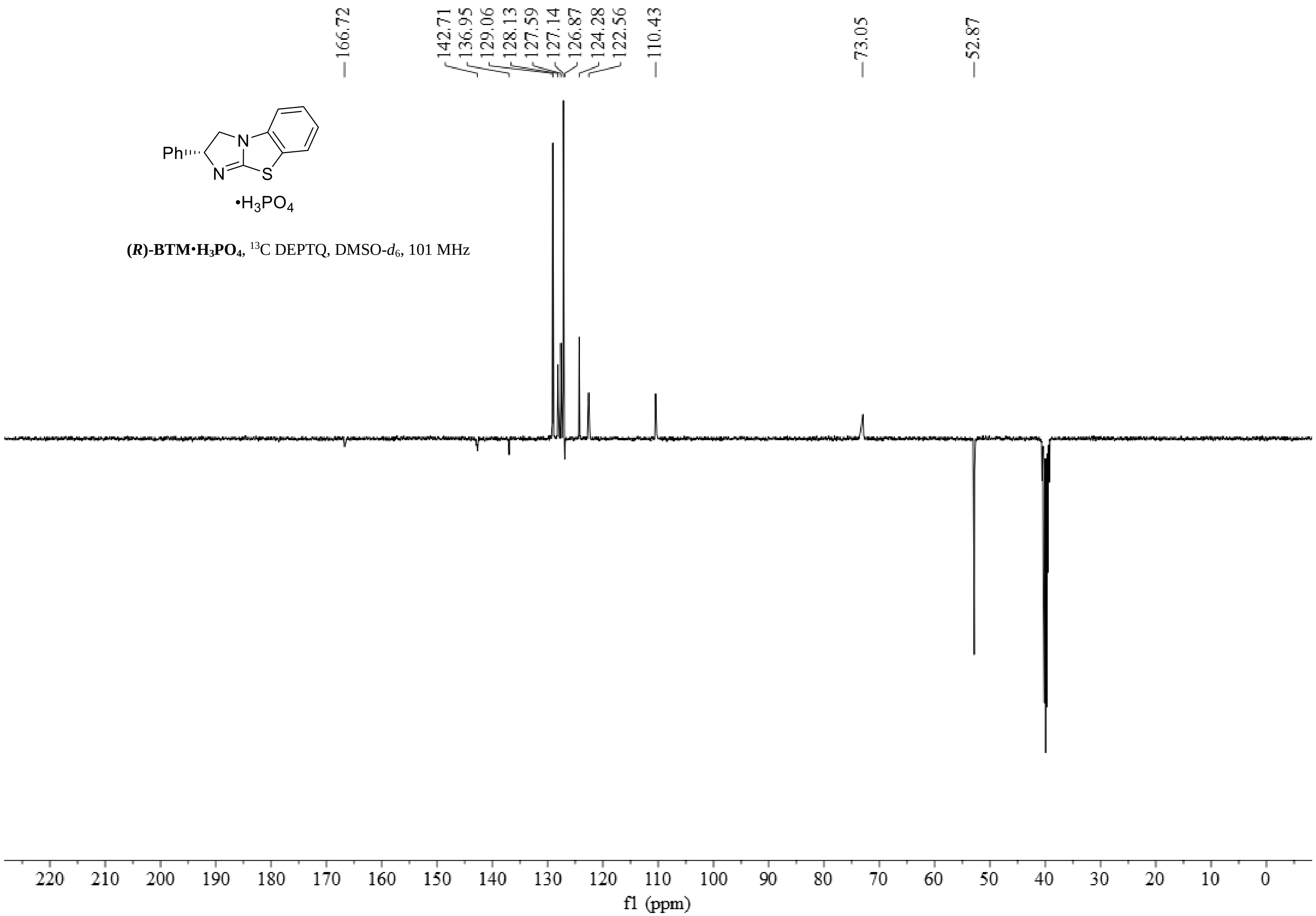

(*R*)-BTM•H_3PO_4, ^{13}C DEPTQ, DMSO-d_6, 101 MHz

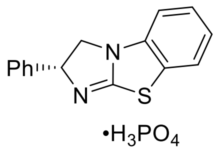

(*R*)-BTM•H_3PO_4, ^{31}P, DMSO-d_6, 162 MHz

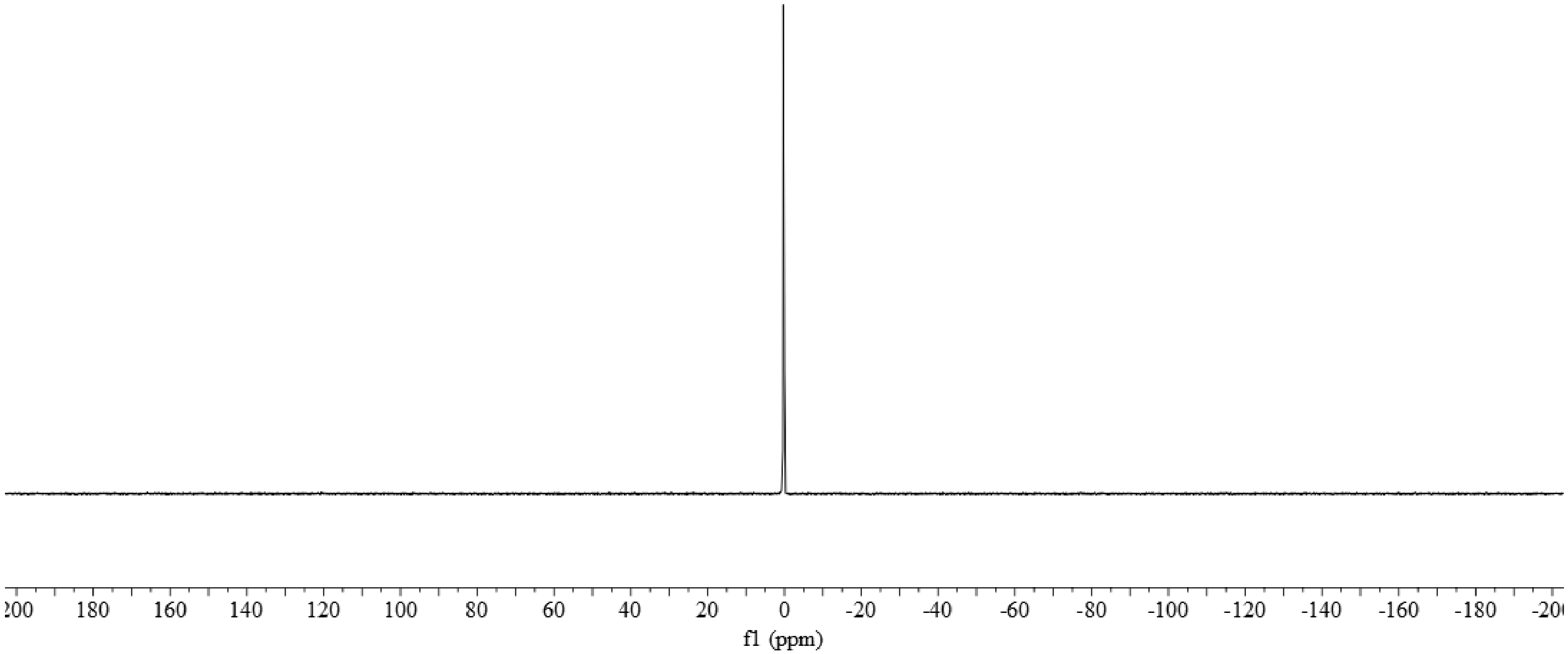

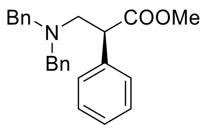

9, ^{1}H, $CDCl_3$, 400 MHz

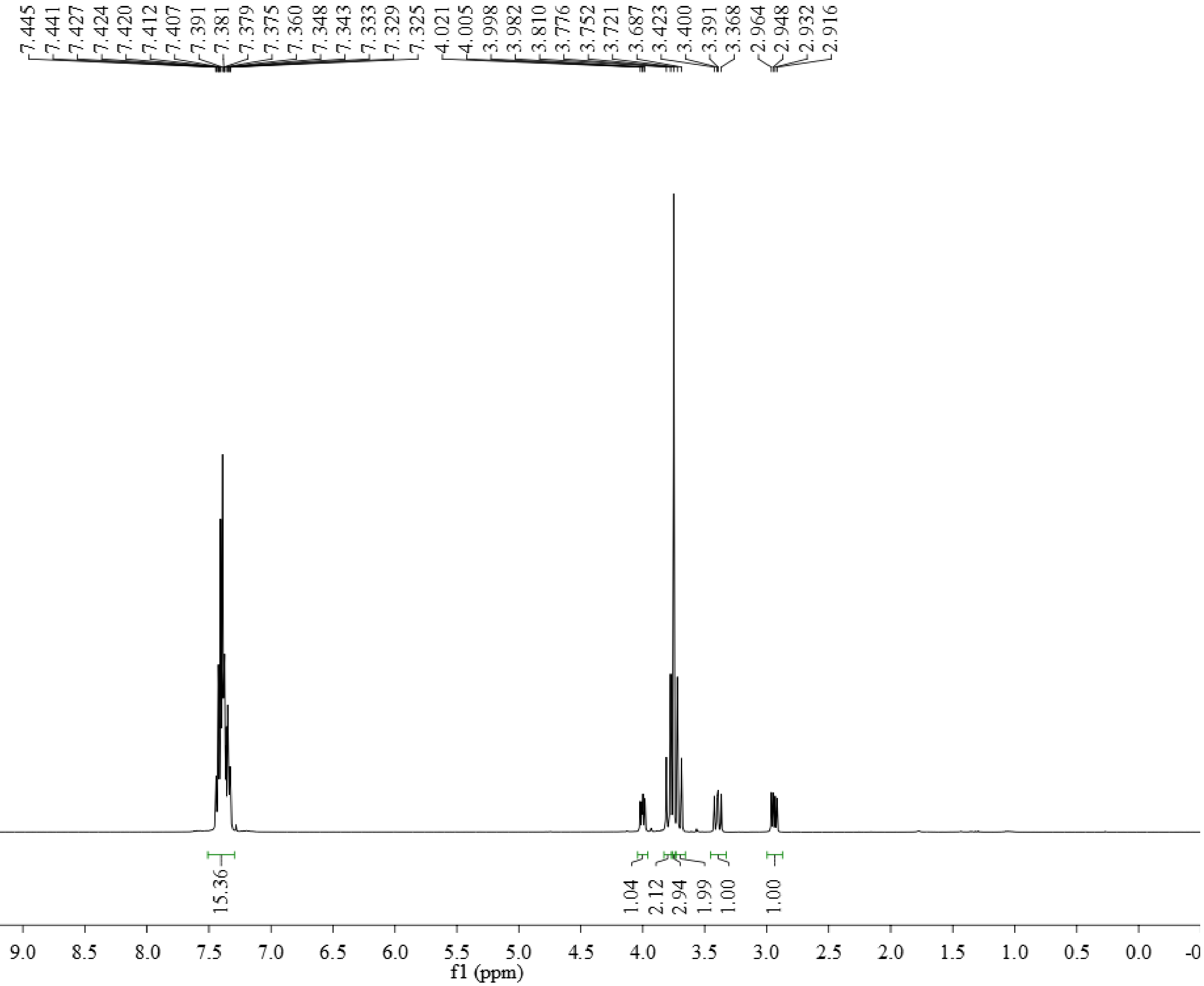

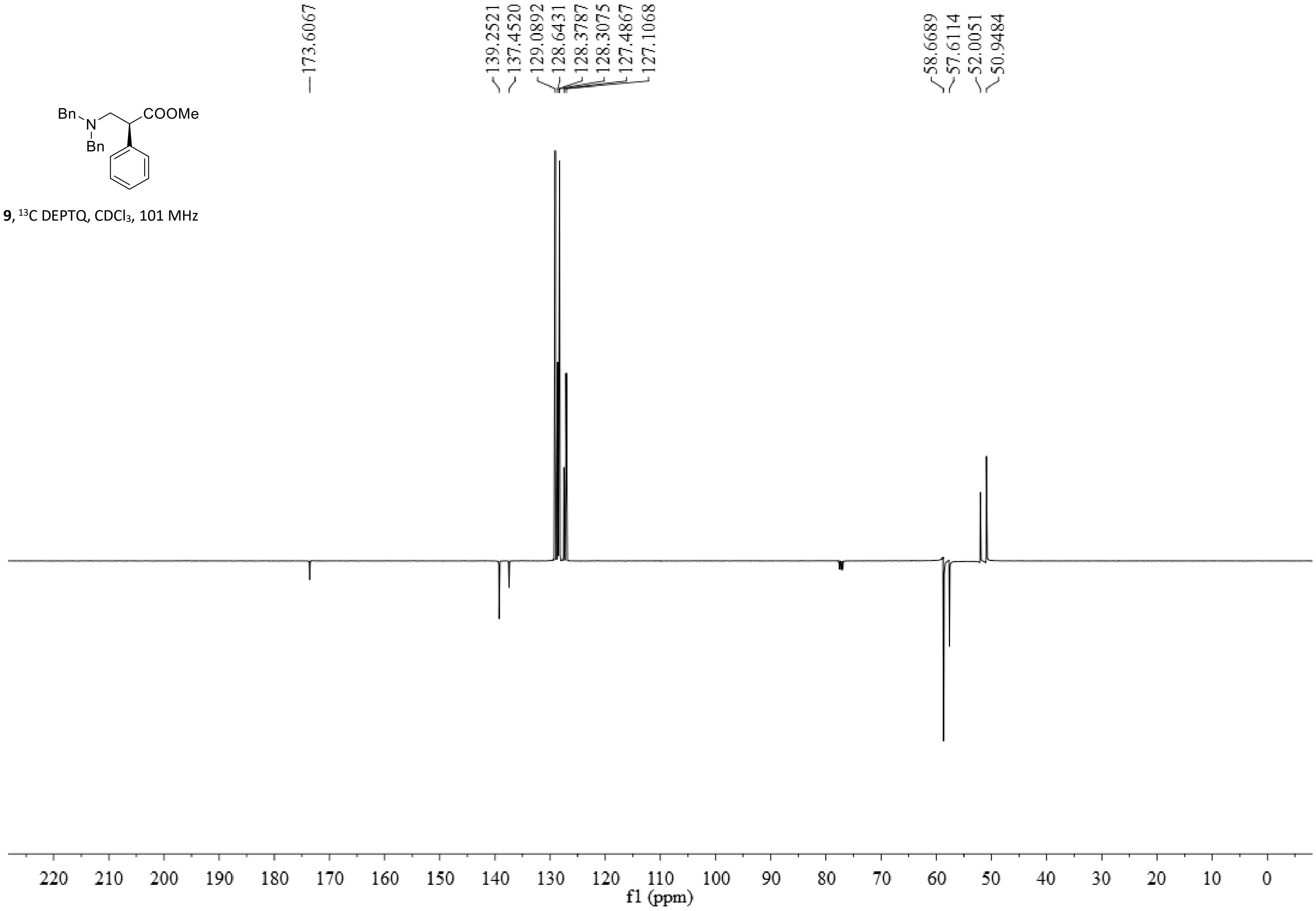

9, ^{13}C DEPTQ, $CDCl_3$, 101 MHz

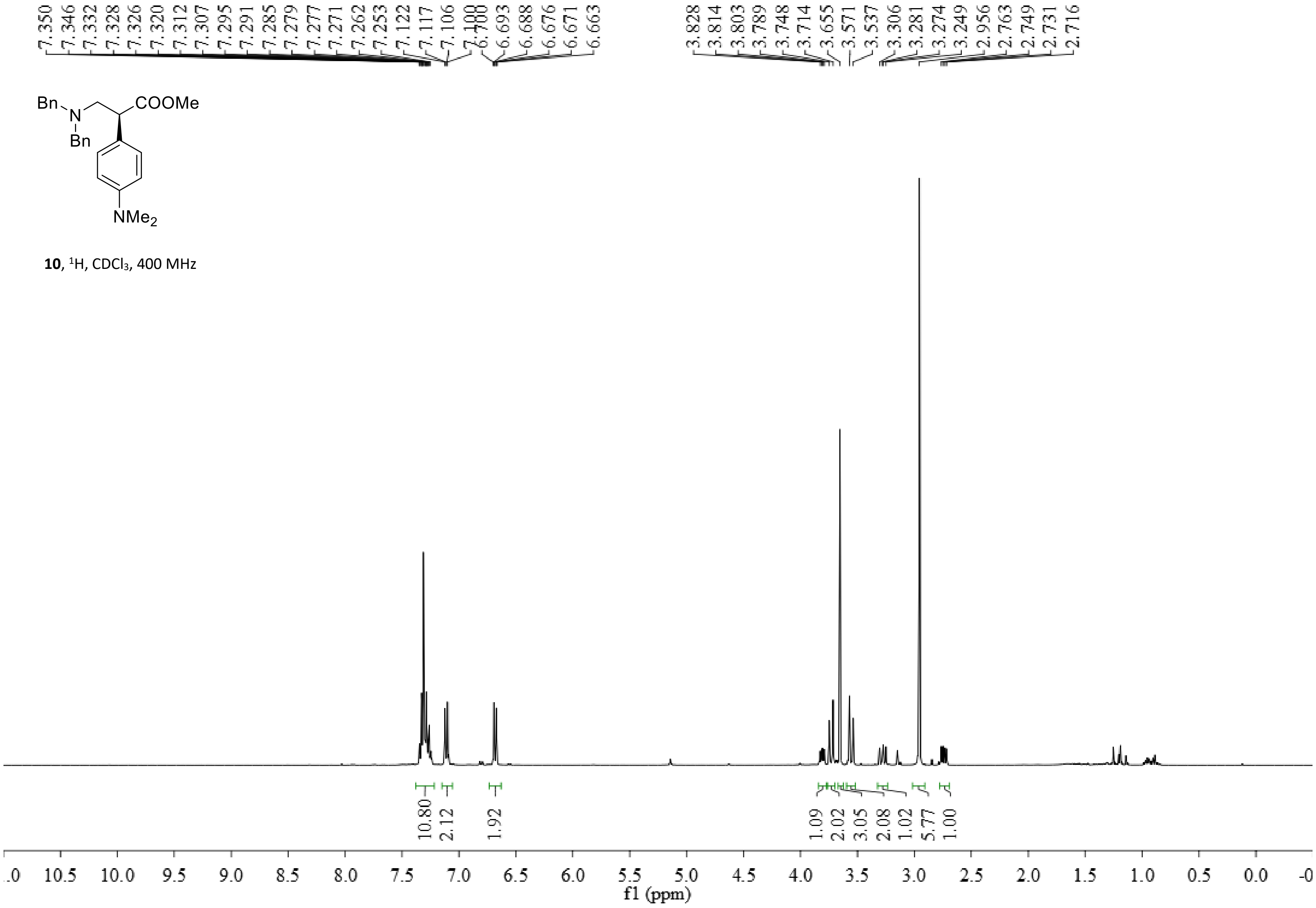

10, ^{1}H, $CDCl_3$, 400 MHz

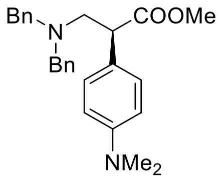

10, ^{13}C, $CDCl_3$, 101 MHz

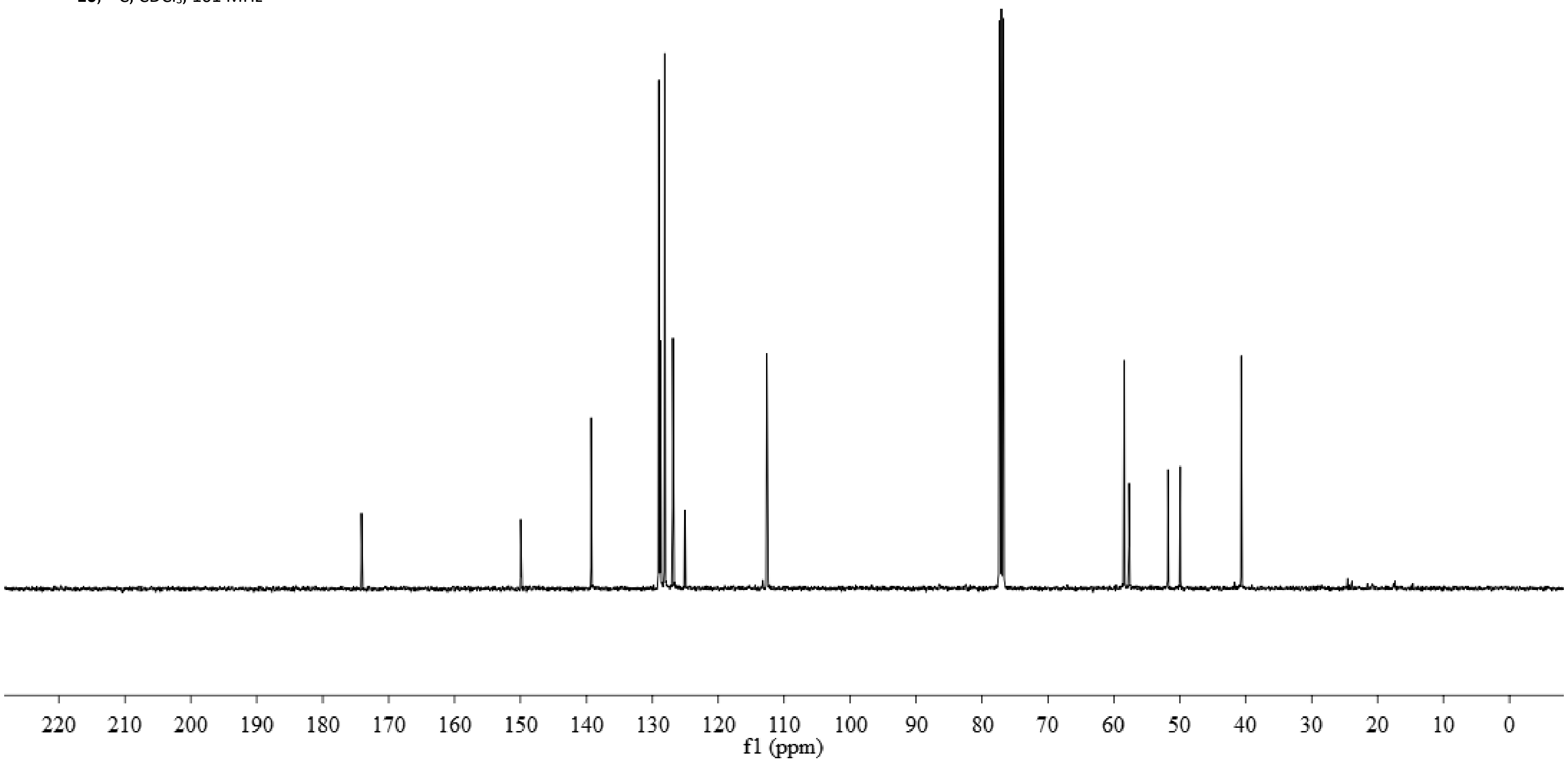

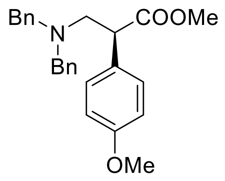

11, ^{1}H, $CDCl_3$, 400 MHz

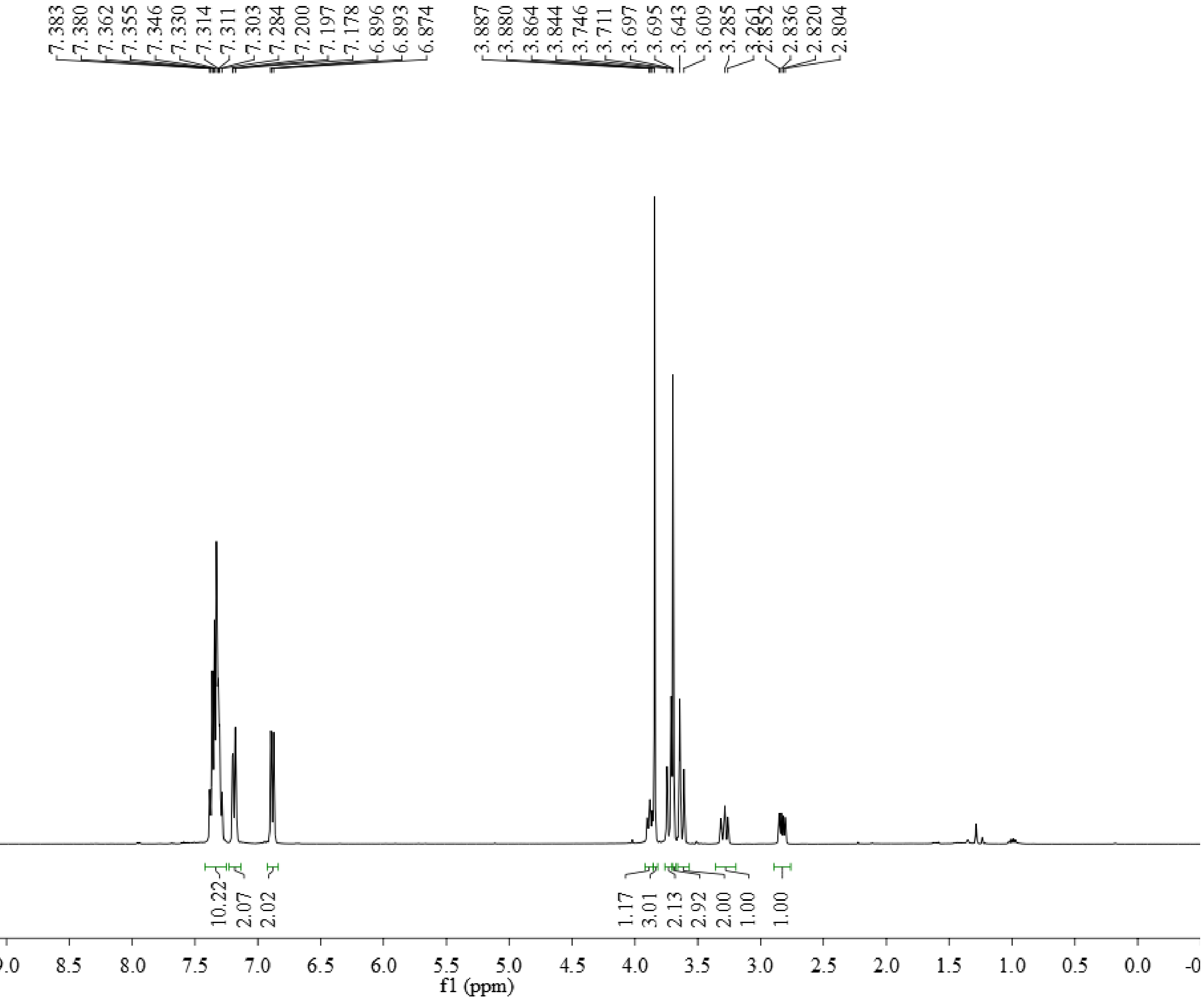

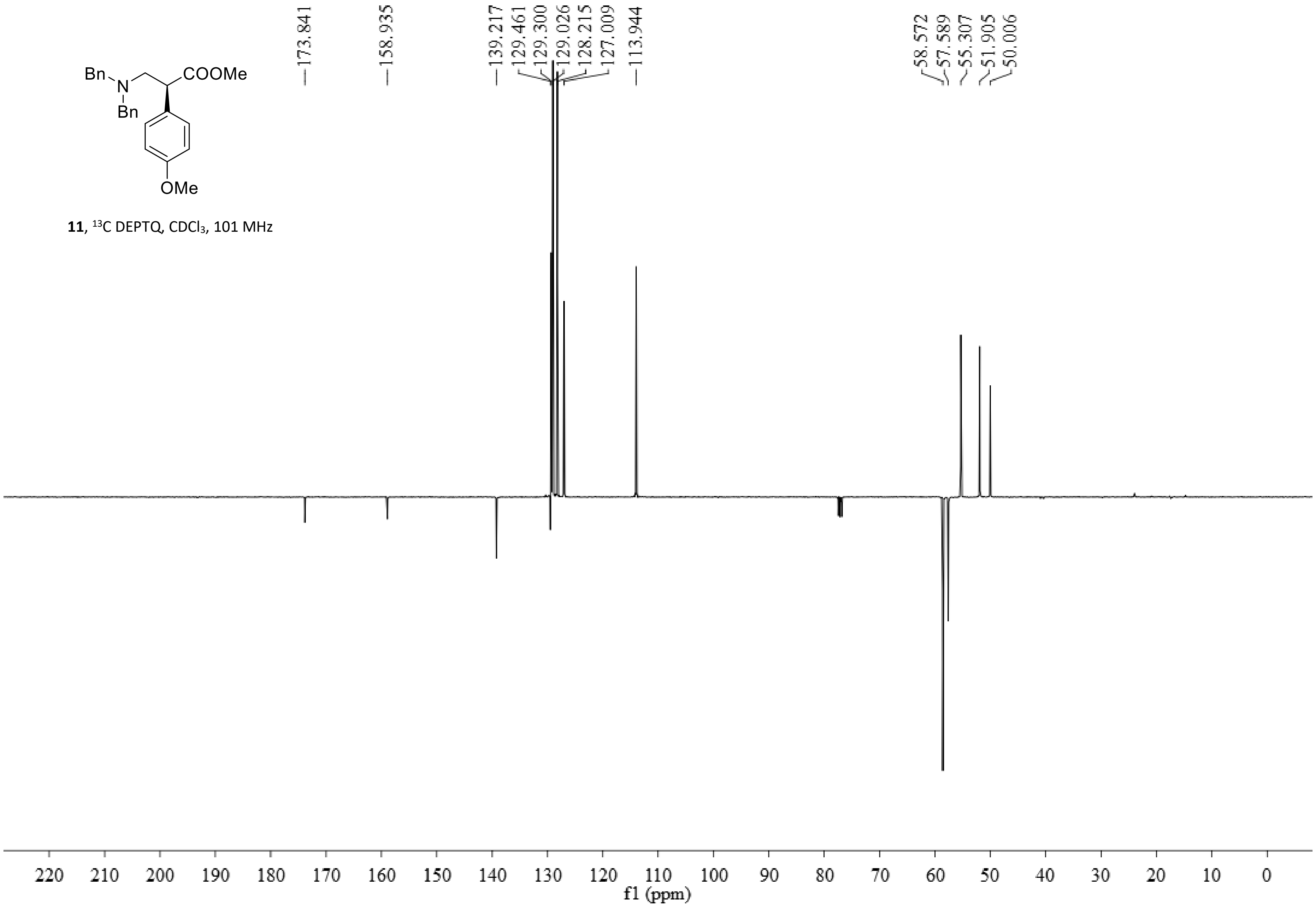

11, ^{13}C DEPTQ, $CDCl_3$, 101 MHz

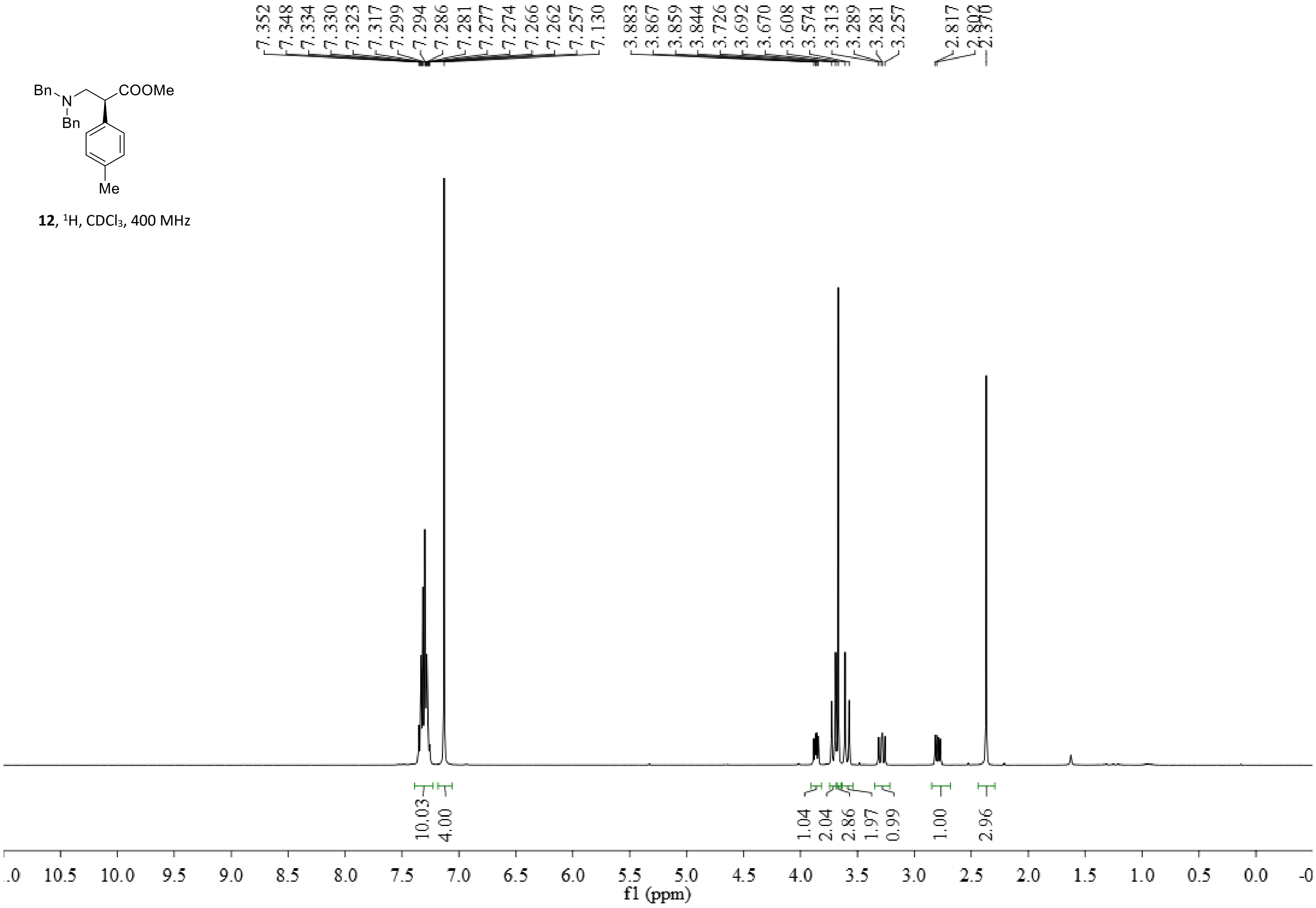

12, ^{1}H, $CDCl_3$, 400 MHz

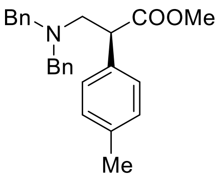

12, ^{13}C, $CDCl_3$, 101 MHz

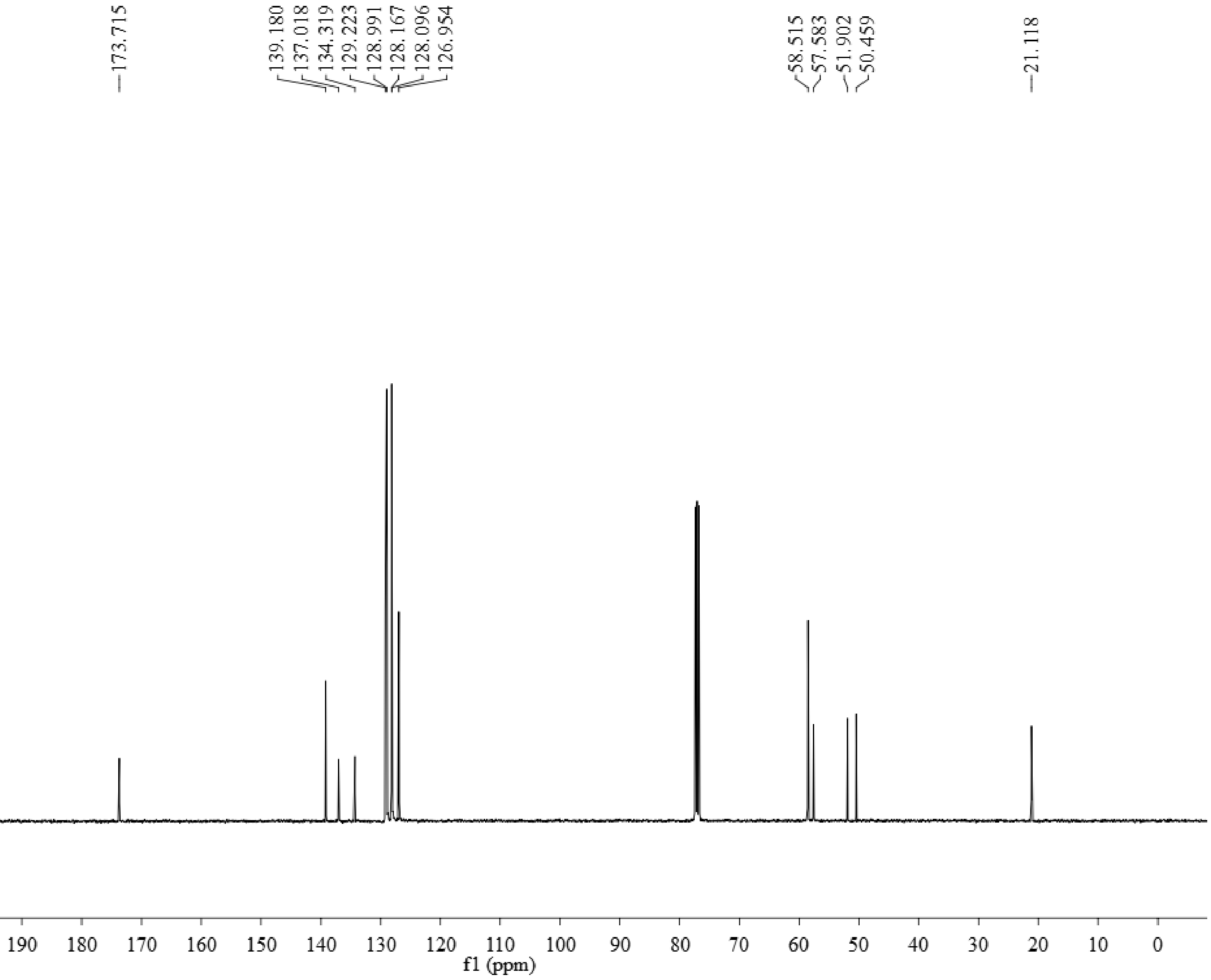

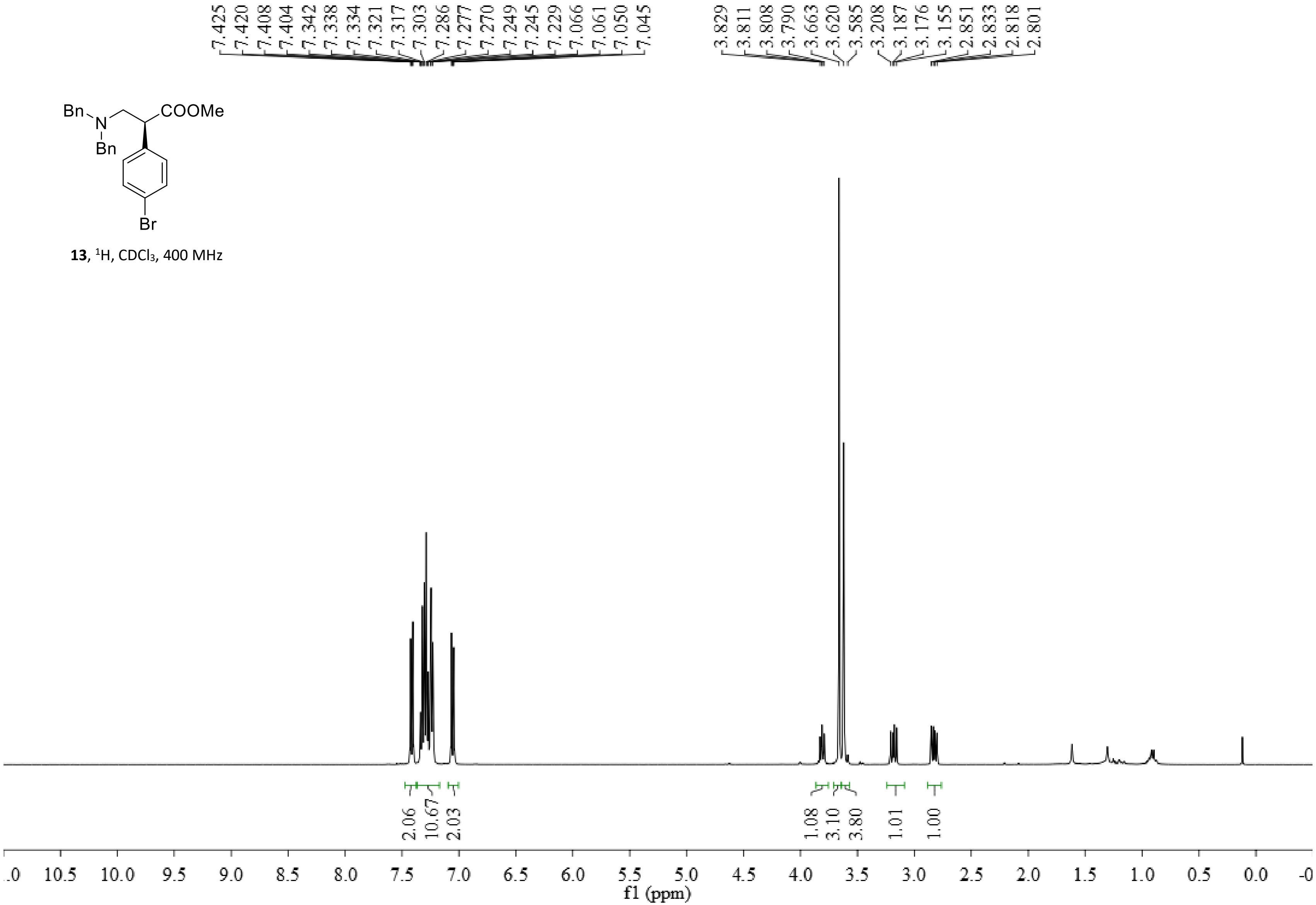

13, ^{1}H, $CDCl_3$, 400 MHz

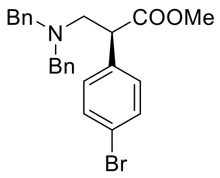

13, ^{13}C, $CDCl_3$, 101 MHz

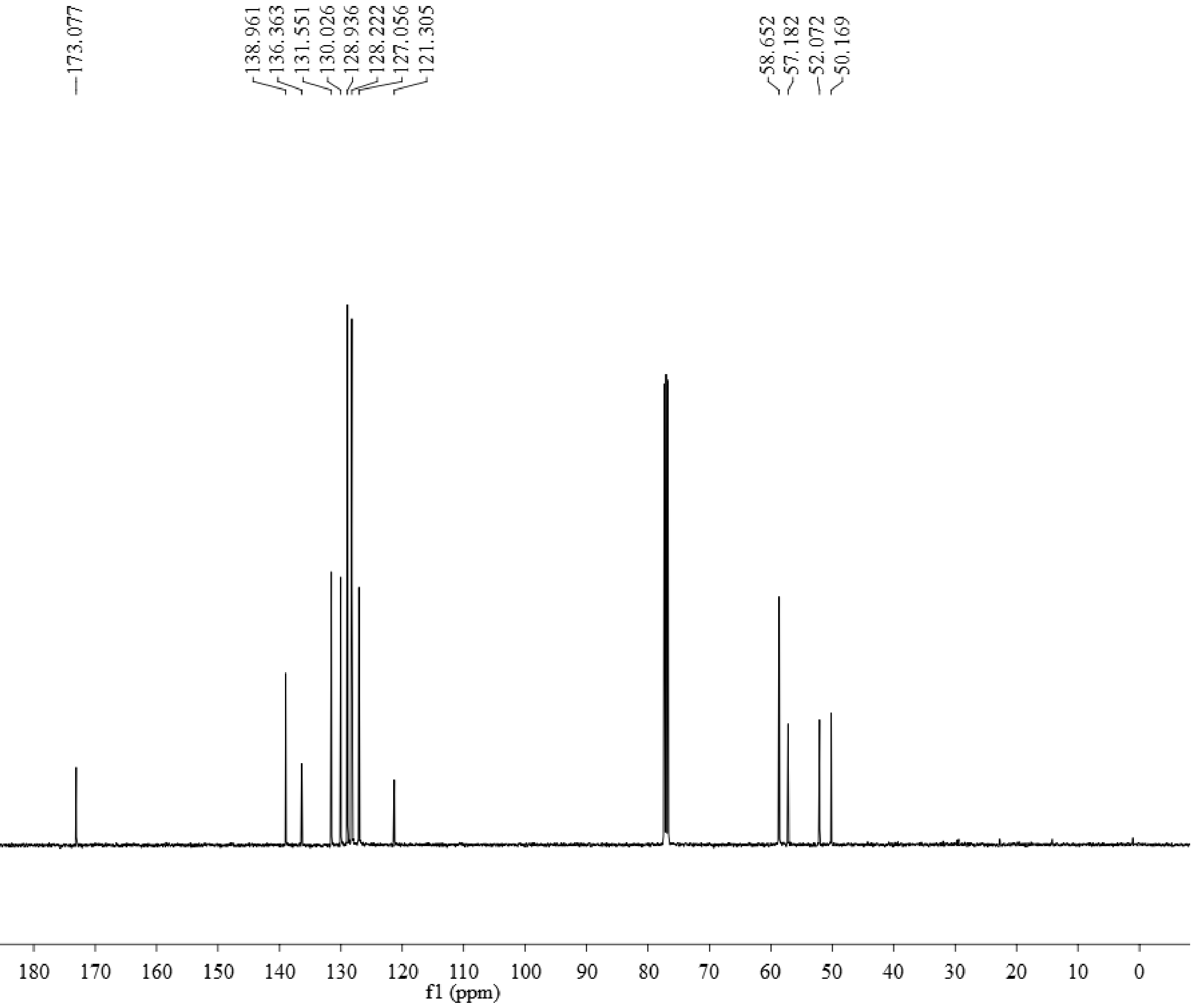

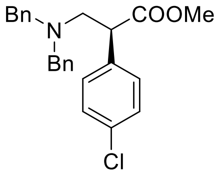

14, ^{1}H, $CDCl_3$, 400 MHz

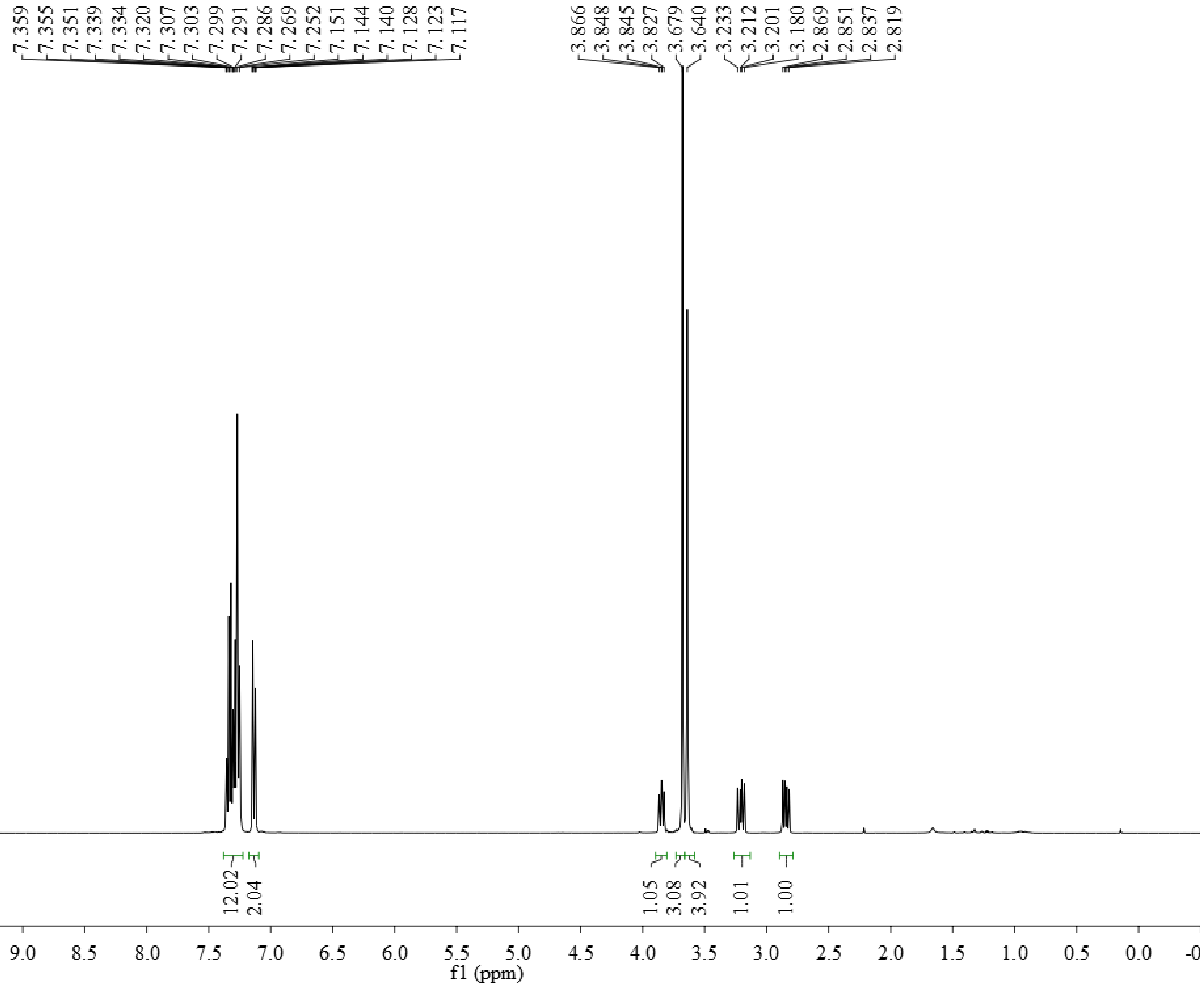

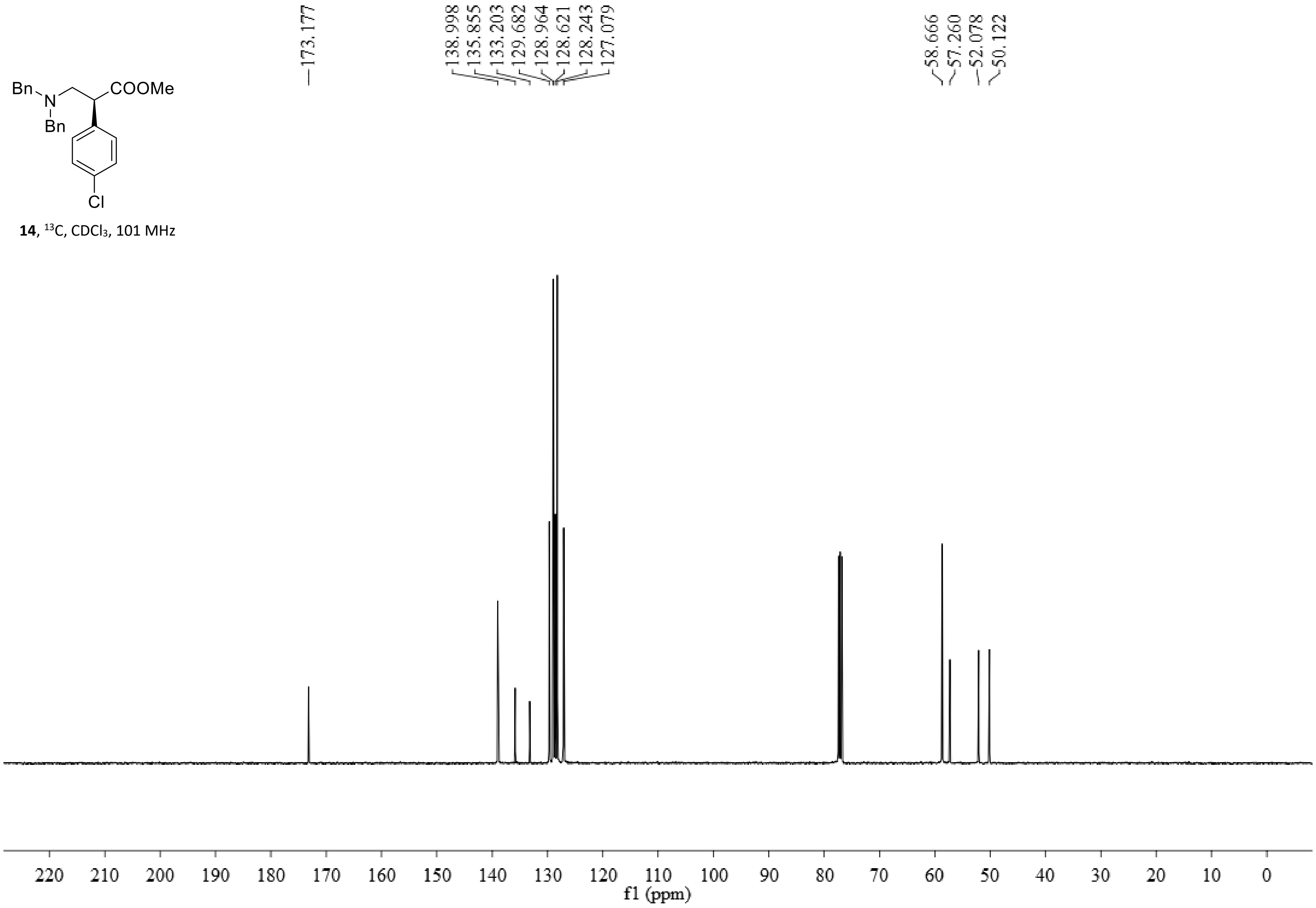

14, ^{13}C, $CDCl_3$, 101 MHz

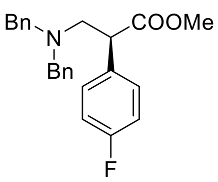

15, ^{1}H, $CDCl_3$, 400 MHz

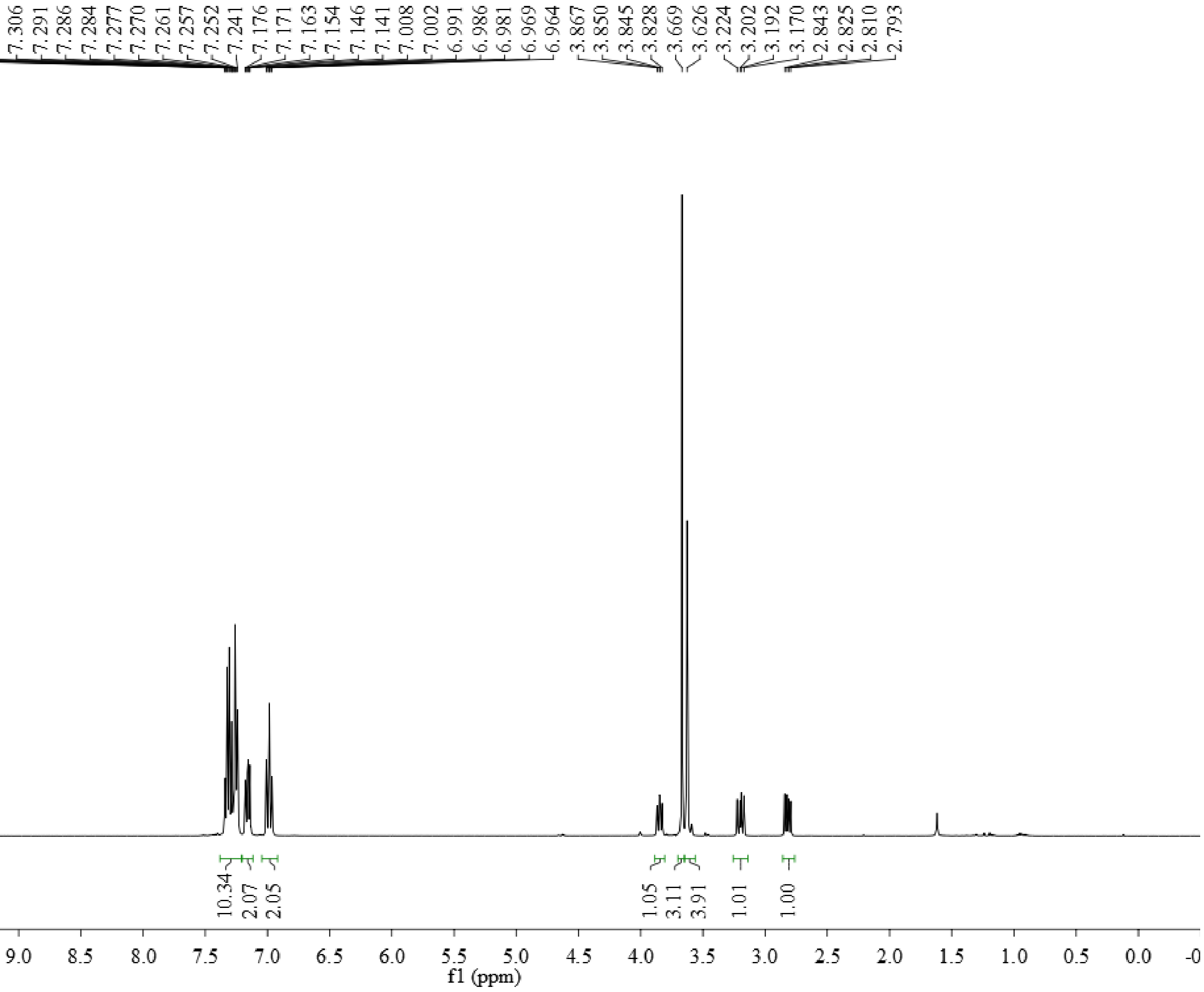

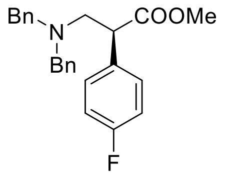

15, ^{13}C, $CDCl_3$, 101 MHz

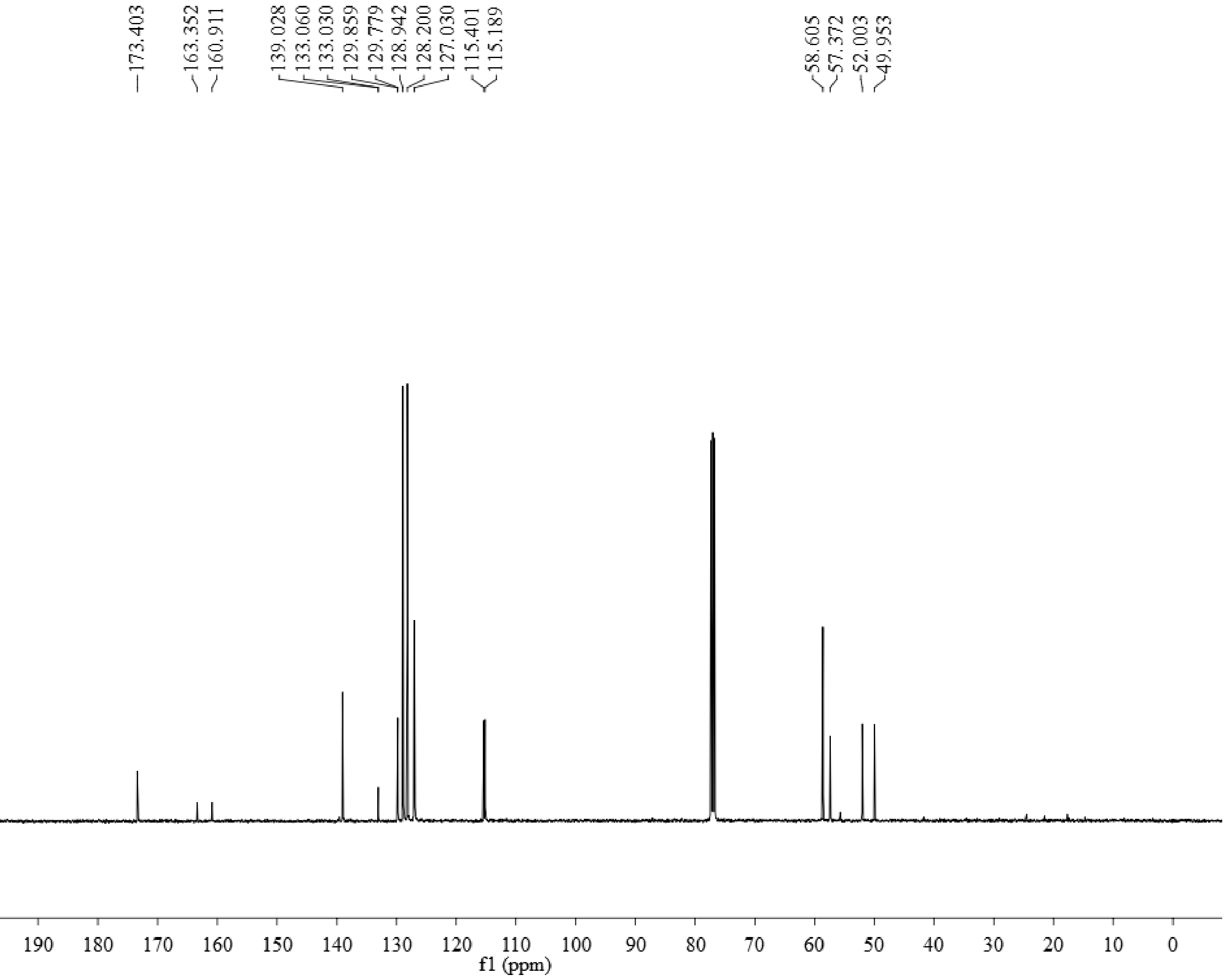

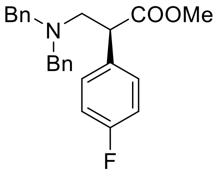

15, ^{19}F, $CDCl_3$, 282 MHz

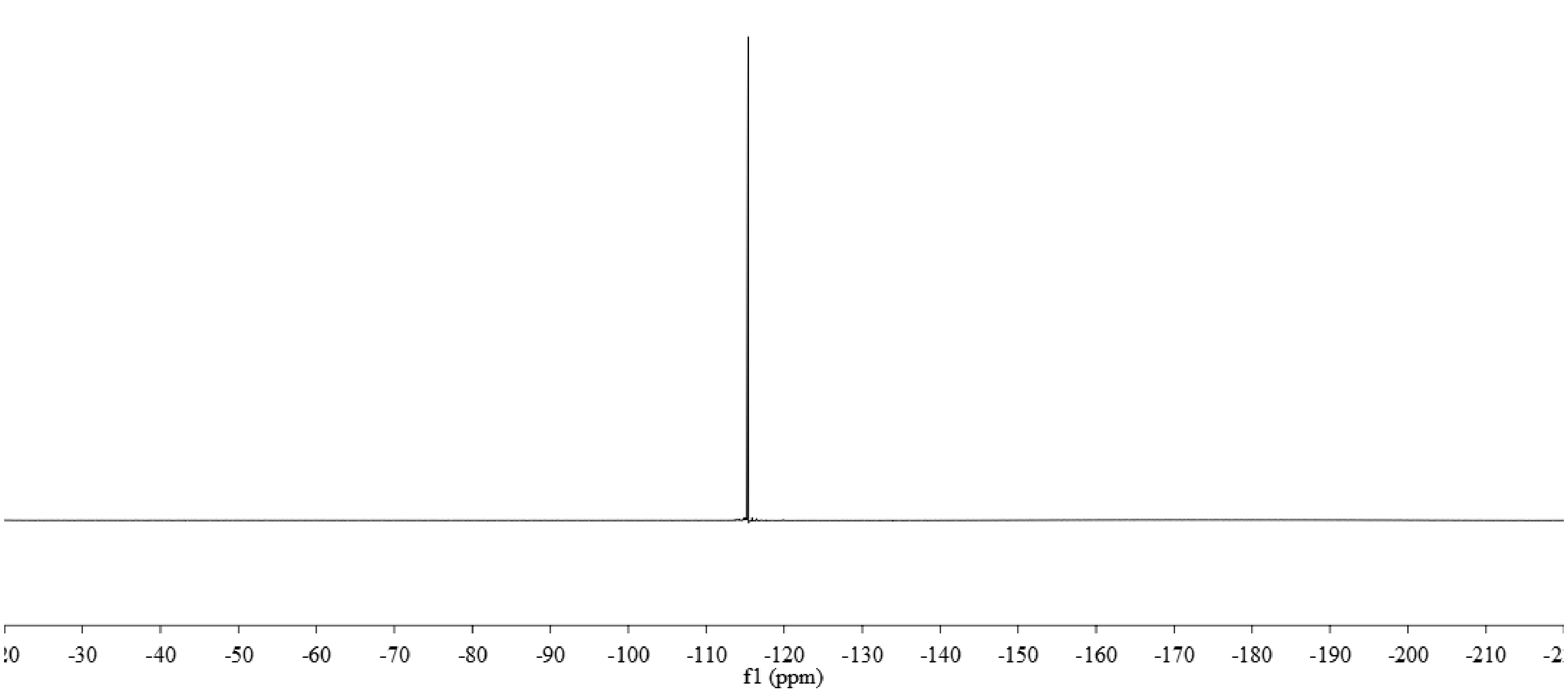

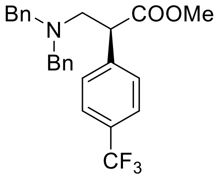

16, ^1H, $CDCl_3$, 400 MHz

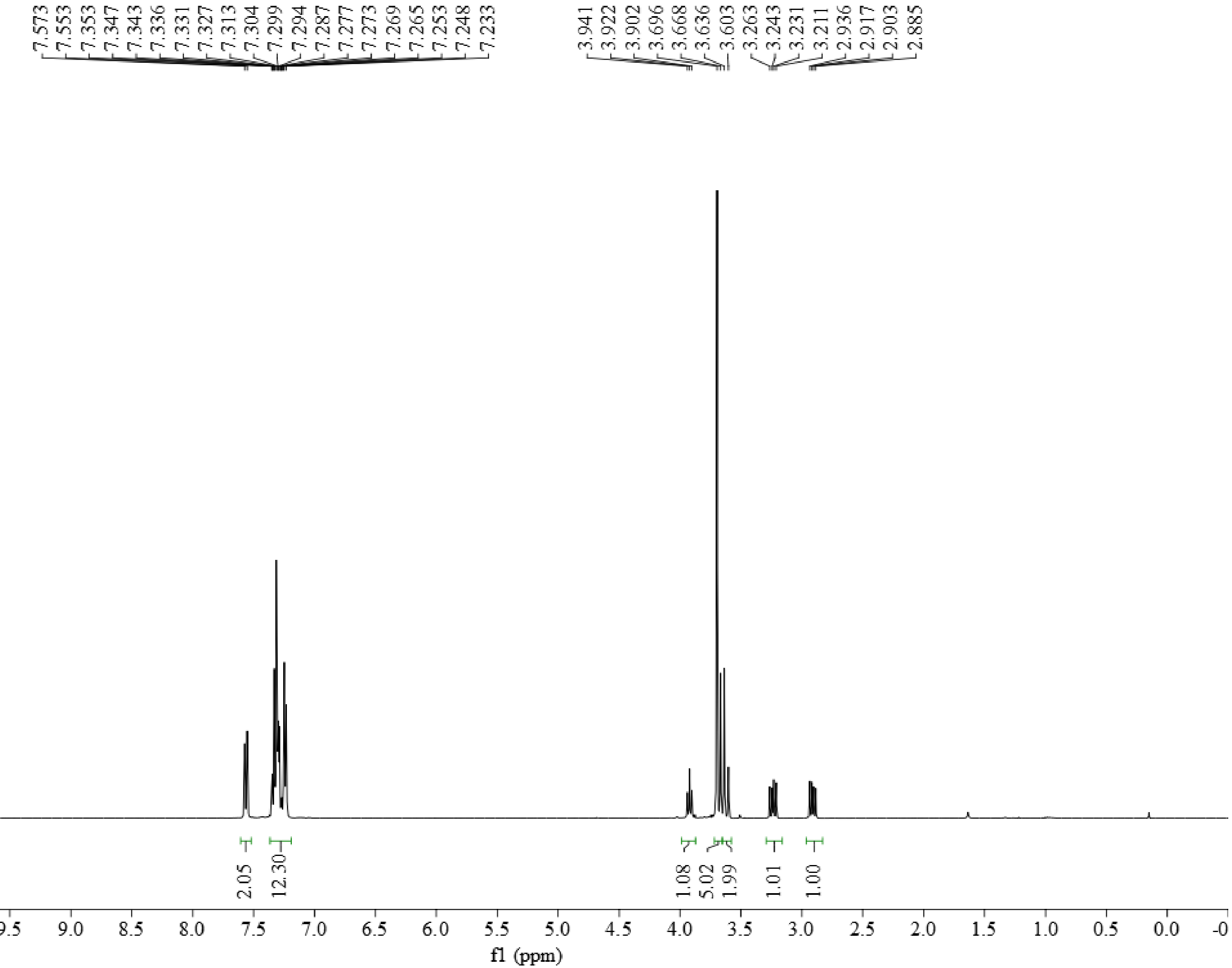

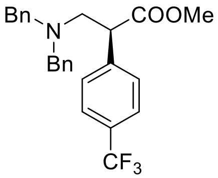

16, ^{13}C, $CDCl_3$, 101 MHz

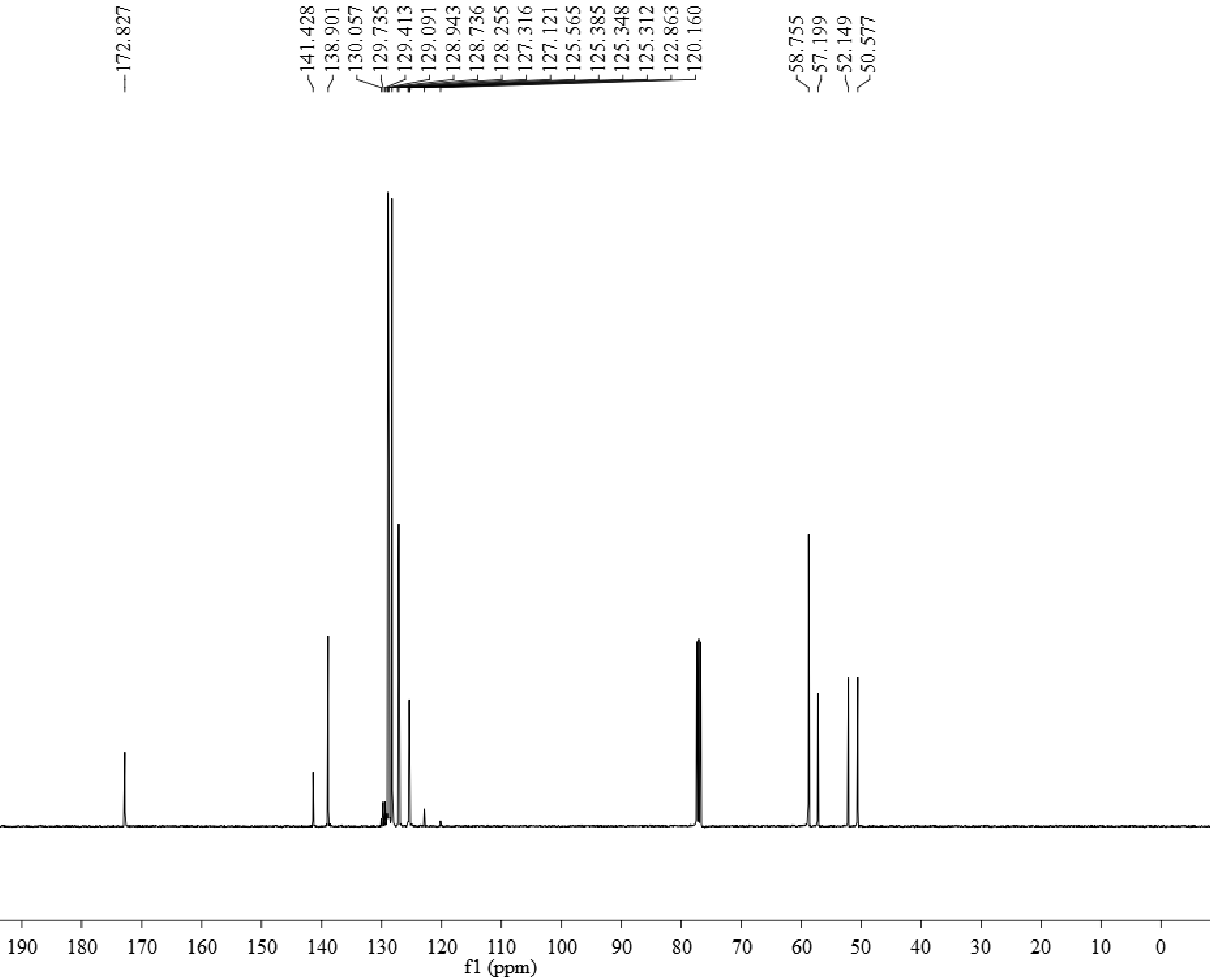

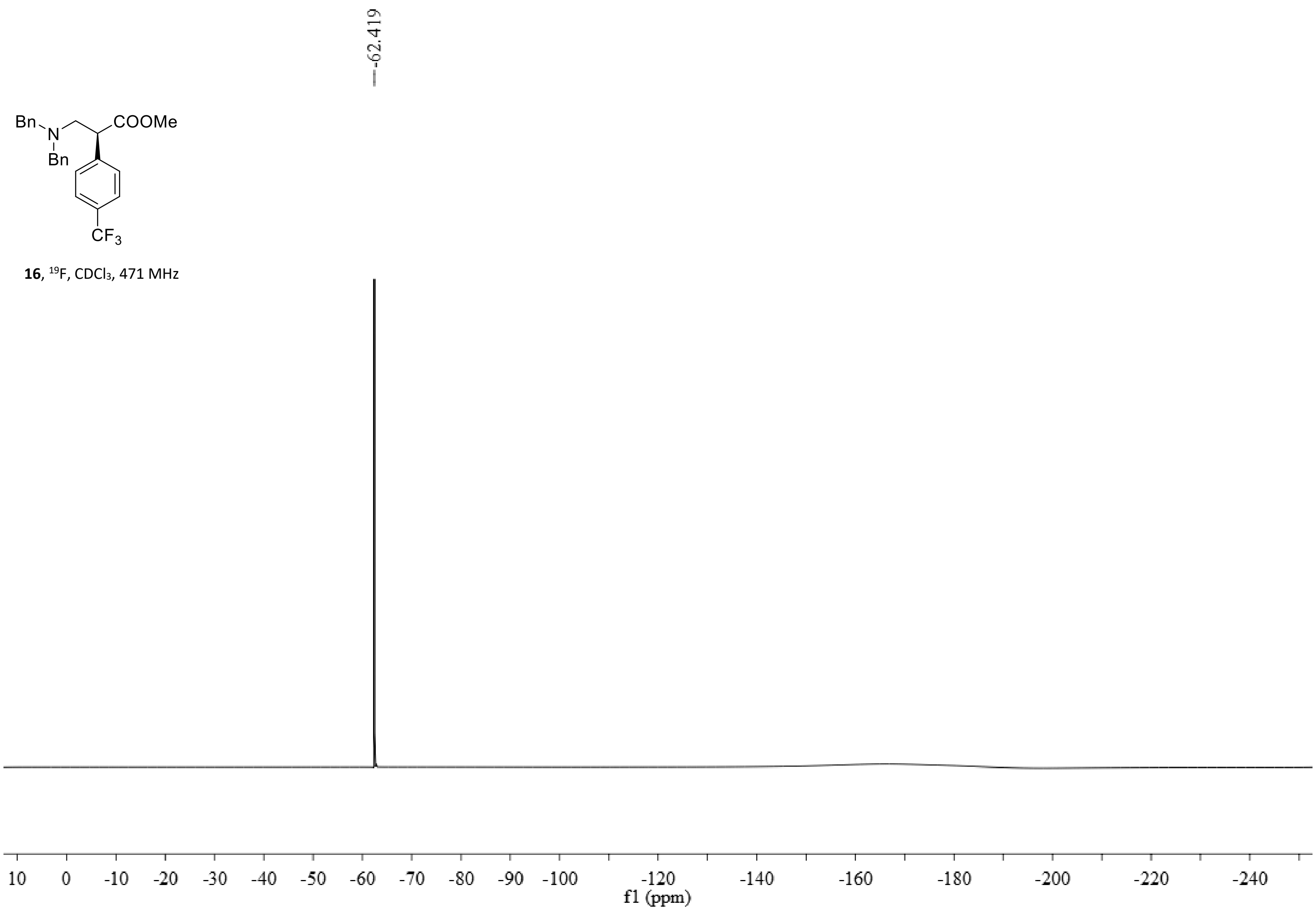

16, ^{19}F, $CDCl_3$, 471 MHz

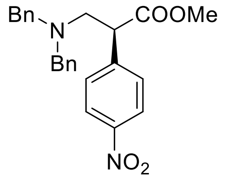

17, ^{1}H, $CDCl_3$, 400 MHz

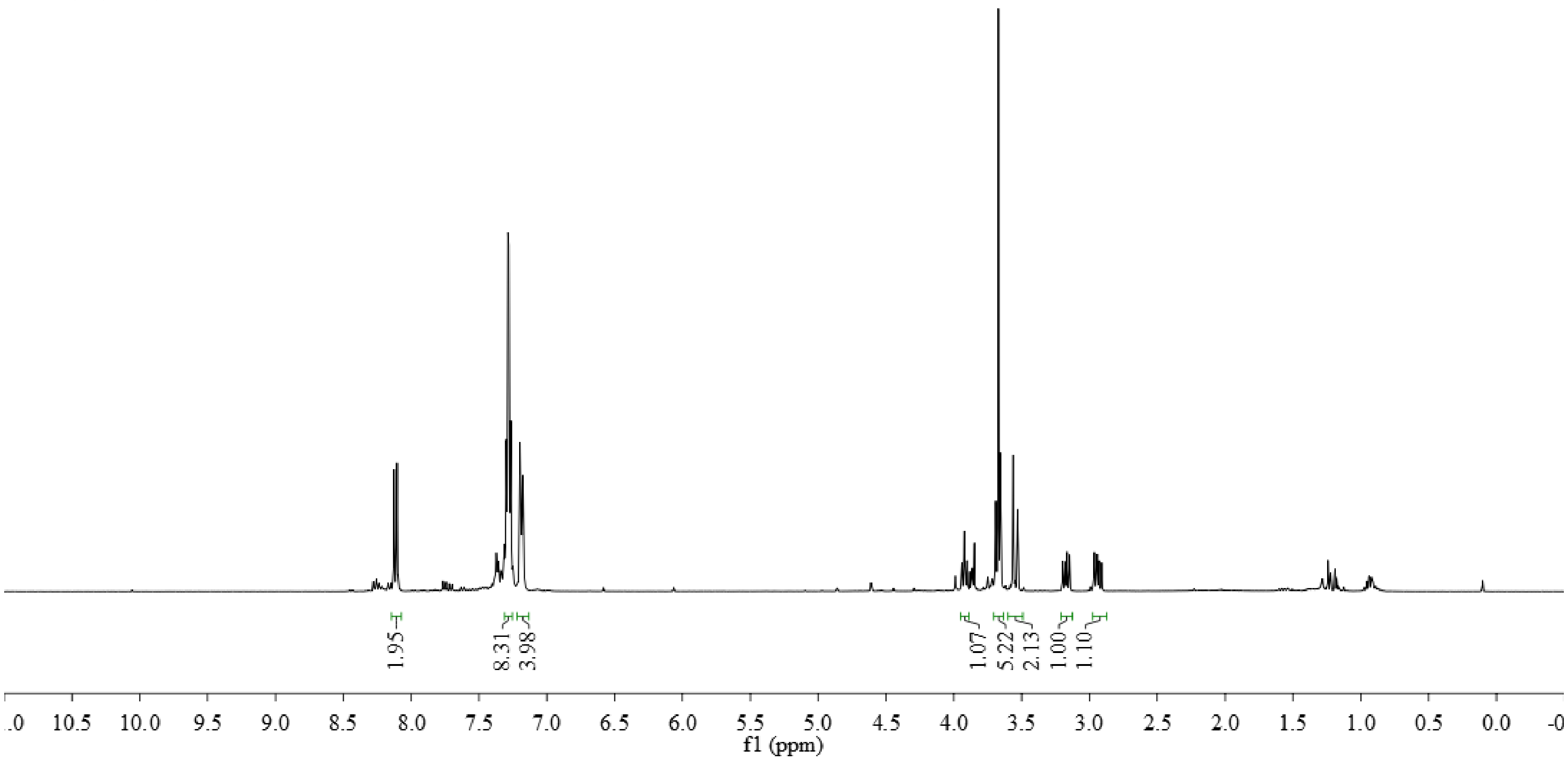

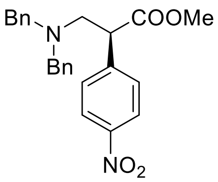

17, ^{13}C, $CDCl_3$, 101 MHz

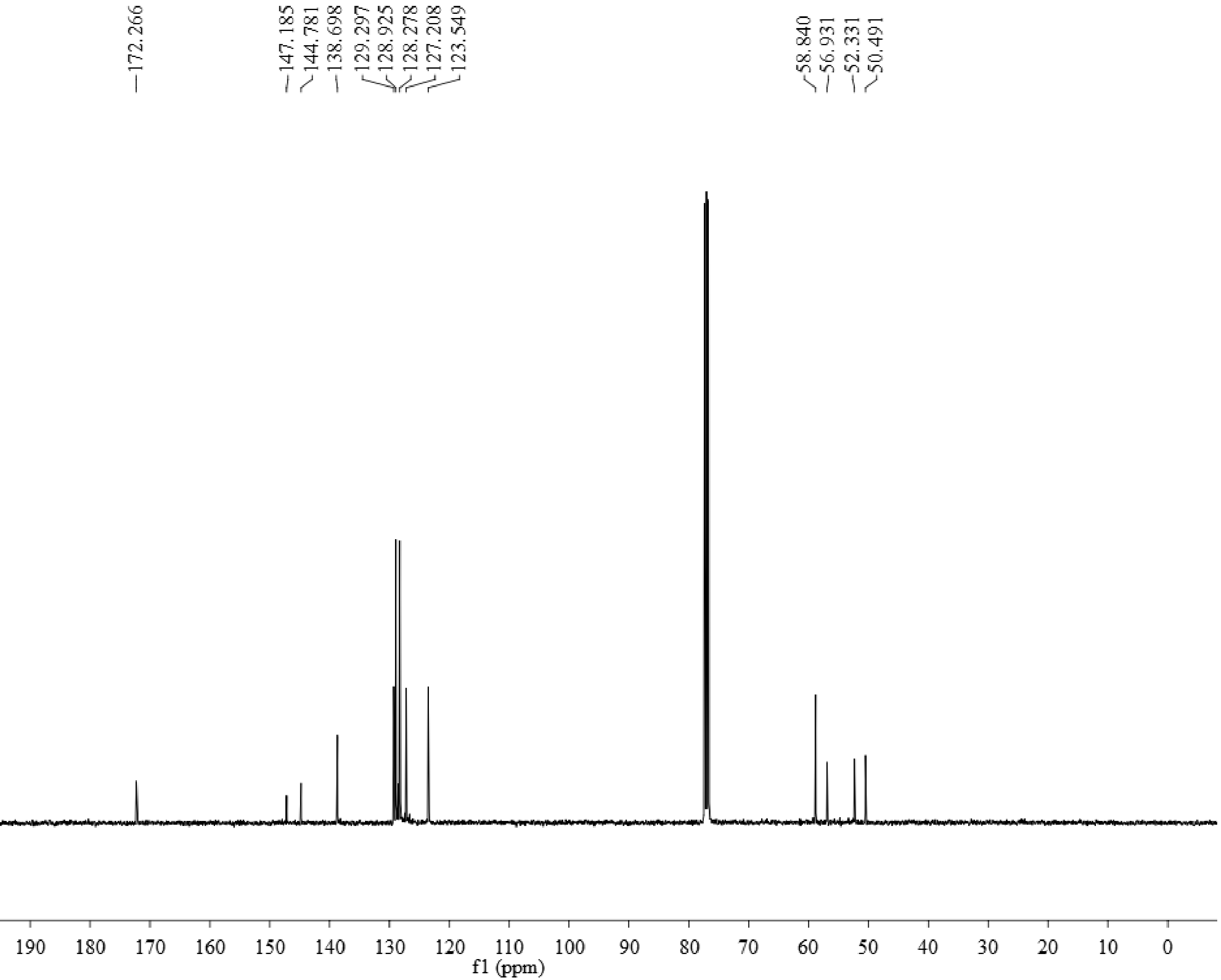

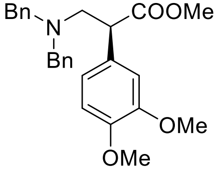

18, ^{1}H, $CDCl_3$, 400 MHz

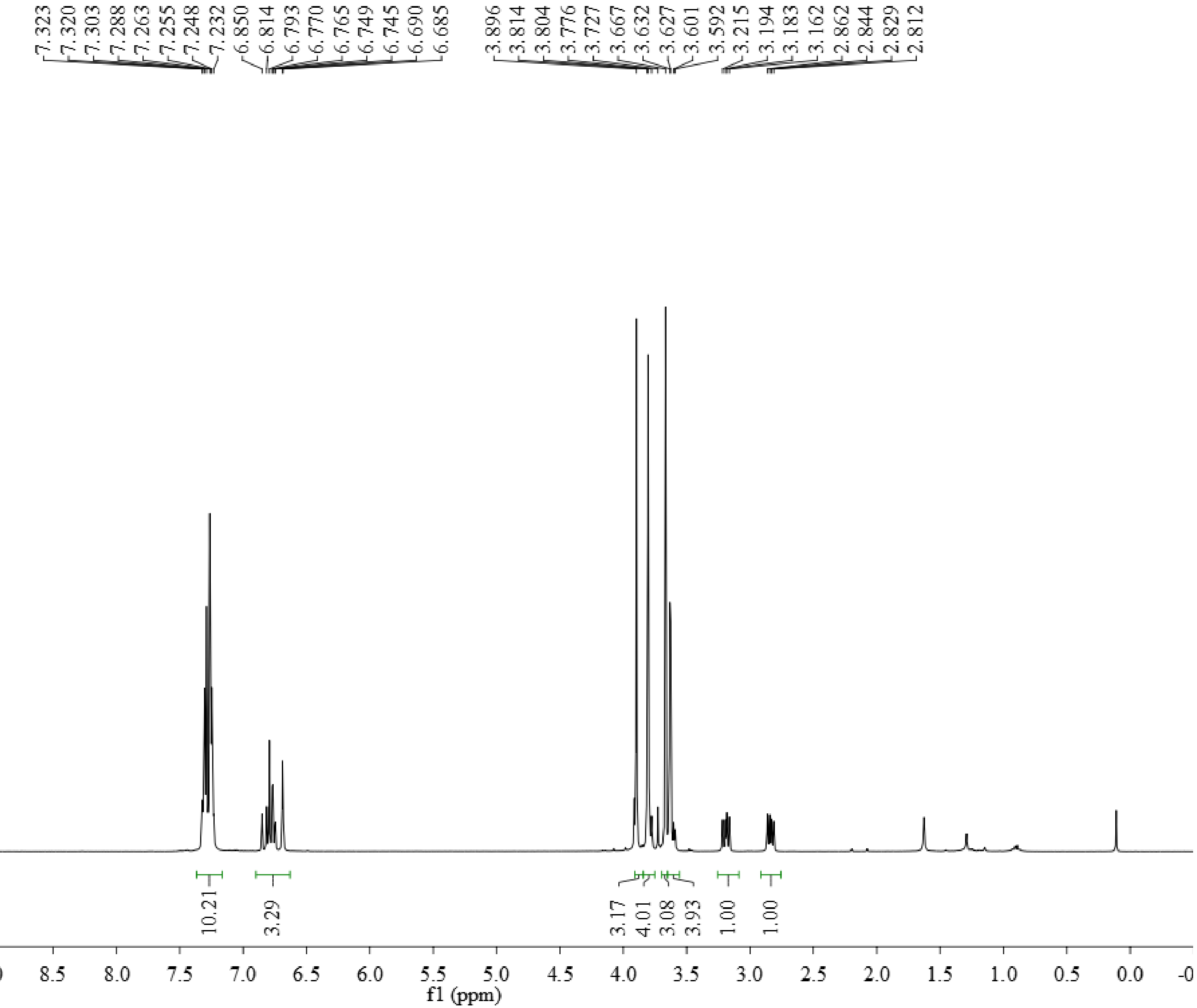

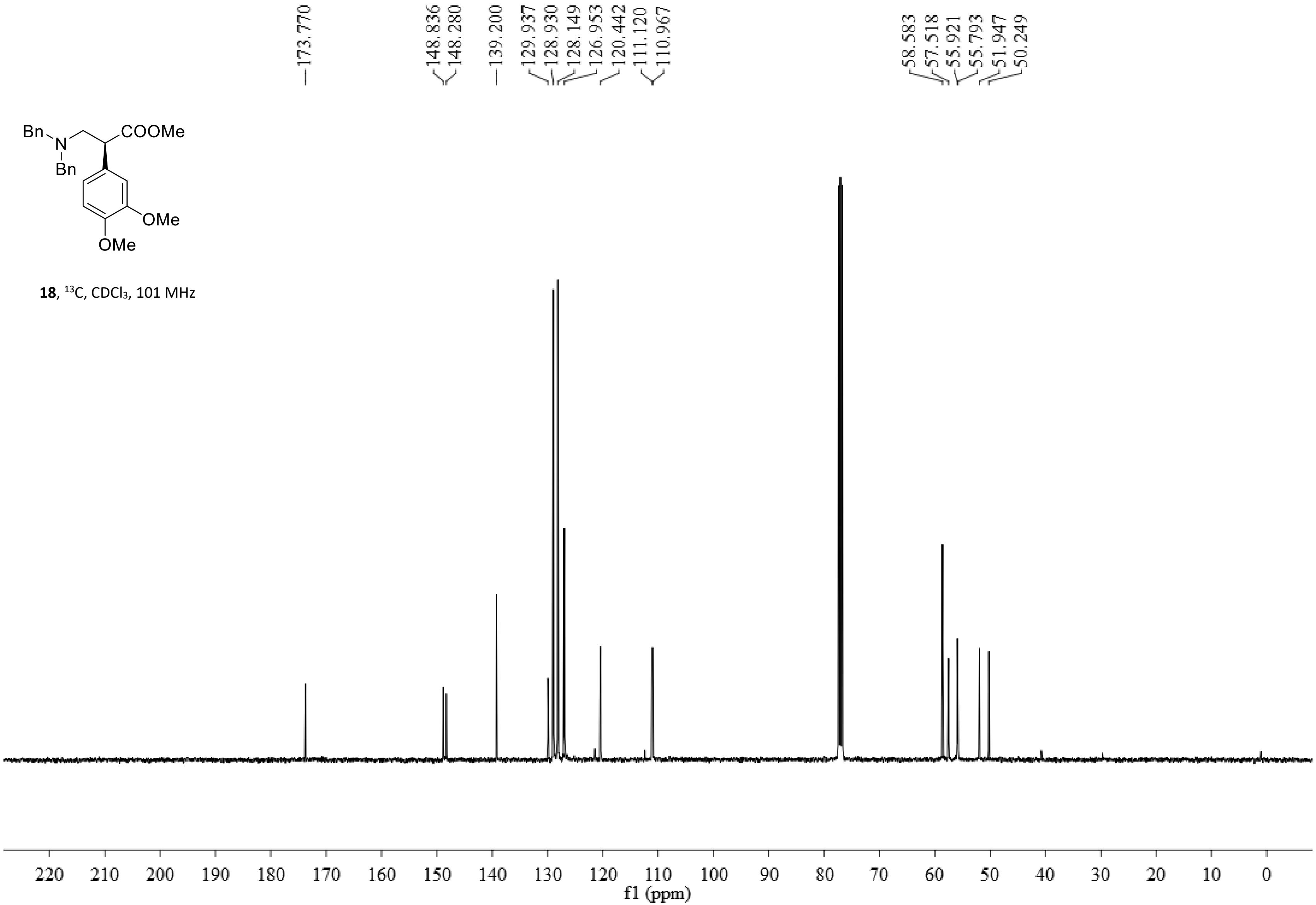

18, ^{13}C, $CDCl_3$, 101 MHz

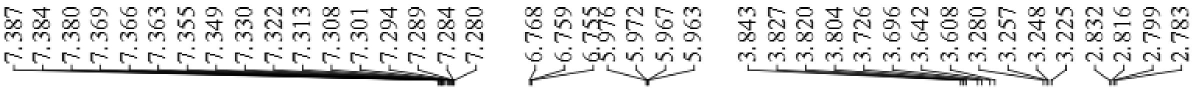

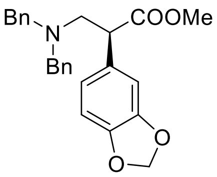

19, ^{1}H, $CDCl_3$, 400 MHz

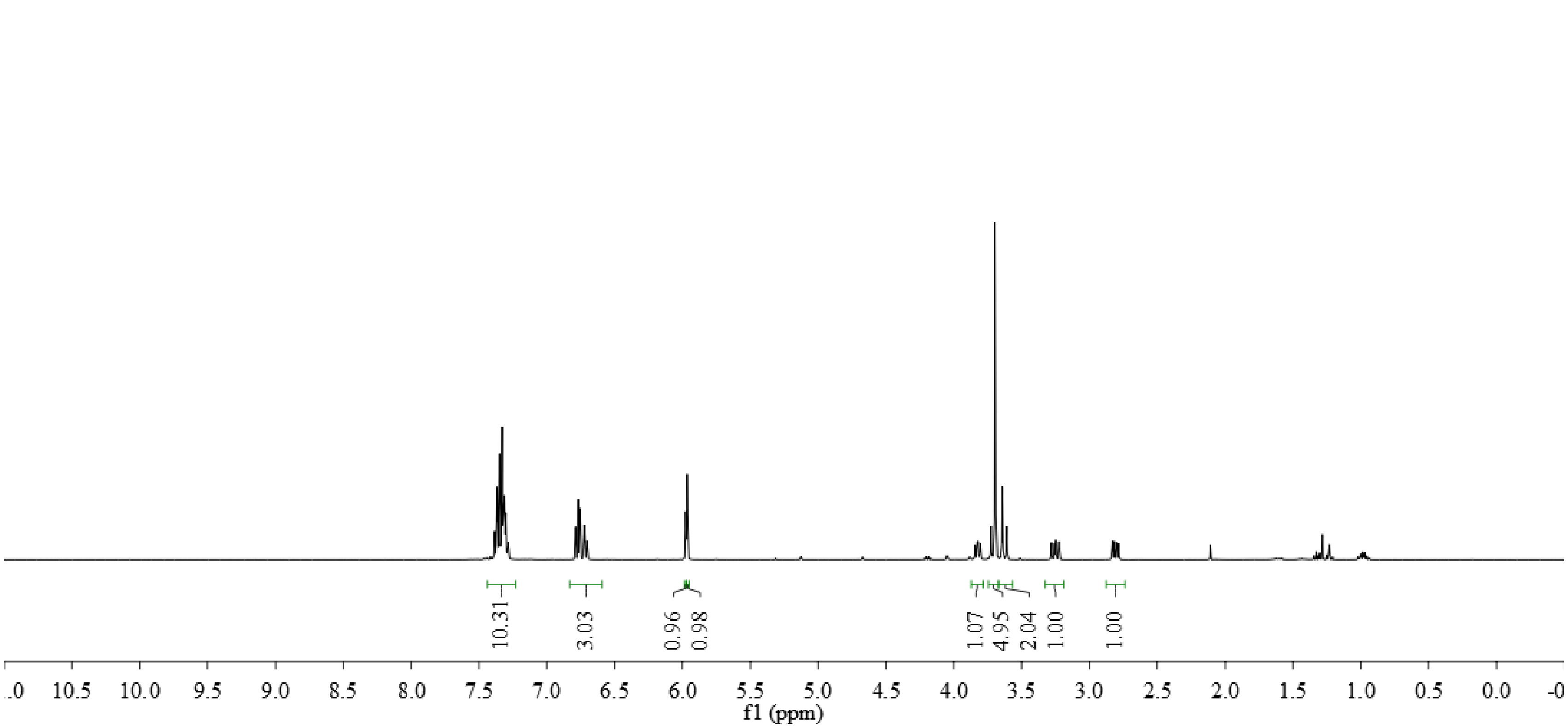

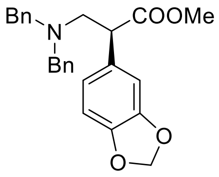

19, ^{13}C DEPTQ, $CDCl_3$, 126 MHz

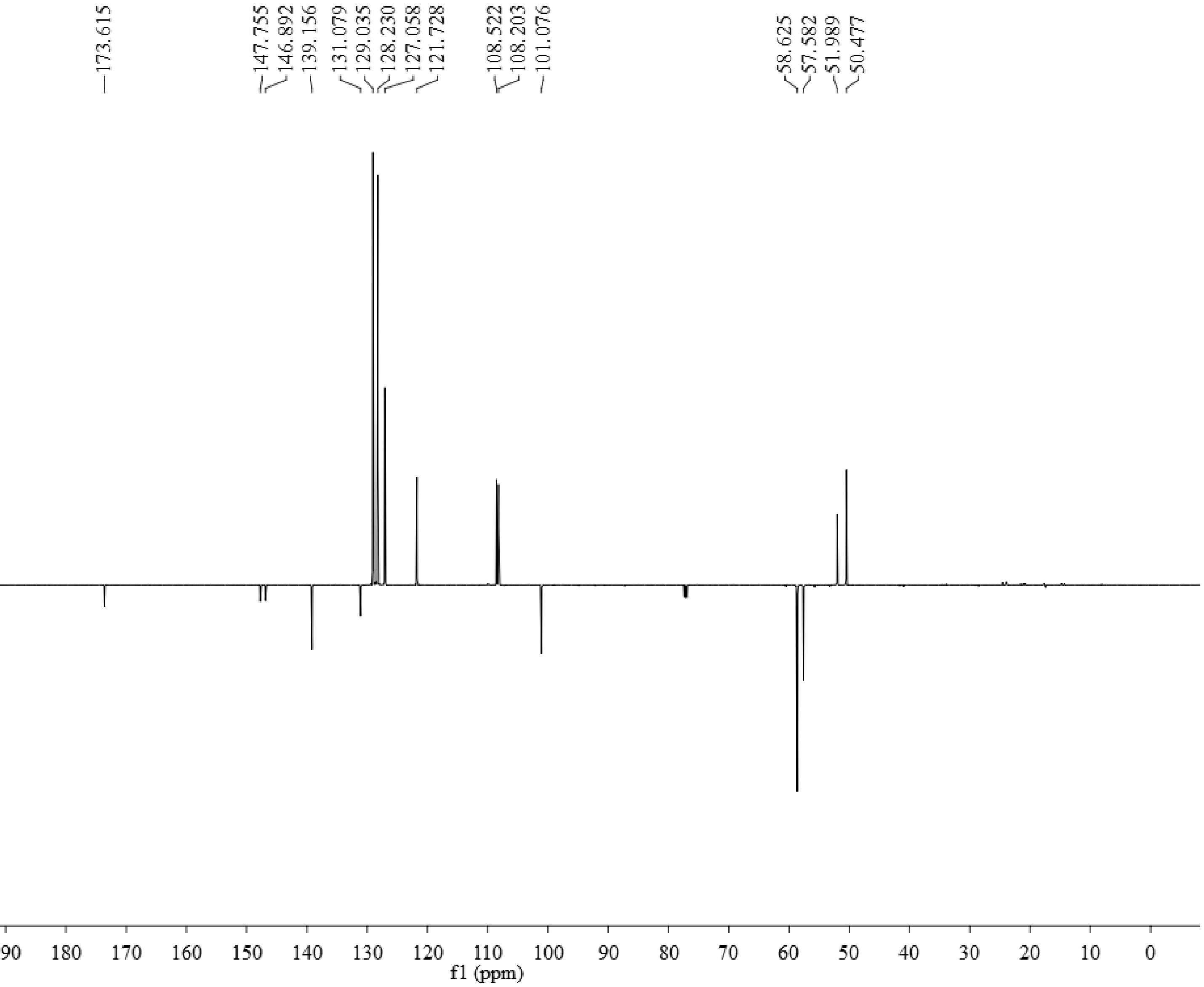

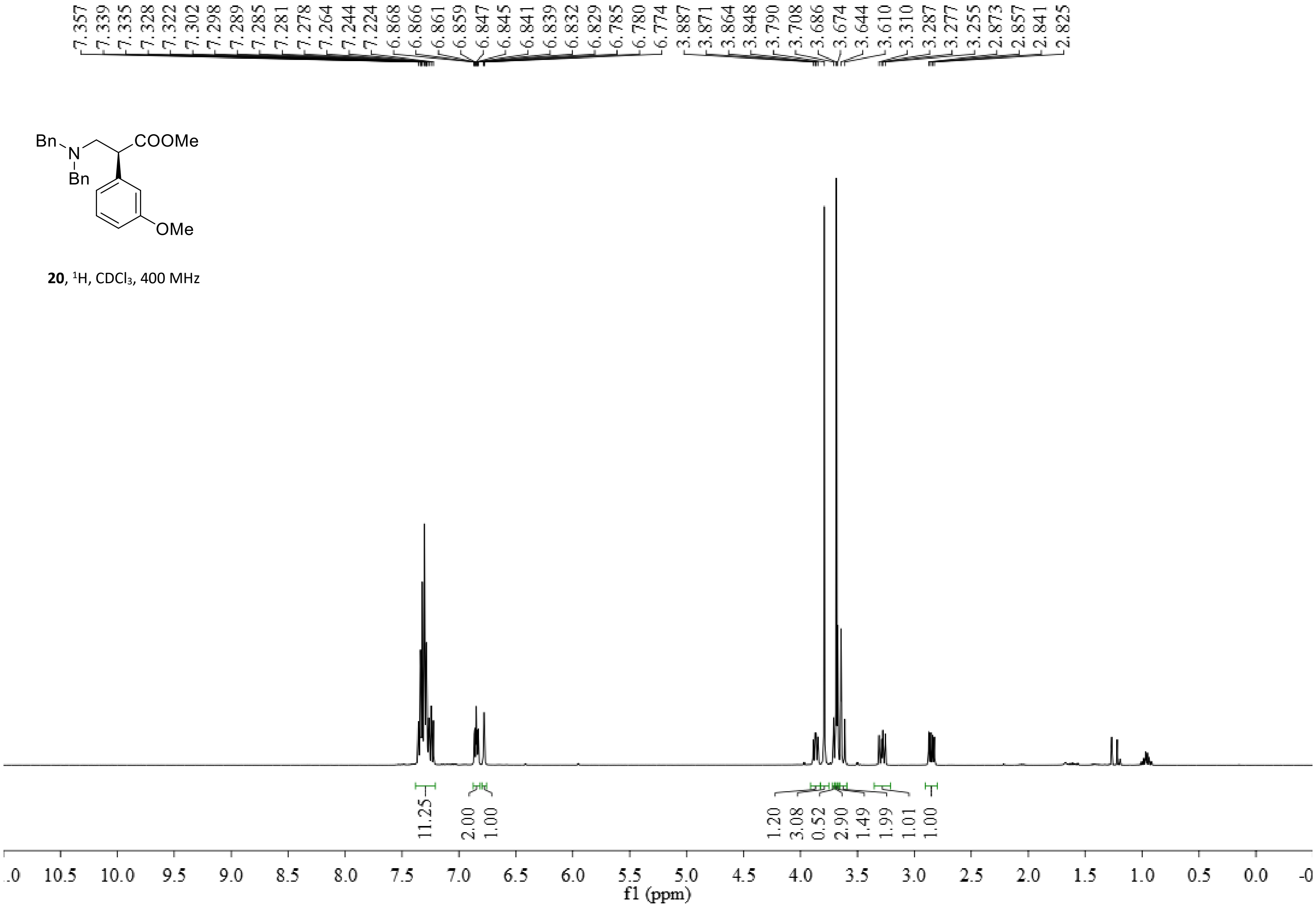

20, ^{1}H, $CDCl_3$, 400 MHz

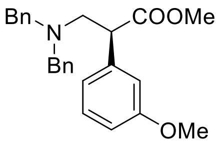

20, ^{13}C DEPTQ, $CDCl_3$, 101 MHz

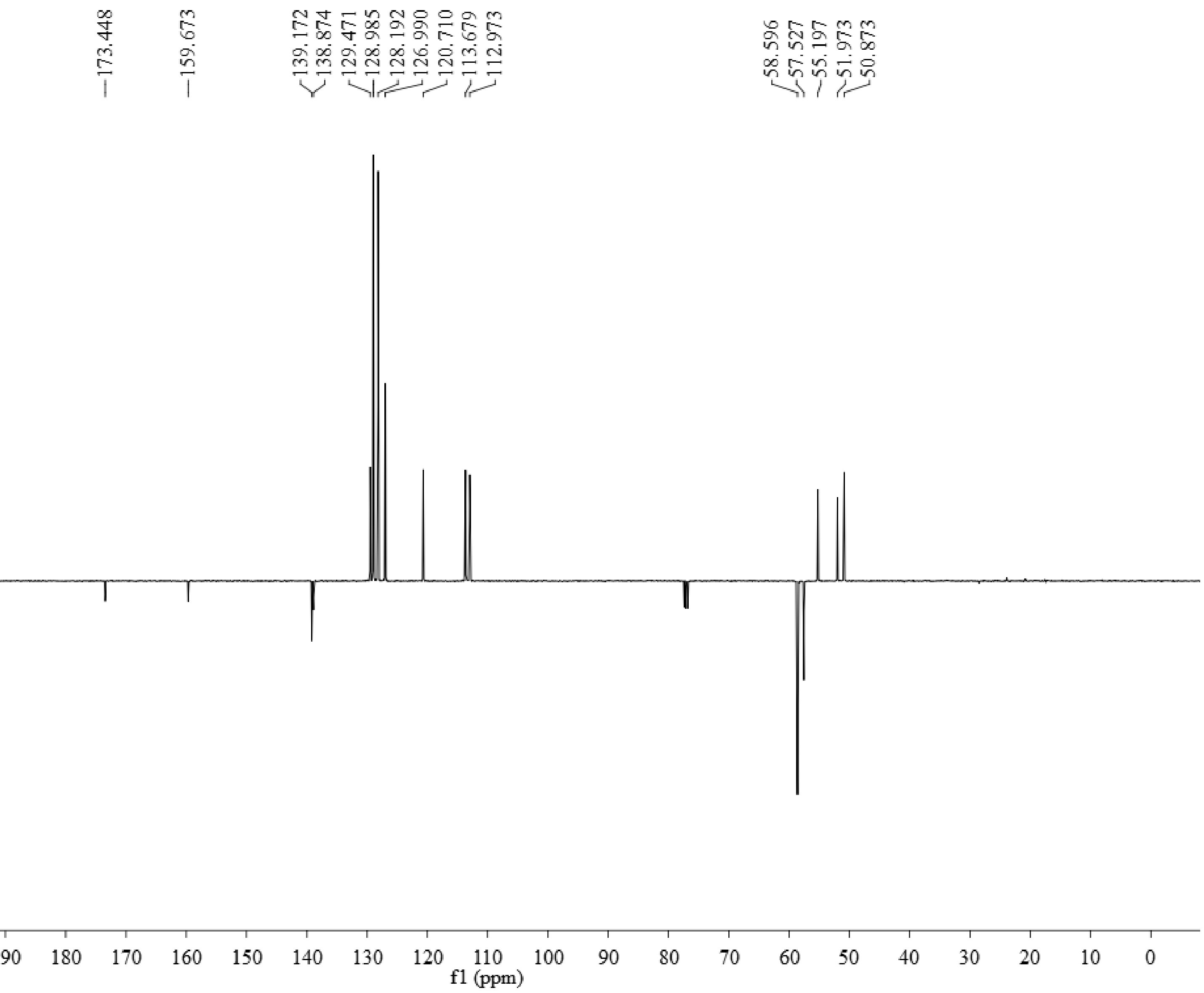

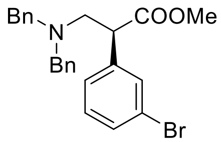

21, ¹H, CDCl$_3$, 500 MHz

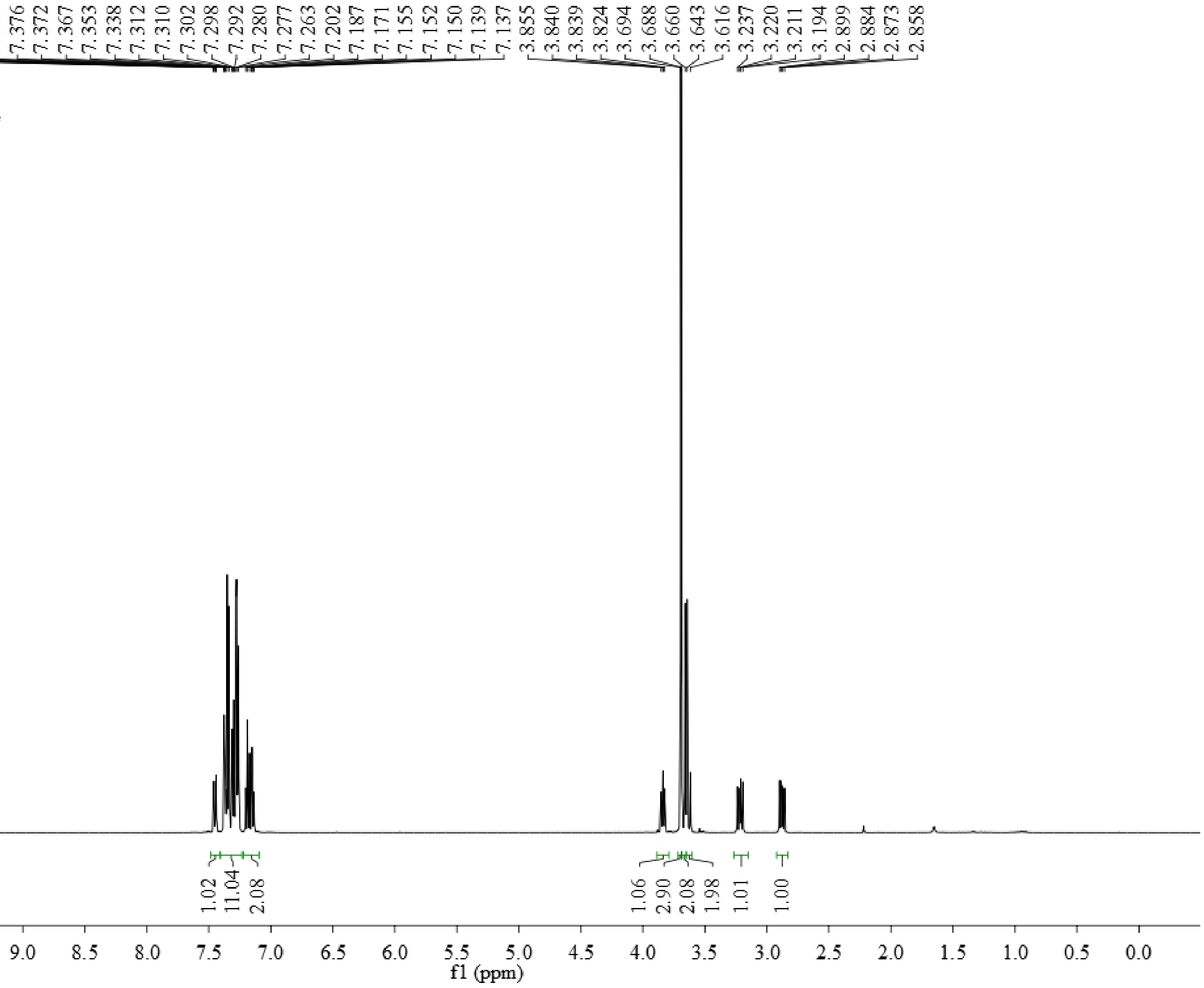

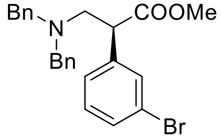

21, ^{13}C DEPTQ, $CDCl_3$, 126 MHz

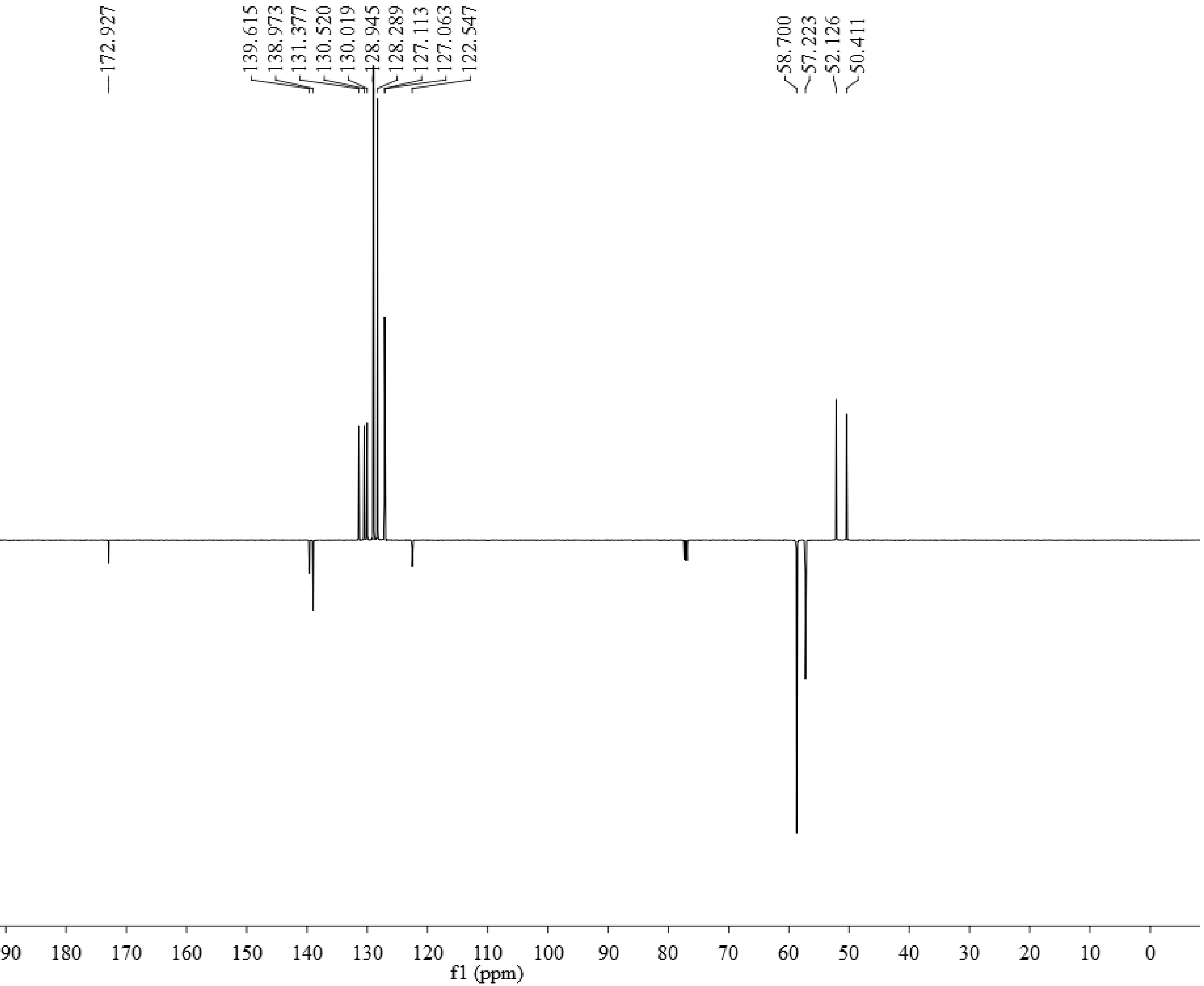

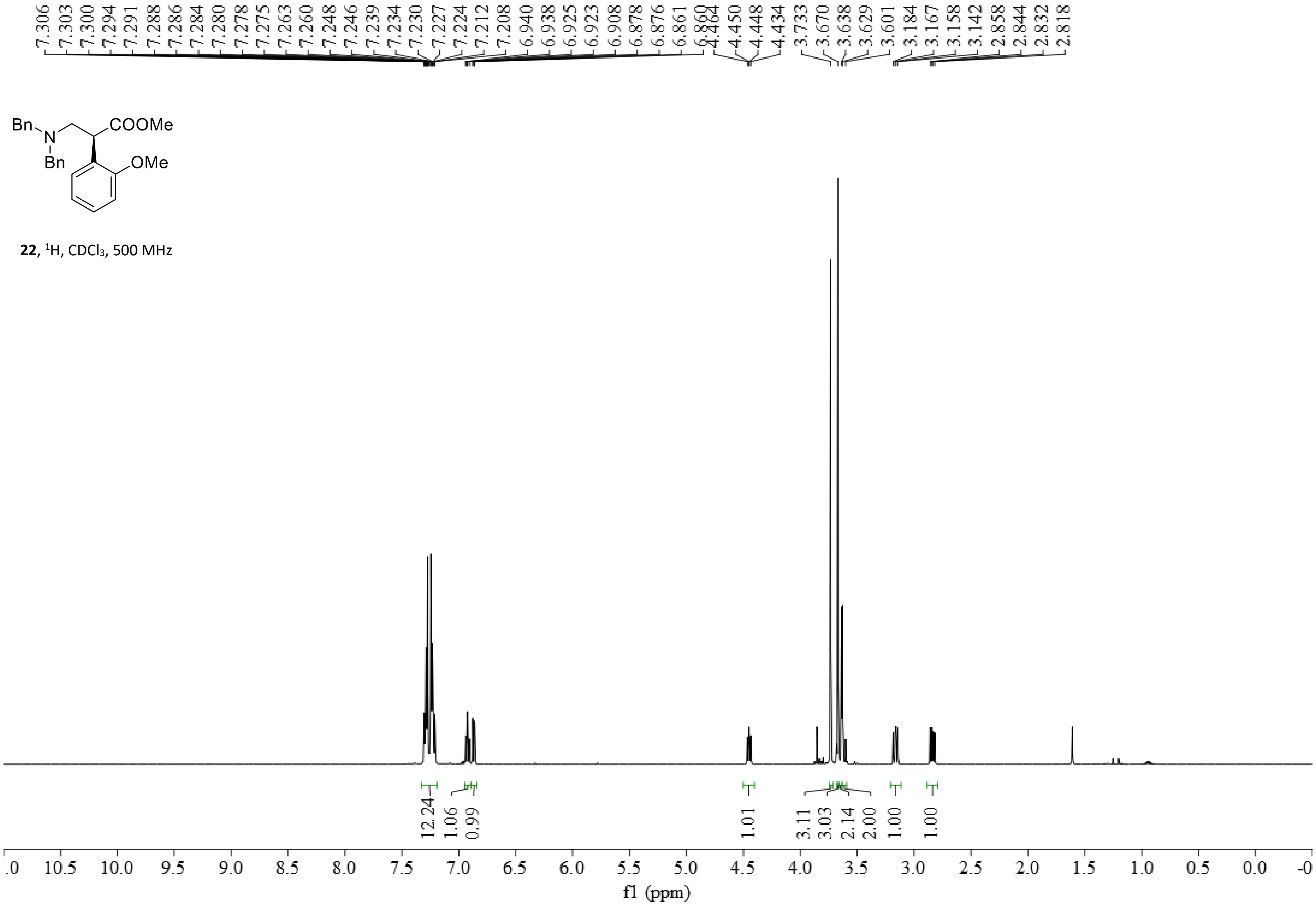

22, ^{1}H, $CDCl_3$, 500 MHz

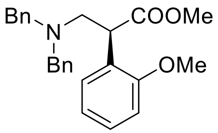

22, ^{13}C DEPTQ, $CDCl_3$, 101 MHz

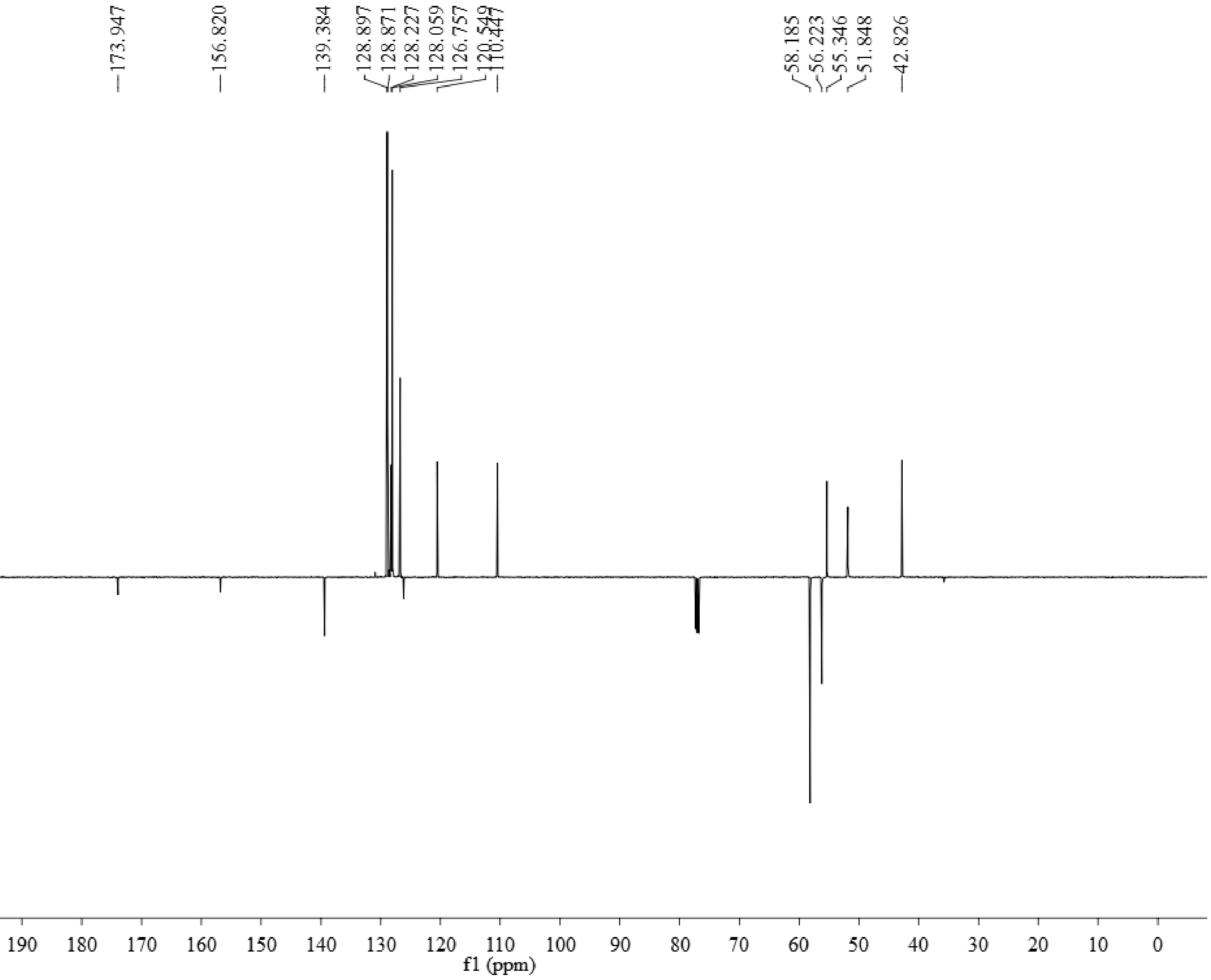

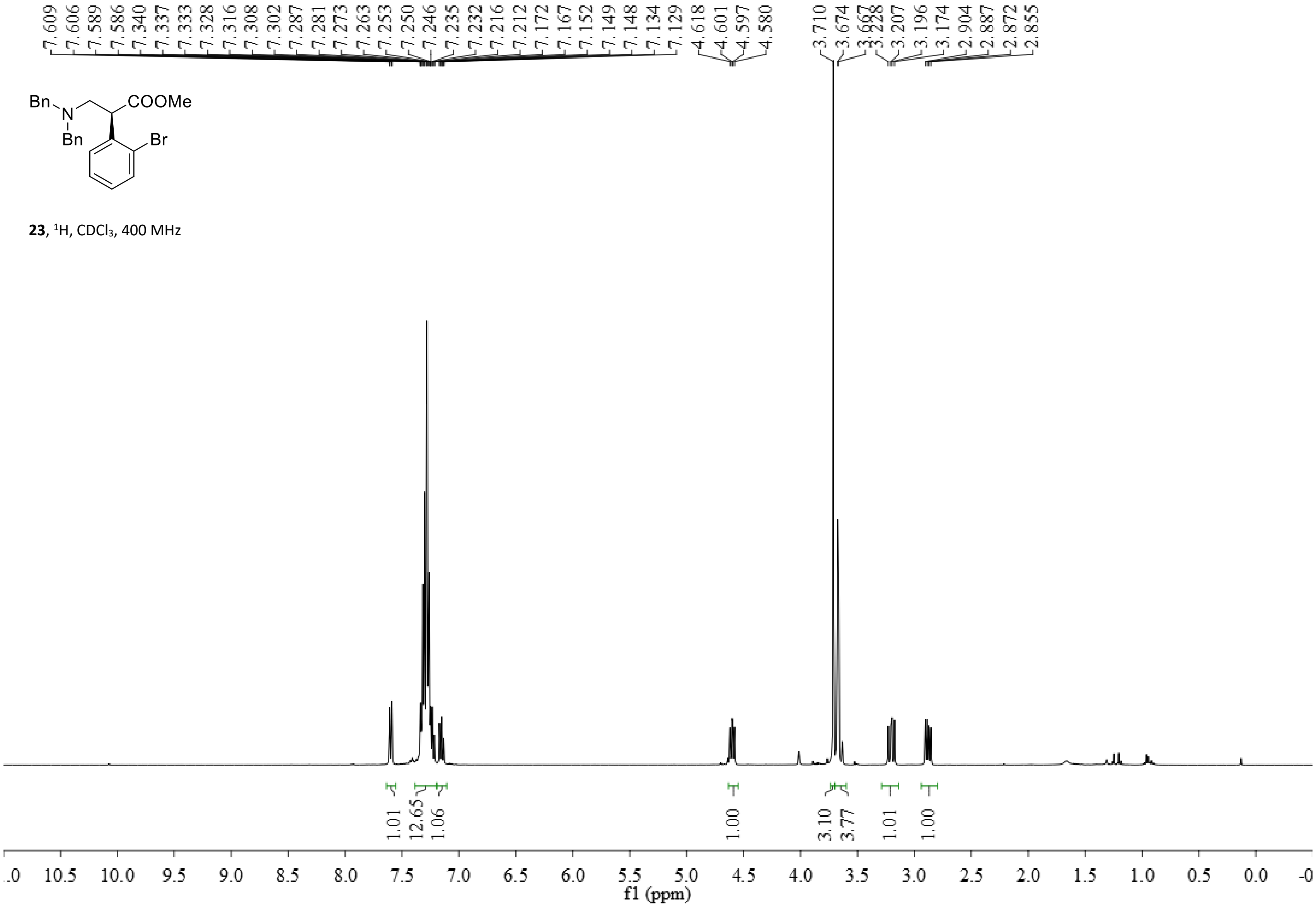

23, ^{1}H, $CDCl_3$, 400 MHz

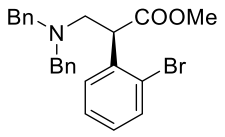

23, ^{13}C, $CDCl_3$, 101 MHz

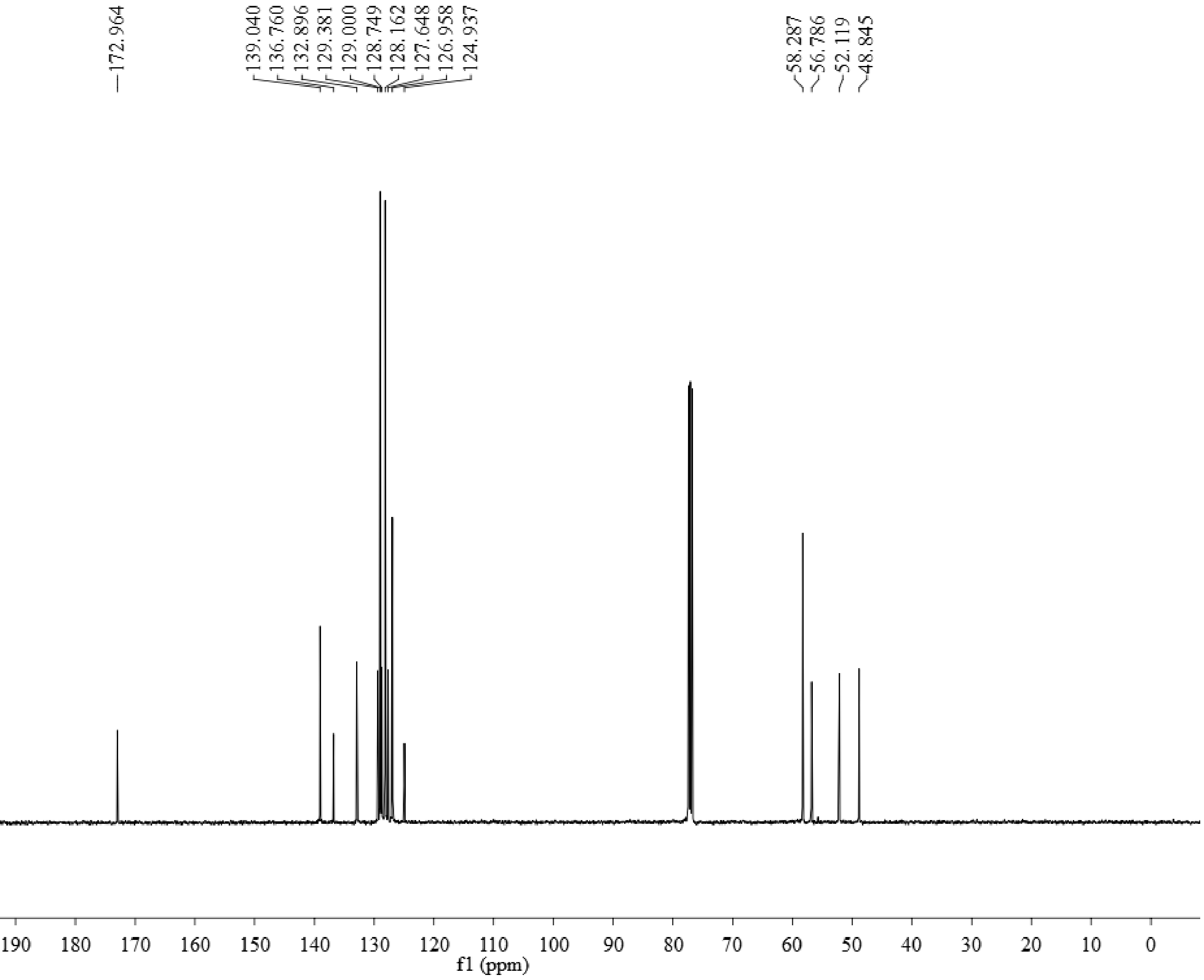

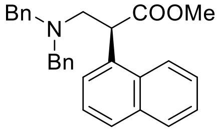

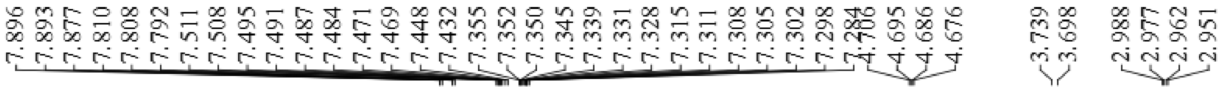

24, ^{1}H, $CDCl_3$, 500 MHz

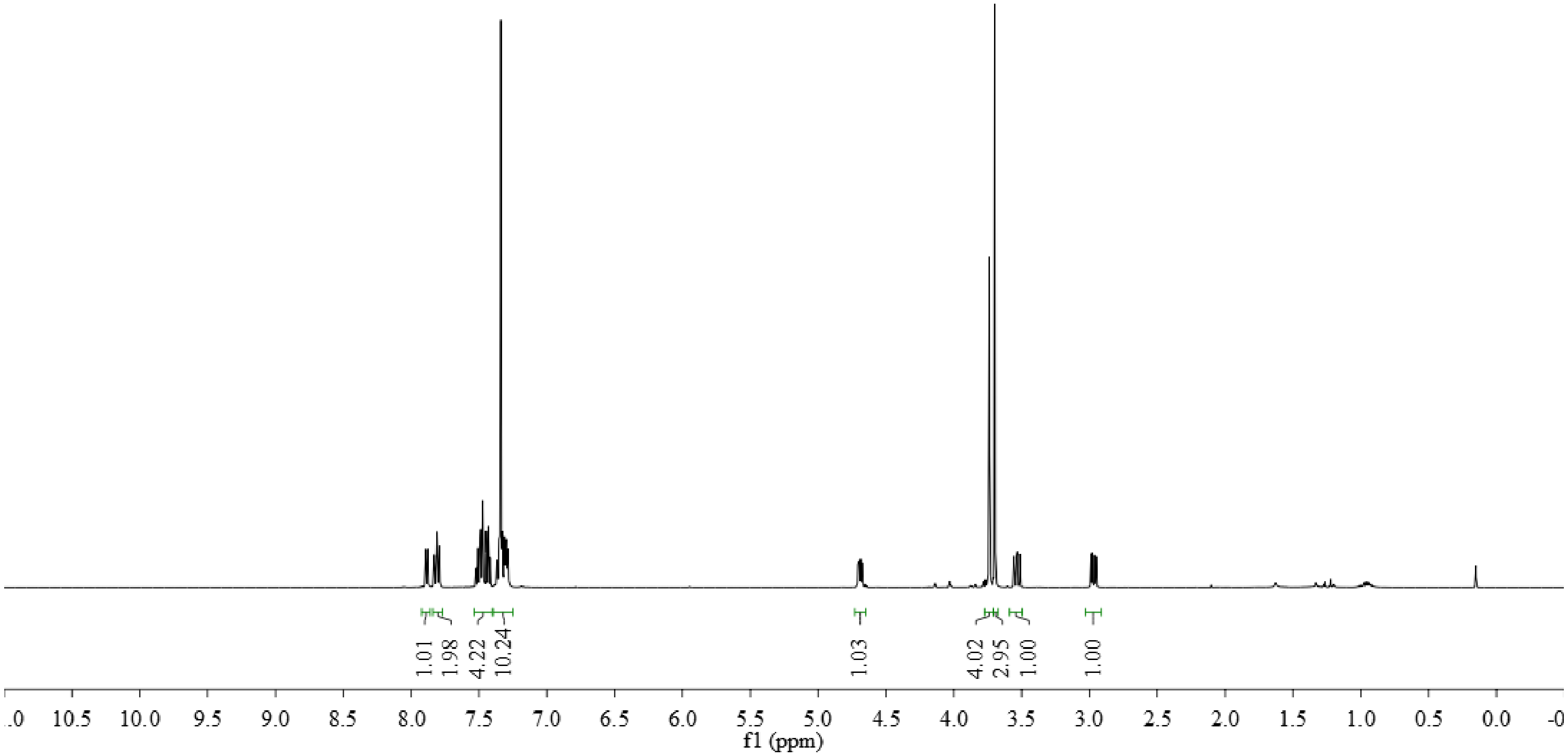

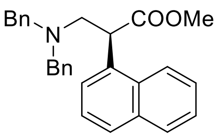

24, ^{13}C DEPTQ, $CDCl_3$, 126 MHz

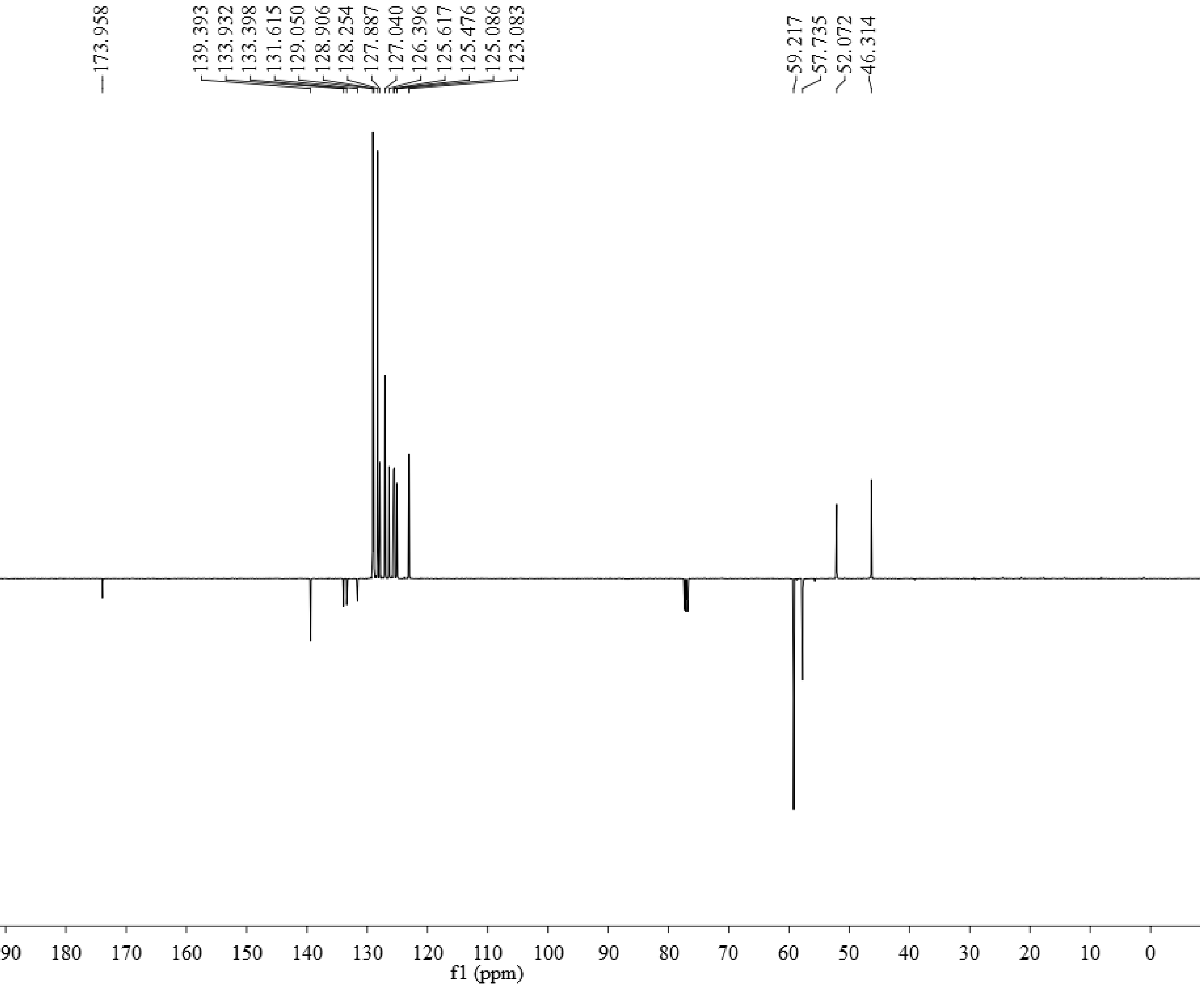

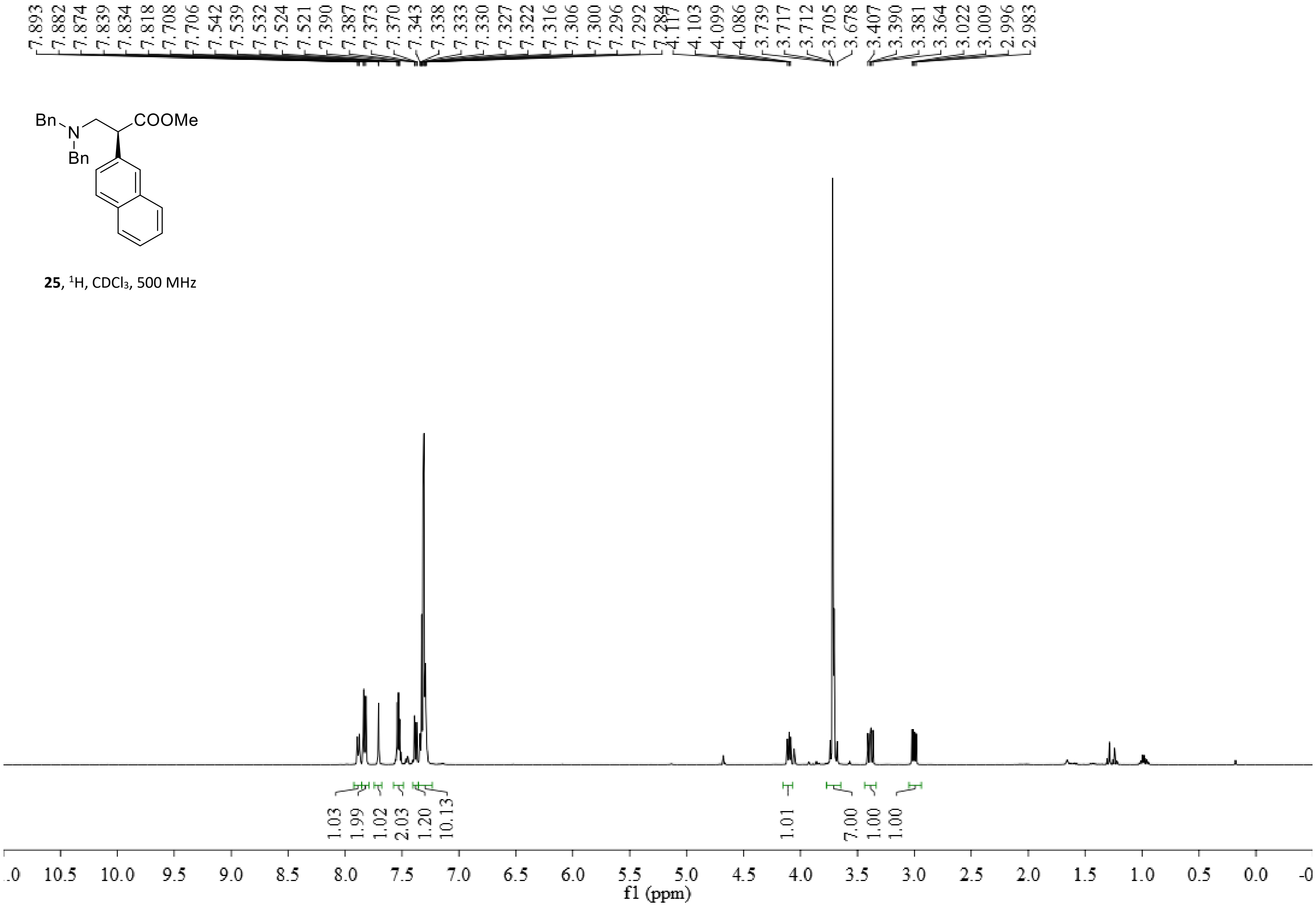

25, ^{1}H, $CDCl_3$, 500 MHz

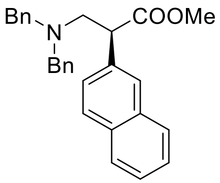

25, ^{13}C DEPTQ, $CDCl_3$, 126 MHz

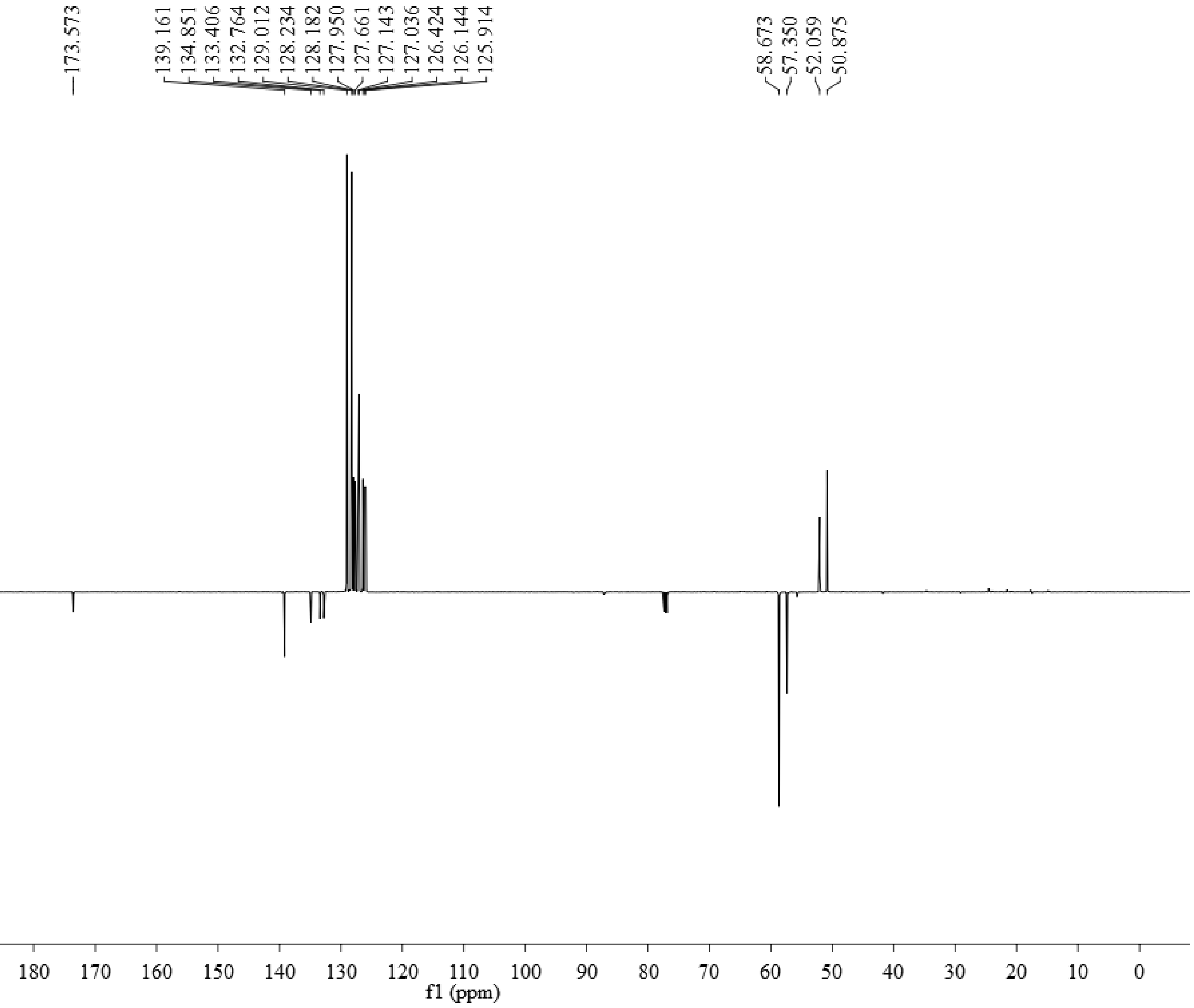

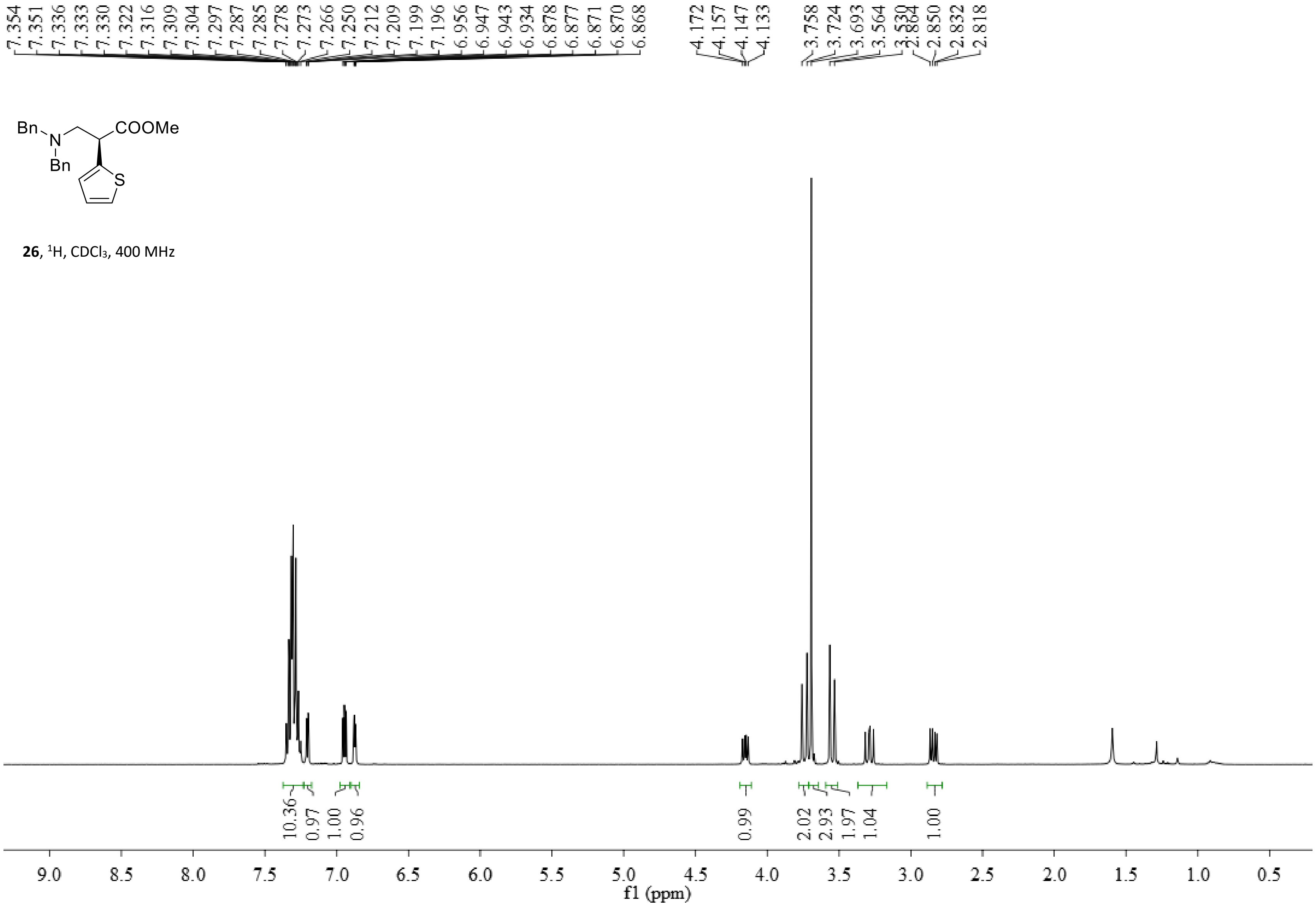

26, ^{1}H, $CDCl_3$, 400 MHz

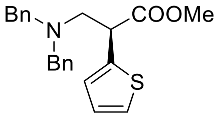

26, ^{13}C, $CDCl_3$, 101 MHz

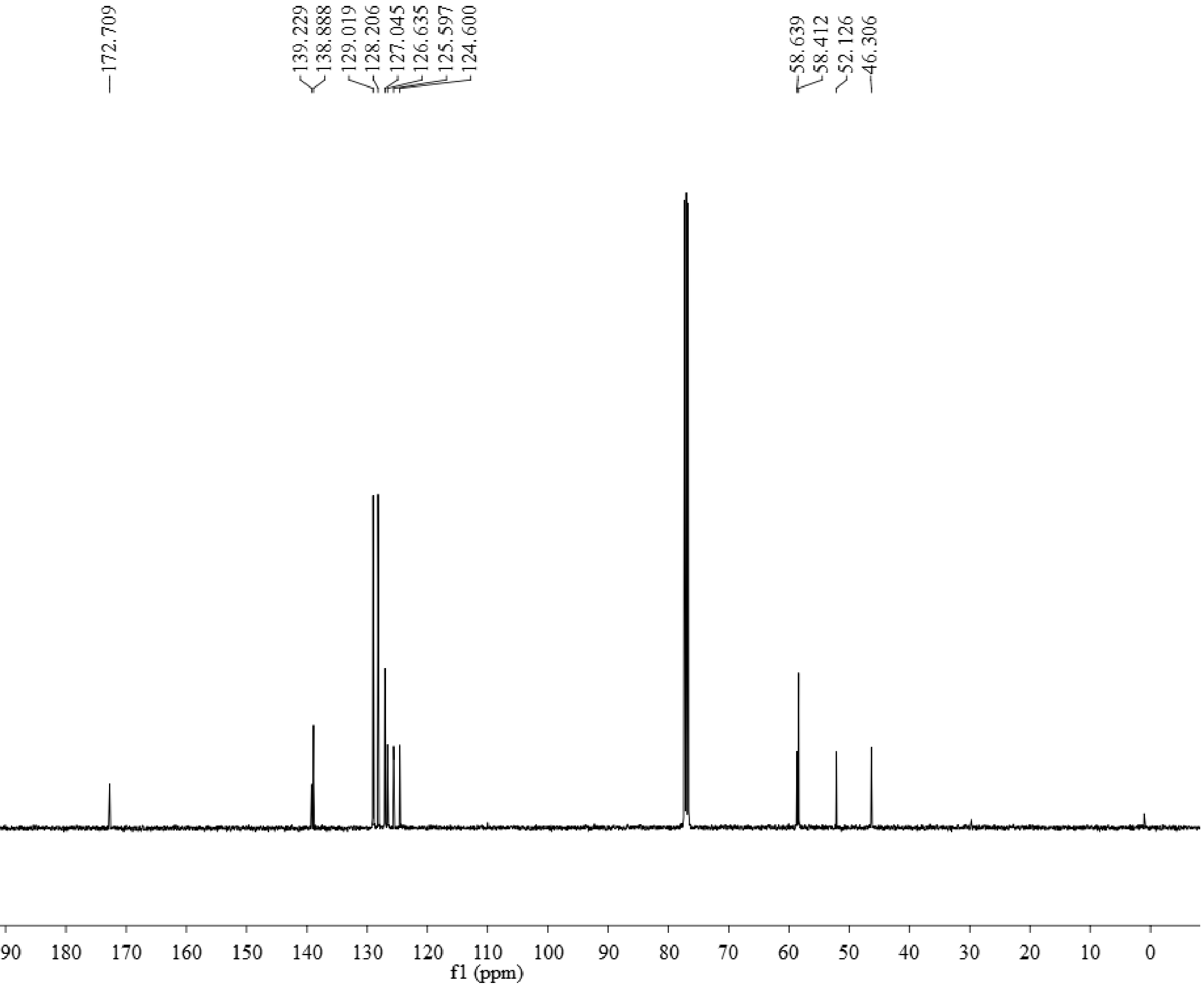

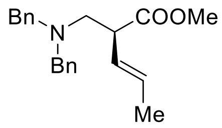

27, ^{1}H, $CDCl_3$, 500 MHz

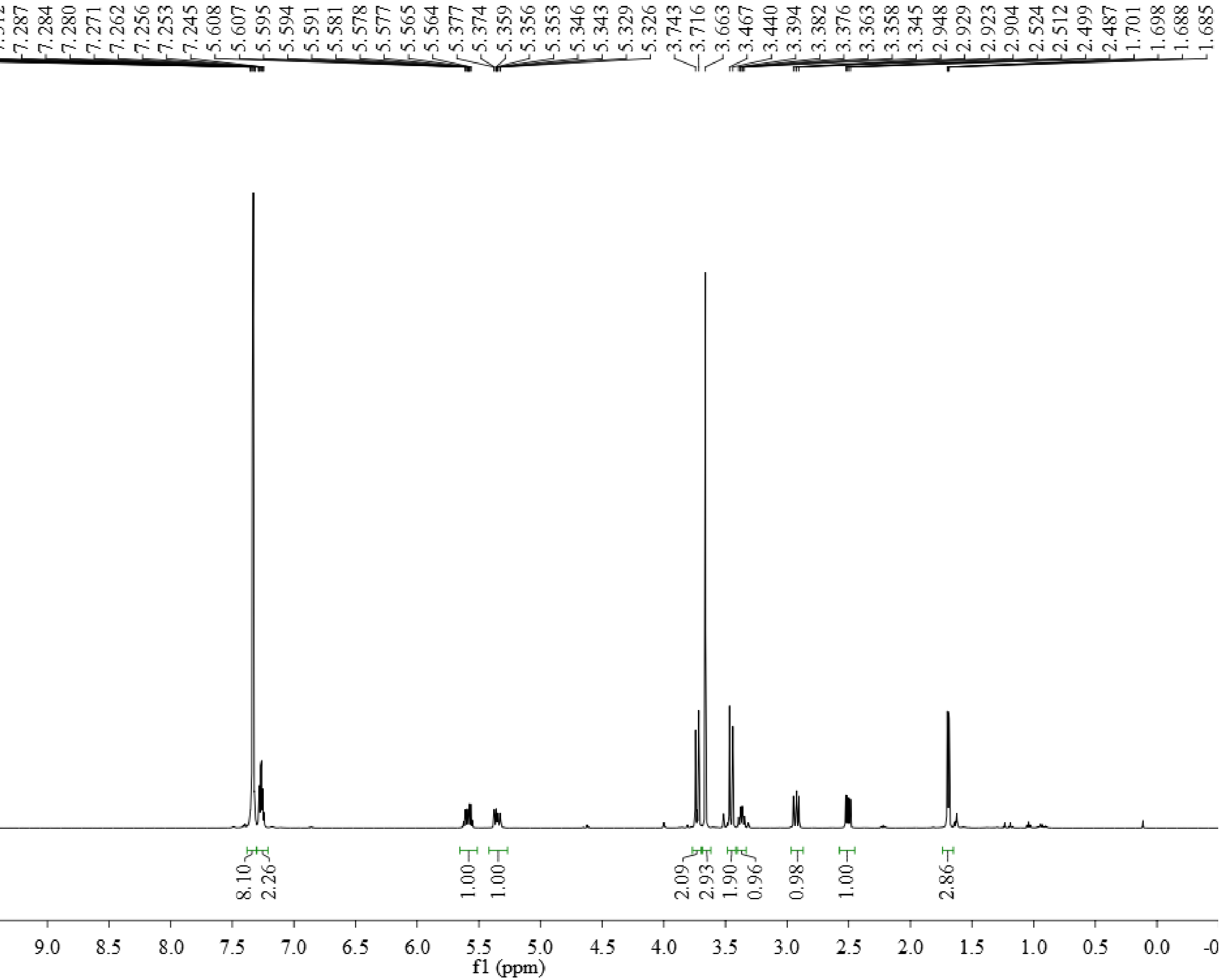

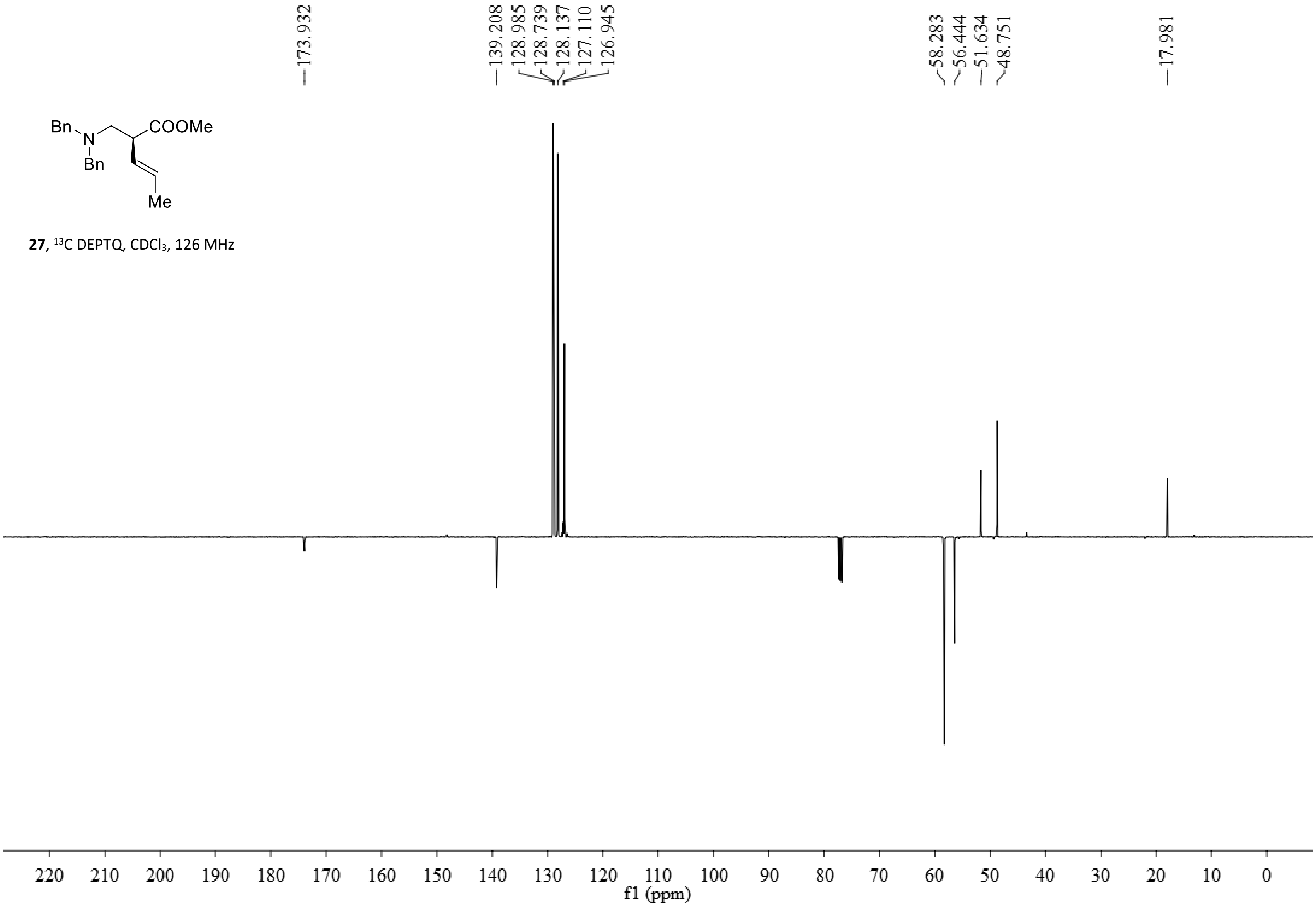

27, ^{13}C DEPTQ, $CDCl_3$, 126 MHz

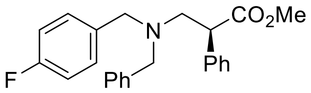

28, ^{1}H, $CDCl_3$, 400 MHz

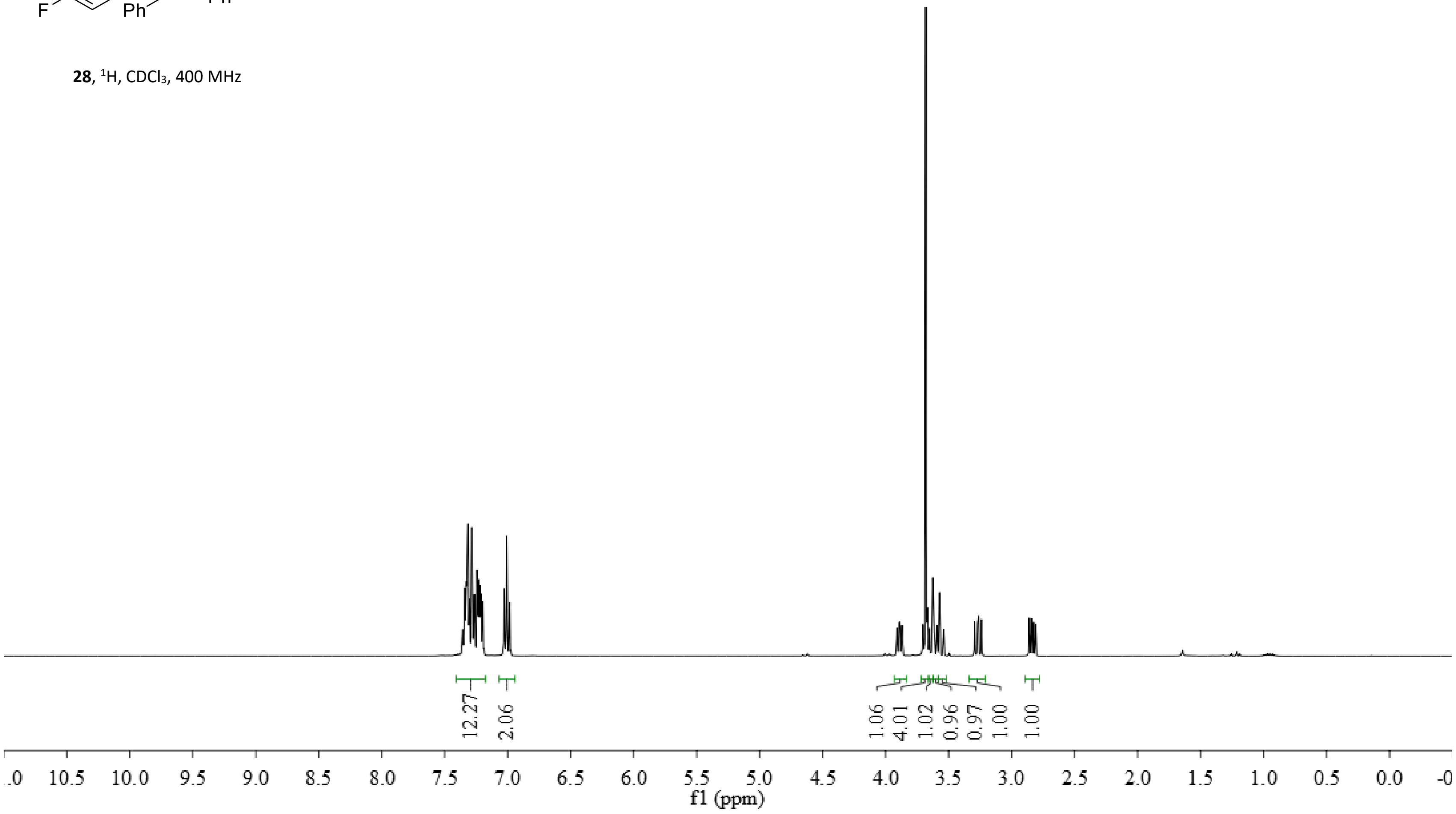

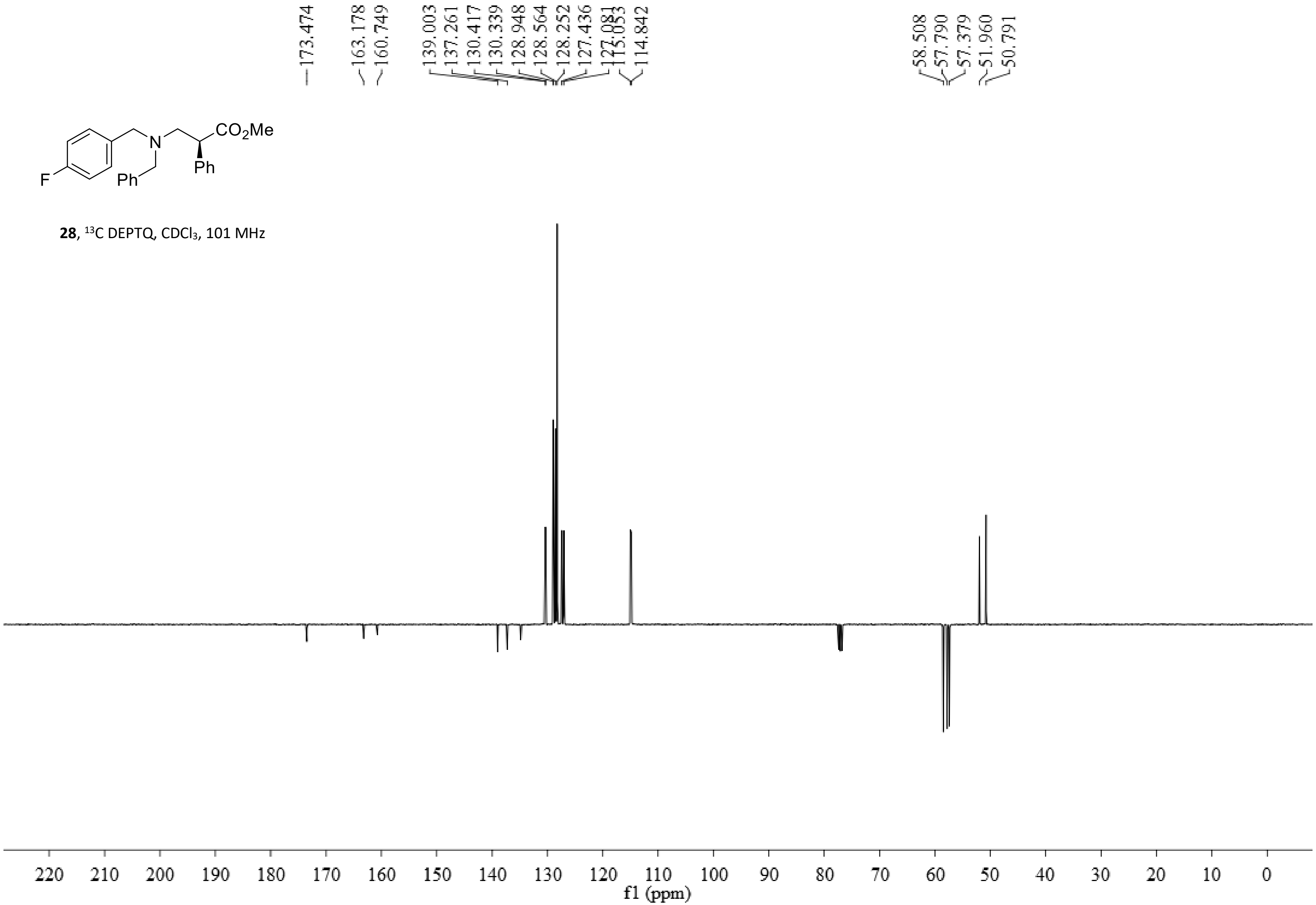

28, ^{13}C DEPTQ, $CDCl_3$, 101 MHz

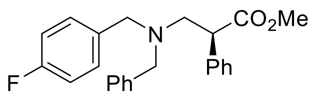

28, ^{19}F, $CDCl_3$, 377 MHz

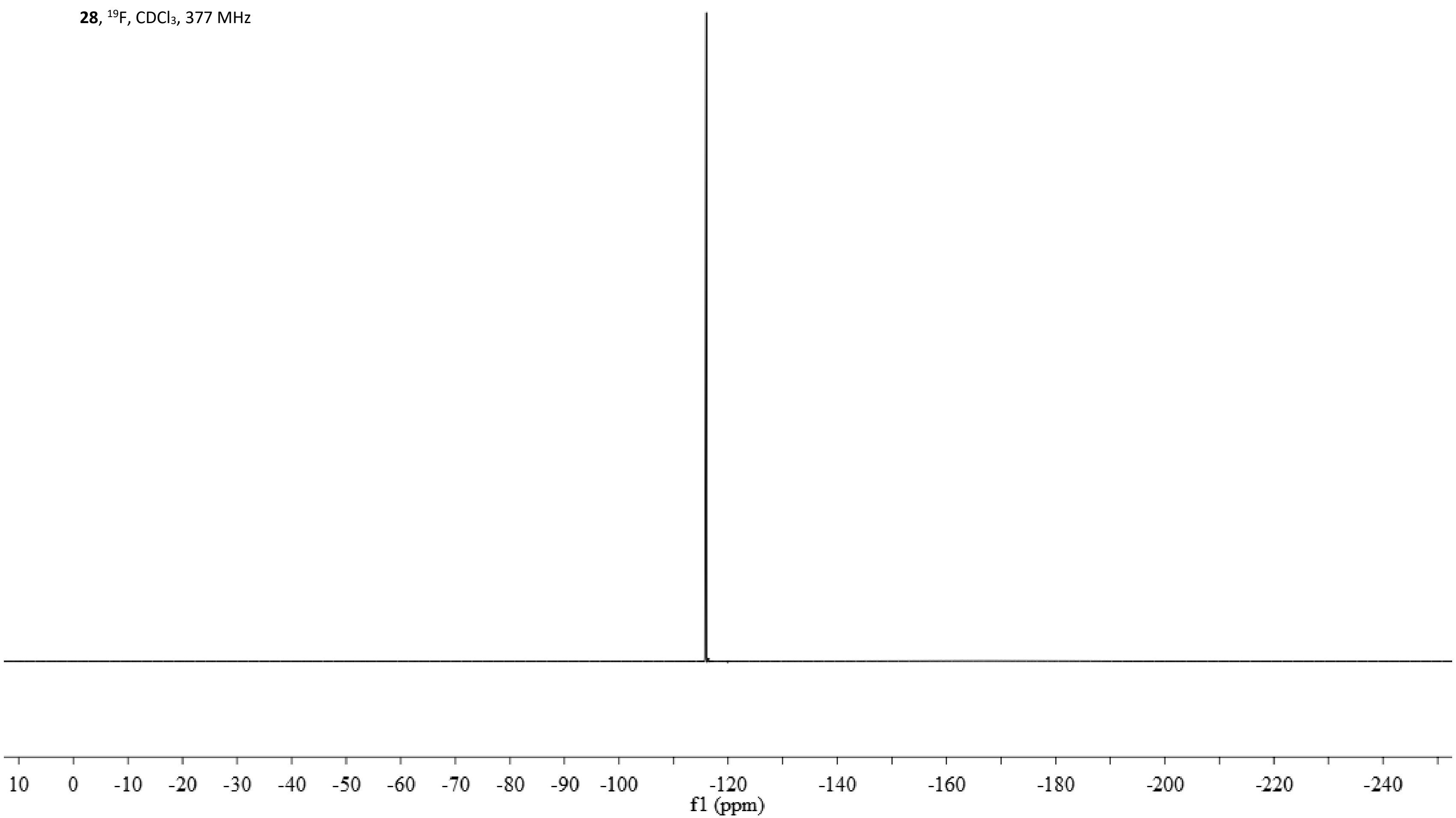

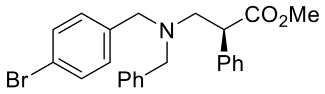

29, ^1H, $CDCl_3$, 500 MHz

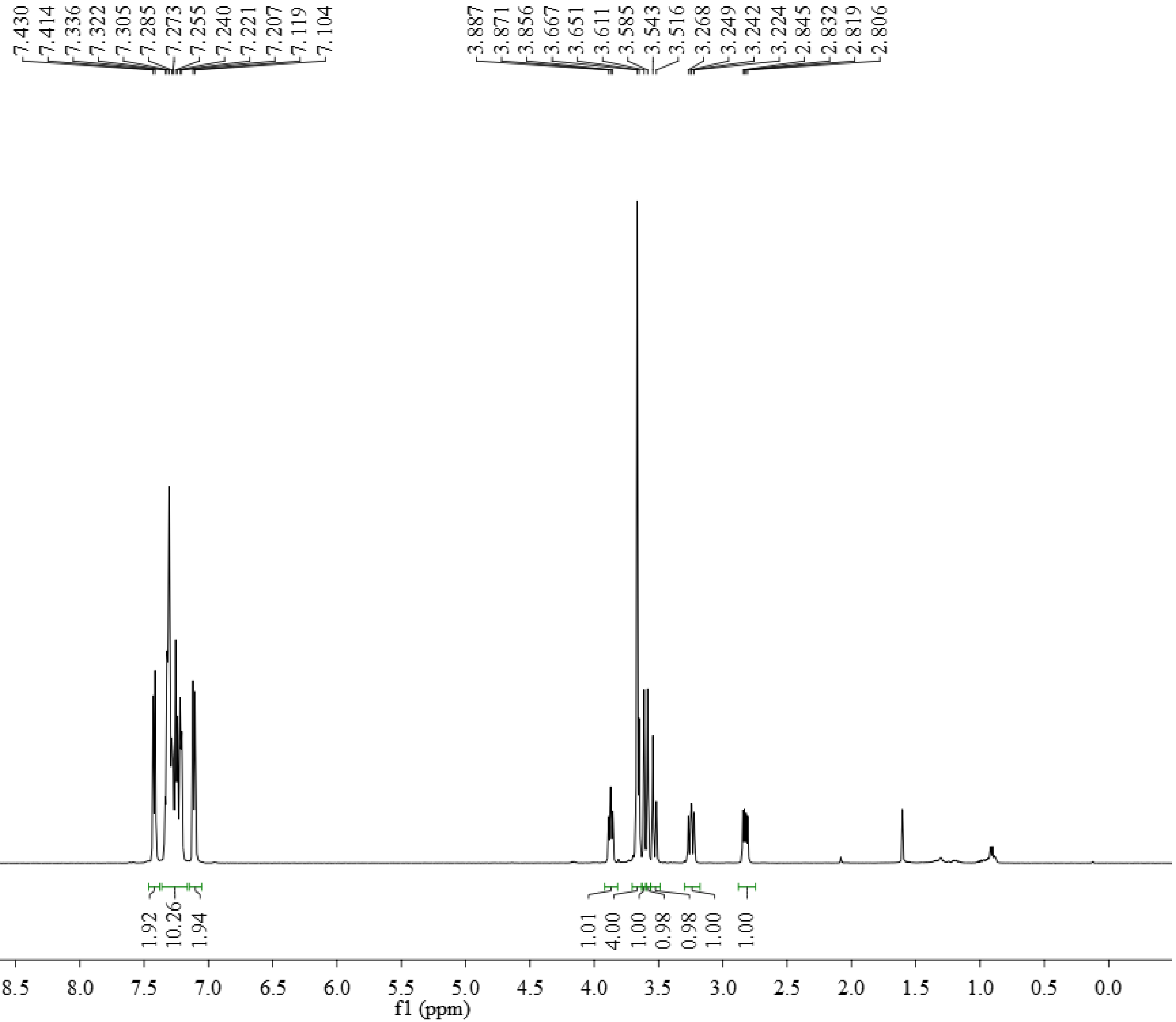

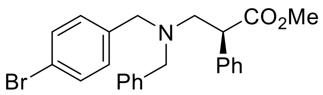

29, ^{13}C DEPTQ, $CDCl_3$, 126 MHz

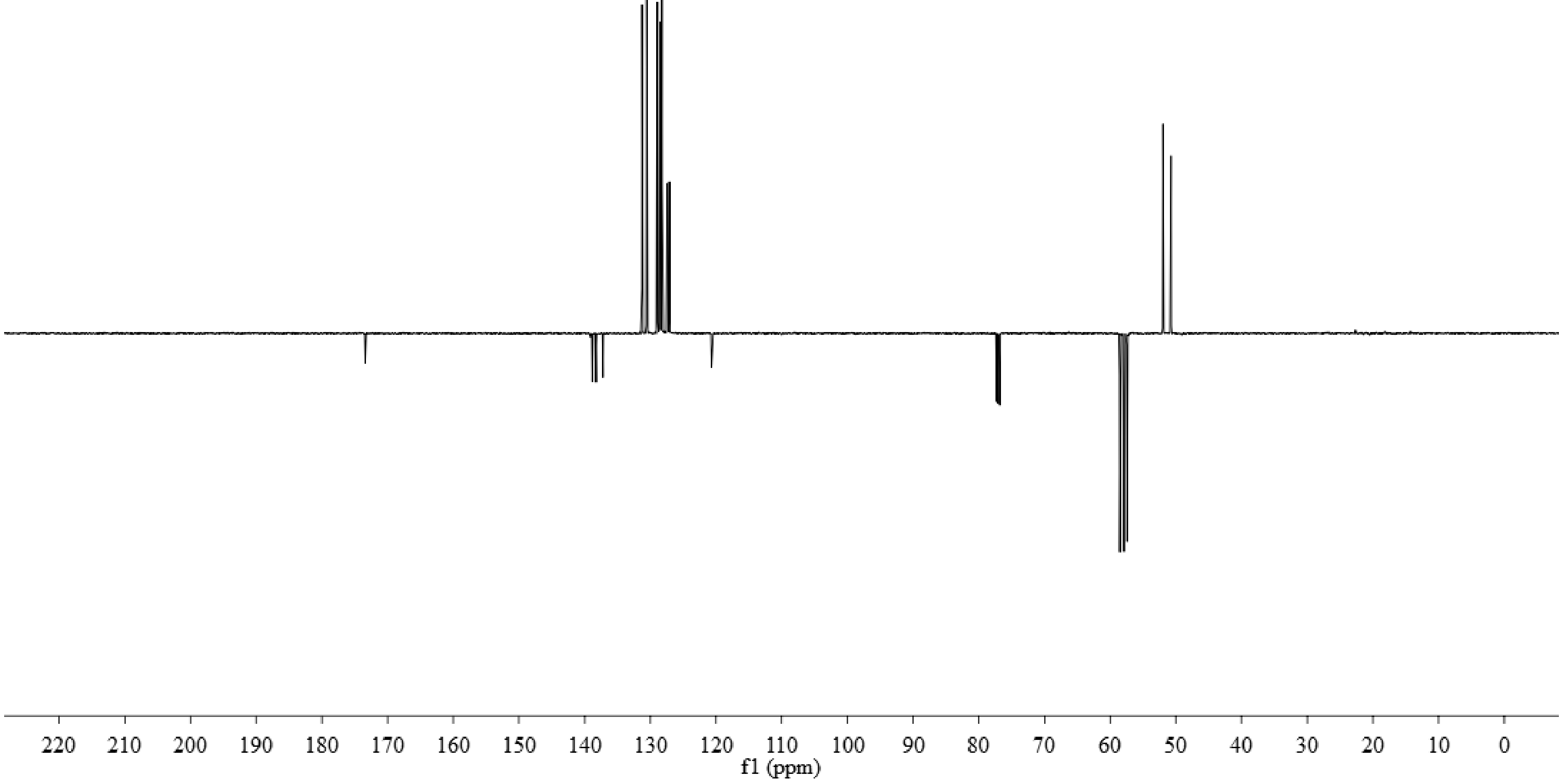

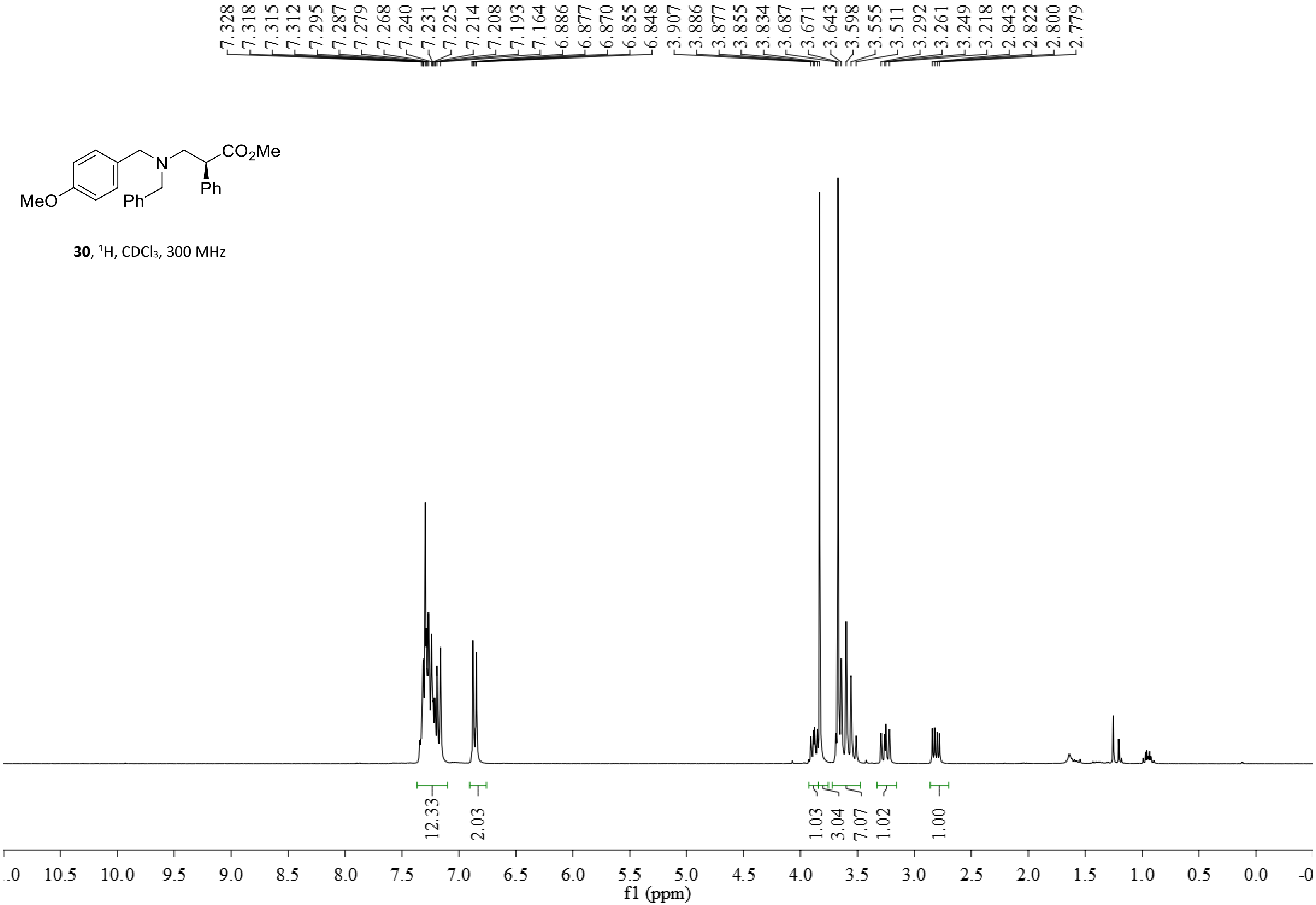

30, ^{1}H, $CDCl_3$, 300 MHz

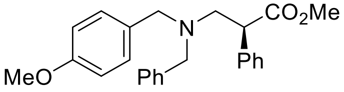

30, ^{13}C DEPTQ, $CDCl_3$, 101 MHz

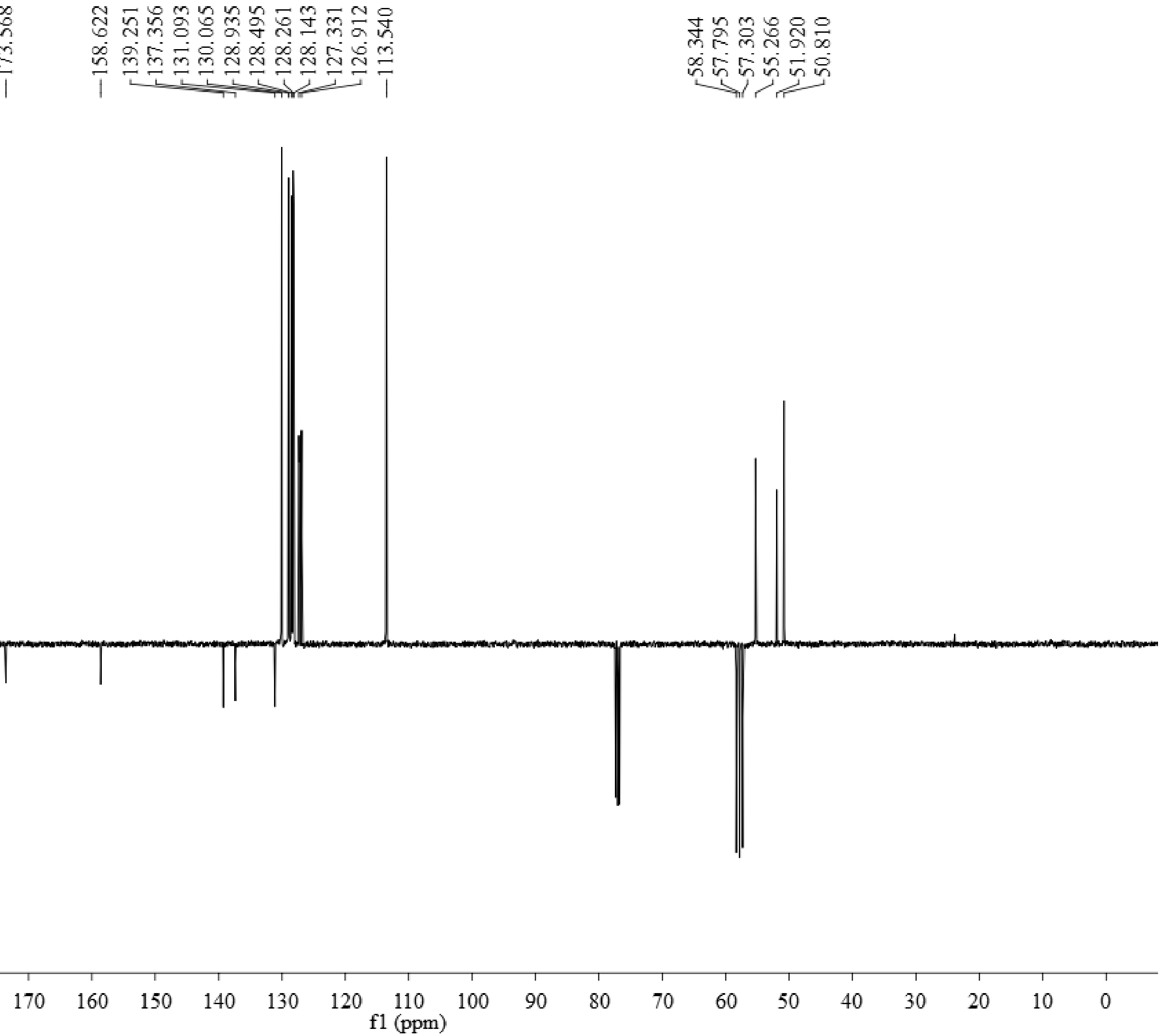

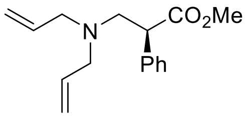

31, ^{1}H, $CDCl_3$, 300 MHz

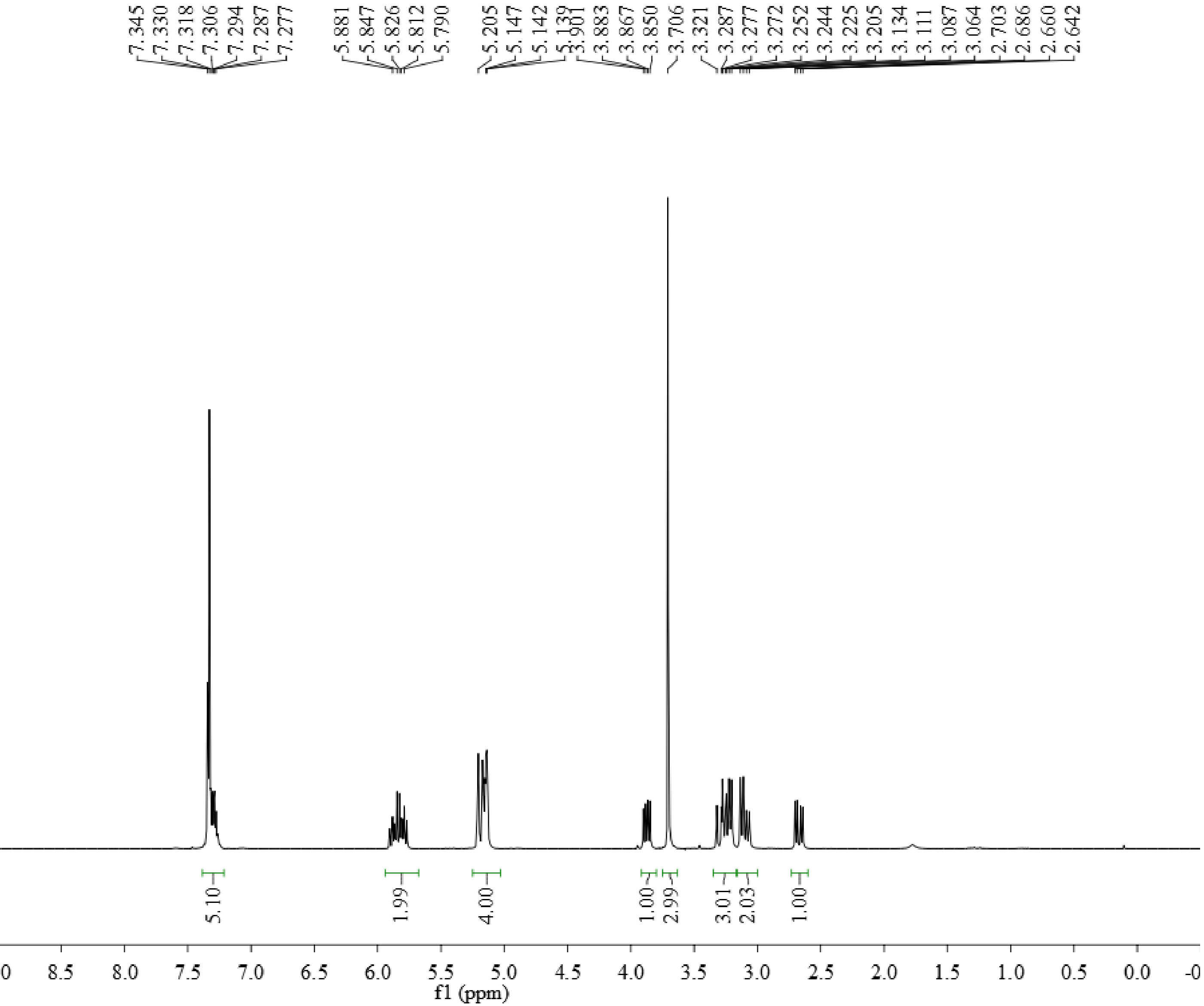

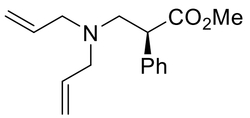

31, ^{13}C DEPTQ, $CDCl_3$, 126 MHz

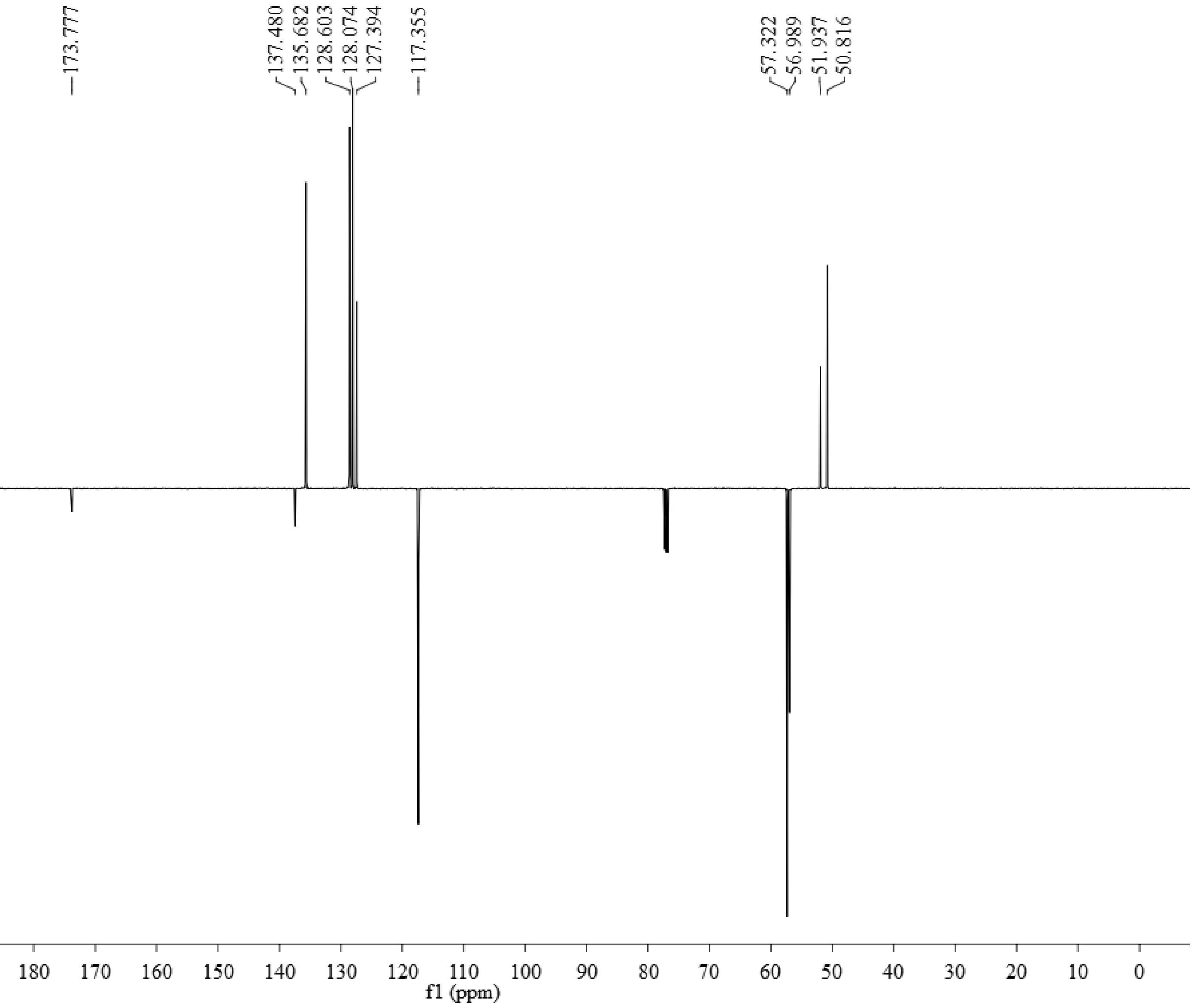

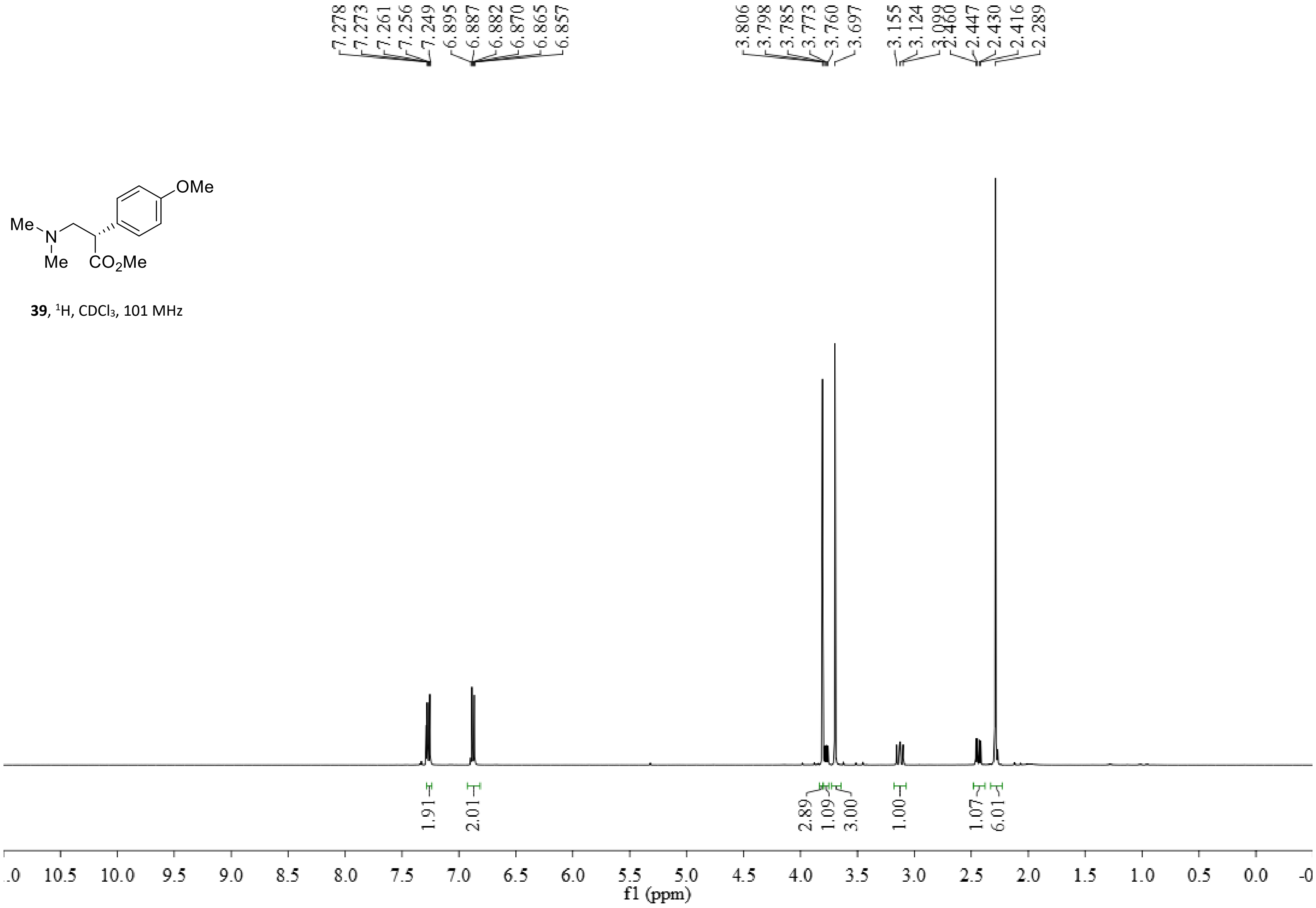

39, ^{1}H, $CDCl_3$, 101 MHz

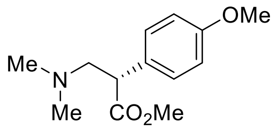

39, ^{13}C DEPTQ, $CDCl_3$, 101 MHz

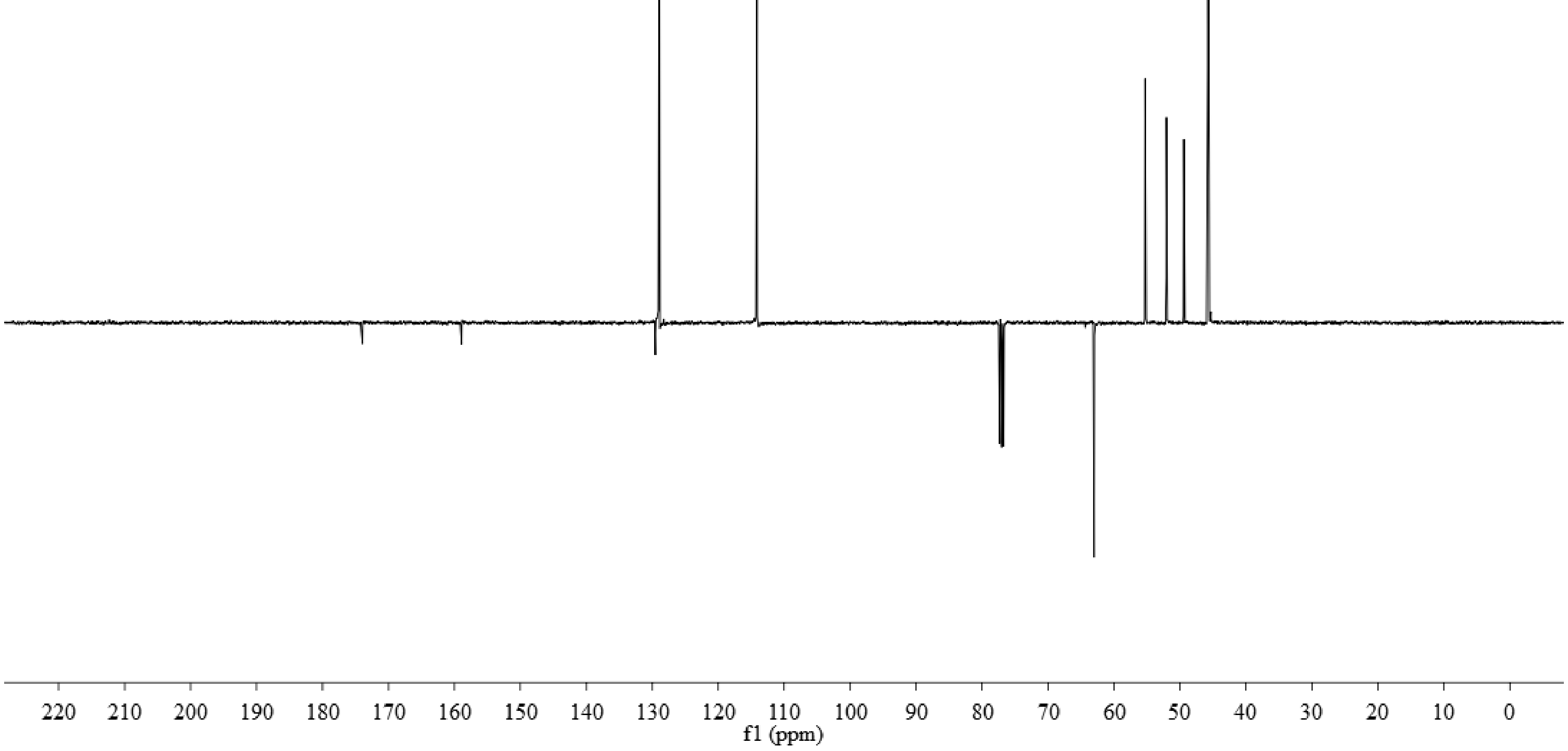

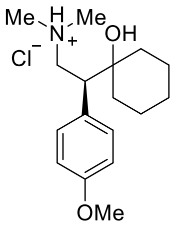

4, ^{1}H, $CDCl_3$, 400 MHz

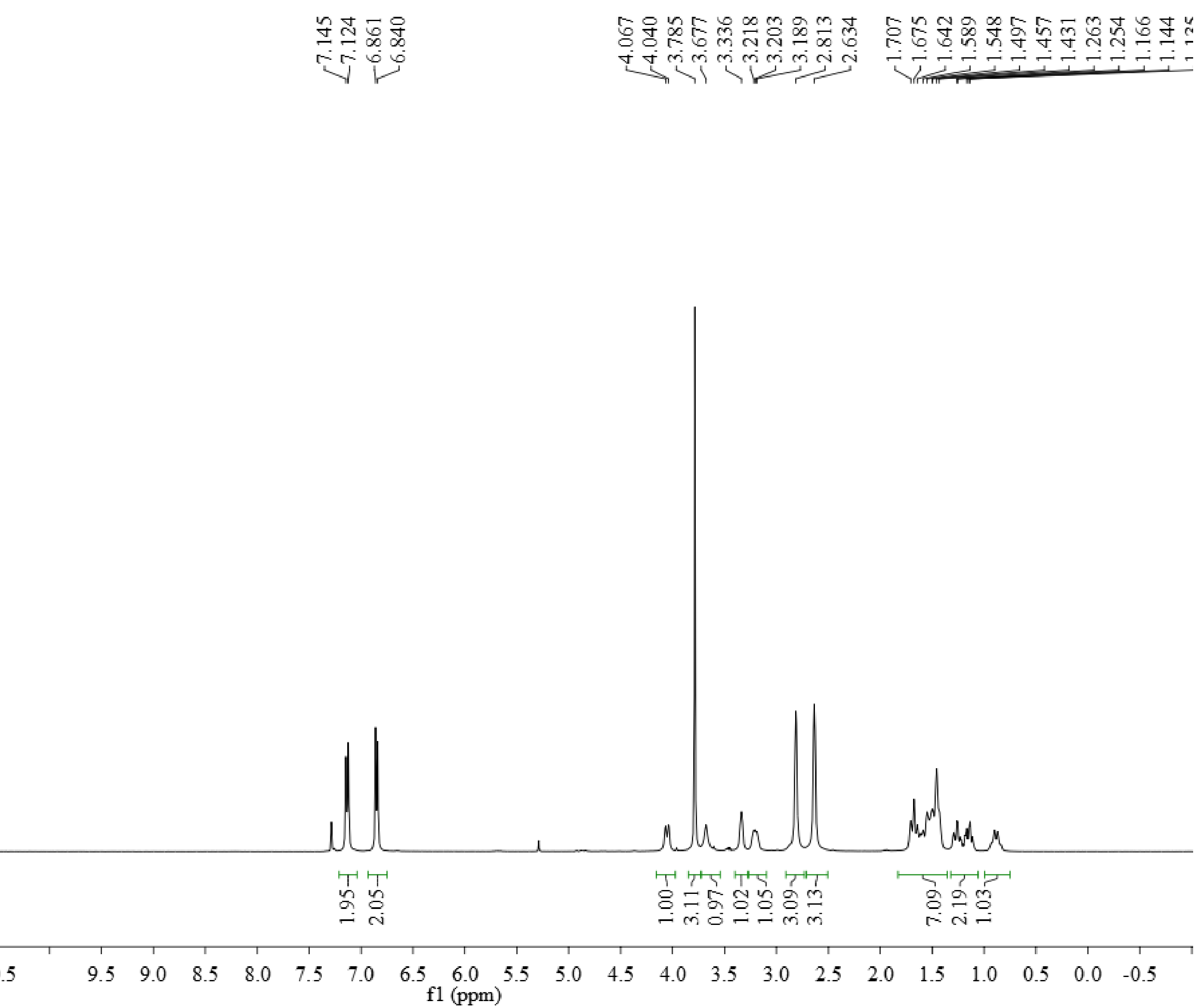

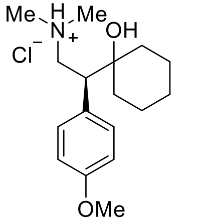

4, ^{13}C DEPTQ, $CDCl_3$, 101 MHz

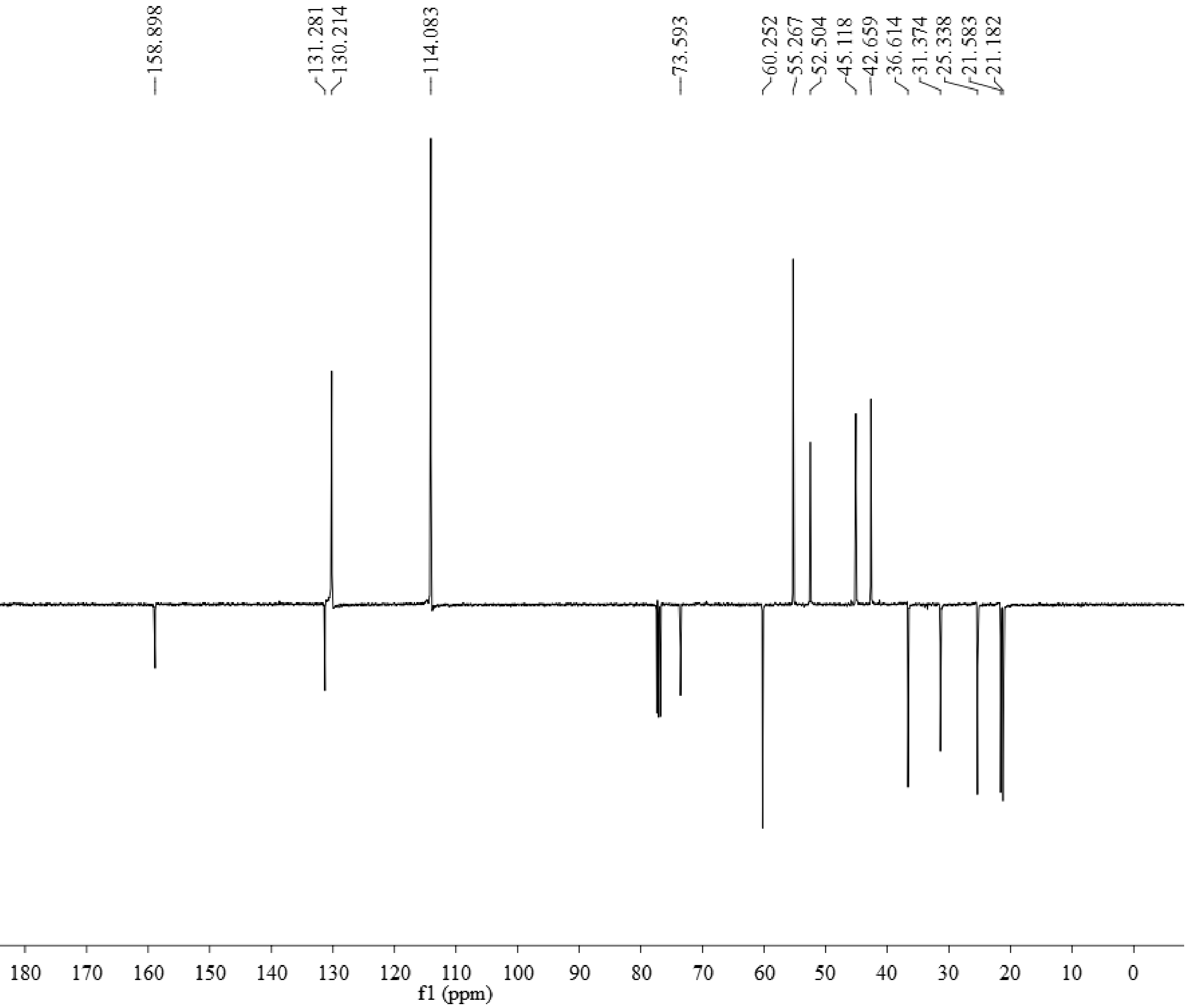

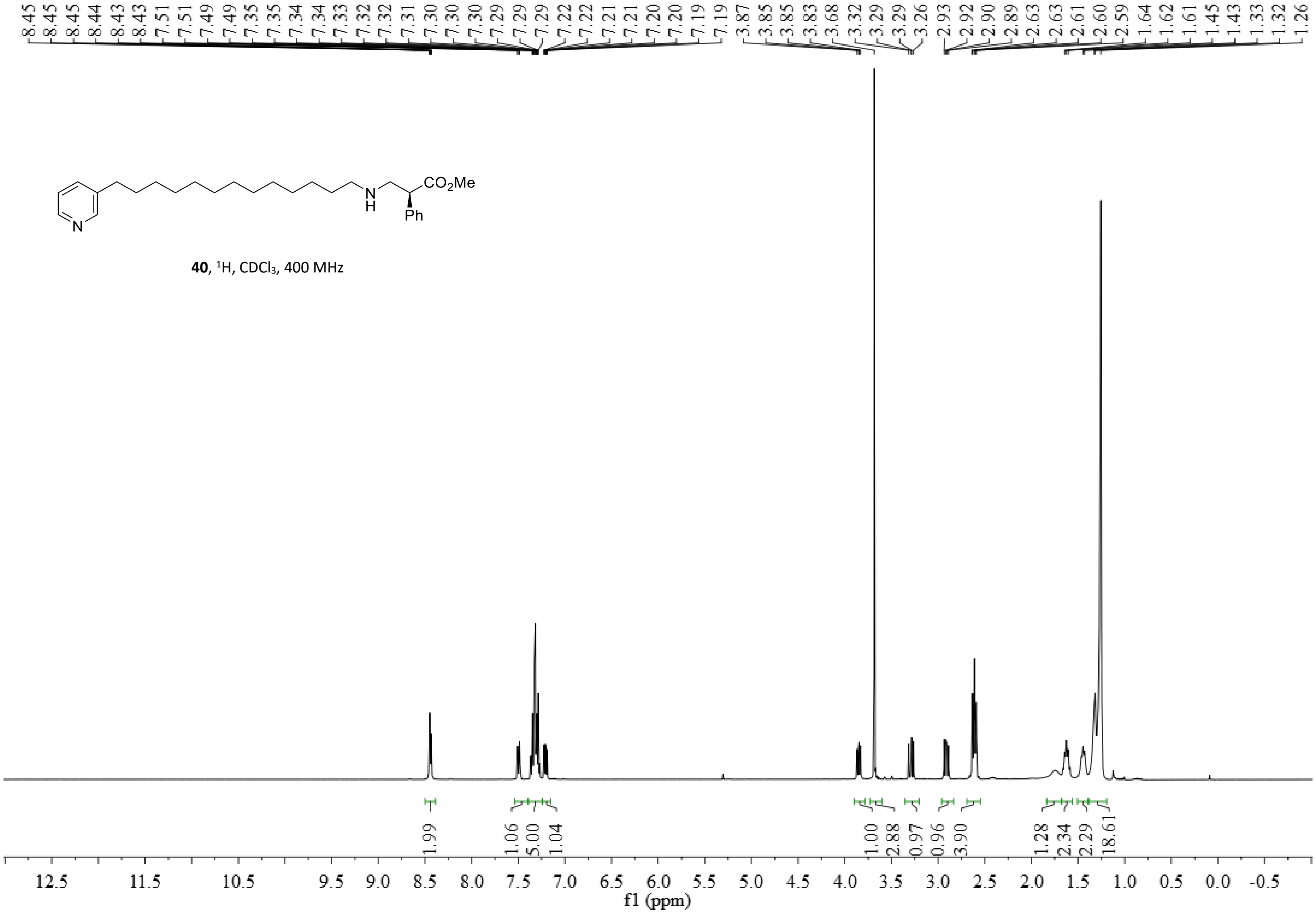

40, ^{1}H, $CDCl_3$, 400 MHz

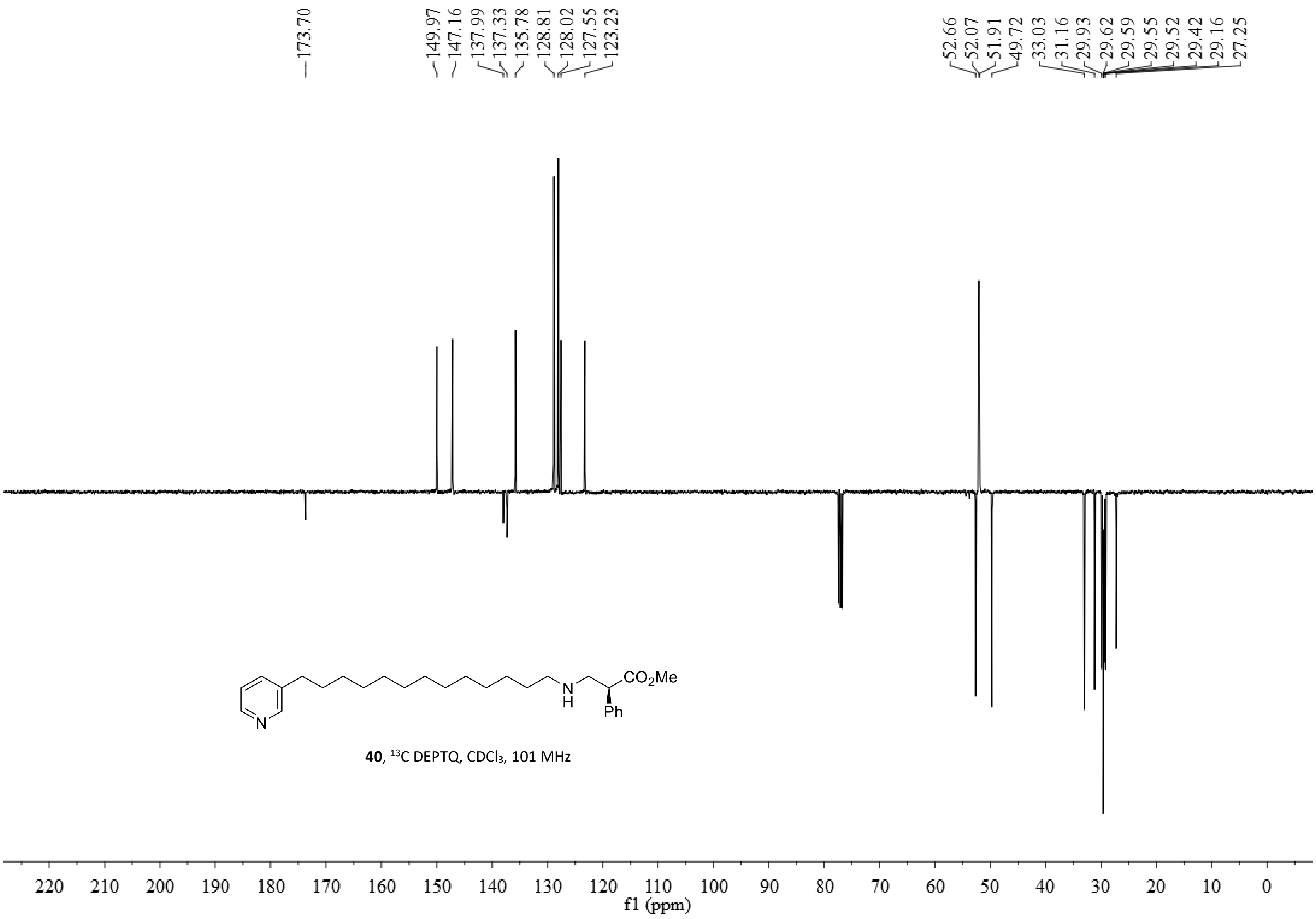

40, ^{13}C DEPTQ, $CDCl_3$, 101 MHz

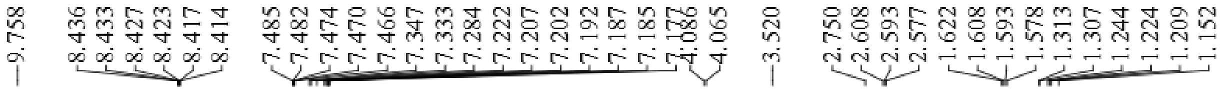

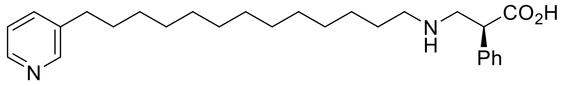

1, ^{1}H, $CDCl_3$, 500 MHz

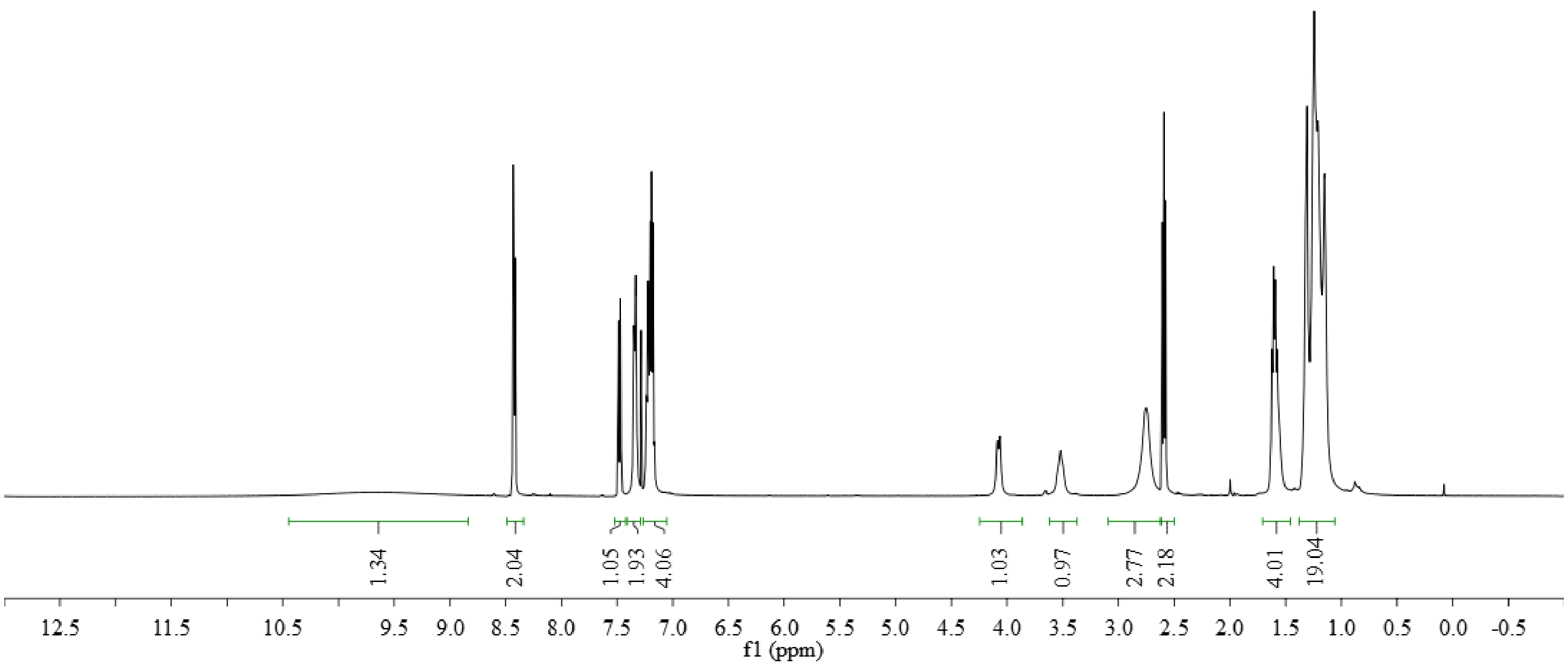

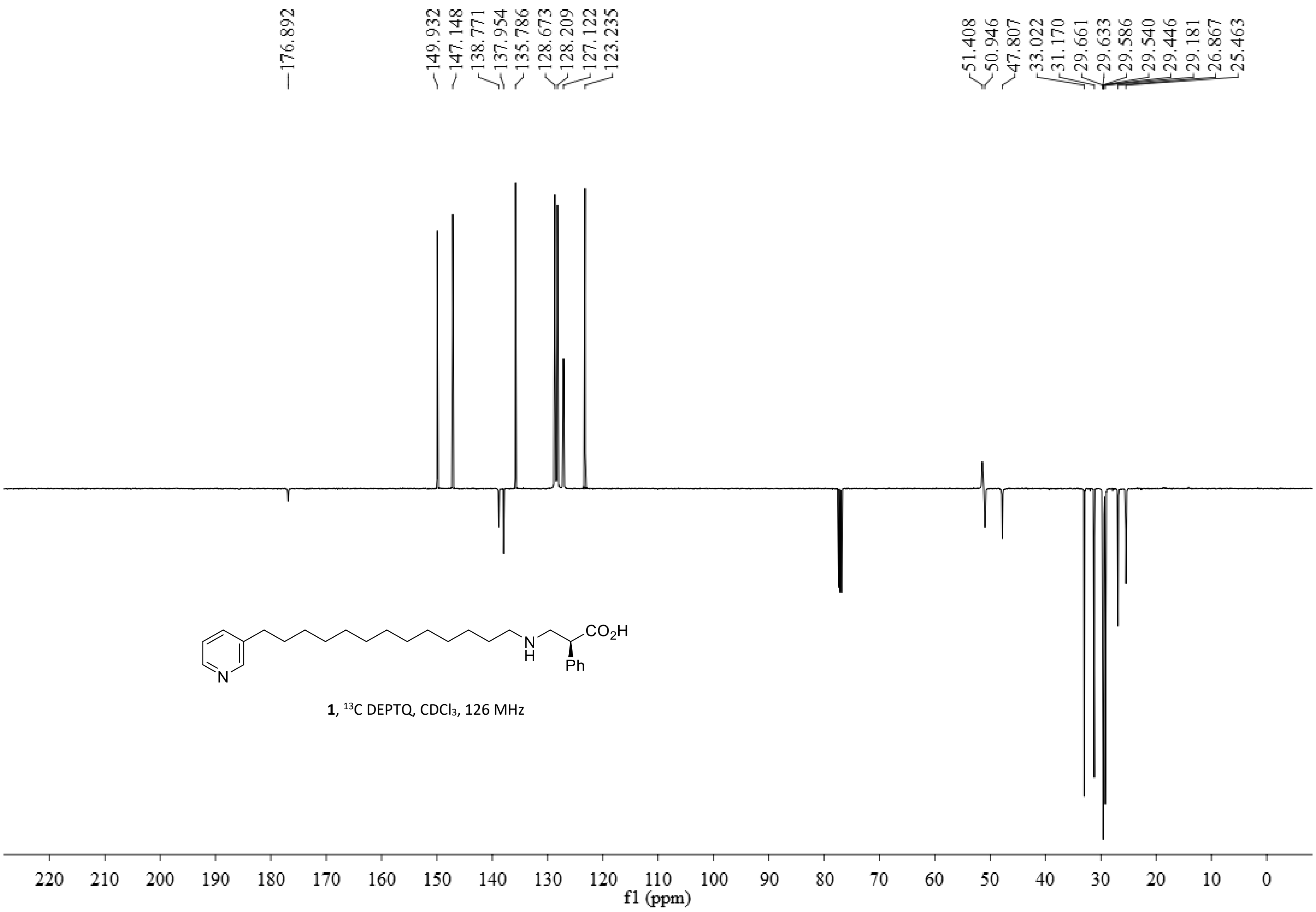

1, ^{13}C DEPTQ, $CDCl_3$, 126 MHz

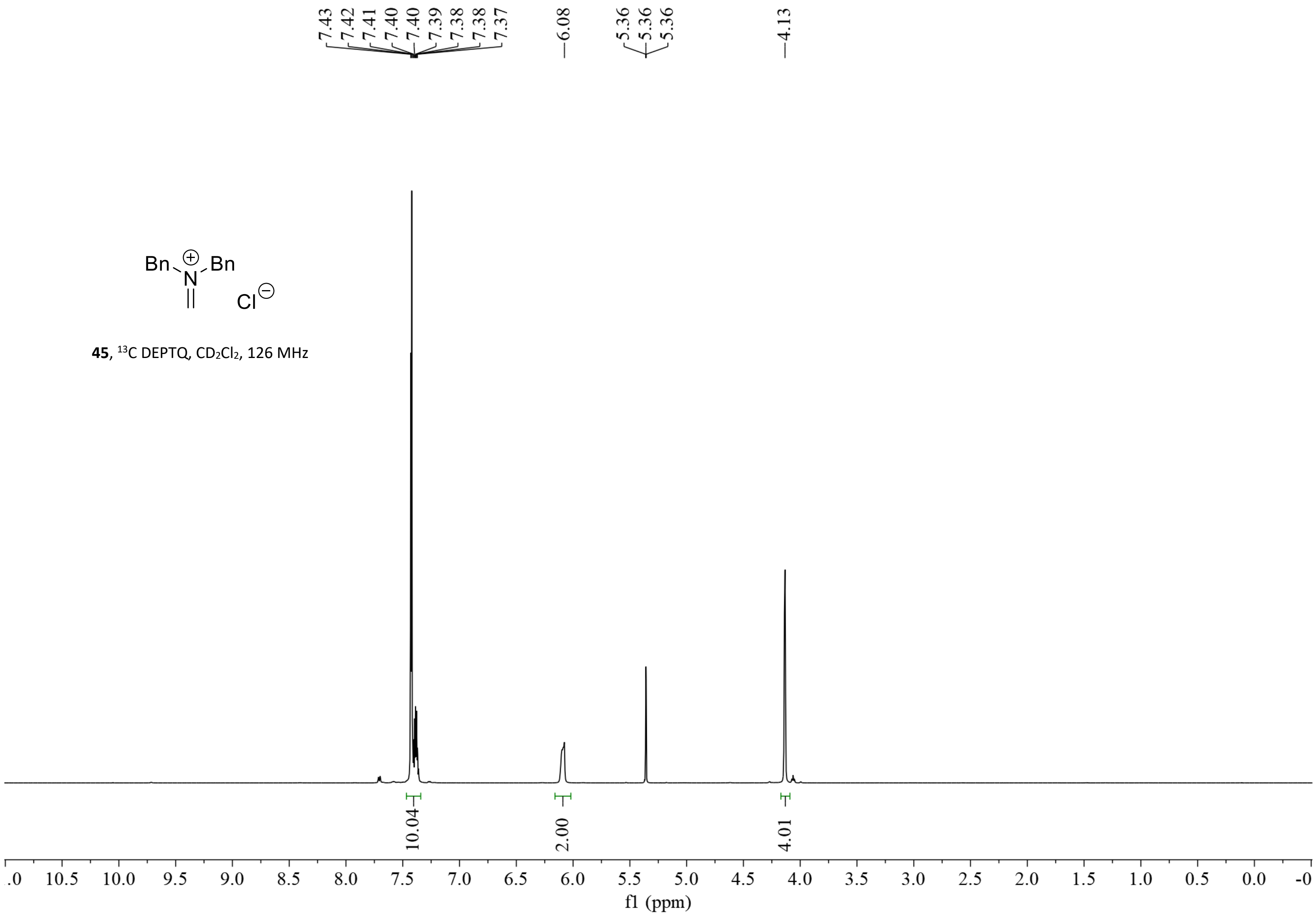

45, ^{13}C DEPTQ, CD_2Cl_2, 126 MHz

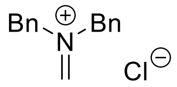

45, ^{13}C DEPTQ, CD_2Cl_2, 126 MHz

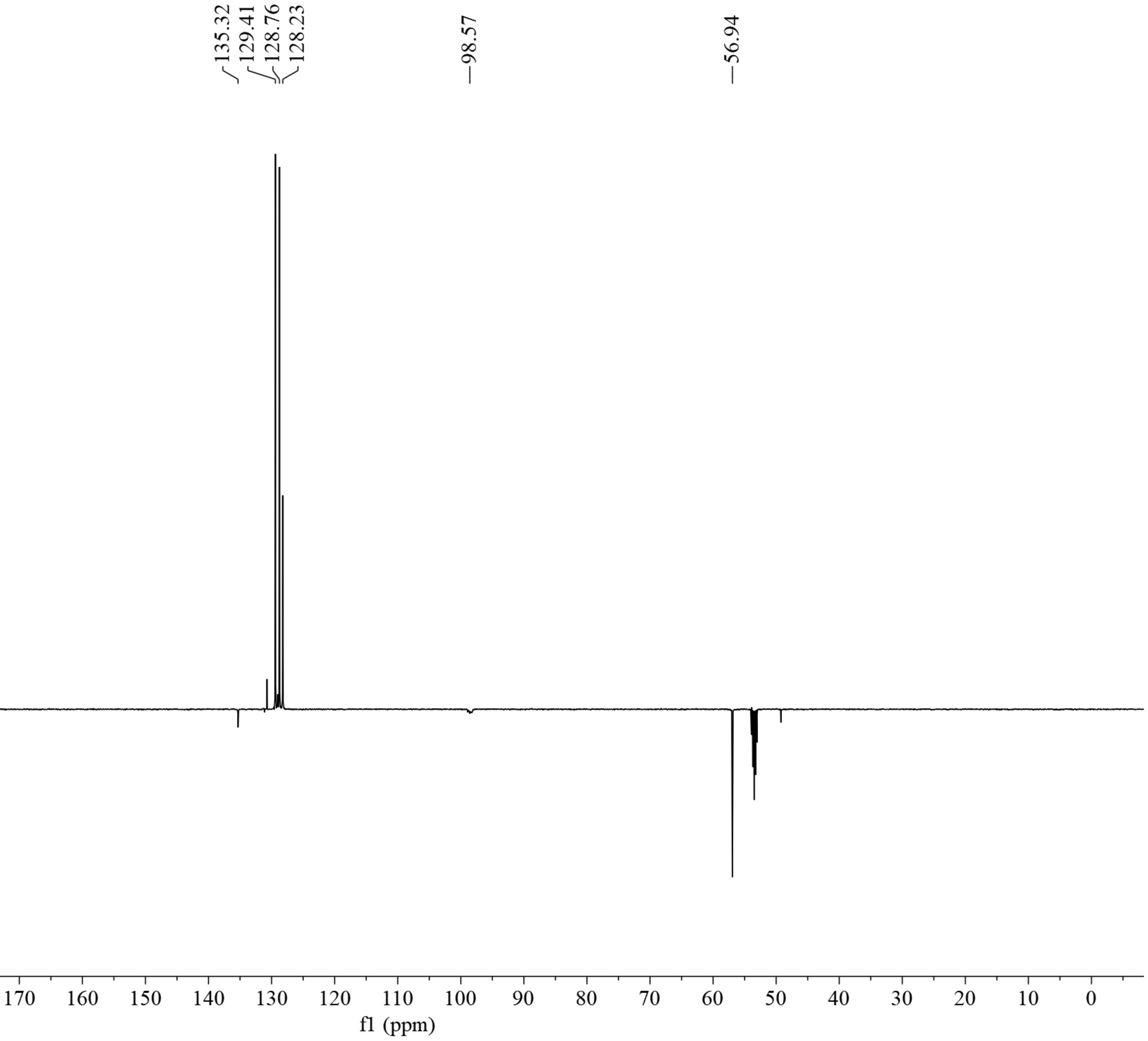

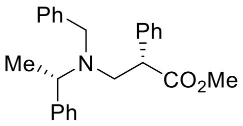

51, ^{1}H, $CDCl_3$, 400 MHz

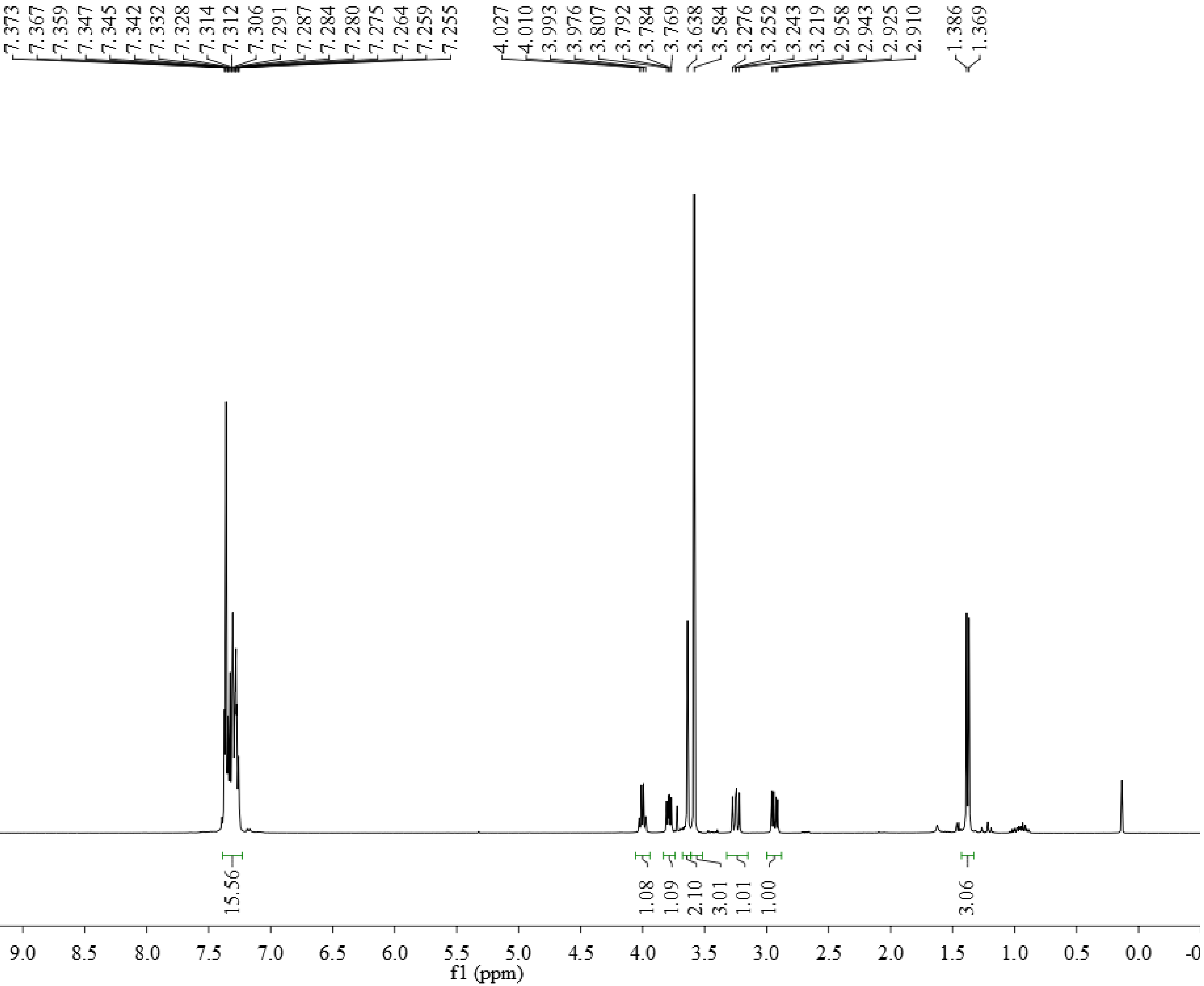

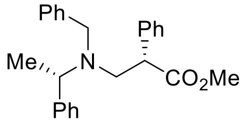

51, ^{13}C DEPTQ, $CDCl_3$, 101 MHz

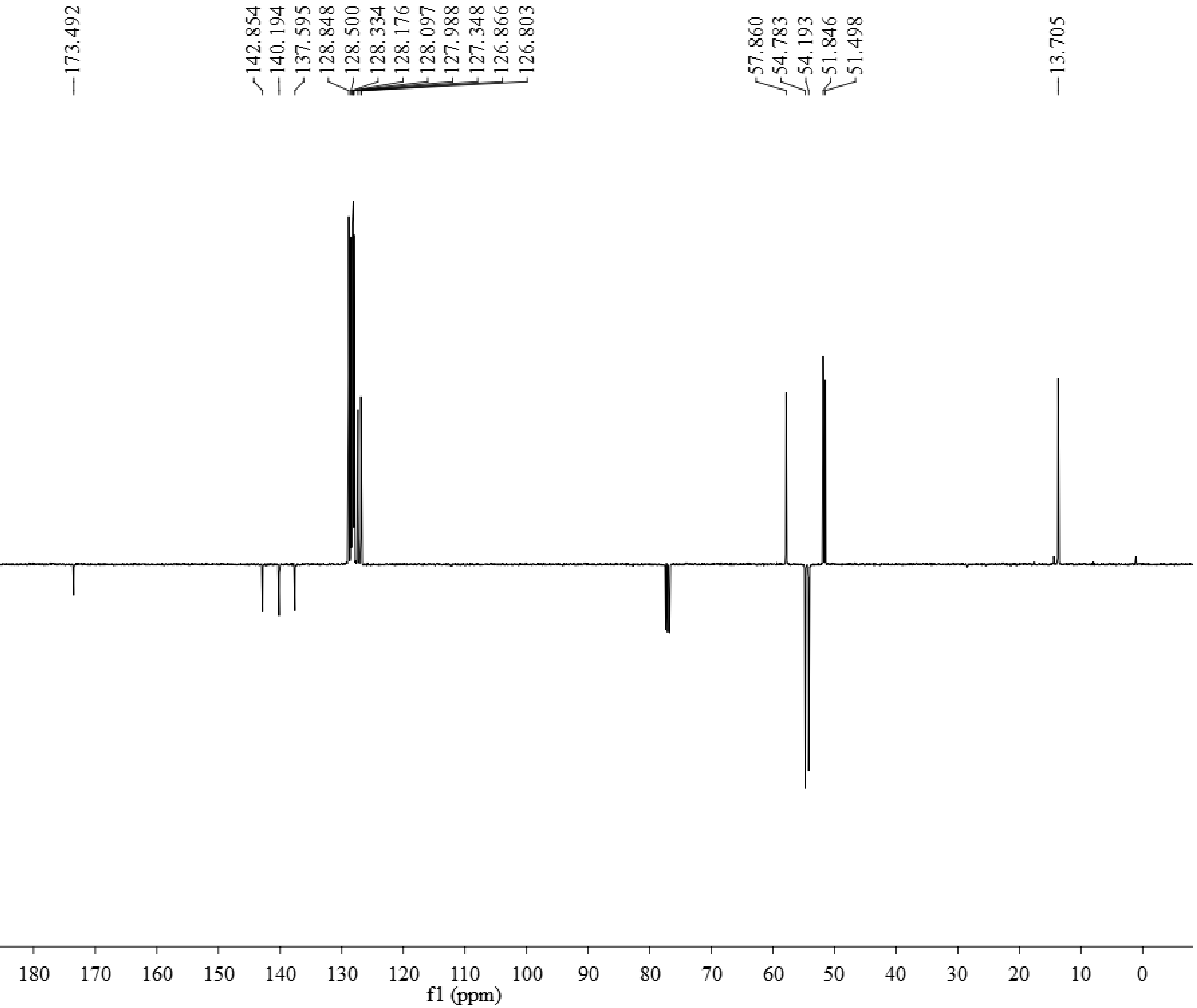

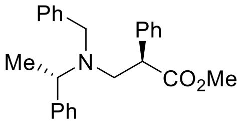

52, ^{1}H, $CDCl_3$, 300 MHz

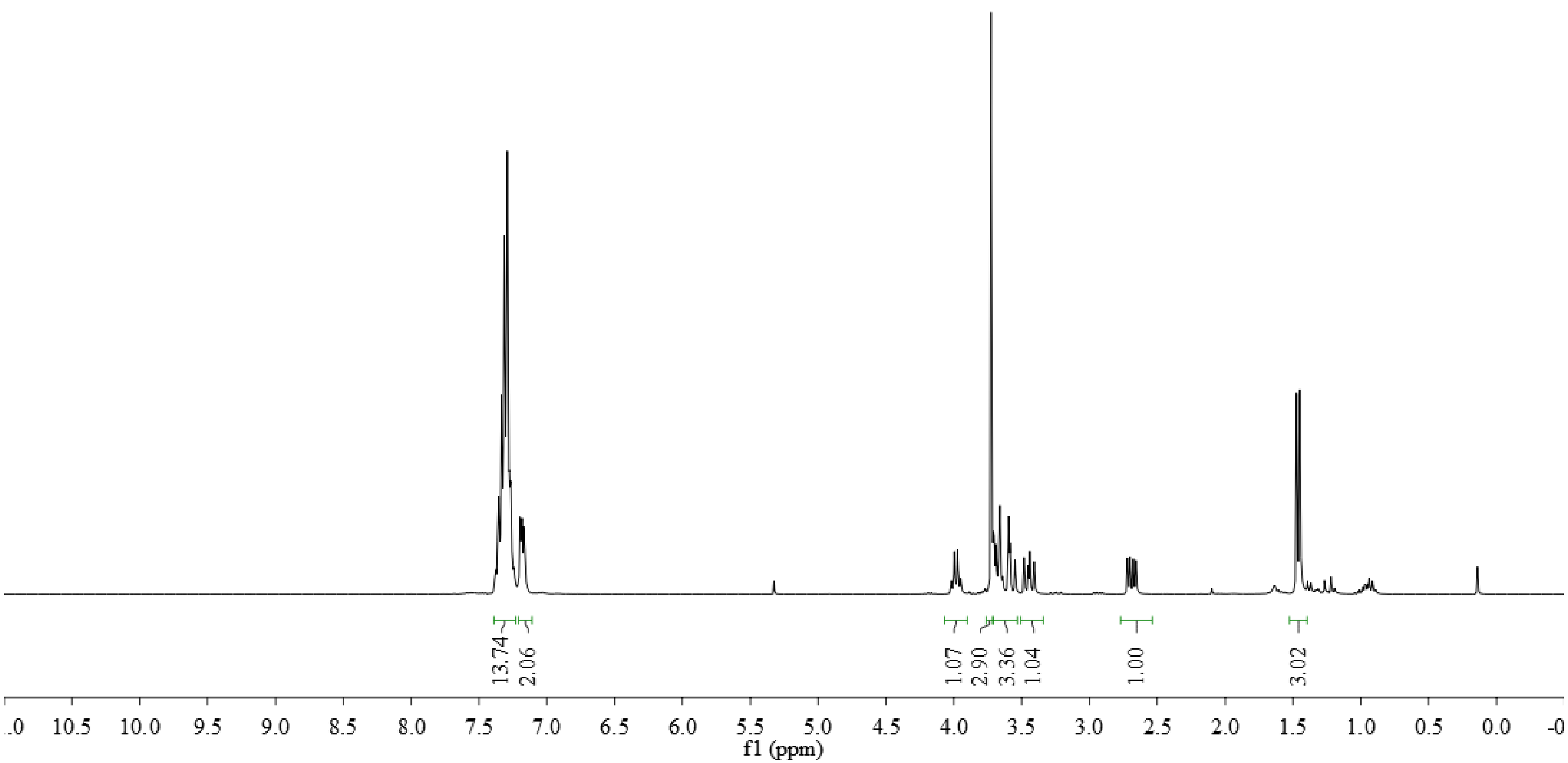

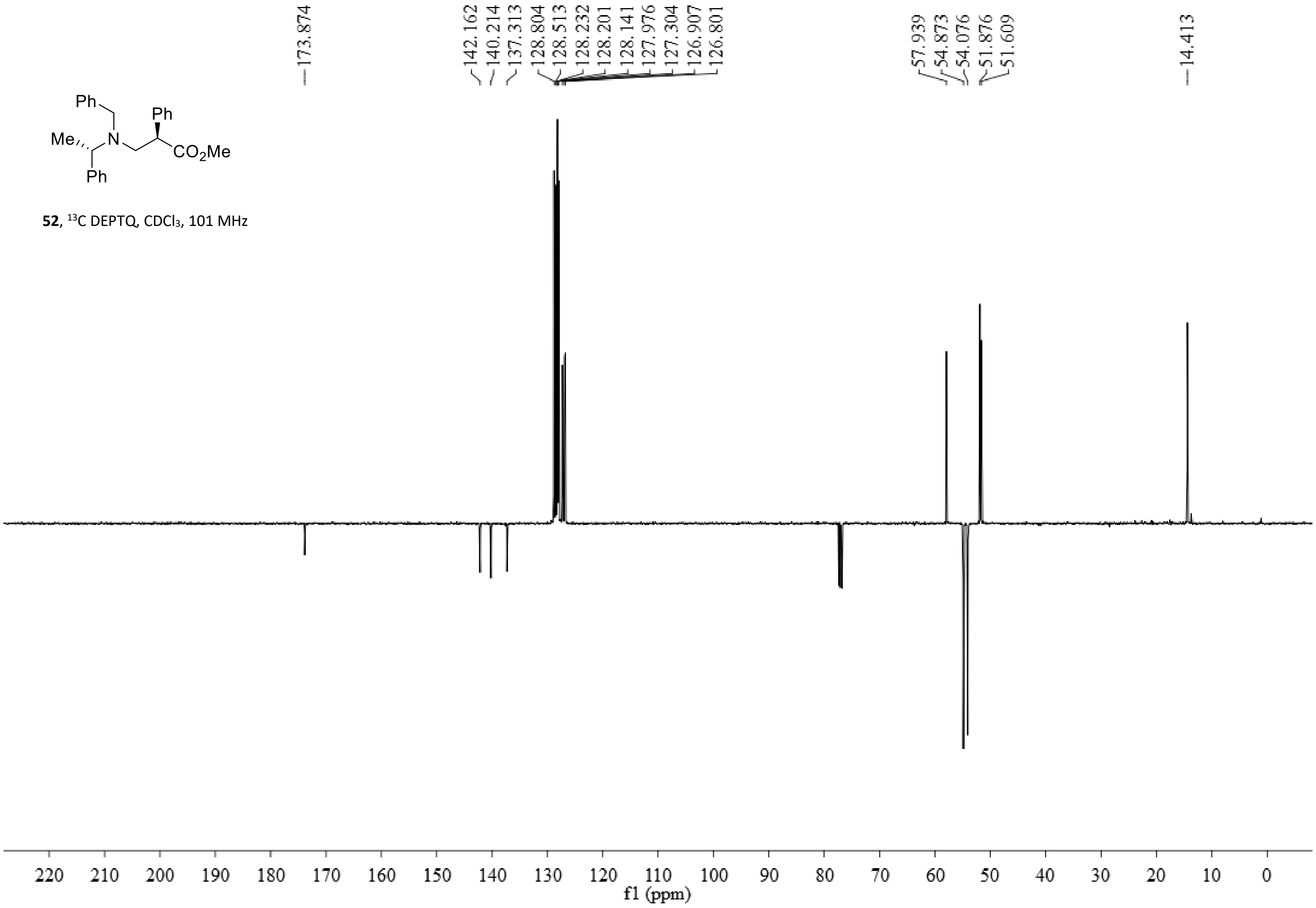

52, ^{13}C DEPTQ, $CDCl_3$, 101 MHz

Appendix II: HPLC Traces

HPLC data for 9

Chiralcel OD-H (98.5:1.5 hexane:IPA, flow rate 1 mLmin^{-1}, 211 nm, 30 °C) t_R (*S*): 6.7 min, t_R (*R*): 8.2 min, 96:4 er

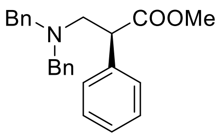

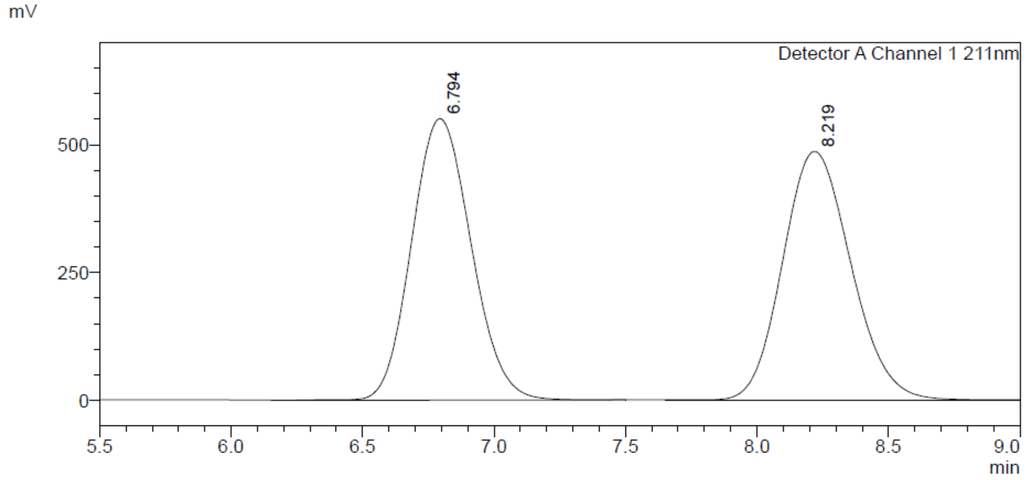

<Peak Table>

Detector A Channel 1 211nm

Peak#	Ret. Time	Area%
1	6.794	49.796
2	8.219	50.204
Total		100.000

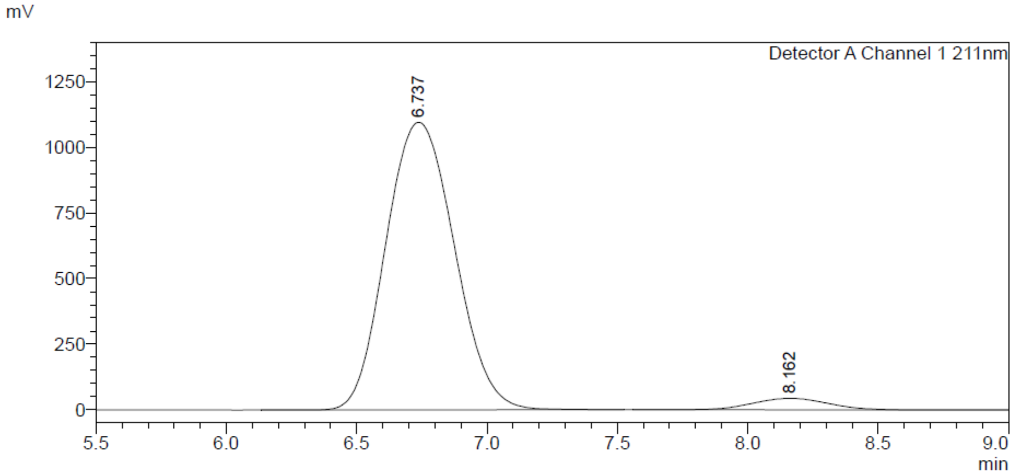

<Peak Table>

Detector A Channel 1 211nm

Peak#	Ret. Time	Area%
1	6.737	95.808
2	8.162	4.192
Total		100.000

HPLC data for 10

Chiralcel OD-H (98.5:1.5 hexane:IPA, flow rate 1 mLmin^{-1}, 211 nm, 30 °C) t_R (*S*): 9.3 min, t_R (*R*): 11.5 min, 95:5 er

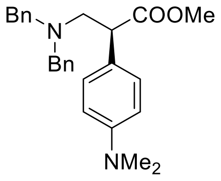

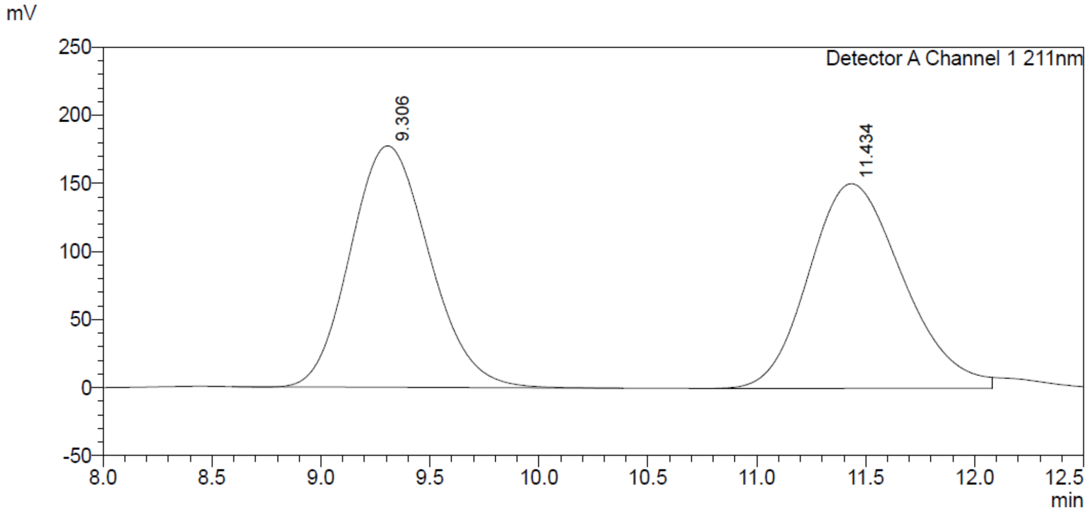

Peak#	Ret. Time	Area%
1	9.306	49.731
2	11.434	50.269
Total		100.000

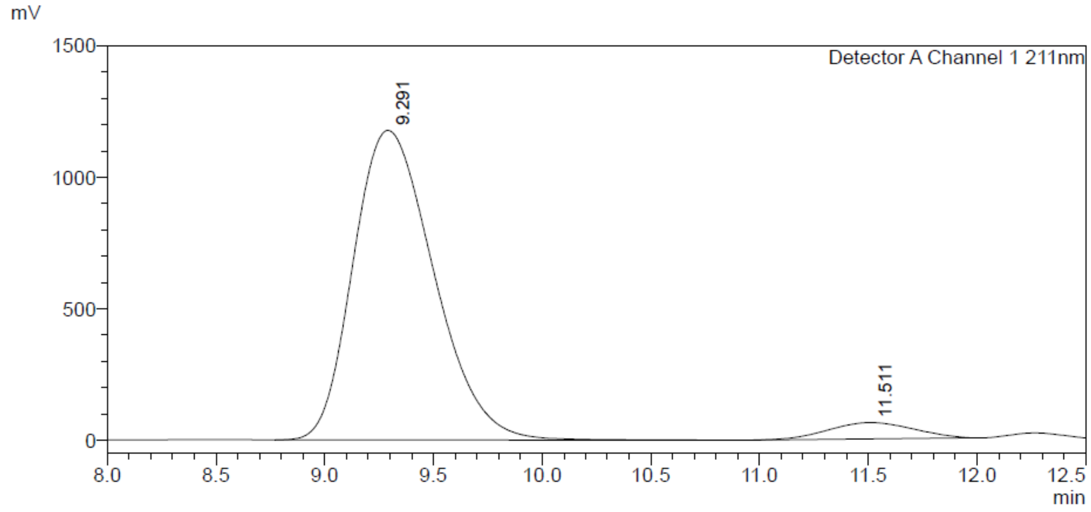

<Peak Table>

Detector A Channel 1 211nm

Peak#	Ret. Time	Area%
1	9.291	94.720
2	11.511	5.280
Total		100.000

HPLC data for 11

Chiralcel OD-H (95:5 hexane:IPA, flow rate 1 mLmin^{-1}, 211 nm, 30 °C) t_R (*S*): 6.1 min, t_R (*R*): 7.2 min, 94:6 er

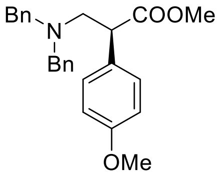

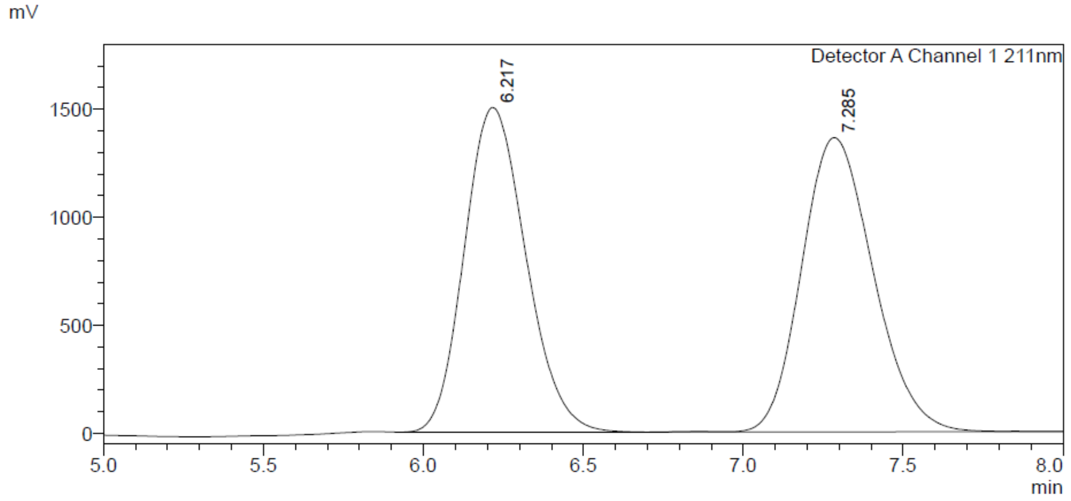

Peak#	Ret. Time	Area%
1	6.217	48.605
2	7.285	51.395
Total		100.000

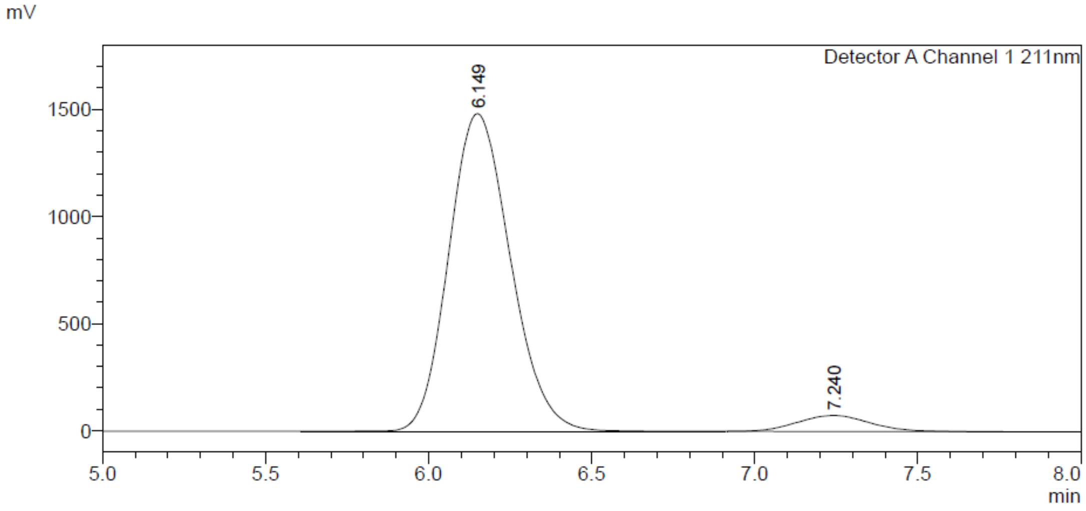

<Peak Table>

Detector A Channel 1 211nm

Peak#	Ret. Time	Area%
1	6.149	94.395
2	7.240	5.605
Total		100.000

HPLC data for 12

Chiralcel OD-H (97:3 hexane:IPA, flow rate 1 mLmin^{-1}, 211 nm, 30 °C) t_R (*S*): 5.1 min, t_R (*R*): 5.7 min, 95:5 er

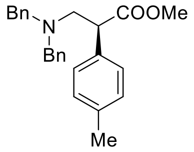

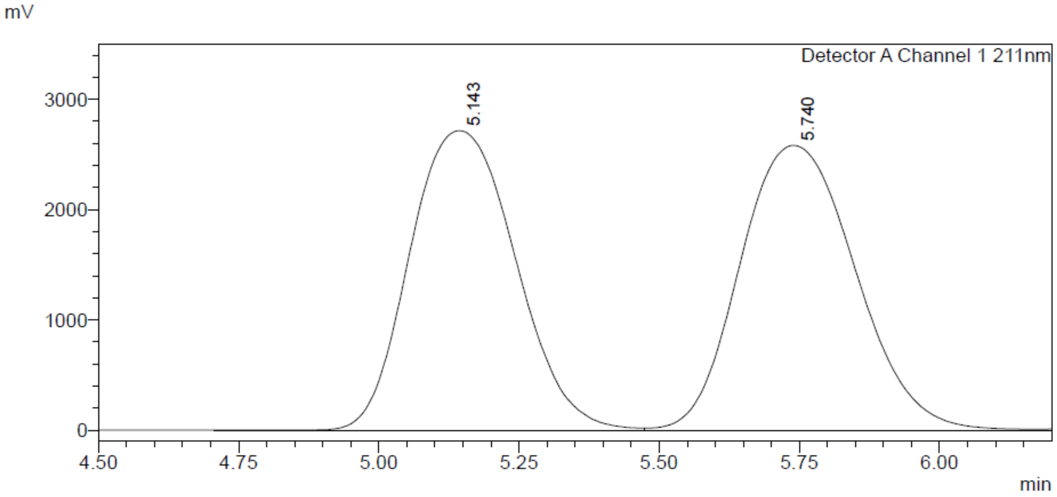

<Peak Table>

Detector A Channel 1 211nm

Peak#	Ret. Time	Area%
1	5.143	48.965
2	5.740	51.035
Total		100.000

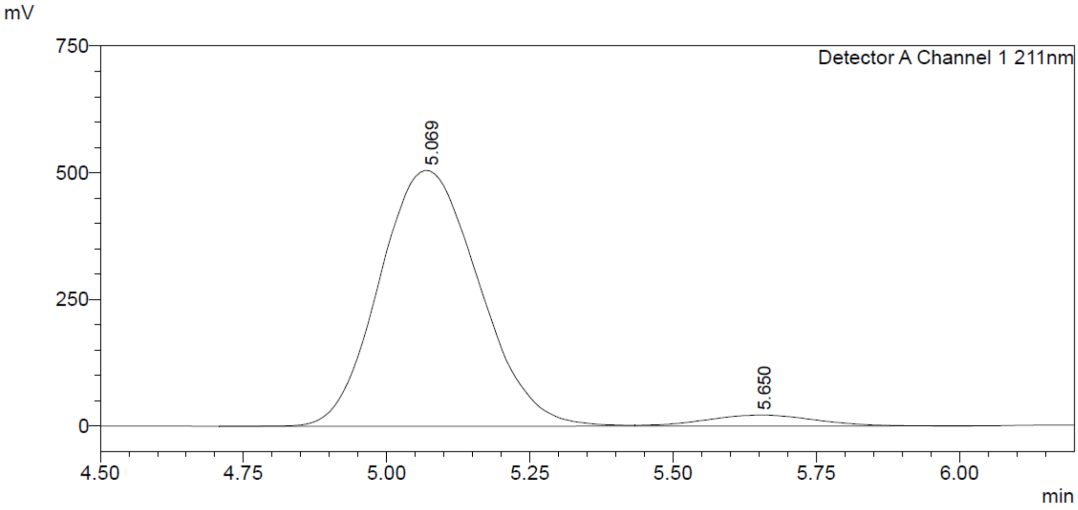

<Peak Table>

Detector A Channel 1 211nm

Peak#	Ret. Time	Area%
1	5.069	95.494
2	5.650	4.506
Total		100.000

HPLC data for 13

Chiralcel OD-H (97:3 hexane:IPA, flow rate 1 mLmin^{-1}, 211 nm, 30 °C) t_R (*S*): 6.2 min, t_R (*R*): 7.6 min, 96:4 er

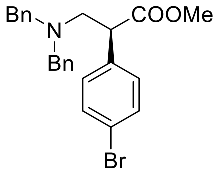

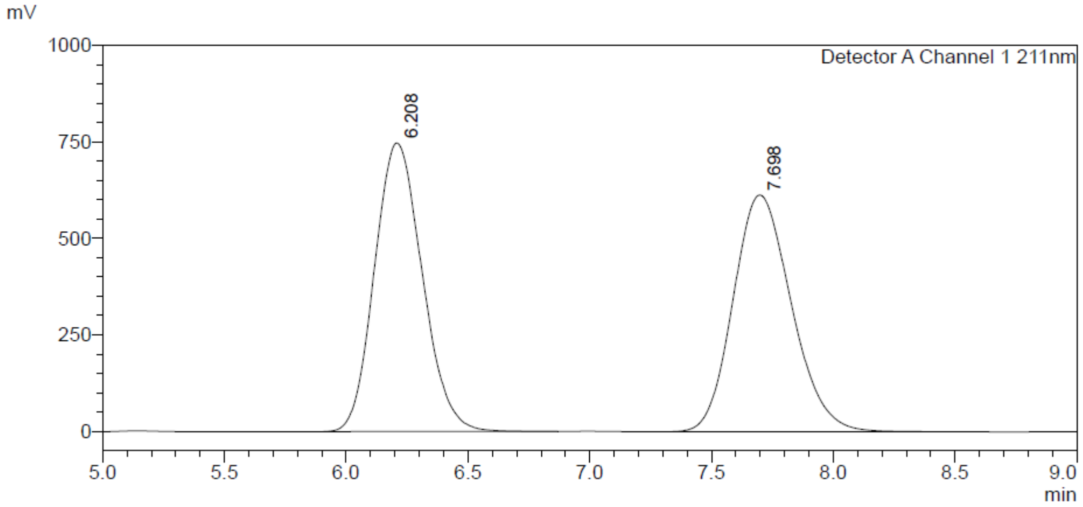

<Peak Table>

Detector A Channel 1 211nm

Peak#	Ret. Time	Area%
1	6.208	50.020
2	7.698	49.980
Total		100.000

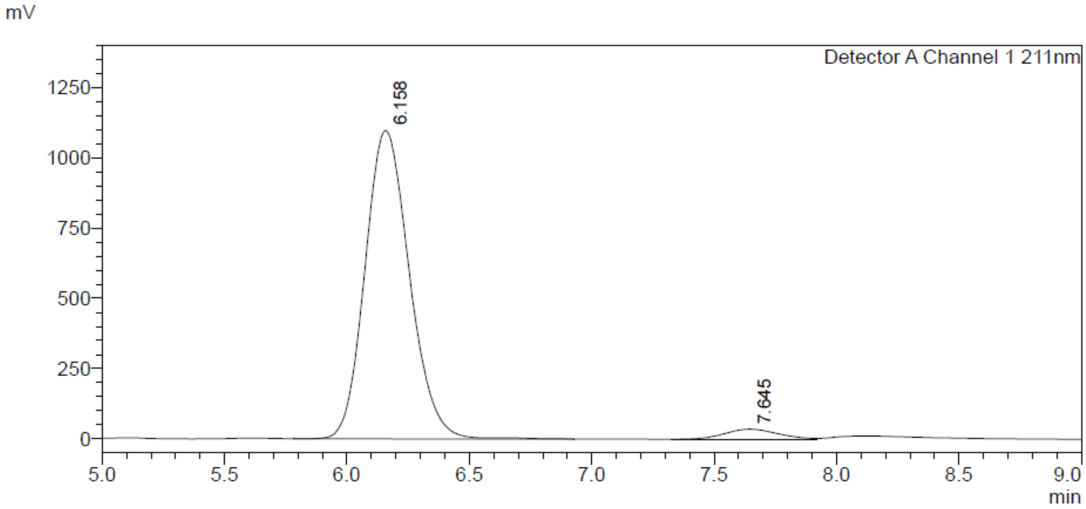

<Peak Table>

Detector A Channel 1 211nm

Peak#	Ret. Time	Area%
1	6.158	96.221
2	7.645	3.779
Total		100.000

HPLC data for 14

Chiralcel OD-H (98.5:1.5 hexane:IPA, flow rate 1 mLmin^{-1}, 211 nm, 30 °C) t_R (*S*): 7.2 min, t_R (*R*): 9.5 min, 96:4 er

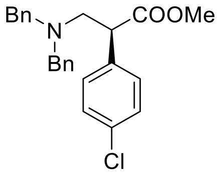

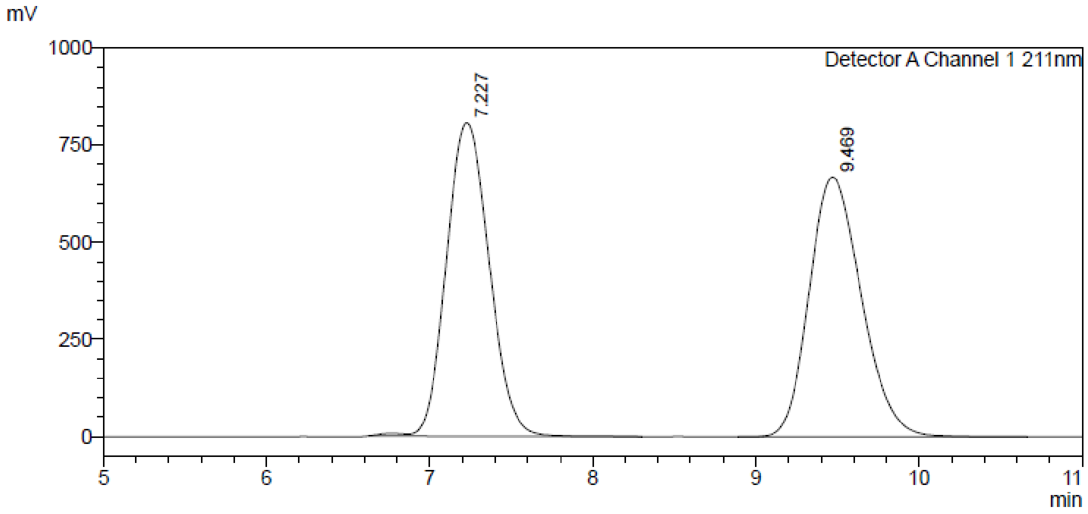

Peak#	Ret. Time	Area%
1	7.227	49.811
2	9.469	50.189
Total		100.000

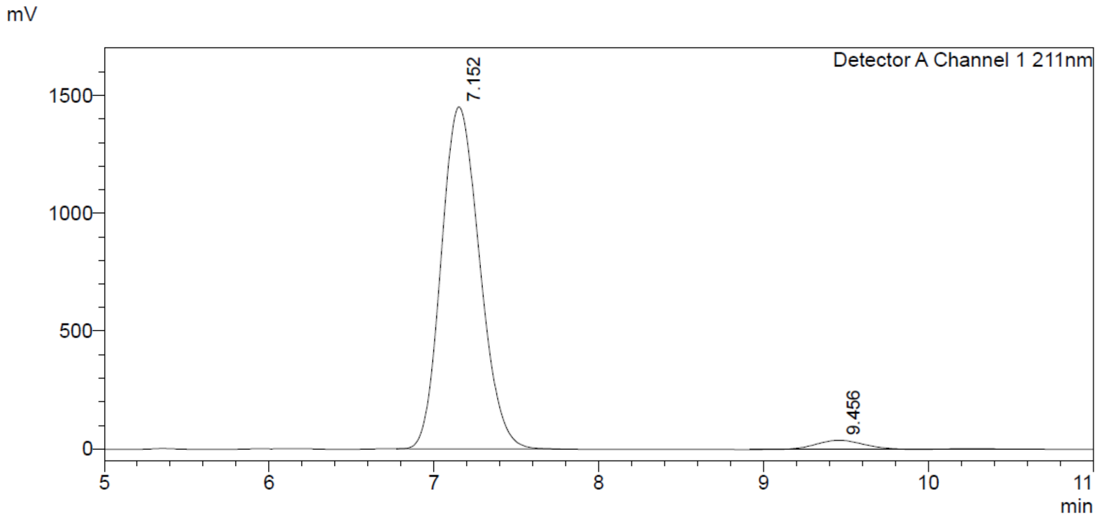

Detector A Channel 1 211nm

Peak#	Ret. Time	Area%
1	7.152	96.342
2	9.456	3.658
Total		100.000

HPLC data for 15

Chiralcel OD-H (98.5:1.5 hexane:IPA, flow rate 1 mLmin^{-1}, 211 nm, 30 °C) t_R (*S*): 7.0 min, t_R (*R*): 9.1 min, 96:4 er

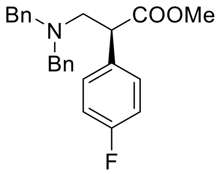

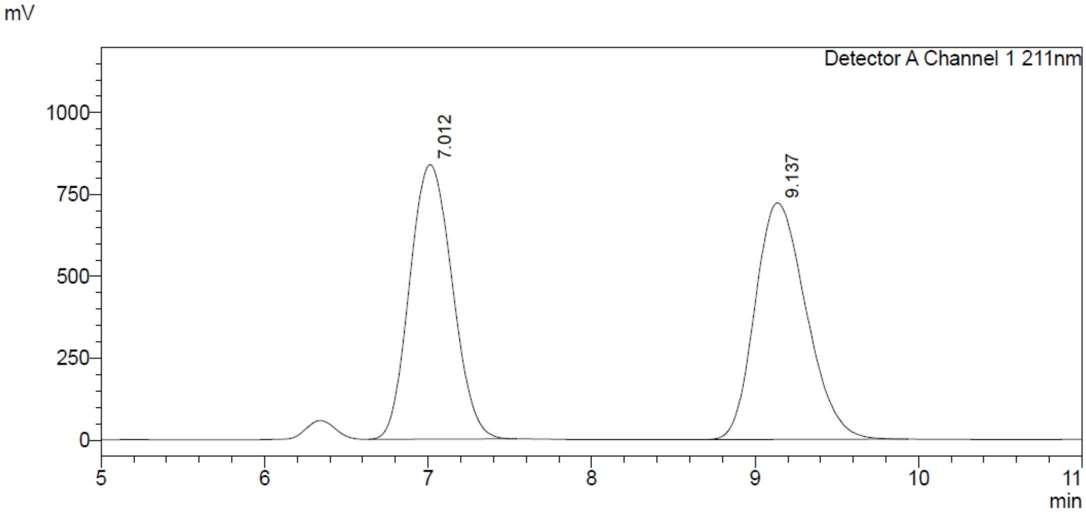

Peak#	Ret. Time	Area%
1	7.012	49.147
2	9.137	50.853
Total		100.000

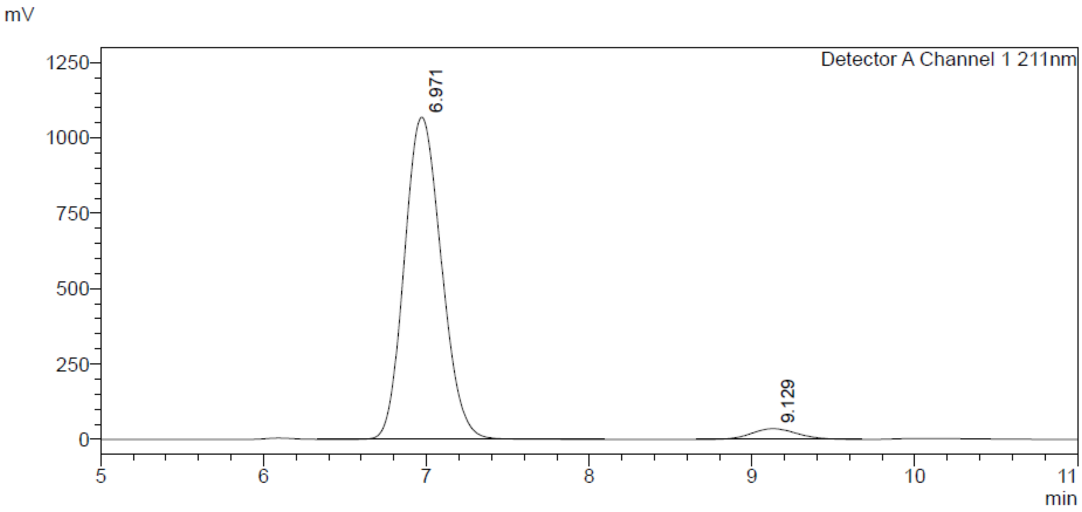

<Peak Table>

Detector A Channel 1 211nm

Peak#	Ret. Time	Area%
1	6.971	96.053
2	9.129	3.947
Total		100.000

HPLC data for 16

Chiralcel OD-H (98.5:1.5 hexane:IPA, flow rate 1 mLmin^{-1}, 211 nm, 30 °C) t_R (*S*): 7.1 min, t_R (*R*): 9.6 min, 96:4 er

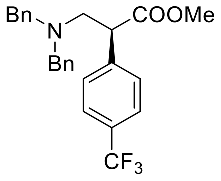

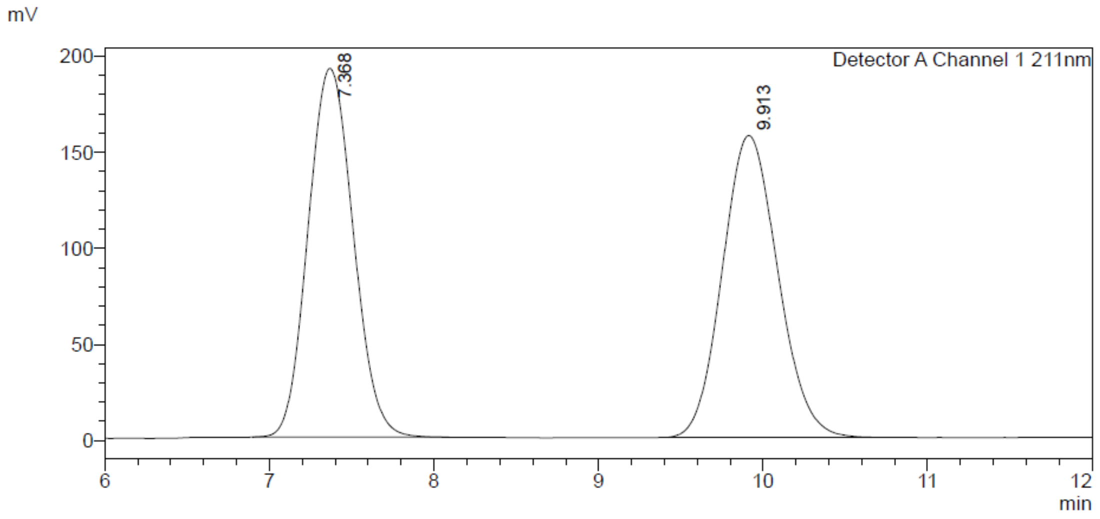

Peak#	Ret. Time	Area%
1	7.368	49.834
2	9.913	50.166
Total		100.000

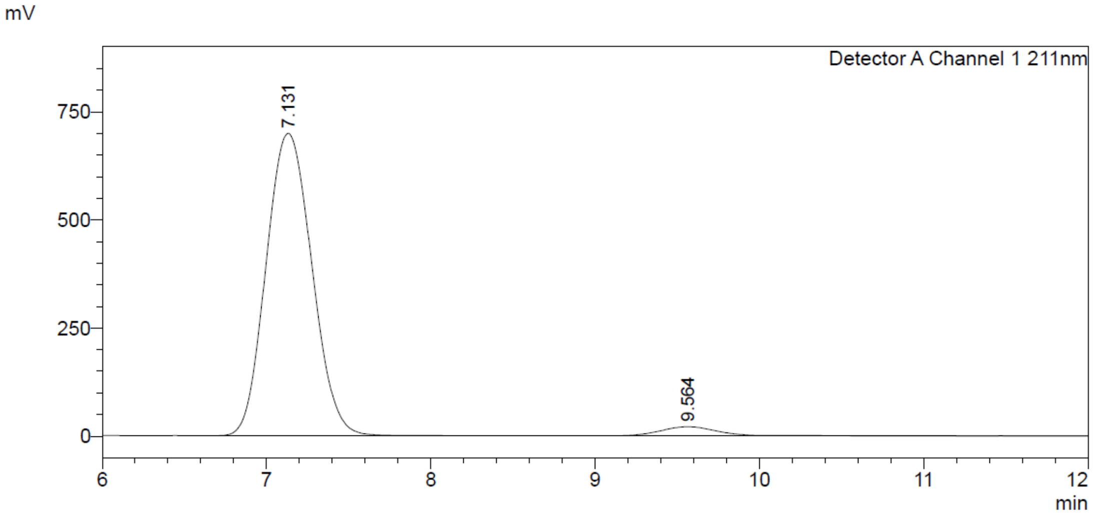

<Peak Table>

Detector A Channel 1 211nm

Peak#	Ret. Time	Area%
1	7.131	96.352
2	9.564	3.648
Total		100.000

HPLC data for 17

Chiralcel OD-H (95:5 hexane:IPA, flow rate 1 mLmin^{-1}, 211 nm, 30 °C) t_R (*S*): 12.1 min, t_R (*R*): 15.9 min, 64:36 er

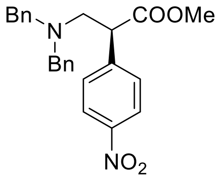

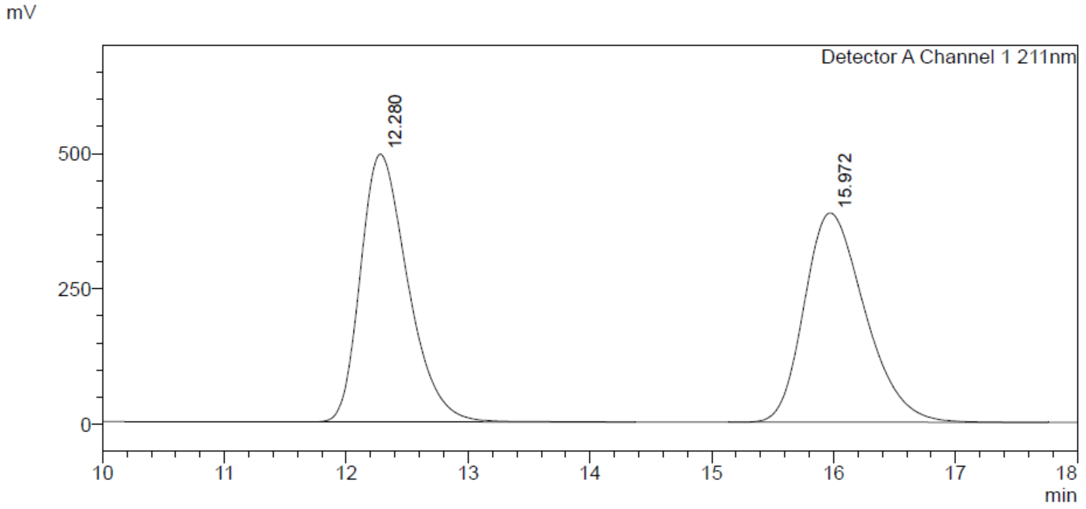

<Peak Table>

Detector A Channel 1 211nm

Peak#	Ret. Time	Area%
1	12.280	49.948
2	15.972	50.052
Total		100.000

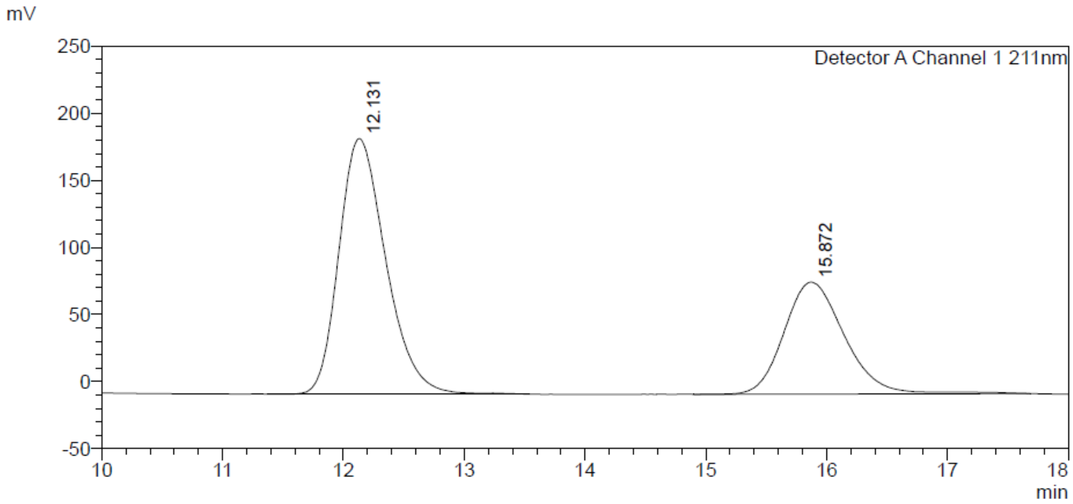

<Peak Table>

Detector A Channel 1 211nm

Peak#	Ret. Time	Area%
1	12.131	63.825
2	15.872	36.175
Total		100.000

HPLC data for 18

Chiralcel AD-H (97.5:2.5 hexane:IPA, flow rate 1 mLmin^{-1}, 211 nm, 30 °C) t_R (*S*): 18.5 min, t_R (*R*): 26.6 min, 96:4 er

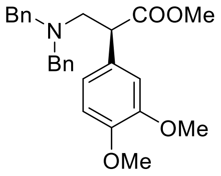

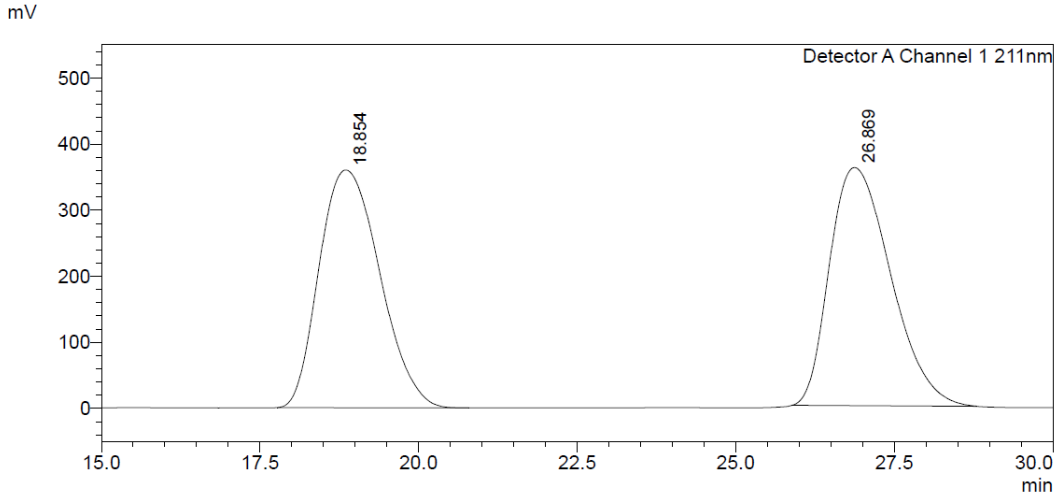

Detector A Channel 1 211nm

Peak#	Ret. Time	Area%
1	18.854	49.698
2	26.869	50.302
Total		100.000

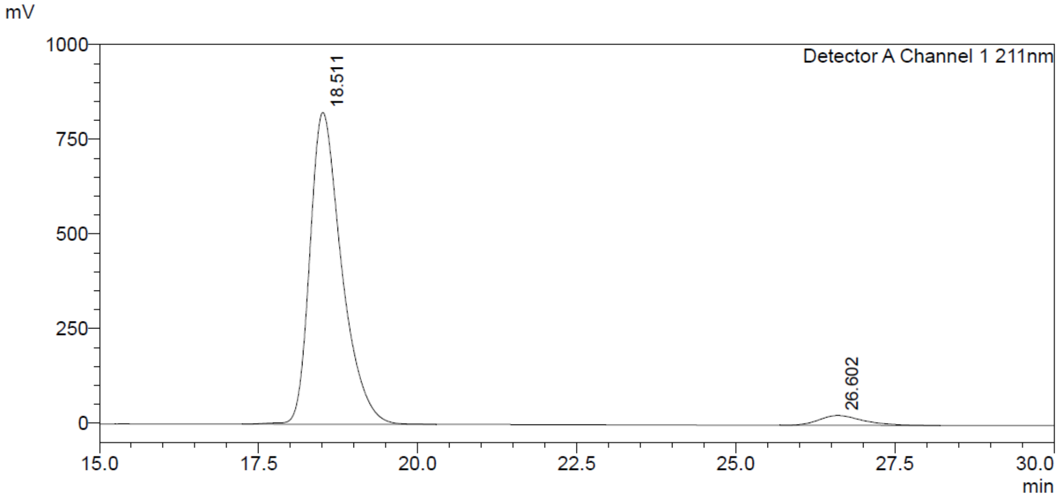

Detector A Channel 1 211nm

Peak#	Ret. Time	Area%
1	18.511	95.845
2	26.602	4.155
Total		100.000

HPLC data for 19

Chiralcel OD-H (98.5:1.5 hexane:IPA, flow rate 1.00 mL.min[-1], 211 nm, 30 ℃): tR (*S*): 10.9 min, tR (*R*): 13.4 min, 96:4 er

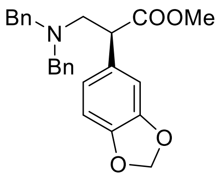

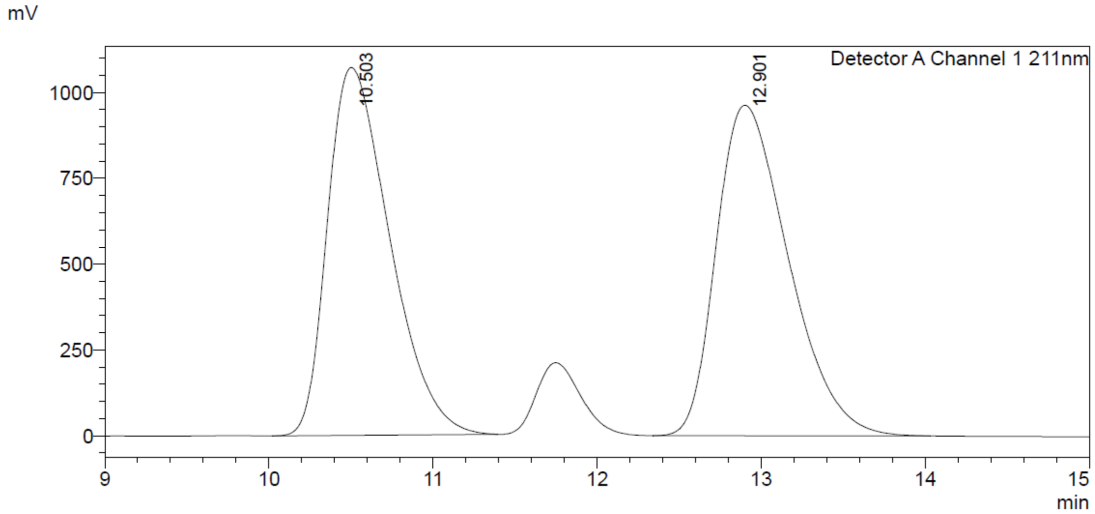

Peak#	Ret. Time	Area%
1	10.503	48.979
2	12.901	51.021
Total		100.000

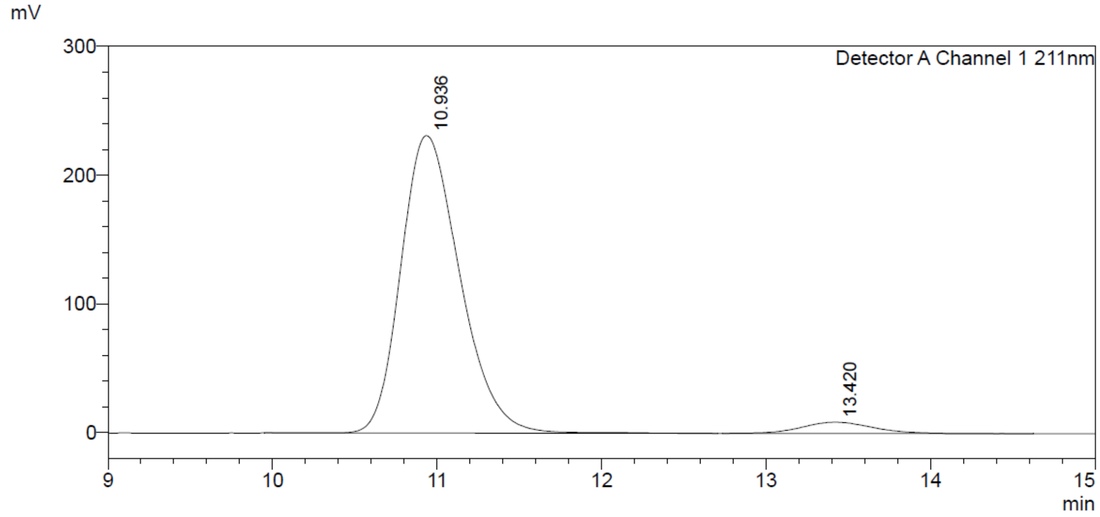

Detector A Channel 1 211nm

Peak#	Ret. Time	Area%
1	10.936	95.682
2	13.420	4.318
Total		100.000

HPLC data for 20

Chiralcel OD-H (97:3 hexane:IPA, flow rate 1 mLmin^{-1}, 211 nm, 30 °C) t_R (*S*): 6.9 min, t_R (*R*): 8.5 min, 95:5 er

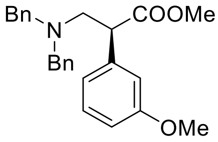

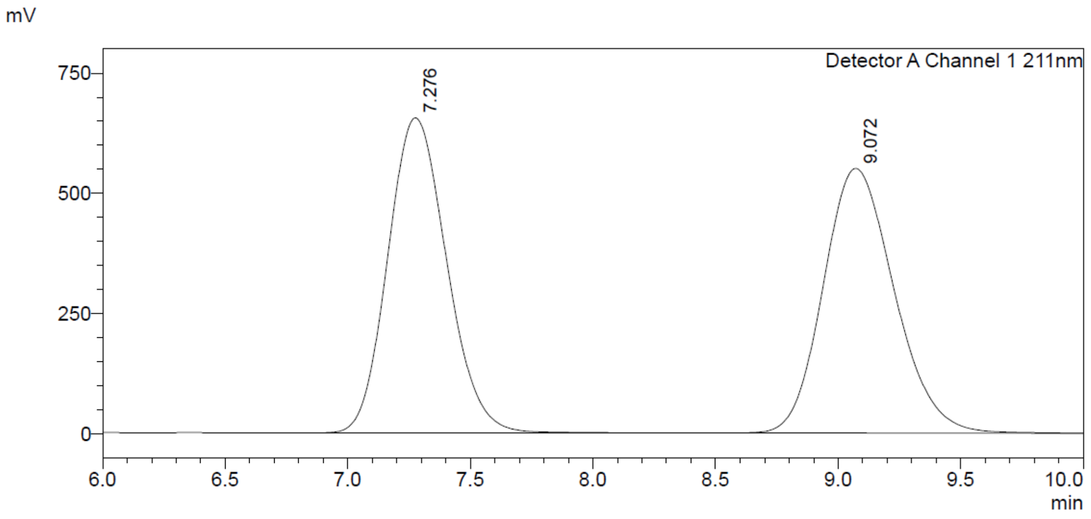

Peak#	Ret. Time	Area%
1	7.276	49.662
2	9.072	50.338
Total		100.000

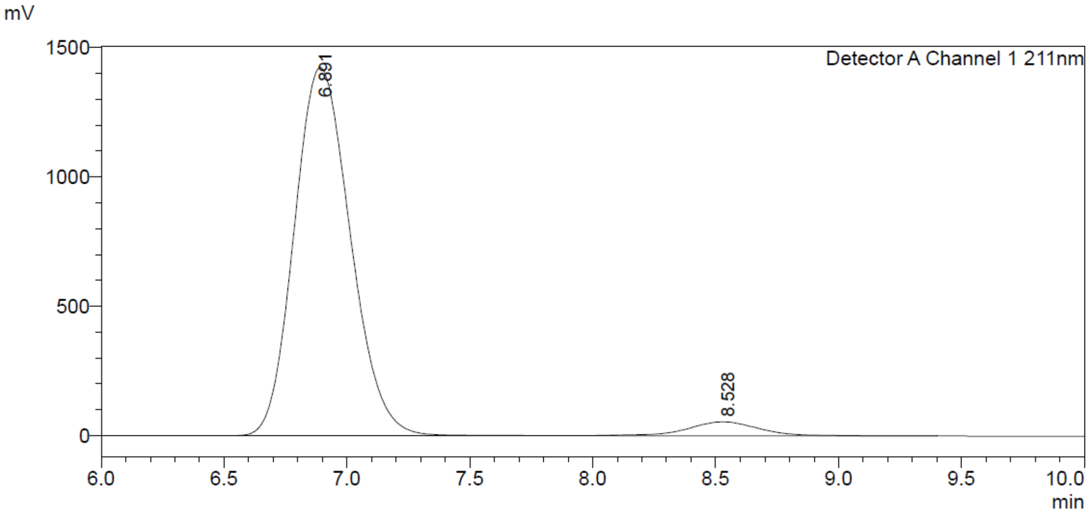

Peak#	Ret. Time	Area%
1	6.891	95.324
2	8.528	4.676
Total		100.000

HPLC data for 21

Chiralcel OD-H (98.5:1.5 hexane:IPA, flow rate 1 mLmin^{-1}, 211 nm, 30 °C) t_R (*S*): 7.2 min, t_R (*R*): 8.8 min, 96:4 er

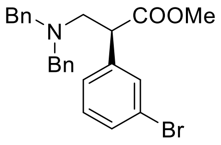

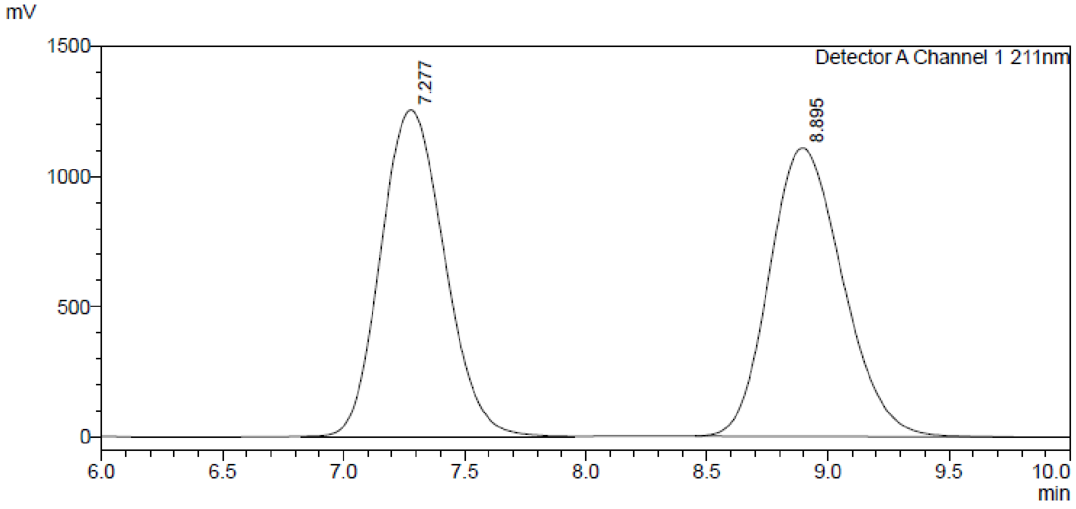

Peak#	Ret. Time	Area%
1	7.277	49.594
2	8.895	50.406
Total		100.000

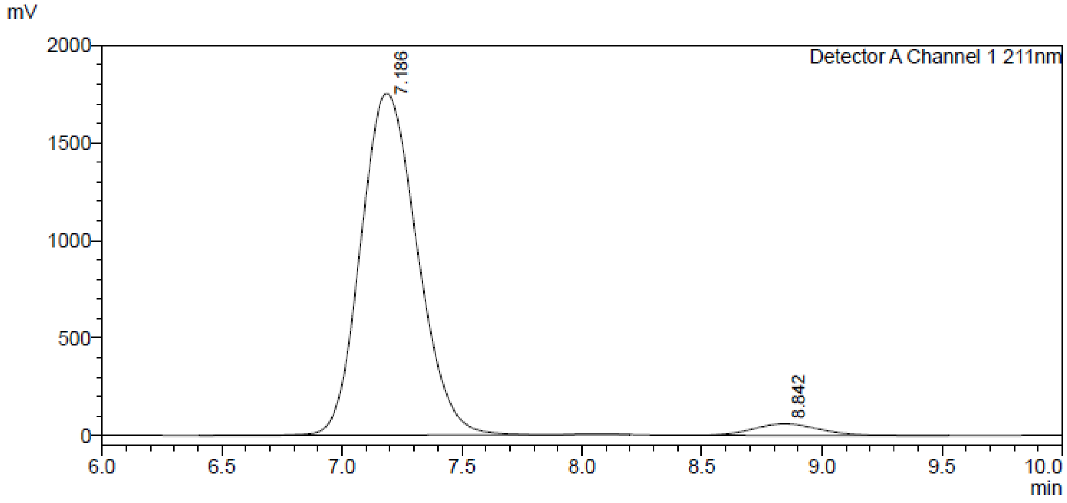

<Peak Table>

Detector A Channel 1 211nm

Peak#	Ret. Time	Area%
1	7.186	96.263
2	8.842	3.737
Total		100.000

HPLC data for 22

Chiralcel OD-H (95:5 hexane:IPA, flow rate 1 mLmin^{-1}, 211 nm, 30 °C) t_R (*S*): 28.9 min, t_R (*R*): 30.6 min, 94:6 er

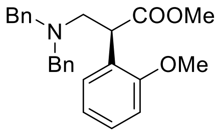

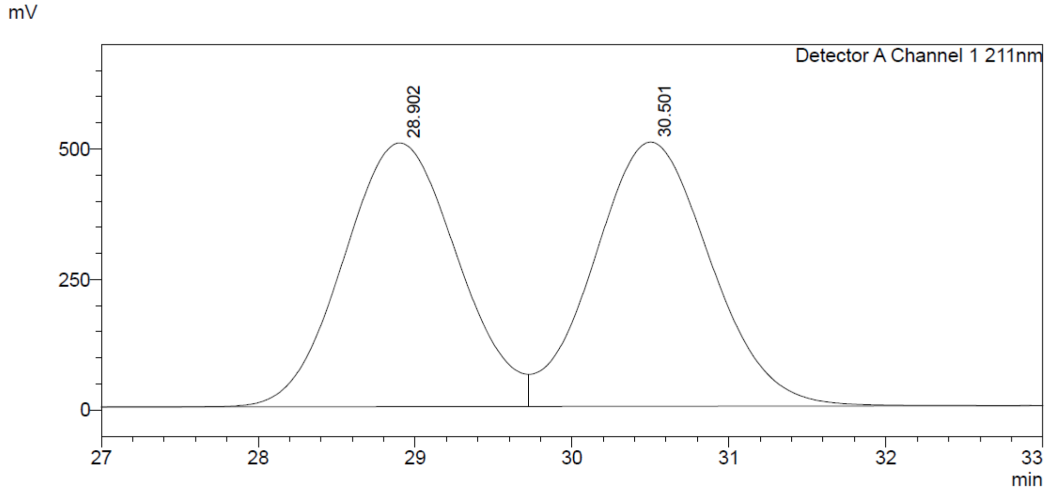

<Peak Table>

Detector A Channel 1 211nm

Peak#	Ret. Time	Area%
1	28.902	49.405
2	30.501	50.595
Total		100.000

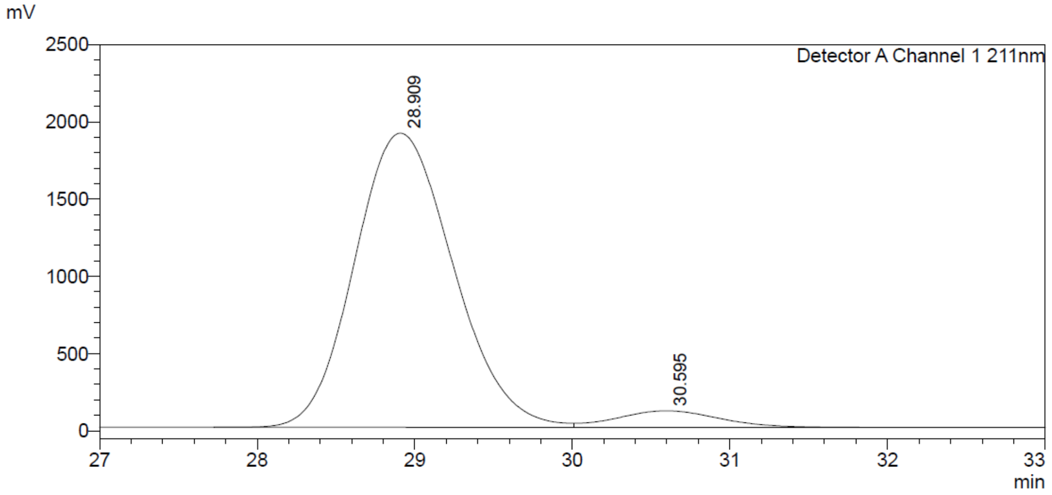

<Peak Table>

Detector A Channel 1 211nm

Peak#	Ret. Time	Area%
1	28.909	94.506
2	30.595	5.494
Total		100.000

HPLC data for 23

Chiralcel OD-H (97:3 hexane:IPA, flow rate 1 mLmin^{-1}, 211 nm, 30 °C) t_R (*S*): 5.4 min, t_R (*R*): 6.0 min, 95:5 er

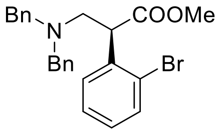

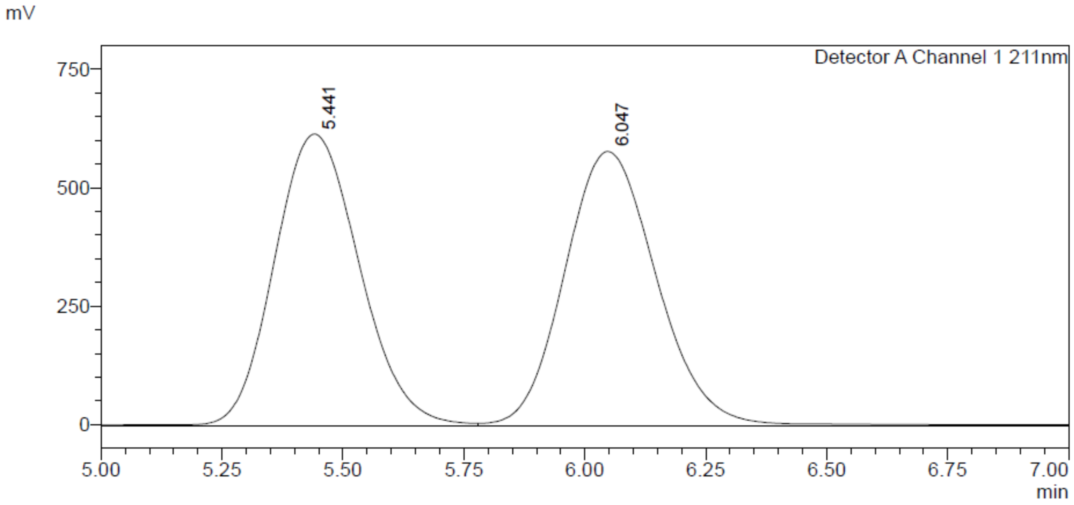

<Peak Table>

Detector A Channel 1 211nm

Peak#	Ret. Time	Area%
1	5.441	49.253
2	6.047	50.747
Total		100.000

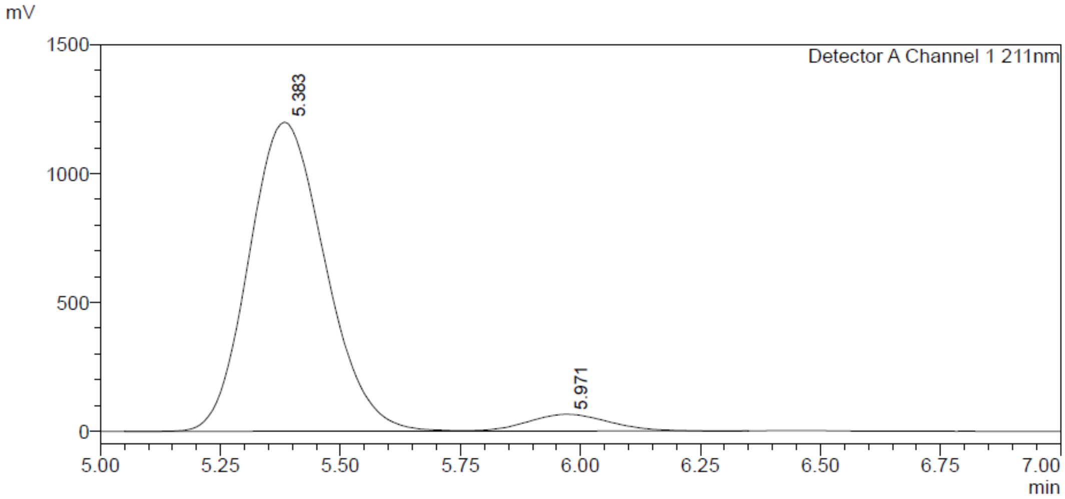

<Peak Table>

Detector A Channel 1 211nm

Peak#	Ret. Time	Area%
1	5.383	94.545
2	5.971	5.455
Total		100.000

HPLC data for 24

Chiralpak IC (99:1 hexane:IPA, flow rate 1 mLmin^{-1}, 211 nm, 30 °C) t_R (*S*): 7.9 min, t_R (*R*): 10.4 min, 94:6 er

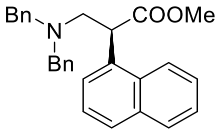

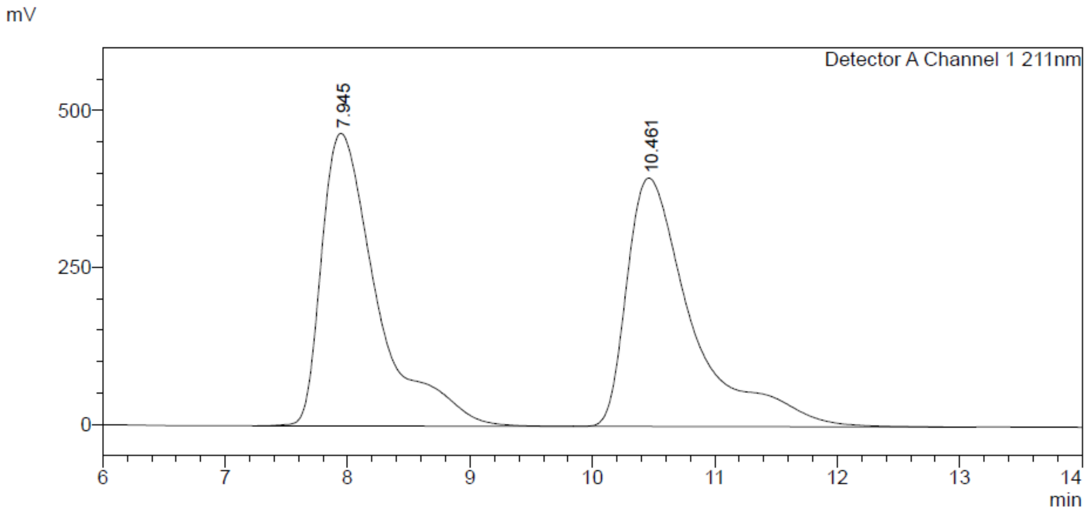

<Peak Table>

Detector A Channel 1 211nm

Peak#	Ret. Time	Area%
1	7.945	49.942
2	10.461	50.058
Total		100.000

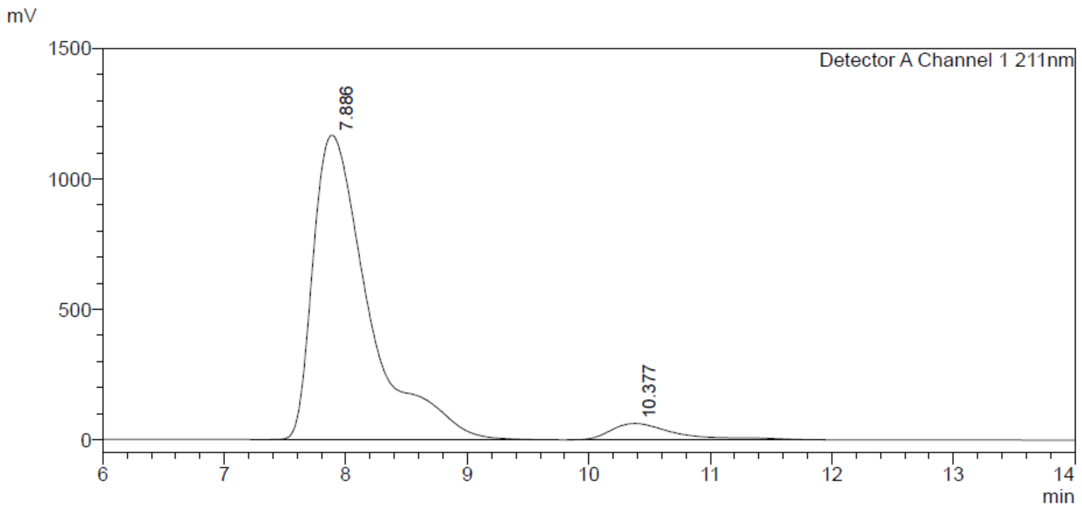

<Peak Table>

Detector A Channel 1 211nm

Peak#	Ret. Time	Area%
1	7.886	94.106
2	10.377	5.894
Total		100.000

HPLC data for 25

Chiralcel OD-H (98.5:1.5 hexane:IPA, flow rate 1 mLmin^{-1}, 211 nm, 30 °C) t_R (*S*): 9.2 min, t_R (*R*): 11.7 min, 96:4 er

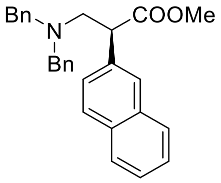

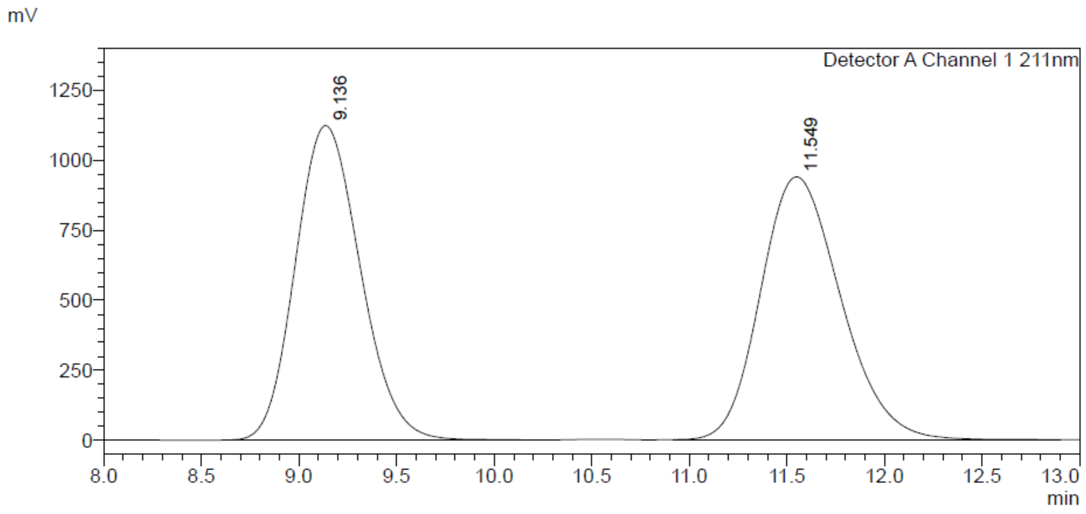

<Peak Table>

Detector A Channel 1 211nm

Peak#	Ret. Time	Area%
1	9.136	49.206
2	11.549	50.794
Total		100.000

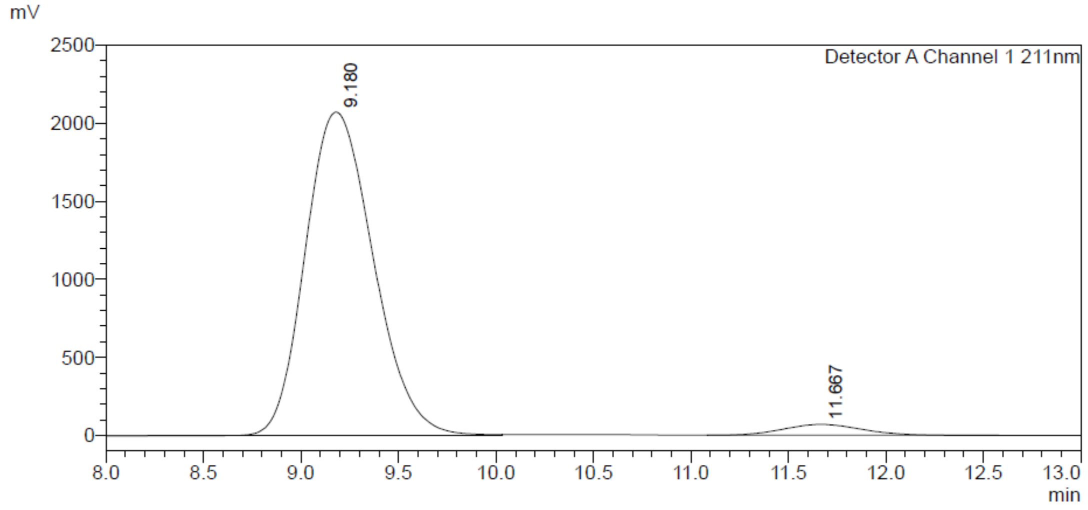

<Peak Table>

Detector A Channel 1 211nm

Peak#	Ret. Time	Area%
1	9.180	96.257
2	11.667	3.743
Total		100.000

HPLC data for 26

Chiralcel OD-H (98.5:1.5 hexane:IPA, flow rate 1 mLmin^{-1}, 211 nm, 30 °C) t$_R$ (*S*): 7.7 min, t$_R$ (*R*): 8.7 min, 94:6 er

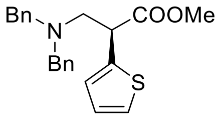

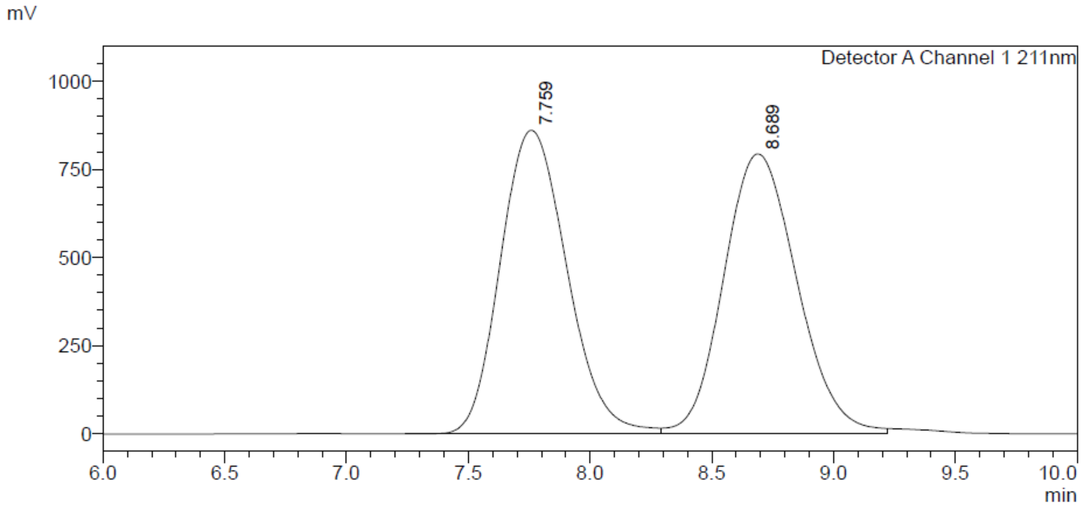

<Peak Table>

Detector A Channel 1 211nm

Peak#	Ret. Time	Area%
1	7.759	49.624
2	8.689	50.376
Total		100.000

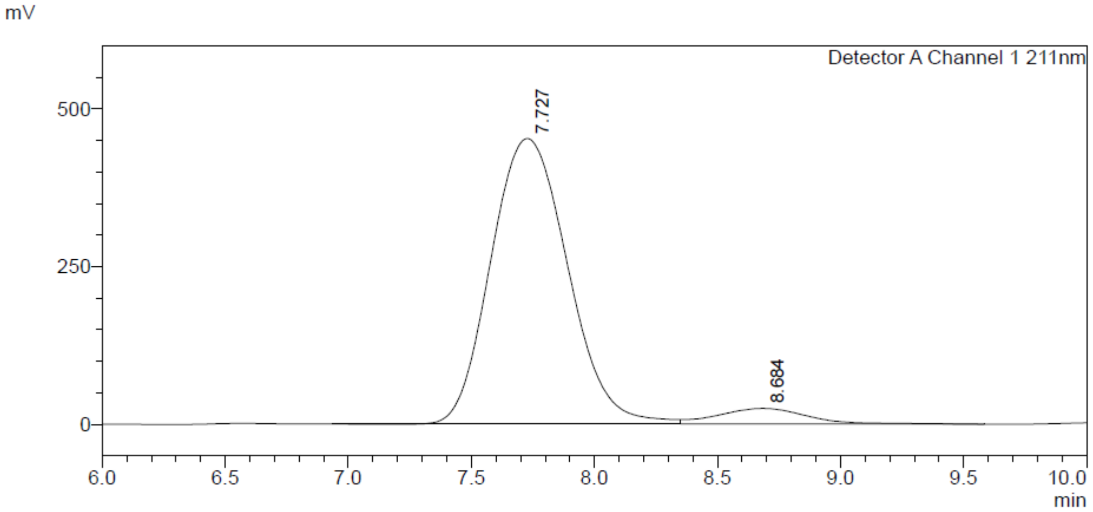

Peak#	Ret. Time	Area%
1	7.727	94.051
2	8.684	5.949
Total		100.000

HPLC data for 27

Chiralcel OJ-H (97:3 hexane:IPA, flow rate 1 mLmin^{-1}, 220 nm, 30 °C) t_R (*S*): 11.5 min, t_R (*R*): 15.9 min, 94:6 er

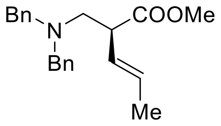

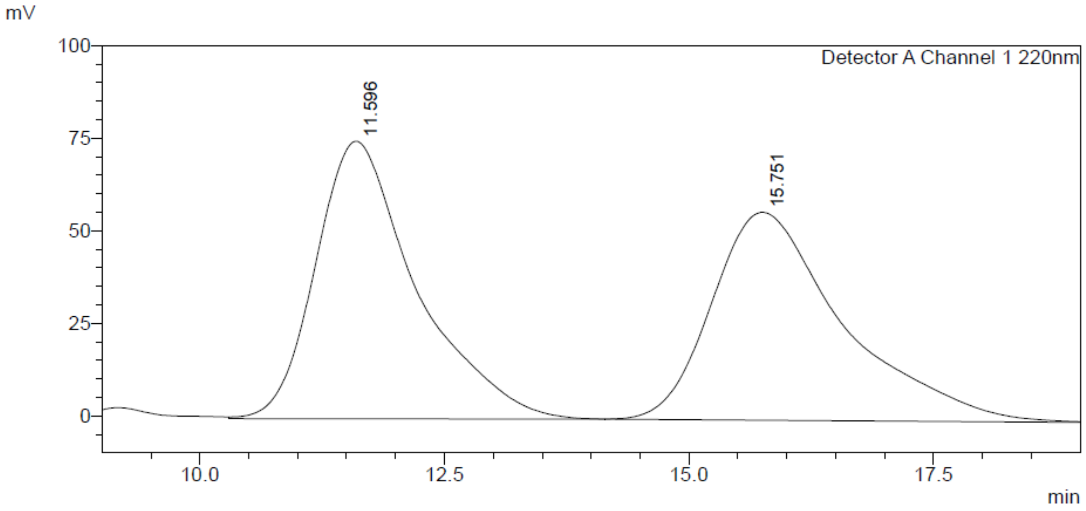

<Peak Table>

Detector A Channel 1 220nm

Peak#	Ret. Time	Area%
1	11.596	50.591
2	15.751	49.409
Total		100.000

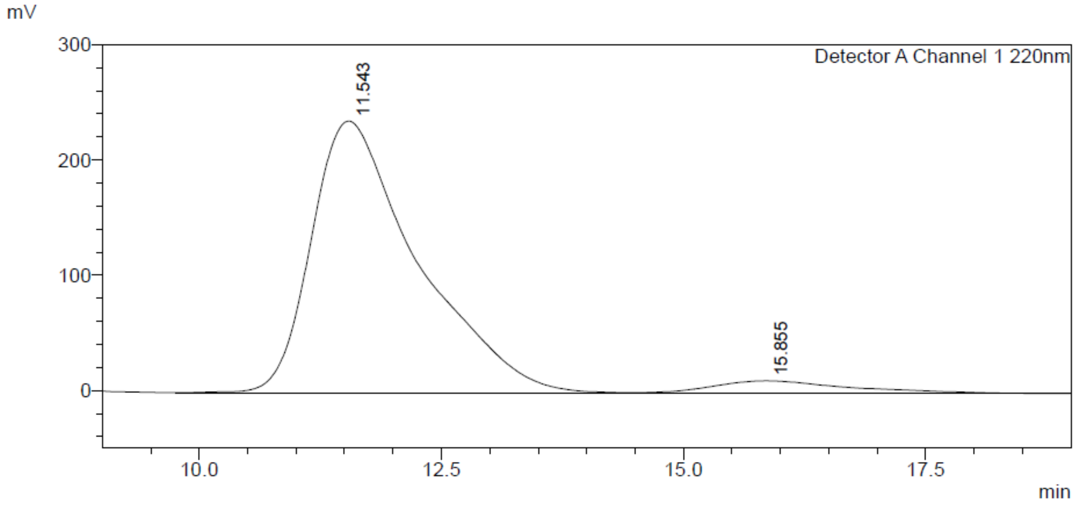

<Peak Table>

Detector A Channel 1 220nm

Peak#	Ret. Time	Area%
1	11.543	94.244
2	15.855	5.756
Total		100.000

HPLC data for 28

Chiralcel OD-H (98.5:1.5 hexane:IPA, flow rate 1 mLmin^{-1}, 211 nm, 30 °C) t_R (*S*): 6.7 min, t_R (*R*): 8.0 min, 95:5 er

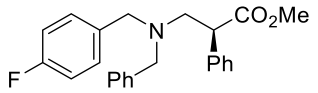

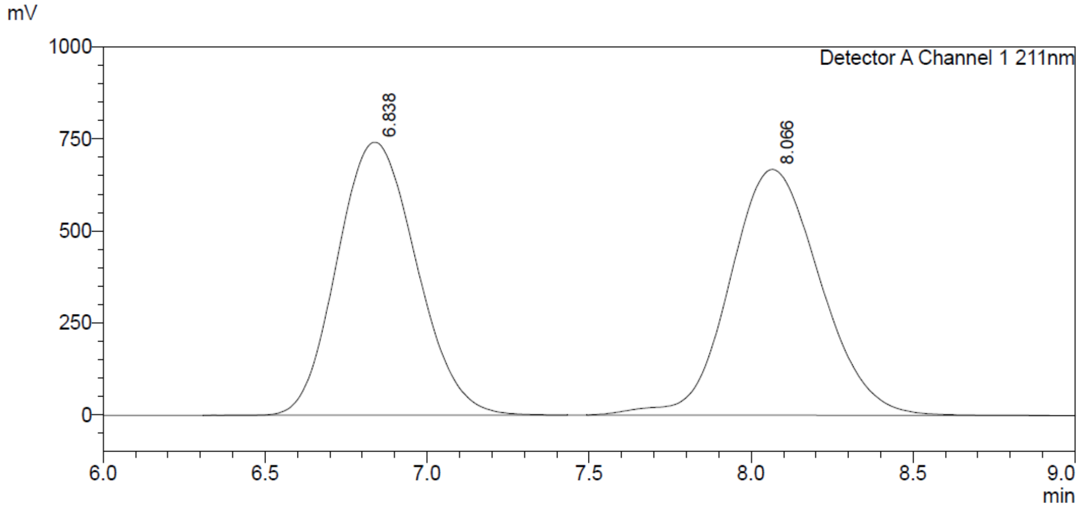

Peak#	Ret. Time	Area%
1	6.838	49.268
2	8.066	50.732
Total		100.000

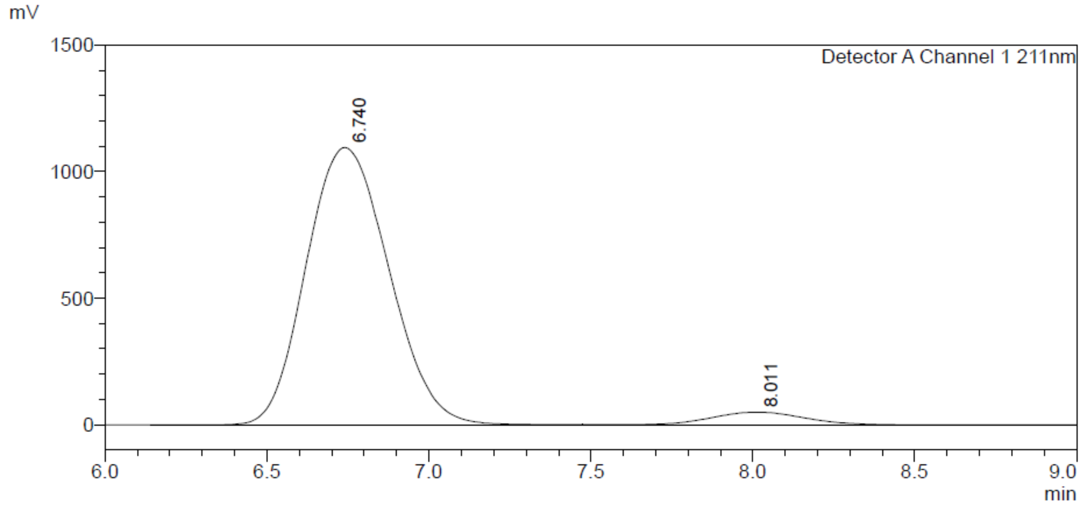

Peak#	Ret. Time	Area%
1	6.740	95.250
2	8.011	4.750
Total		100.000

HPLC data for 29

Chiralcel OD-H (98.5:1.5 hexane:IPA, flow rate 1 $mLmin^{-1}$, 211 nm, 30 °C) t_R (*S*): 7.7 min, t_R (*R*): 9.2 min, 95:5 er

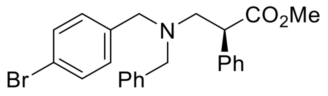

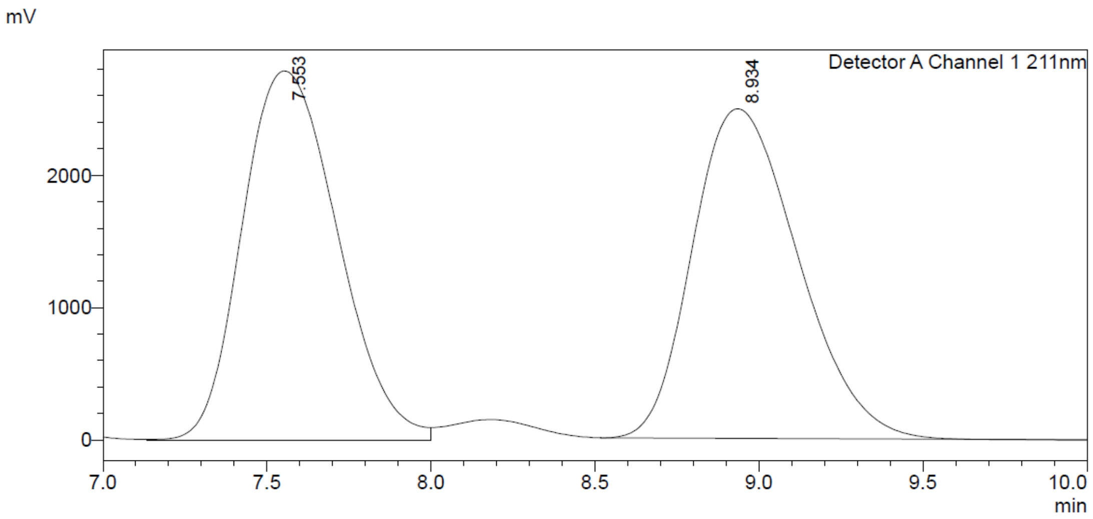

Peak#	Ret. Time	Area%
1	7.553	50.330
2	8.934	49.670
Total		100.000

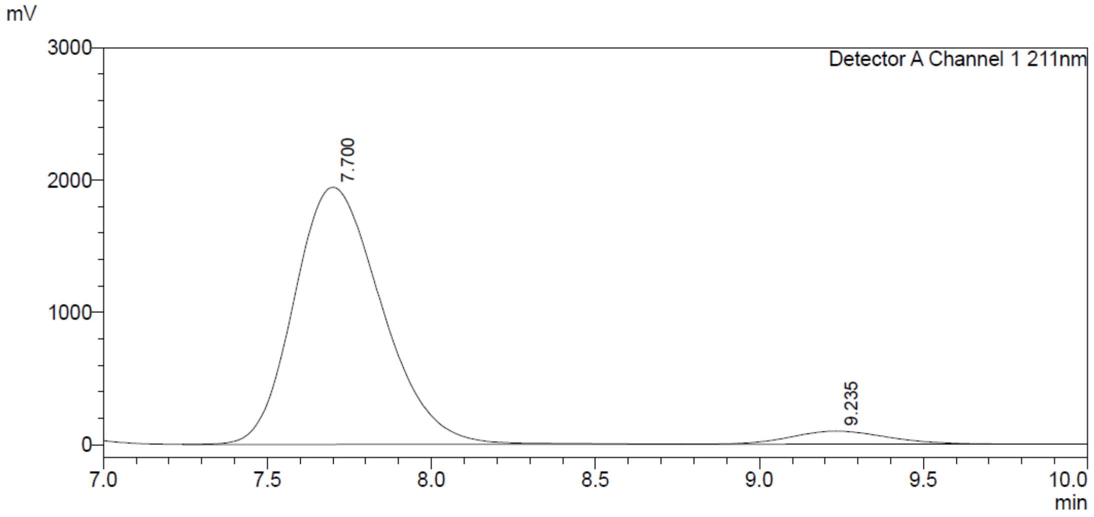

Peak#	Ret. Time	Area%
1	7.700	94.630
2	9.235	5.370
Total		100.000

HPLC data for 30

Chiralcel OD-H (98.5:1.5 hexane:IPA, flow rate 1 mLmin^{-1}, 211 nm, 30 °C) t_R (*S*): 9.1 min, t_R (*R*): 10.4 min, 95:5 er

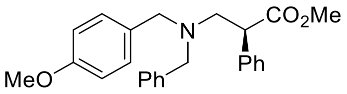

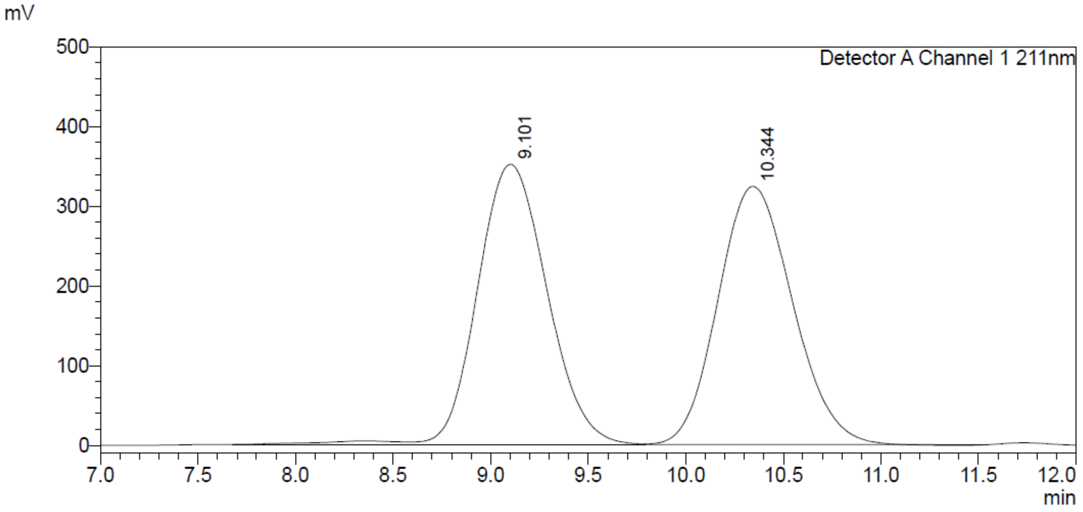

Peak#	Ret. Time	Area%
1	9.101	50.387
2	10.344	49.613
Total		100.000

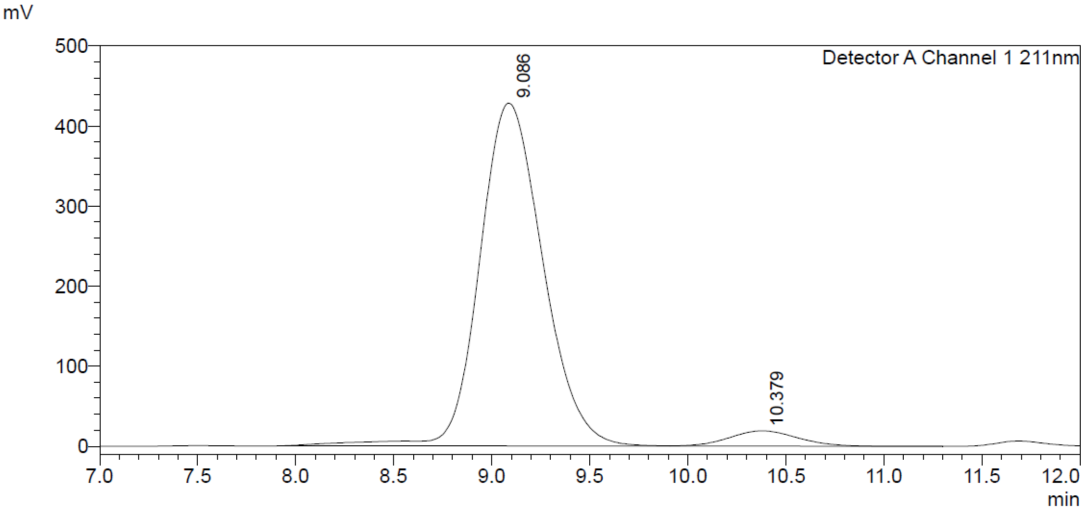

Peak#	Ret. Time	Area%
1	9.086	95.370
2	10.379	4.630
Total		100.000

HPLC data for 31

Chiralcel OD-H (98.5:1.5 hexane:IPA, flow rate 0.3 mLmin^{-1}, 211 nm, 30 °C) t_R (*S*): 13.4 min, t_R (*R*): 14.7 min, 94:6 er

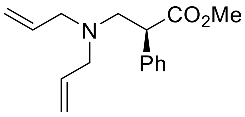

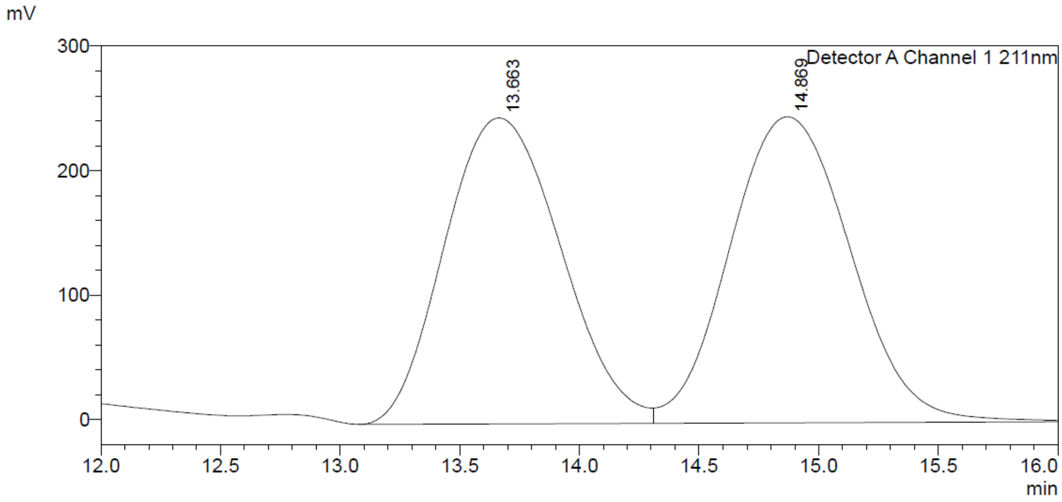

Peak#	Ret. Time	Area%
1	13.663	48.989
2	14.869	51.011
Total		100.000

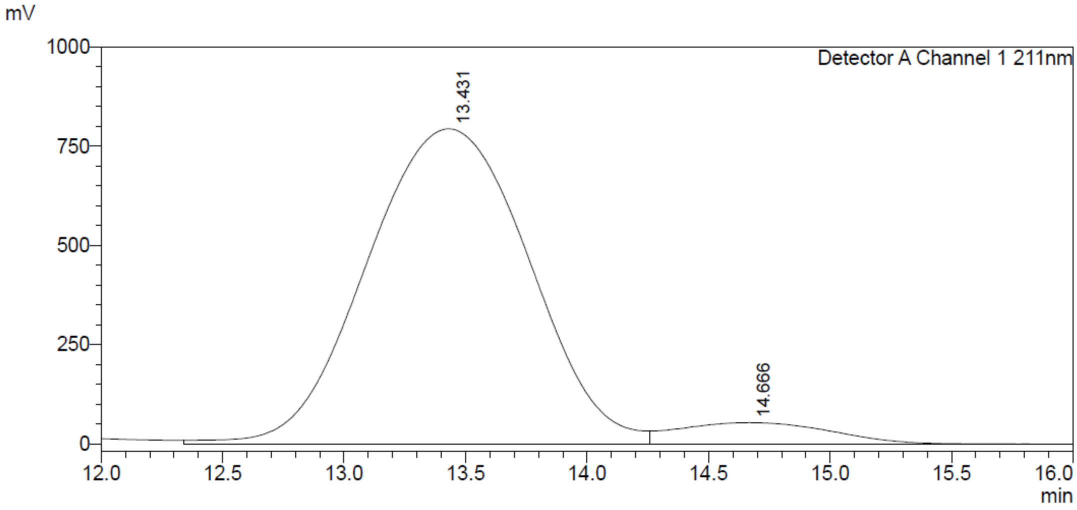

Peak#	Ret. Time	Area%
1	13.431	93.932
2	14.666	6.068
Total		100.000

HPLC data for 39

Chiralcel OD-H (98.5:1.5 hexane:IPA, flow rate 1 mLmin^{-1}, 211 nm, 30 °C) t_R (*S*): 7.1 min, t_R (*R*): 8.6 min, 93:7 er

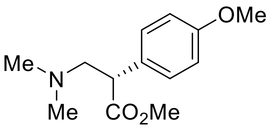

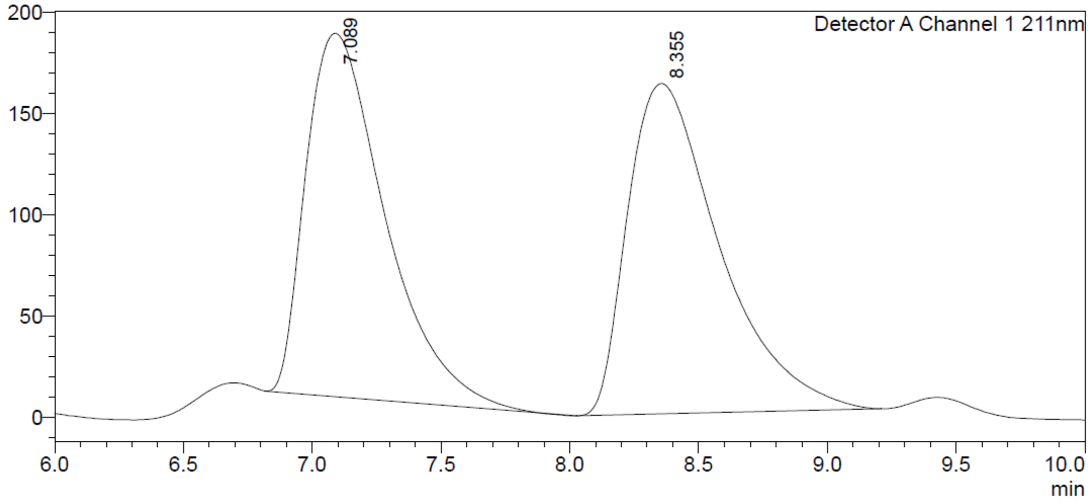

Peak#	Ret. Time	Area%
1	7.089	48.617
2	8.355	51.383
Total		100.000

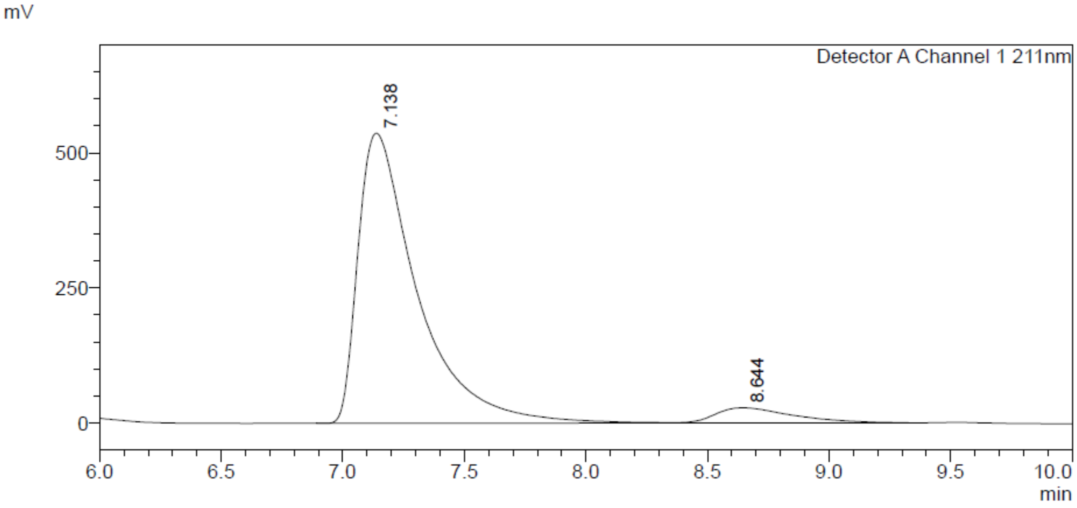

<Peak Table>

Detector A Channel 1 211nm

Peak#	Ret. Time	Area%
1	7.138	93.496
2	8.644	6.504
Total		100.000

HPLC data for 4

Chiralpak AD-H (98.9:1:0.1 hexane:IPA:TEA, flow rate 0.4 mLmin^{-1}, 274 nm, 30 °C) t_R (R): 22.8 min, t_R (S): 27.2 min, 1:99 er

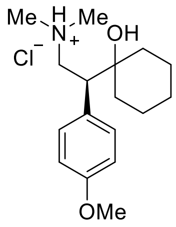

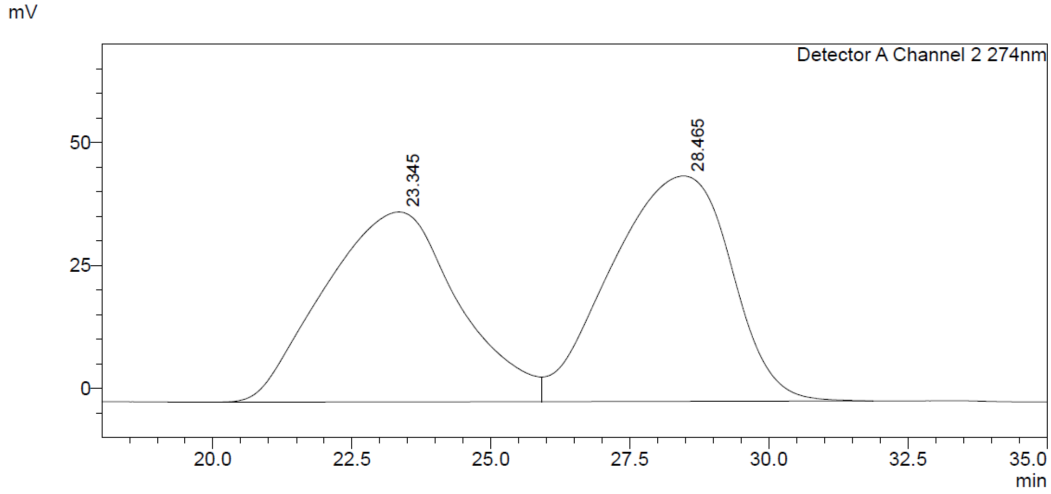

<Peak Table>

Detector A Channel 2 274nm

Peak#	Ret. Time	Area%
1	23.345	48.490
2	28.465	51.510
Total		100.000

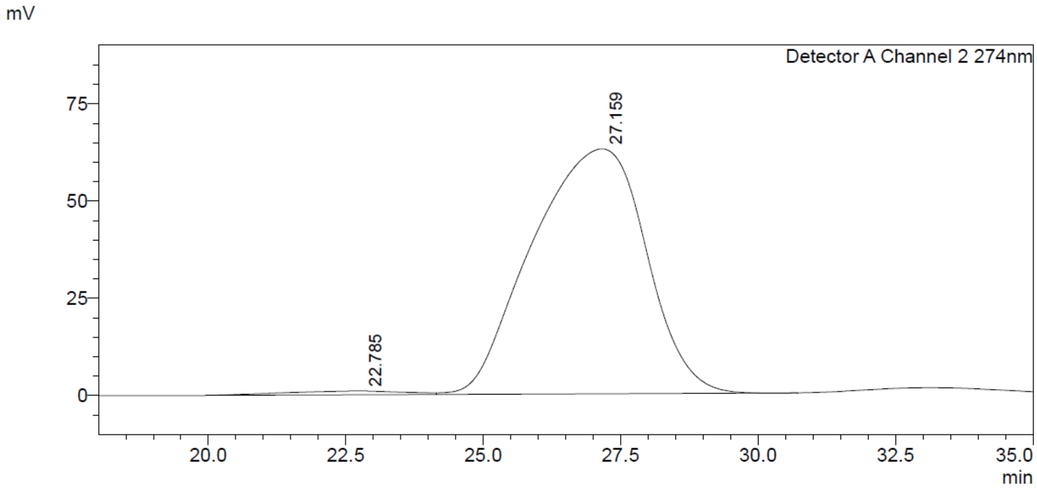

Detector A Channel 2 274nm

Peak#	Ret. Time	Area%
1	22.785	1.542
2	27.159	98.458
Total		100.000

HPLC data for 40

Chiralcel OD-H (96:3:1 hexane:IPA:TEA, flow rate 1 mLmin^{-1}, 274 nm, 30 °C) t_R (*S*): 30.6 min, t_R (*R*): 37.2 min, 95:5 er

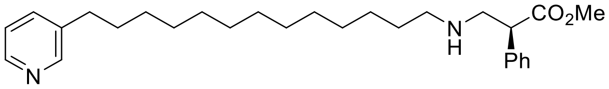

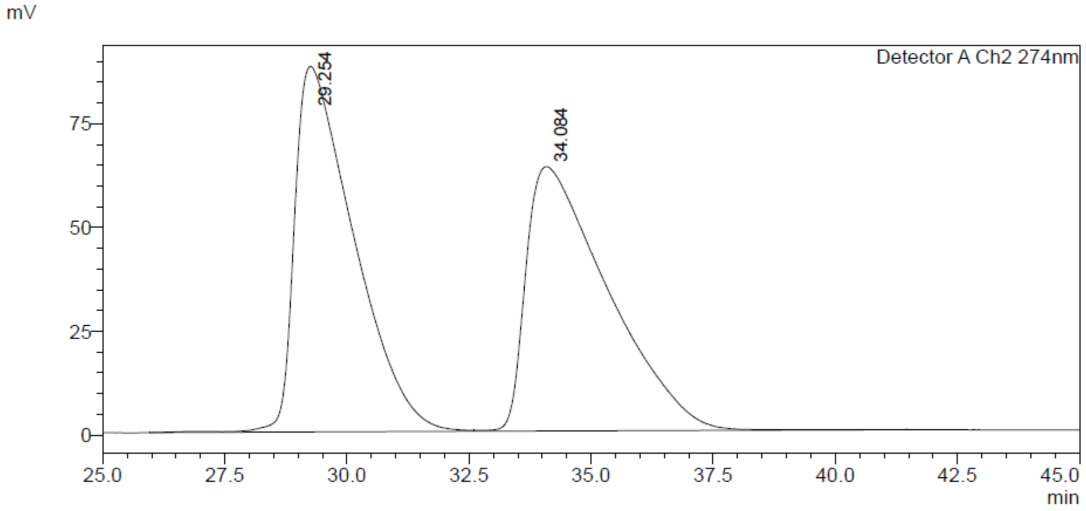

Detector A Channel 2 274nm

Peak#	Ret. Time	Area%
1	29.254	50.044
2	34.084	49.956
Total		100.000

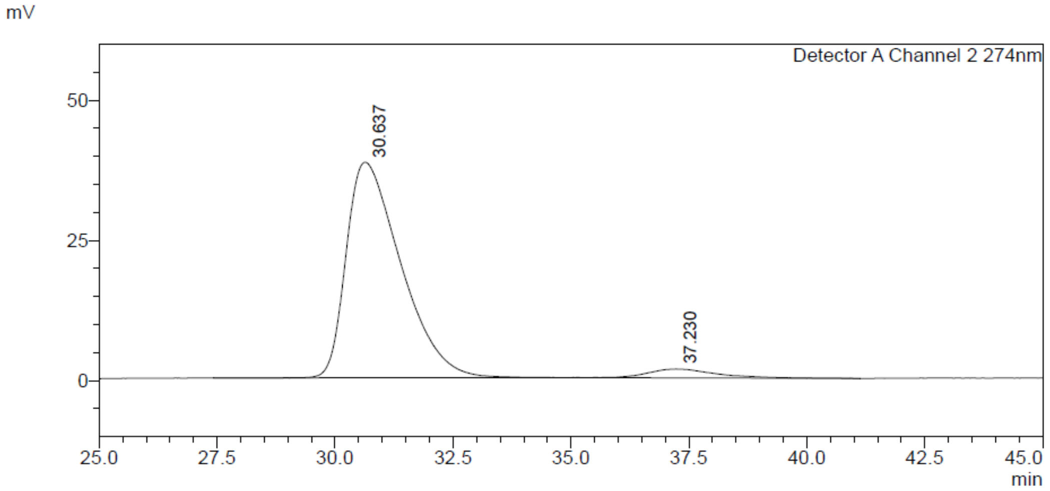

<Peak Table>

Detector A Channel 2 274nm

Peak#	Ret. Time	Area%
1	30.637	95.267
2	37.230	4.733
Total		100.000

HPLC data for 1 (Determined by converting into **40**)

Chiralcel OD-H (96:3:1 hexane:IPA:TEA, flow rate 1 mLmin^{-1}, 274 nm, 30 °C) t_R (*S*): 30.5 min, t_R (*R*): 36.7 min, 93:7 er

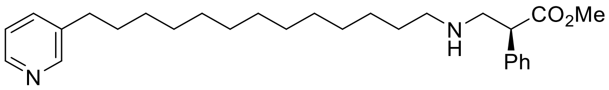

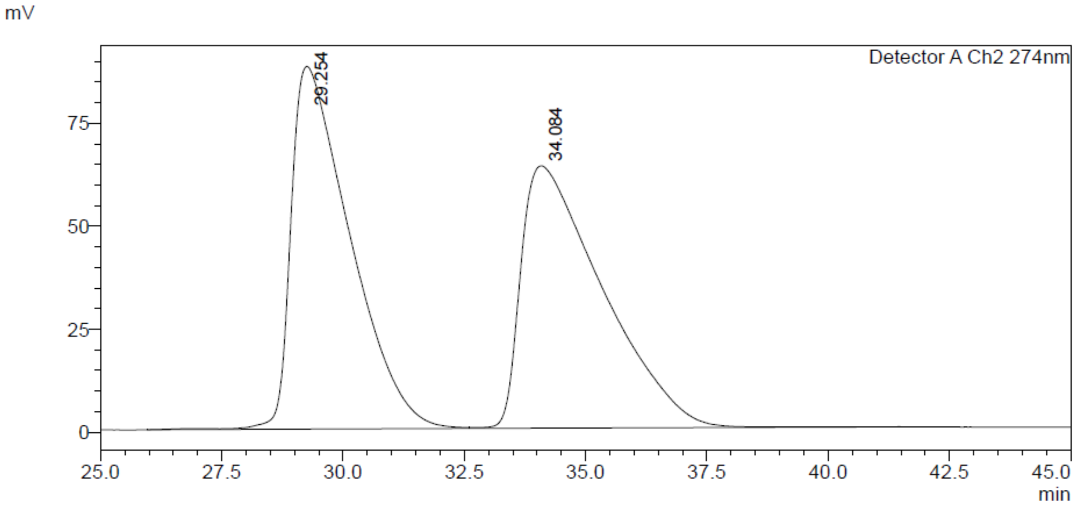

Detector A Channel 2 274nm

Peak#	Ret. Time	Area%
1	29.254	50.044
2	34.084	49.956
Total		100.000

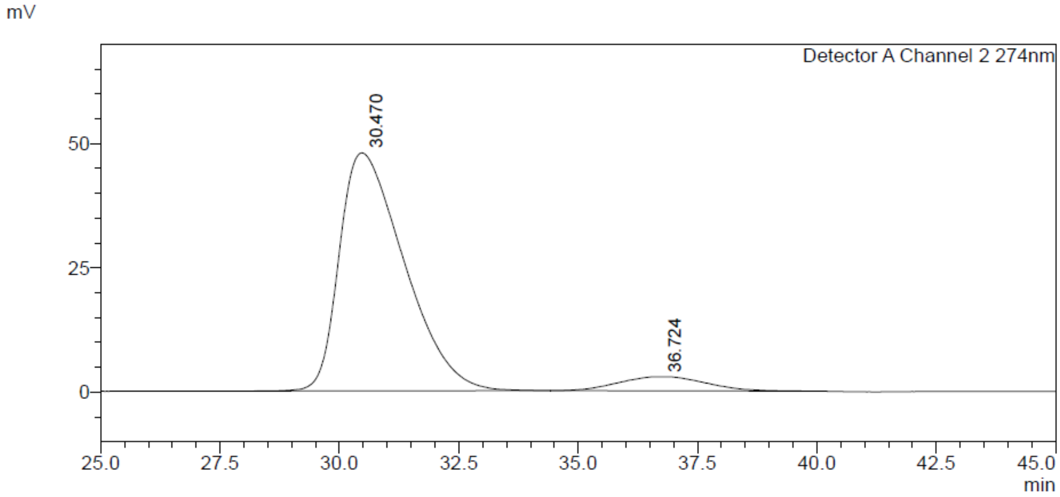

<Peak Table>

Detector A Channel 2 274nm

Peak#	Ret. Time	Area%
1	30.470	92.724
2	36.724	7.276
Total		100.000

谨以此纪念赵峰的学术生涯
